# Enantiopure synthesis of uniform [*n*]cycloparaphenylene-pillar[5]arene multi-macrocycles and their chiroptical properties

**DOI:** 10.1039/d6sc03987a

**Published:** 2026-07-28

**Authors:** Yannik Sebastian Hansmann, Momoko Sugiura, Marcel Mattes, Sascha Feldmann, Birgit Esser

**Affiliations:** a Institute of Organic Chemistry II and Advanced Materials, Ulm University Albert-Einstein-Allee 11 89081 Ulm Germany Birgit.esser@uni-ulm.de; b Laboratory for Energy Materials, École Polytechnique Fédérale de Lausanne (EPFL) 1951 Sion Switzerland

## Abstract

Chiral conjugated nanohoops are attractive based on their potential circularly polarized luminescence (CPL) activity. Using an approach of merging chiral pillar[5]arene (P[5]A) macrocycles with a conjugated nanohoop architecture, we report the enantioselective synthesis of [*n*]cycloparaphenylene ([*n*]CPP)–P[5]A conjugates, [*n*]CPP-PA_n/3_ (*n* = 9, 12 and 15), using a Pt-mediated macrocyclization of enantiopure phenylene-extended P[5]A. The three multi-macrocycles exhibit photophysical behavior closely related to their parent [*n*]CPPs. Their high fluorescence quantum yield (*Φ*_F_ = 56–81%) and circularly polarized luminescence (CPL) with dissymmetry factors *g*_lum_ of 4.2 × 10^−4^–3.0 × 10^−3^ lead to CPL brightnesses *B*_CPL_ of up to 96 M^−1^ cm^−1^ in [15]CPP-PA_3_. This study presents the selective synthesis of statistically disfavored isomers to obtain high-symmetry chiral nanohoops suitable for chiroptical applications.

## Abstract 2

The cyclooligomerization of an enantiopure pillar[5]arene derivative yields three sizes of highly symmetrical chiral nanohoops. The resulting pillarene-cycloparaphenylene multi-macrocycles show size-dependent (chir)optical properties.

## Graphical abstract

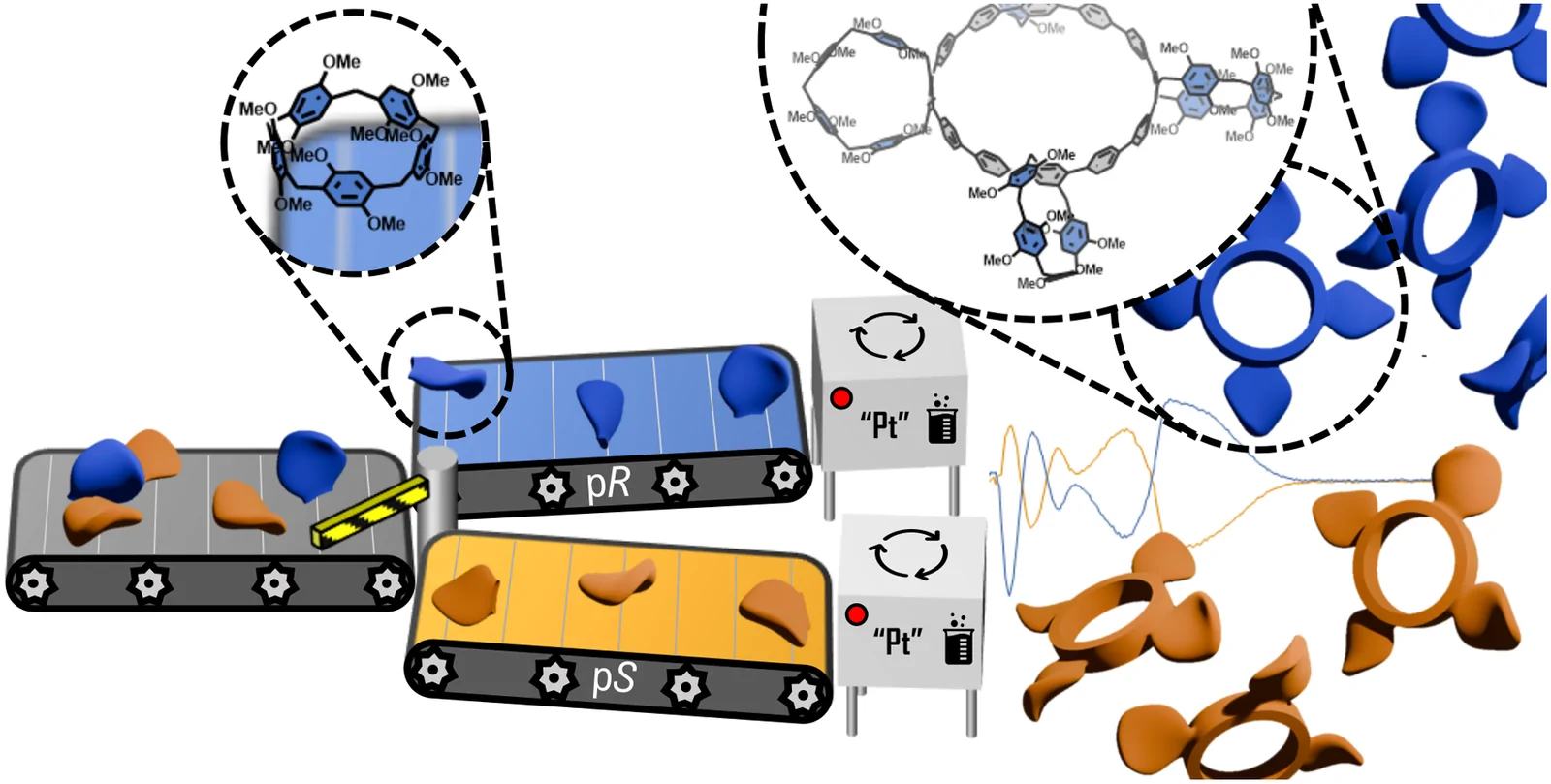

## Introduction

In 2008, two similar yet fundamentally different macrocycle classes were first reported, namely [*n*]cycloparaphenylenes ([*n*]CPPs) and pillar[*n*]arenes (P[*n*]As).^1,2^ In [*n*]CPPs phenylene rings are directly linked in *para*-position to form a nanohoop.^3^ They are fully conjugated and can be highly strained. Meanwhile, P[*n*]As are comprised of *para*-linked dialkoxyphenylene rings separated by CH_2_ groups, relieving strain and breaking conjugation.^4,5^ P[*n*]As have drawn attention because of their facile synthesis and functionalization, their selective guest–recognition properties and their shape persistency.^6,7^ The alkoxy groups give rise to planar chirality, where conformational stability can be obtained through sufficiently large substituents.^8,9^ Functionalization with fluorophores opens up a pathway to circularly-polarized-luminescence (CPL)-active materials.^10–18^ [*n*]CPPs in contrast can be highly emissive, but show no intrinsic chirality.^19^ Through strategic functionalization, chiral derivatives of [*n*]CPPs can be obtained, including chiral double nanohoops, yet there are only few reports on conformationally stable examples and their CPL behavior.^20–26^ Most of these require racemic resolution of the final compound, and since chirality is often introduced during the nanohoop formation step, reports of enantioselective syntheses of chiral [*n*]CPP derivatives are rare.^27,28^ Using enantiopure chiral P[*n*]As as CPP precursors provides an interesting entry. The structural union of [*n*]CPPs and P[*n*]As has been realized before. An example is a bismacrocycle of P[5]A fused with an [8]- or [10]CPP, stable towards racemization.^29,30^ The enantiomers showed high *g*_lum_ values of ∼10^−2^, and the two distinct cavities were able to bind multiple guests. The second report was a trismacrocycle of a central P[6]A fused with two [8]CPPs in opposing positions.^31^ With two chirality planes multiple combinations of configurations are possible, leading to a racemic and a *meso* form, obtained in a statistical 1 : 1 distribution. During preparation of this manuscript, Huang, Hua and coworkers reported on a [9]- and [15]CPP fused with three P[5]As.^32^ In the gold-mediated synthesis of [9]CPP-PA_3_ the authors observed social chiral self-sorting, selectively leading to the p*SSR*/p*RRS* isomers of the resulting tetramacrocycle. Herein, we set out to investigate the platinum-mediated macrocyclization of phenylene-extended P[5]As to furnish the P[5]A-CPP conjugates [*n*]CPP-PA_n/3_. We were able to access three different sizes, namely *n* = 9, 12 and 15, as multi-macrocycles containing four, five and six macrocycles, respectively. By using enantiopure phenylene-extended P[5]As as starting materials, the statistically strongly disfavored, highest-symmetry all-p*R*- and all-p*S*-isomers were enantioselectively formed. The three hoop sizes allowed us to identify size-dependent trends in their photophysical and chiroptical properties.

## Results and discussion

### Synthesis and characterization

**Scheme 1:**
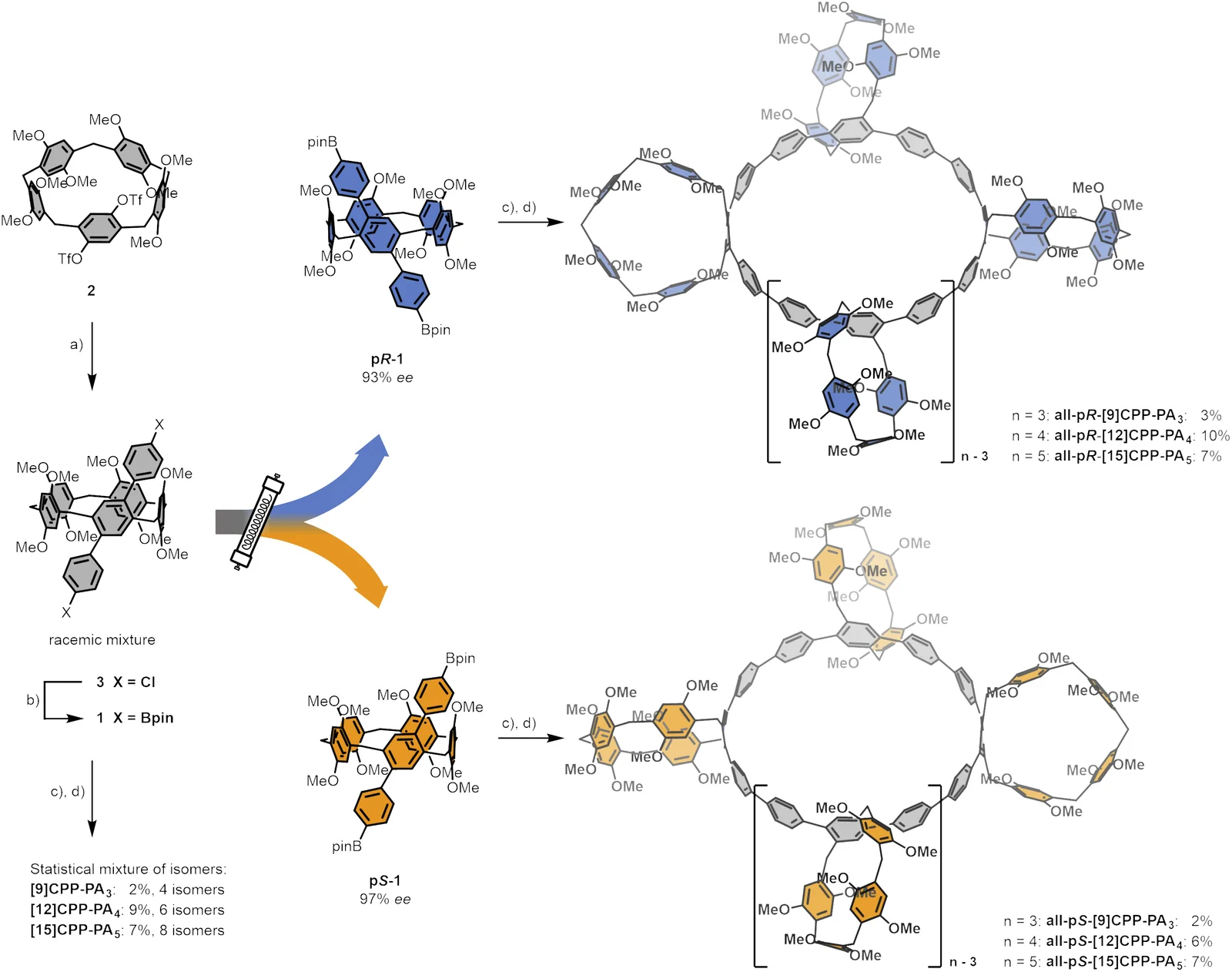
Stereoselective synthesis of multi-macrocycles all-p*R*- and all-p*S*-[*n*]CPP-PA_n/3_ (*n* = 3–5) from P[5]A-bistriflate 2*via* racemic P[5]A-bis(boronic ester) 1 (grey ringfill) and enantiomerically pure p*R*-1 and p*S*-1. PA rings with p*S* configuration are colored in orange, with p*R* configuration blue. Reaction conditions: (a) (4-chlorophenyl)boronic acid, Pd(PPh_3_)_4_, K_2_CO_3_, THF/H_2_O, 70 °C, overnight, 96%. (b) B_2_pin_2_, Pd_2_dba_3_, XPhos, KOAc, 1,4-dioxane, 105 °C, overnight, 81%. (c) Pt(cod)Cl_2_, CsF, THF, 55 °C, 3 d. (d) PPh_3_, *o*-dichlorobenzene, 200 °C, microwave, 5 min.

We synthesized the multi-macrocycles [*n*]CPP-PA_n/3_*via* the platinum-mediated macrocyclization,^33^ starting from P[5]A-bis(boronic ester) 1 (Scheme 1). 1 was synthesized from P[5]A-bistriflate 2 (ref. 34) by Suzuki–Miyaura coupling with (4-chlorophenyl)boronic acid and subsequent Miyaura–borylation in 78% yield over two steps. For the macrocyclization, first, racemic 1 was reacted with Pt(cod)Cl_2_ and cesium fluoride (Scheme 1, bottom left).^35,36^ The resulting crude mixture was subjected – without purification – to the reductive elimination step, performed by ligand exchange with triphenylphosphine and heating in a microwave reactor. Separation by preparative gel-permeation chromatography (GPC) furnished the tetrakis-, pentakis-, and hexakis-multi-macrocycles [9]CPP-PA_3_, [12]CPP-PA_4_, and [15]CPP-PA_5_ in 2%, 9% and 7% yield, respectively. This is surprising, as the Pt-mediated macrocyclization of terphenylenes usually exclusively gives the tetrameric [12]CPP.^37^ Using racemic 1 furnishes different combinations of p*S*- and p*R*-configurations in [*n*]CPP-PA_n/3_, specifically four, six and eight stereoisomers for [9]CPP-PA_3_, [12]CPP-PA_4_, and [15]CPP-PA_5_, respectively, most of which are chiral. ^1^H-NMR-spectroscopy revealed that their distribution matched the statistical prediction (see SI, Section 8). We were unable to separate these isomeric mixtures with GPC or HPLC.

The number of possible isomers can be reduced to one by employing an enantiopure starting material in the macrocyclization. Both 1 and the preceding P[5]A-bis(chlorophenyl) 3 have substituents large enough to prevent racemization. We separated the enantiomers of 3 and 1 on a several hundred milligram scale *via* chiral HPLC, furnishing the enantiomers in *ee*s of >99% and 98% for 3, and 97% and 93% for 1. A single crystal of the second eluted enantiomer of 3 could be grown by vapor diffusion of methanol into a CHCl_3_ solution and revealed the p*R* configuration (see SI). This enabled the structural assignment of p*R*-3 and p*S*-3, whose Miyaura borylation yielded the two respective enantiomers of the P[5]A-bis(boronic ester), p*R*-1 and p*S*-1. As had been the case for p*R*-3, p*R*-1 showed a longer retention time in the chiral stationary phase HPLC than p*S*-1. The conformation in all enantiomers of 3 and 1 was fully retained at room temperature over several months. We then subjected enantiopure p*R*-1 and p*S*-1, separated *via* HPLC, to Pt-mediated macrocyclizations. These yielded all-p*R*-[9]CPP-PA_3_, all-p*R*-[12]CPP-PA_4_, and all-p*R*-[15]CPP-PA_5_ from p*R*-1 in 3%, 10% and 7% yield, respectively, and all-p*S*-[9]CPP-PA_3_, all-p*S*-[12]CPP-PA_4_, and all-p*S*-[15]CPP-PA_5_ from p*S*-1 in 2%, 6% and 7%, respectively. These enantiomers, only containing p*R* or p*S* conformations, are the highest-symmetry isomers and statistically disfavored in the racemic reaction, with a statistical and observed share of 1/4, 1/8, and 1/16 (as racemate) for [9]CPP-PA_3_, [12]CPP-PA_4_, and [15]CPP-PA_5_, respectively (see SI, Section 8).

**Fig. 1:**
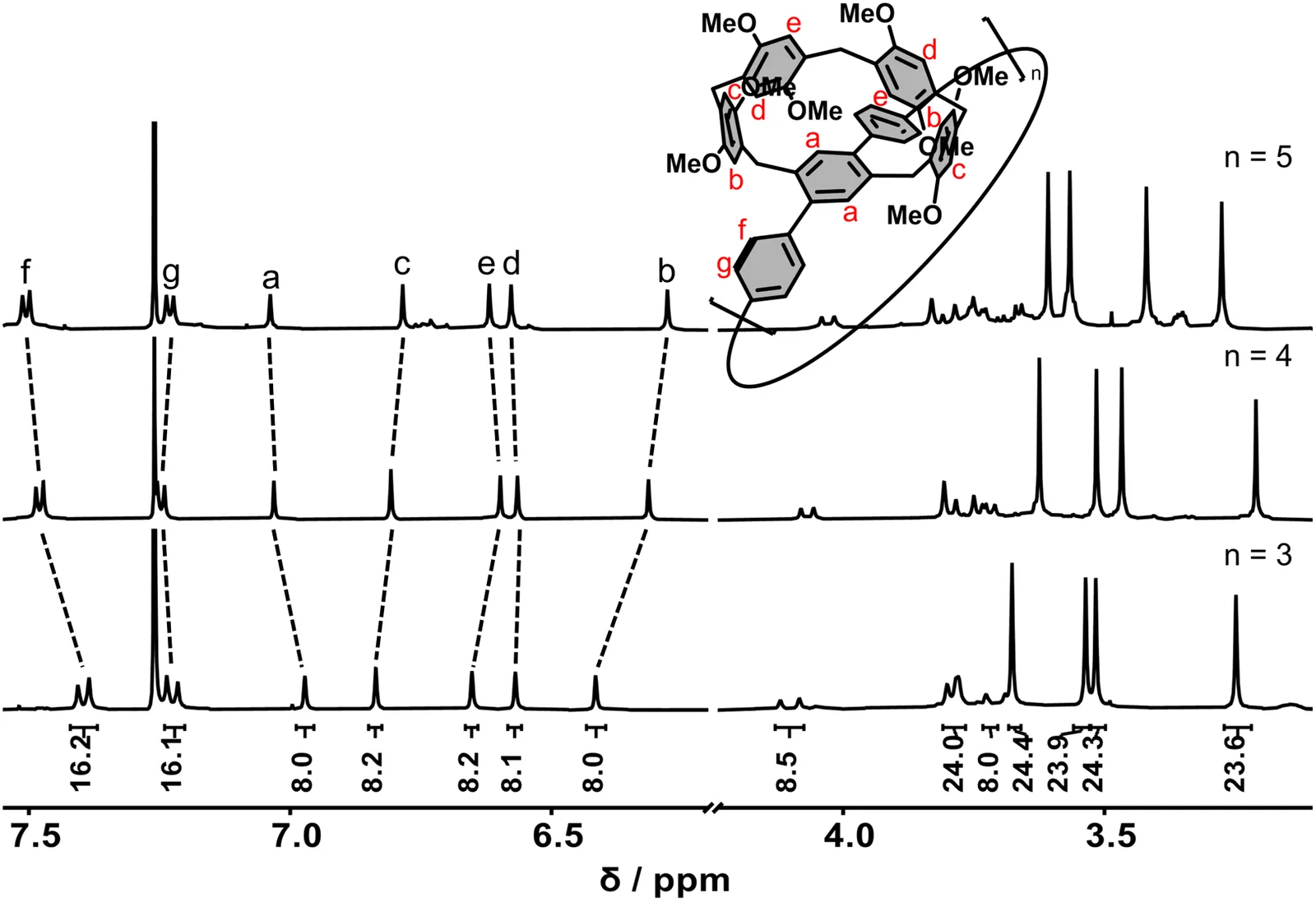
Partial ^1^H-NMR spectra (CDCl_3_, 298 K, 600 MHz) of racemic all-p*R*/all-p*S*-[9]CPP-PA_3_, all-p*R*/all-p*S*-[12]CPP-PA_4_, and all-p*R*/all-p*S*-[15]CPP-PA_5_. Assignment of the aromatic signals is based on 2D-NMR spectra.

The molecular structures of all compounds were confirmed by 1- and 2D-NMR spectroscopy and high-resolution mass spectrometry. The high symmetry of the all-p*R*- and all-p*S*-[*n*]CPP-PA_n/3_ (point groups: *D*_3_, *D*_4_, and *D*_5_) results in simple NMR spectra (Fig. 1). Since all repeating units of the [*n*]CPP-PA_n/3_ are chemically equivalent, their NMR spectra only differ in the chemical shifts of otherwise identical signals. The P[5]A subunits all lie on a *C*_2_ axis and therefore have five sets of two equivalent aromatic protons each, resulting in five singlets in the aromatic region (labelled a–e in Fig. 1). The remaining phenylene rings of the [*n*]CPP give two more signals in the aromatic region, each accounting for four protons of a repeating unit (labelled f and g). Molecular geometries were optimized with the self-consistent tight-binding quantum chemical method GFN2-xTB using implicit solvation by CH_2_Cl_2_ (see SI).^38,39^ While the P[5]A units largely adopt their signature pentagonal structure, their steric demand enforces large dihedral angles between the phenylene rings of the [*n*]CPPs. Median dihedral angles were calculated to be 42° for [9]CPP-PA_3_ (*vs.* 30° in [9]CPP), and 49° for both [12]CPP-PA_4_ and [15]CPP-PA_5_ (*vs.* 34° in [12]CPP and [15]CPP).^40^

### Photophysical and chiroptical properties

We studied the photophysical and chiroptical properties of the enantiopure all-p*R*- and/or all-p*S*-[*n*]CPP-PA_n/3_ multi-macrocycles by UV/Vis absorption and emission spectroscopy, fluorescence quantum yield and lifetime measurements, electronic circular dichroism (ECD) and CPL spectroscopy in CH_2_Cl_2_ (see Table 1 with comparison to the respective [*n*]CPPs).^19,41^ For absorption and emission data we used the all-p*R* enantiomers.

**Table 1:** Photophysical and chiroptical data of all-p*R*- and all-p*S*-[*n*]CPP-PA_n/3_ in comparison to the respective [*n*]CPPs^a^^19,41,42^

Compound	*λ*_max_ nm^−1^	*λ*_em_ nm^−1^	*ε*/10^5^ M^−1^ cm^−1^	Stokes shift eV^−1^	*Φ*_F_ (*τ*^b^ ns^−1^)	*g*_abs_ c	*g*_lum_ d	*B*_CPL_ e/M^−1^ cm^−1^
[9]CPP-P[5]A_3_	304	458	1.1	1.38	0.56 (2.2)	1.3 × 10^−3^	3.0 × 10^−3^	92
[9]CPP	340	494	1.2	1.14	0.38 (10.6)	—	—	—
[12]CPP-P[5]A_4_	303	425	1.6	1.18	0.81 (1.3)	1.2 × 10^−3^	4.2 × 10^−4^	27
[12]CPP	339	450	1.4	0.90	0.81 (2.2)	—	—	—
[15]CPP-P[5]A_5_	301	413	1.9	1.12	0.79 (0.78)	1.3 × 10^−3^	1.3 × 10^−3^	96
[15]CPP	339	416, 440	1.7	0.84	0.90 (—)	—	—	—

a In CH_2_Cl_2_.

b *τ* = 1/(*k*_r_ + ∑*k*_nr_).

c At the lowest-energy maximum. Mean value of both enantiomers.

d Mean value of a 50 nm range around the emission maximum and of both enantiomers.

e *B*_CPL_ = *ε* × *ϕ*_F_ × *g*_lum_/2.

**Fig. 2:**
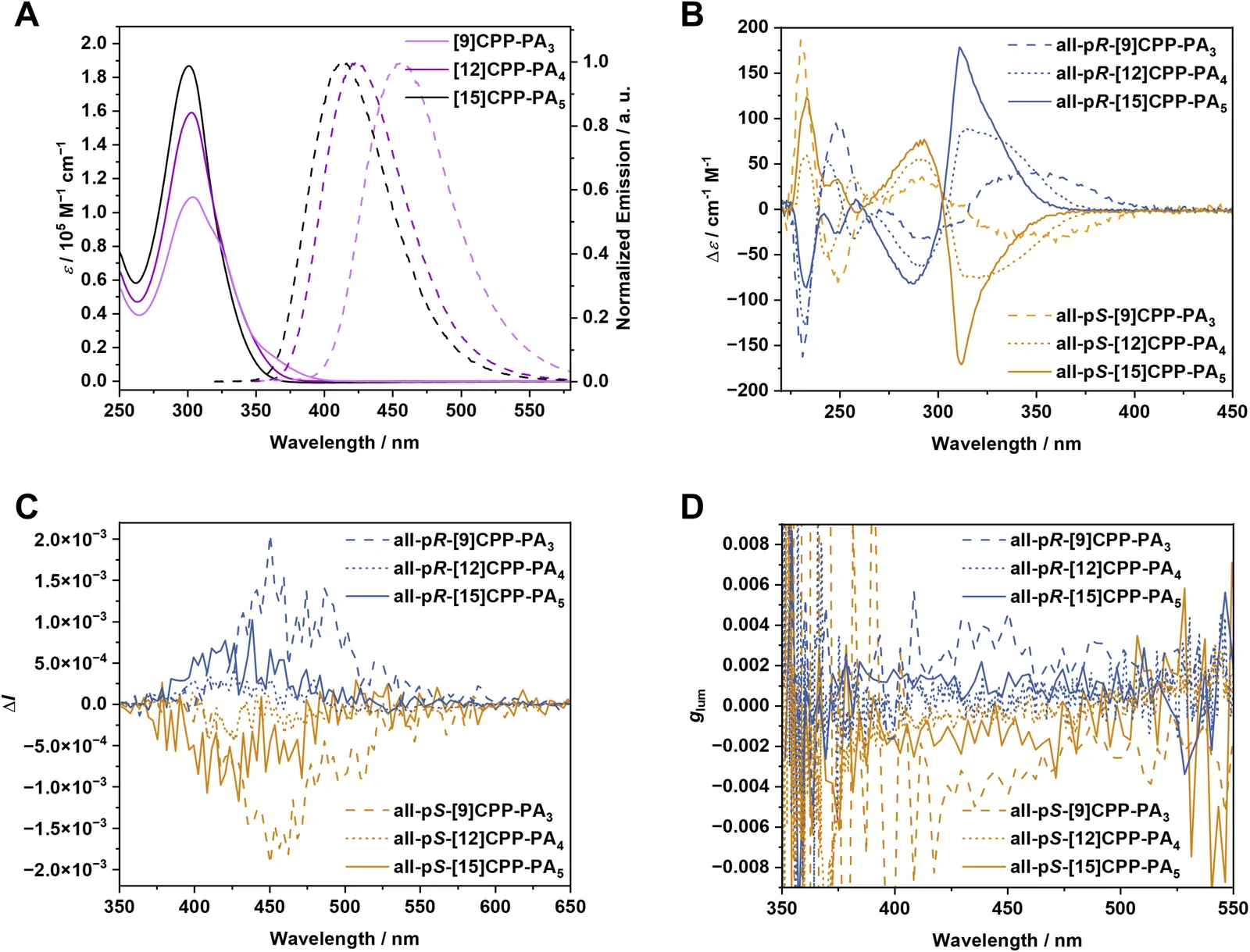
(A) UV/vis absorption (10^−5^ to 10^−6^m, solid lines) and emission (10^−7^ to 10^−8^m, dashed lines) spectra of all-p*R*-[9]CPP-PA_3_, all-p*R*-[12]CPP-PA_4_, and all-p*R*-[15]CPP-PA_5_. (B) ECD spectra of all-p*R*- and all-p*S*-[9]CPP-PA_3_, all-p*R*- and all-p*S*-[12]CPP-PA_4_, and all-p*R*- and all-p*S*-[15]CPP-PA_5_; 10^−5^ to 10^−6^m. (C) CPL spectra of all-p*R*- and all-p*S*-[9]CPP-PA_3_ (PEM-based setup), all-p*R*- and all-p*S*-[12]CPP-PA_4_ (QWP-based setup), and all-p*R*- and all-p*S*-[15]CPP-PA_5_ (PEM-based setup); 10^−5^ to 10^−6^m, *λ*_exc_ = 335, 343 nm. (D) *g*_lum_ plots of all-p*R*- and all-p*S*-[9]CPP-PA_3_ (PEM-based setup), all-p*R*- and all-p*S*-[12]CPP-PA_4_ (QWP-based setup), and all-p*R*- and all-p*S*-[15]CPP-PA_5_ (PEM-based setup); 10^−5^ to 10^−6^m, *λ*_exc_ = 335, 343 nm. All in CH_2_Cl_2_ at 298 K; PEM = photoelastic modulator; QWP = quarter-waveplate.

The three hoop sizes have a similar absorption spectrum with a maximum at *ca.* 300 nm (Fig. 2A). [9]CPP-PA_3_ additionally exhibits a shoulder at 325 nm; shoulder-like absorption bands have also been reported for [*n*]CPPs, in particular [9]CPP.^41^ The molar extinction coefficients lie above 1 × 10^5^ m^−1^ cm^−1^ and are increasing with hoop size, which is consistent with [9]-, [12]-, and [15]CPP, but the absorption maxima of the [*n*]CPP-PA_n/3_ are blue-shifted by around 0.43 eV.^19,41^ This hypsochromic shift could be explained by the steric demand of the P[5]A rings enforcing larger dihedral angles upon the [*n*]CPP and reducing conjugation. In [*n*]CPPs, the common absorption maximum can be attributed to transitions from HOMO−1/HOMO−2 to the LUMO and from HOMO to LUMO+1/LUMO+2, which are insensitive to hoop size.^41^ The HOMO–LUMO transition is Laporte forbidden due to their centrosymmetry, and the increasing band gap only manifests itself as a blue-shift of the absorption onset with increasing size.^37,41^ Simplified time-dependent density functional theory (sTDDFT) calculations were performed to investigate the nature of the electronic transitions (see SI, Section 12.2 and 12.3).^43^ The first transition energy is size-dependent, but has a low oscillator strength (0.04–0.19) in all [*n*]CPP-PA_n/3_. The second and third transitions differ less in energy between the three sizes and have high oscillator strengths (1.39–2.36). The energies match experimental observations reasonably well (see SI for simulated absorption spectra). The emission bands have maxima at 458 nm, 425 nm, and 413 nm for [9]CPP-PA_3_, [12]CPP-PA_4_, and [15]CPP-PA_5_, respectively (Fig. 2A). This blue-shift with increasing hoop size is in line with the series of [*n*]CPPs and can be explained by the higher hoop strain, smaller torsional angles and better conjugation between phenylene rings in smaller hoops.^40,44^ As with absorption, the emission maxima are significantly blue-shifted in the [*n*]CPP-PA_n/3_ compared to [*n*]CPPs, likely due to the generally larger dihedral angles.^19,41^ The Stokes shifts are larger than in the parent [*n*]CPPs. The fluorescence quantum yields *Φ*_F_ are 56%, 81%, and 79% for [9]CPP-PA_3_, [12]CPP-PA_4_, and [15]CPP-PA_5_, respectively, and are comparable to those of the respective [*n*]CPPs. Fluorescence lifetime measurements revealed short excited-state lifetimes of 2.2 ns, 1.3 ns, and 0.78 ns for [9]CPP-PA_3_, [12]CPP-PA_4_ and [15]CPP-PA_5_, respectively (see SI, Section 9). This follows a decreasing trend with increasing hoop size, while these lifetimes are slightly lower than those of the corresponding [*n*]CPPs (no data for [15]CPP).^41^

The ECD spectra of each pair of enantiomers of the [*n*]CPP-PA_n/3_ display a mirror–image relationship with pronounced cotton effects (Fig. 2B and S43–S45). The experimental spectra match the calculated ones reasonably well and confirm the absolute configuration obtained from the single crystal of 3 (see SI, Section 12.4). The first cotton effect, attributable to the first excitation, is more pronounced than that at the absorption maximum around 300 nm. This suggests a higher asymmetry in the first excitation than in the second. The molar extinction values of the first cotton effect increase with hoop size, following the trend observed in UV/vis spectroscopy, leading to similar *g*_abs_ (Δ*ε*/*ε*) values between 1.2 × 10^−3^ and 1.3 × 10^−3^ in all hoop sizes.

All CPL spectra were measured on a photoelastic modulator (PEM)-based CPL spectrometer.^45^all-p*R* and all-p*S*-[12]CPP-PA_4_ were additionally measured on a quarter-waveplate (QWP)-based setup^46^ to validate the measured values, yielding very similar results (Fig. 2C and S49–S52). The CPL spectra of all compounds show the same Δ*I* maxima as the emission spectra. The *g*_lum_ values were calculated as the mean of a 50 nm range around the emission maximum. [9]CPP-PA_3_ and [15]CPP-PA_5_ show similar *g*_lum_ values with 3.0 × 10^−3^ and 1.3 × 10^−3^, respectively, while [12]CPP-PA_4_ has a lower *g*_lum_ of 4.2 × 10^−4^ (Fig. 2D). The *g*_lum_ values do not follow a size-dependent trend. It is noteworthy that the lower value is observed in the hoop size with the highest symmetry [*n*]CPP core, as only [12]CPP-PA_4_ has an even number of phenylene rings. The CPL brightness *B*_CPL_ was calculated from the *g*_lum_ values, molar extinction coefficients and *Φ*_F_ values. With 92 M^−1^ cm^−1^ ([9]CPP-PA_3_), 27 M^−1^ cm^−1^ (12]CPP-PA_4_) and 96 M^−1^ cm^−1^ ([15]CPP-PA_5_), the *B*_CPL_ values are comparable to those of other reported chiral nanohoops,^21,47,48^ with [15]CPP-PA_5_ giving the highest value.

## Conclusions

We herein demonstrated the stereoselective synthesis of the uniform [*n*]CPP-P[5]A multi-macrocycles [*n*]CPP-PA_n/3_ (*n* = 9, 12, 15) and studied their photophysical and chiroptical properties. By employing an enantiopure starting material in the platinum-mediated macrocyclization, only the highest-symmetry all-p*R* or all-p*S* isomers of the three different hoop sizes were obtained as otherwise statistically disfavored isomers. Between all sizes, a shared absorption band and blue-shifting emission with larger size was observed, following the reported behavior of the parent [*n*]CPPs. The bulky P[5]A groups enforce large dihedral angles on the [*n*]CPP, causing a blue-shift in absorption and emission. The enantiopure materials displayed characteristic ECD spectra and CPL with *g*_lum_ values between 4.2 × 10^−4^ and 3.0 × 10^−3^. The CPL brightness *B*_CPL_ reached up to 96 M^−1^ cm^−1^, a comparably high value based on an enantioselective synthesis.

## Author contributions

Conceptualization: Y. S. H. and B. E.; data curation: all authors; formal analysis: Y. S. H., M. S., and M. M.; funding acquisition: B. E., M. M. and S. F.; investigation: Y. S. H., M. S., and M. M.; methodology: Y. S. H., M. S., and M. M.; project administration: B. E.; supervision: B. E., S. F.; validation: all authors; visualization: Y. S. H.; writing – original draft: Y. S. H. and B. E.; writing – review and editing: all authors.

## Conflicts of interest

There are no conflicts to declare.

## Supplementary Material

SC-OLF-D6SC03987A-s001

SC-OLF-D6SC03987A-s002

## Data Availability

The data supporting this article are freely available on Zenodo under https://doi.org/10.5281/zenodo.20039336. Supplementary information (SI): materials and methods, synthetic procedures, NMR and mass spectra, HPLC chromatograms, fluorescence lifetime measurements, CD and CPL spectra, and computational results. See DOI: https://doi.org/10.1039/d6sc03987a. CCDC 2546080 contains the supplementary crystallographic data for this paper.^49,50^
