# Supplementary material for: Enantiopure synthesis of uniform [n]cycloparaphenylene-pillar[5]arene multi-macrocycles and their chiroptical properties
Source: Chem Sci. 2026 Jul 28;17(38):18161–6. doi: 10.1039/d6sc03987a (PMC13460006; doi:10.1039/d6sc03987a)
Supplement: Supplementary file 1 [file SC-017-D6SC03987A-s001.pdf]

Supplementary Information (SI) for Chemical Science.
This journal is © The Royal Society of Chemistry 2026

Supporting Information

Enantiopure Synthesis of Uniform [*n*]Cycloparaphenylene Pillar[5]arene Multi Macrocycles and their Chiroptical Properties

Table of Contents

1. Materials and Methods S3
2. Synthetic Procedures S6
3. Assignment of Stereodescriptors S12
4. NMR Spectra S13
5. High-Resolution Mass Spectra S26
6. Single-crystal X-ray Diffraction of p*R*-3 .... S27
7. Chiral Stationary Phase Chromatograms S29
8. Analysis of the Statistical Isomer Mixture Obtained from the Cyclooligomerization of Racemic S1 .... S31
9. Fluorescence Lifetime Measurements S37
10. Electronic Circular Dichroism Spectra S39
11. Circularly Polarized Luminescence Spectra S42
12. Computational Results S46
13. References S80

1. Materials and Methods

Chemicals and solvents were purchased from ABCR, ACROS ORGANICS, ALFA AESAR, BLDPHARM, CARBOLUTION, FISHER SCIENTIFIC, FLUOROCHEM, GRÜSSING, SIGMA-ALDRICH, THERMO SCIENTIFIC or VWR CHEMICALS and used directly without further purification unless otherwise specified. Moisture- or oxygen-sensitive reactions were carried out in dried glassware, using standard Schlenk techniques in a dry argon atmosphere (Argon 4.6 from MTI INDUSTRIEGASE). Solvents were degassed using the freeze-pump-thaw method or sparging with argon, hereafter denoted as argon-purged. Anhydrous (anh.) THF and toluene were obtained from an M. BRAUN solvent purification system (MB-SPS-800) and stored over molecular sieves (3 Å). Other anhydrous solvents were obtained by drying over activated molecular sieves (3 Å) for several days. Other solvents were purchased and used in technical or laboratory grade.

Analytical thin layer chromatography (TLC) was carried out using silica gel-coated aluminum plates with a fluorescence indicator (MACHEREY-NAGEL Alugram® *Xtra SIL G UV254*). Detection was carried out by using UV light (λ_{max} = 254 nm and 366 nm).

Flash column chromatography was carried out using silica gel 60, grain size 40–63 µm (230– 400 mesh) from MACHEREY-NAGEL.

Semipreparative HPLC was used to separate the enantiomers on a chiral stationary phase using a KNAUER Azura P2.1L Series with a semi-preparative CHIRALPAK® IK column (250 x 21 mm, 5 µm) at a flow rate of 20 ml min^{-1} and an injection amount of 10-20 mg racemate in 2 ml eluent.

Preparative size-exclusion chromatography (SEC) was performed on a JAI LaboACE LC7080 Plus recycling preparative HPLC with a set of two JAI *JAIGEL-2HR Plus* columns. HPLC-grade CH_2Cl_2 (amylene stabilized) was used as eluent.

Nuclear magnetic resonance (NMR) spectra were recorded at 298 K on the following spectrometers: BRUKER Avance Neo 400 (400.1 MHz (^{1}H), 101.6 MHz (^{13}C), 161.9 MHz (31P)) or BRUKER Avance Neo 600 (600.2 MHz (^{1}H), 150.9 MHz (^{13}C)). Chemical shifts are reported in parts per million (ppm, δ scale). ^{1}H NMR spectra are referenced to the residual proton signal of the respective solvent: $CDCl_3$: 7.26 ppm. ^{13}C NMR spectra are referenced to the following signals: $CDCl_3$: 77.16 ppm.[1] Analysis followed first order, and the following abbreviations for multiplets were used: singlet (s), doublet (d), multiplet (m) and combinations thereof, *i. e.*, doublet of doublets (dd). Coupling constants (*J*) are given in Hertz (Hz). For analysis, MESTRENOVA V14.2.0 was used.

High-resolution mass spectra (HRMS) were measured on a BRUKER solarX Hybrid 7T FTICR *via* matrix-assisted laser desorption/ionization (MALDI) using *trans*-2-[3-(4-*tert*-butylphenyl)-2-methyl-2-propenylidene]malononitrile (DCTB) as matrix.

UV/Vis absorption spectra were measured on a SHIMADZU UV-3600i Plus UV-VIS-NIR spectrophotometer using Quartz (SUPRASIL) cuvettes (1 cm path length) from HELLMA ANALYTICS. Extinction coefficients were measured by serial dillution.

Fluorescence spectra were measured on a PERKINELMER LS 55 luminescence spectrometer using Quartz (SUPRASIL) cuvettes (1 cm path length) from HELLMA ANALYTICS. Analyte concentrations were below 10^{-6} M.

Fluorescence quantum yields Φ_F were determined using a HAMAMATSU QuantaurusQY (C11347) (HAMAMATSU PHOTONICS, Herrsching am Ammersee, Germany) for CH_2Cl_2 solutions. Analyte concentrations were below 10^{-6} M.

Photoluminescence lifetime measurements were recorded with a DeltaPro from HORIBA SCIENTIFIC using a 372 nm pulsed Laser source (Class 3B Laser Product, <0.5 W peak in pulsed and CW mode). The Delta Pro consists of: DeltaDiode (Picosecond diode controller), DeltaHub (High throughput TCSPC controller), DPS-1 (Detector Power supply) and a PPD (Picosecond photon detection module). The IRF (Instrument response function) was measured with LUDOX silica nanoparticles. Fits were done with the software DECAYFIT – Emission Decay Analysis Software (Version 1.4).

Circular Dichroism (CD) spectra were recorded on a JASCO J-1500 spectrometer using Quartz (SUPRASIL) cuvettes (1 cm path length). Detection was performed using the standard photomultiplier tube (PMT) detector. Spectra were acquired over the range 200–600 nm with a step size of 1 nm and an integration time of 1 s per step.

Circularly Polarized Luminescence (CPL) measurements were performed using two different setups: A photoelastic modulator (PEM)-based CPL spectrometer[2] and a broadband quarter-waveplate (QWP)-based CPL setup.[3]

For the PEM-based measurements, excitation was provided by a broadband white light source (ENERGETIQ EQ-99X LDLS) in combination with a 343 nm bandpass filter (Thorlabs FBH343-10), delivering 0.45 mW at a beam diameter of 1862.25 µm at the sample. Details of the PEM-based setup and its methodology have been described previously.[2]

For the QWP-based measurements, a pulsed amplified laser system (PHAROS, Light Conversion; Yb:KGW lasing medium) coupled to an optical parametric amplifier (ORPHEUS, Light Conversion) was used as the excitation source creating ca. 300 fs long pulses at 335 nm with a repetition rate of 50 kHz.

Excitation powers were 0.43 mW and 0.40 mW for samples **all-p*S*-[12]CPP-PA$_4$** and **all-p*R*-[12]CPP-PA$_4$**, respectively, corresponding to laser fluences of approximately 5.25 µJ/cm² and 4.88 µJ/cm² at a beam diameter of 456.8 µm. Further details of the QWP-based setup have been reported previously.[3]

Computational Details

All computations were done on the Justus High Performance Computing Cluster (bwForCluster JUSTUS 2 as part of the bwHPC facility).

Geometry optimizations were done with the self-consistent tight-binding quantum chemical method GFN2-xTB[4,5] using the ALPB implicit solvation model[6] with CH_2Cl_2. Vertical excitations were calculated with the ORCA quantum chemistry software package[7] (Version 6.0.1) using simplified time-dependent density functional theory[8] on the ωB97X/def2-SV(P)[9,10] level of theory. Absorption and CD spectra were simulated with a Gaussian band shape using SpecDis[11]. FWHM values were $\sigma = 0.16$ and 0.25 for absorption and CD spectra, respectively.

2. Synthetic Procedures

Synthesis of Pillar[5]arene (S1)[12]

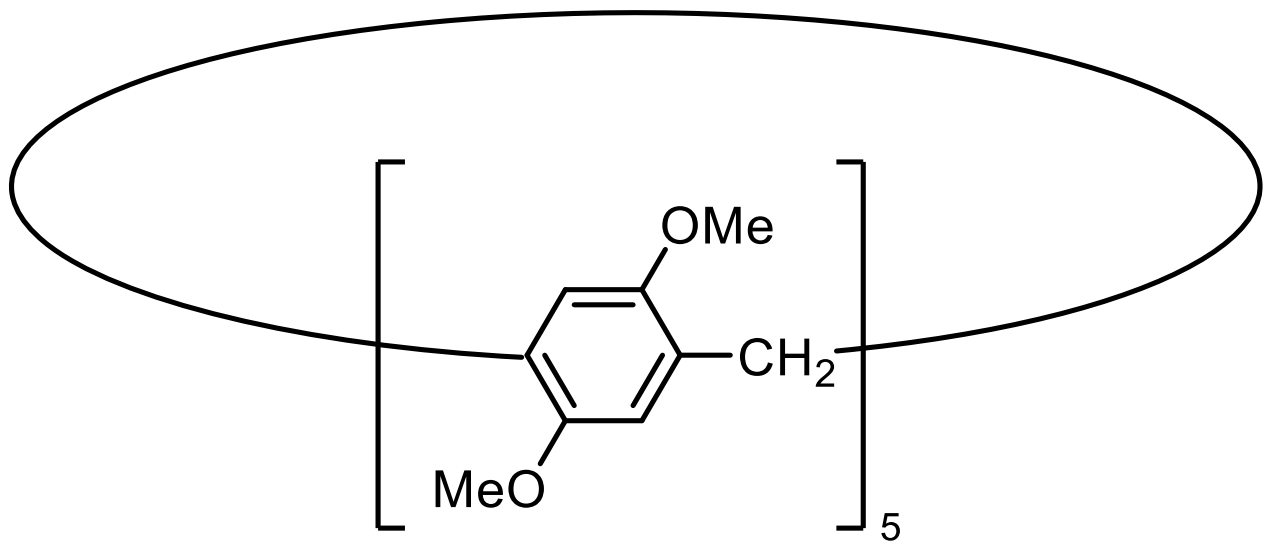

In a round-bottom flask 1,4-dimethoxybenzene (5.60 g, 40.5 mmol), paraformaldehyde (1.22 g, 40.5 mmol, 1.00 equiv.) and trifluoroacetic acid (15 ml) were dissolved in 1,2-dichloroethane (200 ml) and refluxed at 90 °C for 4 h. After cooling to room temperature, the mixture was poured into methanol (300 ml). The resulting solid was collected *via* filtration and washed with methanol to obtain pillar[5]arene (12,15,32,35,52,55,72,75,92,95-decamethoxy-1,3,5,7,9(1,4)-pentabenzenacyclodeca-phane, **S1**, 5.39 g, 7.18 mmol, 88 %) as a light-green solid.

^{1}H NMR (400 MHz, $CDCl_3$): δ [ppm] = 6.87 (s, 10H), 3.76 (s, 10H), 3.73 (s, 30H). The analytical data were in accordance to literature.[12]

Synthesis of (Z)-12,15,32,35,52,55,72,75-octamethoxy-1,3,5,7(1,4)-tetrabenzena-9(1,4)-cyclohexanacyclodecaphane-91(96),93-diene-92,95-dione (S2)[13]

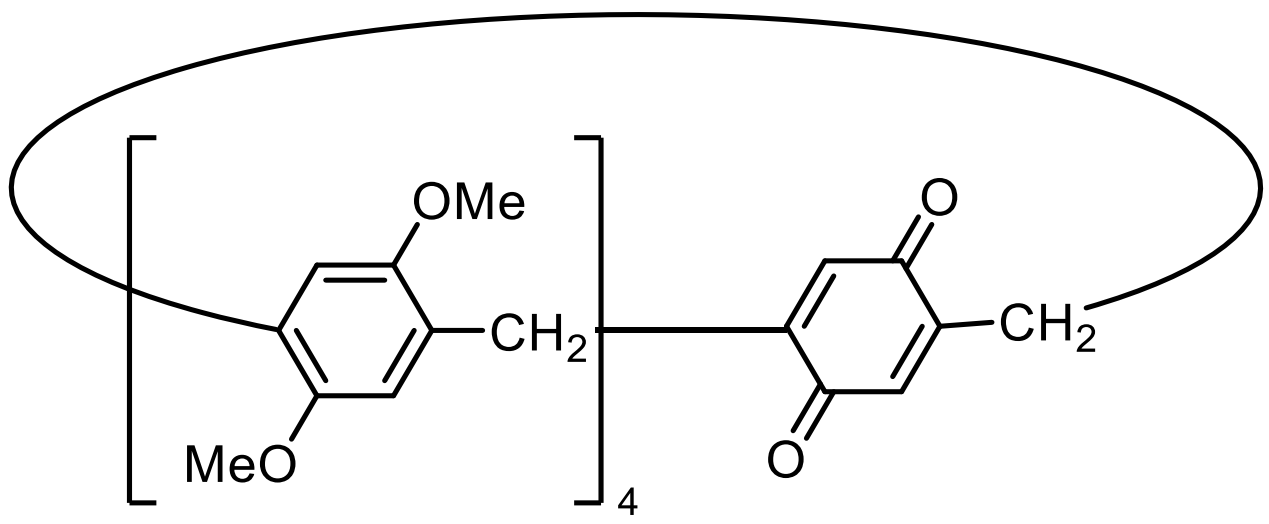

In a round-bottom flask P[5]A (**S1**, 1.00 g, 1.33 mmol) was dissolved in DCM (70 ml), and an aqueous solution of diammonium cerium(IV) nitrate (1.61 g, 2.93 mmol, 2.20 equiv. in 15 ml H_2O) was added dropwise. The resulting mixture was stirred at room temperature for 2 h. The phases were separated and the organic phase was washed with water (2 × 50 ml) and brine (50 ml) and dried over sodium sulfate. The solvent was removed under reduced pressure. The crude product was purified by flash column chromatography (CH_2Cl_2:ethyl acetate 99:1) to obtain a mixture of (Z)-12,15,32,35,52,55,72,75-octamethoxy-1,3,5,7(1,4)-tetrabenzena-9(1,4)-cyclohexanacyclodecaphane-91(96),93-diene-92,95-dione (**S2**) and unreacted P[5]A (620 mg, ~9:1 according to ^{1}H-NMR, ~57%) as a dark red solid.

Synthesis of 32,35,52,55,72,75,92,95-octamethoxy-1,3,5,7,9(1,4)-pentabenzenacyclodecaphane-12,15-diyl bis(trifluoromethanesulfonate) (2)[14]

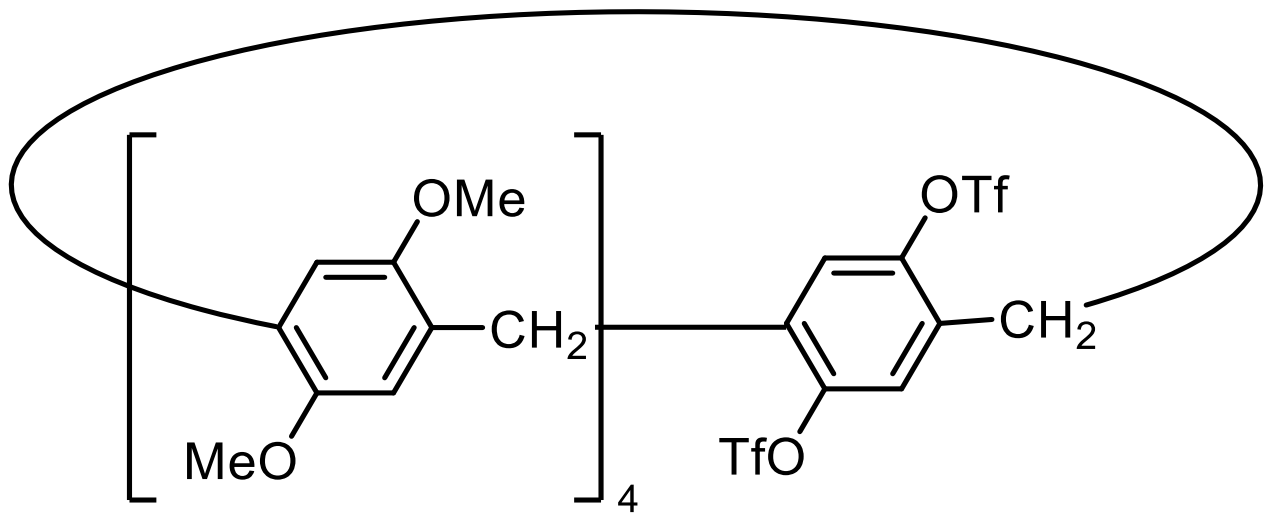

In a round-bottom flask (Z)-12,15,32,35,52,55,72,75-octamethoxy-1,3,5,7(1,4)-tetrabenzena-9(1,4)-cyclohexanacyclodecaphane-91(96),93-diene-92,95-dione (**S2**, 2.94 g, 80% purity in a mixture with P[5]A, 3.26 mmol) was dissolved in a mixture of CH_2Cl_2 (60 ml) and MeOH (30 ml), and $NaBH_4$ (580 mg, 15.3 mmol, 5.00 equiv. was added portion-wise. The mixture was stirred at room temperature for 15 min, and 1 M HCl was added until the mixture was neutral. Water (50 ml) was added, and the aqueous phase was extracted with CH_2Cl_2 (3 × 30 ml). The organic phase was washed with brine (30 ml), dried over sodium sulfate, and the solvent was removed under reduced pressure. The resulting colorless solid was used for the subsequent step without further purification.

In a Schlenk flask the solid from the last step was dissolved in anhydrous CH_2Cl_2 and cooled to 0 °C. Anhydrous pyridine (836 µl, 10.4 mmol, 3.50 equiv.) was added, and the mixture was stirred for 15 min. Trifluoromethanesulfonic anhydride (1.25 ml, 7.42 mmol, 2.50 equiv.) was added dropwise, and the resulting mixture was stirred at 0 °C for 30 min and at room temperature for 2 h. The organic phase was washed with water (100 ml), 1 M HCl (50 ml) and brine (50 ml), dried over sodium sulfate, and the solvent was removed under reduced pressure. The crude product was purified by flash column chromatography (cyclohexane:CH_2Cl_2 1:3) to obtain 32,35,52,55,72,75,92,95-octamethoxy-1,3,5,7,9(1,4)-pentabenzenacyclodecaphane-12,15-diyl bis(trifluoromethanesulfonate) (**2**, 1.63 g, 1.65 mmol, 50%) as a white solid.

^{1}H NMR (400 MHz, $CDCl_3$): δ [ppm] = 7.33 (s, 2H), 6.80 (s, 2H), 6.78 (s, 2H), 6.76 (s, 2H), 6.69 (s, 2H), 3.85 (s, 4H), 3.79 (s, 6H), 3.72 (s, 6H), 3.68 (s, 6H), 3.66 (s, 6H), 3.61 (s, 6H).

The analytical data were in accordance to literature.[15]

Synthesis of 12,15-bis(4-chlorophenyl)-32,35,52,55,72,75,92,95-octamethoxy-1,3,5,7,9(1,4)-pentabenzenacyclodecaphane (3)

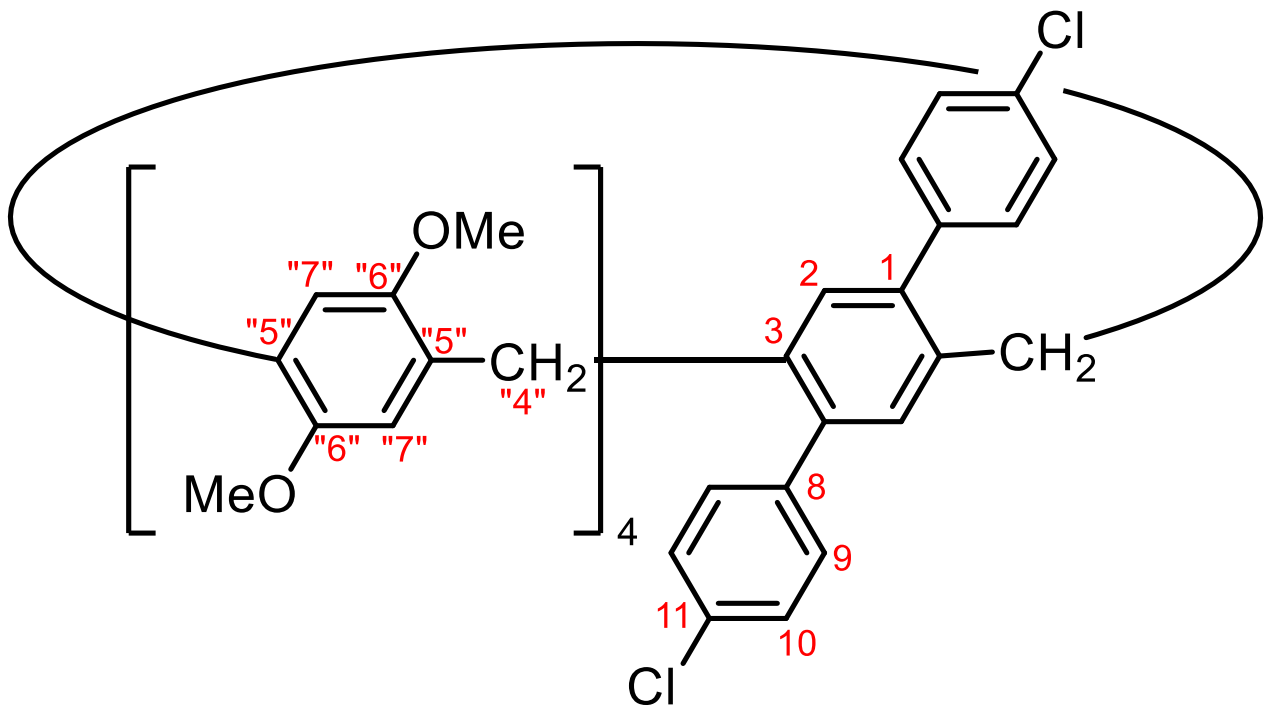

In a Schlenk tube 32,35,52,55,72,75,92,95-octamethoxy-1,3,5,7,9(1,4)-pentabenzenacyclodecaphane-12,15-diyl bis(trifluoromethanesulfonate) (**2**, 1.24 g, 1.26 mmol), (4-chlorophenyl)boronic acid (2.20 equiv., 432 mg, 2.76 mmol) and tetrakis(triphenylphosphane)palladium(0) (0.20 equiv., 290 mg, 251 µmol) were evacuated and back-filled with argon three times. Degassed THF (12 ml) and a degassed 1 M aqueous K_2CO_3 solution (4 ml) were added, and the resulting mixture was stirred at 70 °C overnight. Water (100 ml) was added, and the aqueous phase was extracted with 3 × 30 ml CH_2Cl_2. The organic phase was washed with water (2 × 50 ml) and brine (50 ml) and dried over sodium sulfate. The solvent was removed under reduced pressure. The crude product was purified by flash-column chromatography (PE:CH_2Cl_2 1:1 → 1:3) to give 12,15-bis(4-chlorophenyl)-32,35,52,55,72,75,92,95-octamethoxy-1,3,5,7,9(1,4)-pentabenzenacyclodecaphane (**3**, 1.24 g, 1.21 mmol, 96 %) as a white solid.

***R*f** (PE:CH_2Cl_2 1:3) = 0.53.

^{1}H NMR[1] (600 MHz, $CDCl_3$): δ [ppm] = 7.09 (d, J = 8.4 Hz, 4H, H-10), 6.81 (d, J = 8.0 Hz, 4H, H-9), 6.79 (s, 2H, H-2), 6.74 (s, 2H, H-7), 6.73 (s, 2H, H-7), 6.53 (s, 2H, H-7), 5.94 (s, 2H, H-7), 3.90 (s, 2H, H-4), 3.86–3.70 (m, 14H, H-4, *O*CH3), 3.57 (s, 6H, *O*CH3), 3.44 (s, 6H, *O*CH3), 3.29 (s, 6H, *O*CH3).

^{13}C NMR[1] (151 MHz, $CDCl_3$): δ [ppm] = 151.1 (C-6), 151.1 (C-6), 151.0 (C-6), 150.8 (C-6), 140.6 (C-8), 139.6 (C-1), 136.9 (C-3), 132.5 (C-11), 131.8 (C-2), 130.5 (C-9), 129.1 (C-5), 128.7 (C-5), 128.7 (C-5), 128.2 (C-10), 127.8 (C-5), 114.3 (C-7), 114.1 (C-7), 114.1 (C-7), 56.2 (*OCH3*), 56.1 (*OCH3*), 55.8 (*OCH3*), 55.7 (*OCH3*), 34.1 (C-4), 30.1 (C-4), 29.5 (C-4).

HRMS (MALDI$^+$): m/z calcd. for $C_{55}H_{52}Cl_2O_8$ [$M^{\bullet+}$]: 910.3039, found: 910.3036.

---

[1] Numbers 4,5,6, and 7 each mark multiple chemically inequivalent atoms in the repeating pillarene motif that could not be unambiguously assigned.

Synthesis of 2,2'-((32,35,52,55,72,75,92,95-octamethoxy-1,3,5,7,9(1,4)-pentabenzenacyclodecaphane-12,15-diyl)bis(4,1-phenylene))bis(4,4,5,5-tetramethyl-1,3,2-dioxaborolane) (1)

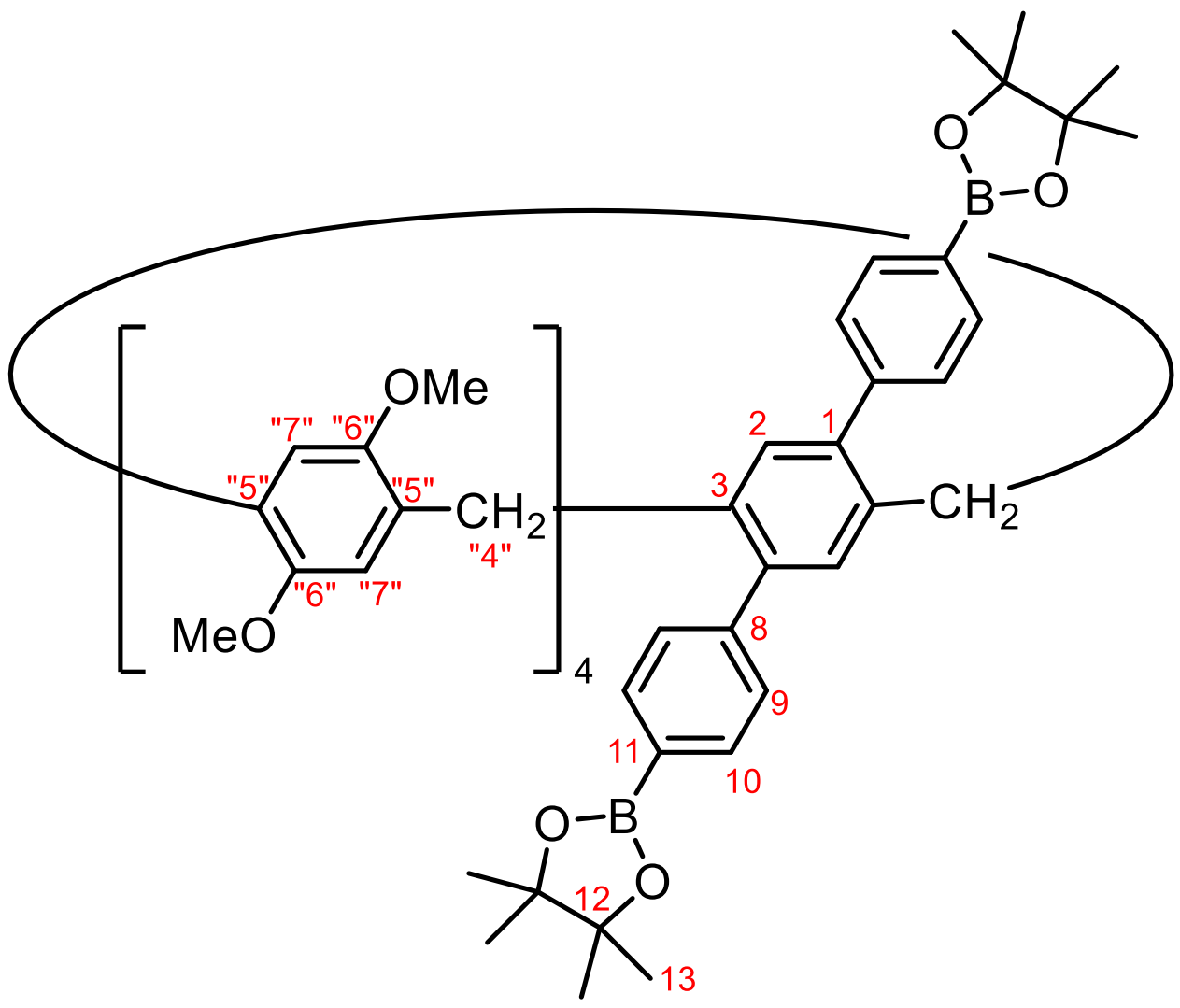

In a Schlenk tube 12,15-bis(4-chlorophenyl)-32,35,52,55,72,75,92,95-octamethoxy-1,3,5,7,9(1,4)-pentabenzenacyclodecaphane (**3**, 200 mg, 219 µmol), bis(pinacolato)diboron (2.40 equiv., 134 mg, 526 µmol), tris(dibenzylideneacetone)dipalladium(0) (0.02 equiv., 4.02 mg, 4.39 µmol), XPhos (0.08 equiv., 8.36 mg, 17.6 µmol) and potassium acetate (3.00 equiv., 64.6 mg, 658 µmol) were evacuated and back-filled with argon three times. Degassed 1,4-dioxane (4 ml) was added, and the resulting mixture was stirred at 105 °C overnight. After cooling, the mixture was filtered through a short plug of silica, rinsed with ethyl acetate, and the solvent was removed under reduced pressure. The residue was suspended in methanol (5 ml), sonicated for 10 min, and the solid was filtered off to give 2,2'-((32,35,52,55,72,75,92,95-octamethoxy-1,3,5,7,9(1,4)-pentabenzenacyclodecaphane-12,15-diyl)bis(4,1-phenylene))bis(4,4,5,5-tetramethyl-1,3,2-dioxaborolane) (**1**, 196 mg, 179 µmol, 81 %) as a white solid.

^{1}H NMR[1] (600 MHz, $CDCl_3$): *δ* [ppm] = 7.76 (d, *J* = 7.7 Hz, 4H, H-10), 7.12–7.10 (m, 6H, H-2, H-9), 6.80 (s, 2H), 6.71 (s, 2H, H-7), 6.50 (s, 2H, H-7), 5.80 (s, 2H, H-7), 3.93–3.77 (m, 6H, H-4), 3.73 (s, 4H, H-4), 3.70 (s, 6H, OCH_3), 3.61 (s, 6H, OCH_3), 3.37 (s, 6H, OCH_3), 3.32 (s, 6H, OCH_3), 1.38 (s, 24H, H-13).

^{13}C NMR[1] (151 MHz, $CDCl_3$): *δ* [ppm] = 151.2 (C-6), 151.2 (C-6), 150.9 (C-6), 150.6 (C-6), 145.5 (C-8), 140.5 (C-1), 136.4 (C-3), 134.6 (C-10), 132.4 (C-2), 129.1 (C-9), 128.7 (C-5), 128.7 (C-5), 128.6 (C-5), 128.0 (C-5), 114.7 (C-7), 114.1 (C-7), 113.9 (C-7), 113.9 (C-7), 83.9 (C-12), 56.3 (OCH_3), 56.2 (OCH_3), 55.7 (OCH_3), 55.5 (OCH_3), 33.2 (C-4), 30.0 (C-4), 29.9 (C-4), 25.1 (C-13), 25.0 (C-13).

HRMS (MALDI$^+$): m/z calcd. for $C_{67}H_{76}B_2O_{12}$ [$M^{•+}$]: 1094.5532, found: 1094.5528.

Synthesis of [*n*]CPP-PA$_{n/3}$

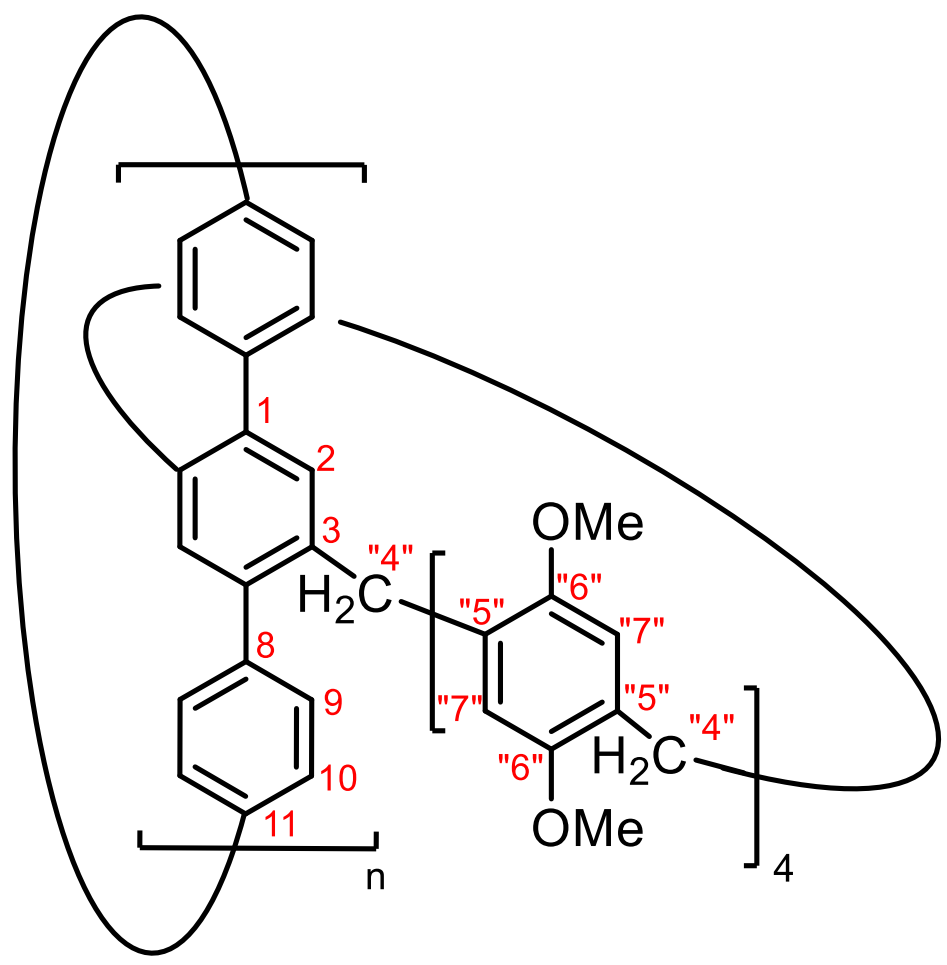

In a Schlenk flask 2,2'-((32,35,52,55,72,75,92,95-octamethoxy-1,3,5,7,9(1,4)-pentabenzenacyclodecaphane-12,15-diyl)bis(4,1-phenylene))bis(4,4,5,5-tetramethyl-1,3,2-dioxaborolane) (**1**, 145 mg, 132 µmol), dichloro(1,5-cyclooctadiene)platinum(II) (1.00 equiv., 49.6 mg, 132 µmol) and cesium fluoride (6.00 equiv., 121 mg, 795 µmol) were dissolved in anhydrous THF (130 ml), and the resulting mixture was stirred at 55 °C for 3 days. The solvent was removed under reduced pressure. The crude mixture was transferred to a microwave vial and flushed with argon. Triphenylphosphine (40 equiv., 1.39 g, 5.30 mmol) and anhydrous *o*-dichlorobenzene (20 ml) were added, and the mixture was stirred in a microwave reactor at 200 °C for 5 min. The solvent was removed under reduced pressure. Methanol (20 ml) was added, and after sonication for 20 min, the solid was filtered off. It was dissolved in CH_2Cl_2 and filtered through a short plug of silica. After preparative recycling gel-permeation chromatography (CH_2Cl_2) **[9]CPP-PA$_3$** (3.8 %, 4.3 mg), **[12]CPP-PA$_4$** (10 %, 11.8 mg), and **[15]CPP-PA$_5$** (7.6 %, 8.5 mg) were obtained as colorless solids. In enantiopure reactions, all products were furthermore purified by HPLC (CHIRALPAK® IK, CH_2Cl_2/heptane 60/40 – 90/10).

Analytical data for [9]CPP-PA$_3$

^{1}H NMR[1] (600 MHz, $CDCl_3$): δ [ppm] = 7.40 (d, *J* = 8.4 Hz, 12H, H-9), 7.23 (d, *J* = 8.3 Hz, 12H, H-10), 6.97 (s, 6H, H-2), 6.84 (s, 6H, H-7), 6.65 (s, 6H, H-7), 6.57 (s, 6H, H-7), 6.42 (s, 6H, H-7), 4.10 (d, *J* = 14.5 Hz, 6H, H-4), 3.81–3.77 (m, 18H, H-4), 3.71 (d, *J* = 14.7 Hz, 6H, H-4), 3.68 (s, 18H, *O*CH_3), 3.54 (s, 18H, *O*CH_3), 3.52 (s, 18H, *O*CH_3), 3.25 (s, 18H, *O*CH_3).

^{13}C NMR[1] (151 MHz, $CDCl_3$): δ [ppm] = 151.5 (C-6), 151.2 (C-6), 151.0 (C-6), 150.8 (C-6), 139.7 (C-11), 139.3 (C-1), 138.8 (C-8), 136.8 (C-3), 132.8 (C-2), 130.3 (C-10), 128.9 (C-5), 128.7 (C-5), 128.6 (C-5),

128.2 (C-5), 127.2 (C-9), 114.6 (C-7), 114.5 (C-7), 114.3 (C-7), 56.2 (*OCH₃*), 56.1 (*OCH₃*), 56.1 (1*OCH₃*), 29.9 (C-4), 29.8 (C-4).

HRMS (MALDI$^+$): m/z calcd. for $C_{165}H_{156}O_{24}$ [$M^{•+}$]: 2521.0987, found: 2521.1007.

Analytical data for [12]CPP-PA$_4$

^{1}H NMR[1] (600 MHz, $CDCl_3$): *δ* [ppm] = 7.48 (d, *J* = 8.5 Hz, 16H, H-9), 7.25 (d, *J* = 8.5 Hz, 16H, H-10), 7.03 (s, 8H, H-2), 6.81 (s, 8H, H-7), 6.60 (s, 8H, H-7), 6.57 (s, 8H, H-7), 6.31 (s, 8H, H-7), 4.07 (d, *J* = 14.4 Hz, 8H, H-4), 3.80 (d, *J* = 13.5 Hz, 16H, H-4), 3.76–3.71 (m, 16H, H-4), 3.62 (s, 24H, *O*CH_3), 3.52 (s, 24H, *O*CH_3), 3.47 (s, 24H, *O*CH_3), 3.21 (s, 24H, *O*CH_3).

^{13}C NMR[1] (151 MHz, $CDCl_3$): *δ* [ppm] = 151.4 (C-6), 151.3 (C-6), 151.1 (C-6), 150.8 (C-6), 140.5 (C-11), 139.6 (C-1), 139.0 (C-8), 136.7 (C-3), 132.9 (C-2), 130.2 (C-10), 129.3 (C-5), 129.03 (C-5), 128.99 (C-5), 128.4 (C-5), 127.0 (C-9), 114.49 (C-7), 114.45 (C-7), 114.3 (C-7), 56.3 (*OCH$_3$*), 56.1 (*OCH$_3$*), 56.00 (*OCH$_3$*), 55.96 (*OCH$_3$*), 33.4 (C-4), 30.87 (C-4), 30.84 (C-4).

HRMS (MALDI$^+$): m/z calcd. for $C_{220}H_{208}O_{32}$ [$M^{•+}$]: 3361.4649, found: 3361.4409.

Analytical data for [15]CPP-PA$_5$

^{1}H NMR[1] (600 MHz, $CDCl_3$): *δ* [ppm] = 7.51 (d, *J* = 8.2 Hz, 20H, H-9), 7.23 (d, *J* = 8.2 Hz, 20H, H-10), 7.04 (s, 10H, H-2), 6.78 (s, 10H, H-7), 6.62 (s, 10H, H-7), 6.58 (s, 10H, H-7), 6.28 (s, 10H, H-7), 4.03 (d, *J* = 14.4 Hz, 10H, H-4), 3.84 – 3.74 (m, 40H, H-4), 3.61 (s, 30H, *O*CH_3), 3.57 (s, 30H, *O*CH_3), 3.42 (s, 30H, *O*CH_3), 3.28 (s, 30H, *O*CH_3).

^{13}C NMR[1] (151 MHz, $CDCl_3$): *δ* [ppm] = 151.28 (C-6), 151.26 (C-6), 151.1 (C-6), 150.9 (C-6), 140.9 (C-11), 139.8 (C-8), 139.2 (C-3), 136.7 (C-3), 132.7 (C-2), 130.1 (C-10), 129.1 (C-5), 129.0 (C-5), 128.5 (C-5), 126.9 (C-9), 114.52 (C-7), 114.47 (C-7), 114.4 (C-7), 56.3 (*OCH$_3$*), 56.2 (*OCH$_3$*), 56.0 (*OCH$_3$*), 55.9 (*OCH$_3$*), 33.5 (C-4), 32.1 (C-4), 30.8 (C-4).

HRMS (MALDI$^+$): m/z calcd. for $C_{275}H_{260}O_{40}$ [$M^{•+}$]: 4201.8312, found: 4201.8458.

3. Assignment of Stereodescriptors

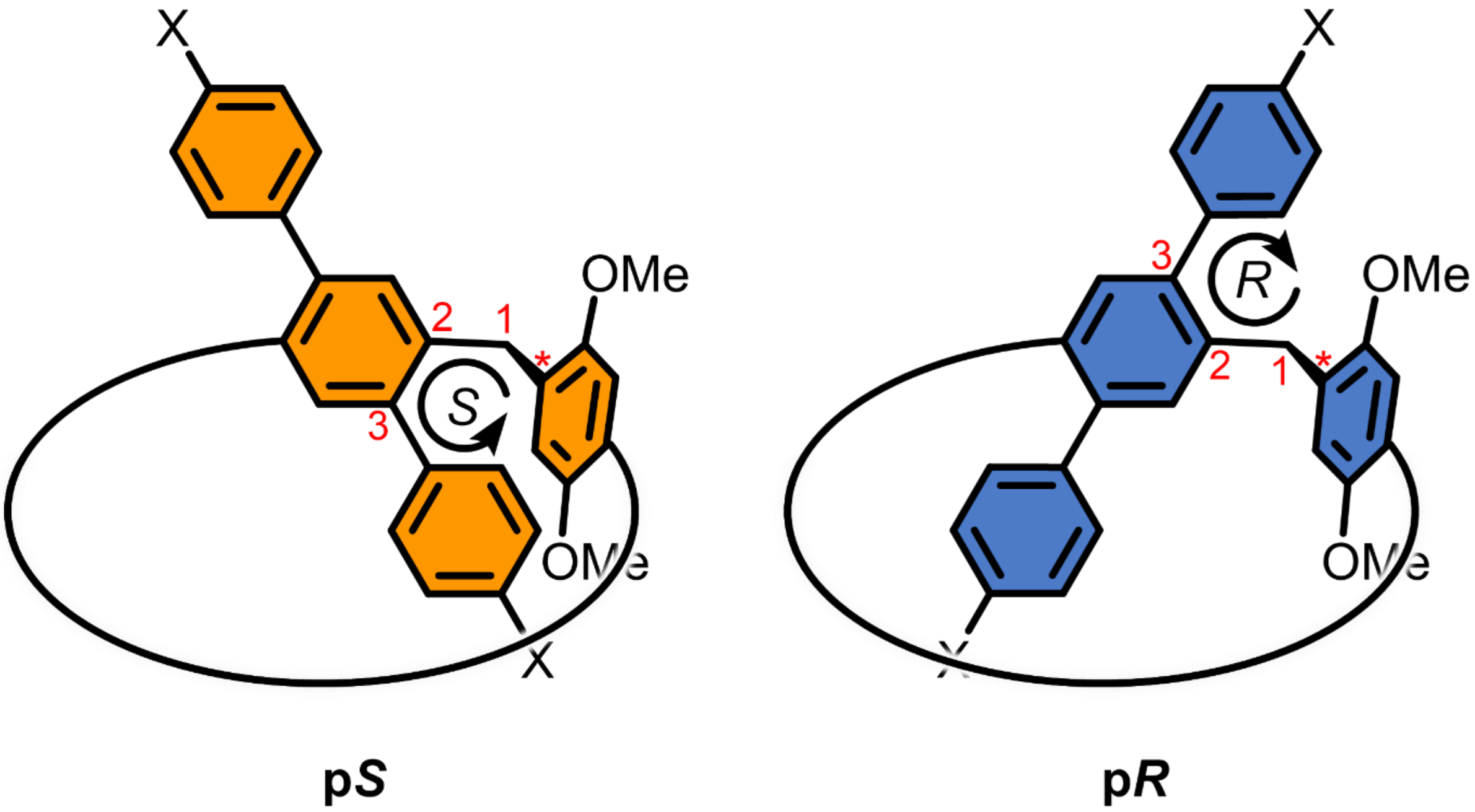

Figure S 1: Assignment of stereodescriptors p*S* (orange) and p*R* (blue). The terphenyl unit marks the chirality plane, atoms 1, 2 and 3 lie in this plane. The highest priority atom directly attached to an atom in the chiral plane, but not in the plane itself, is the pilot atom, here marked with an asterisk. The next three atoms in the chirality plane following the highest priority path from the pilot atom are numbered from 1 – 3. The sense of revolution of these three atoms as seen from the pilot atom defines the planar chirality configuration p*S* or p*R*. The above shown assignment holds regardless of the nature of group X.

4. NMR Spectra

4.1 1D NMR Spectra

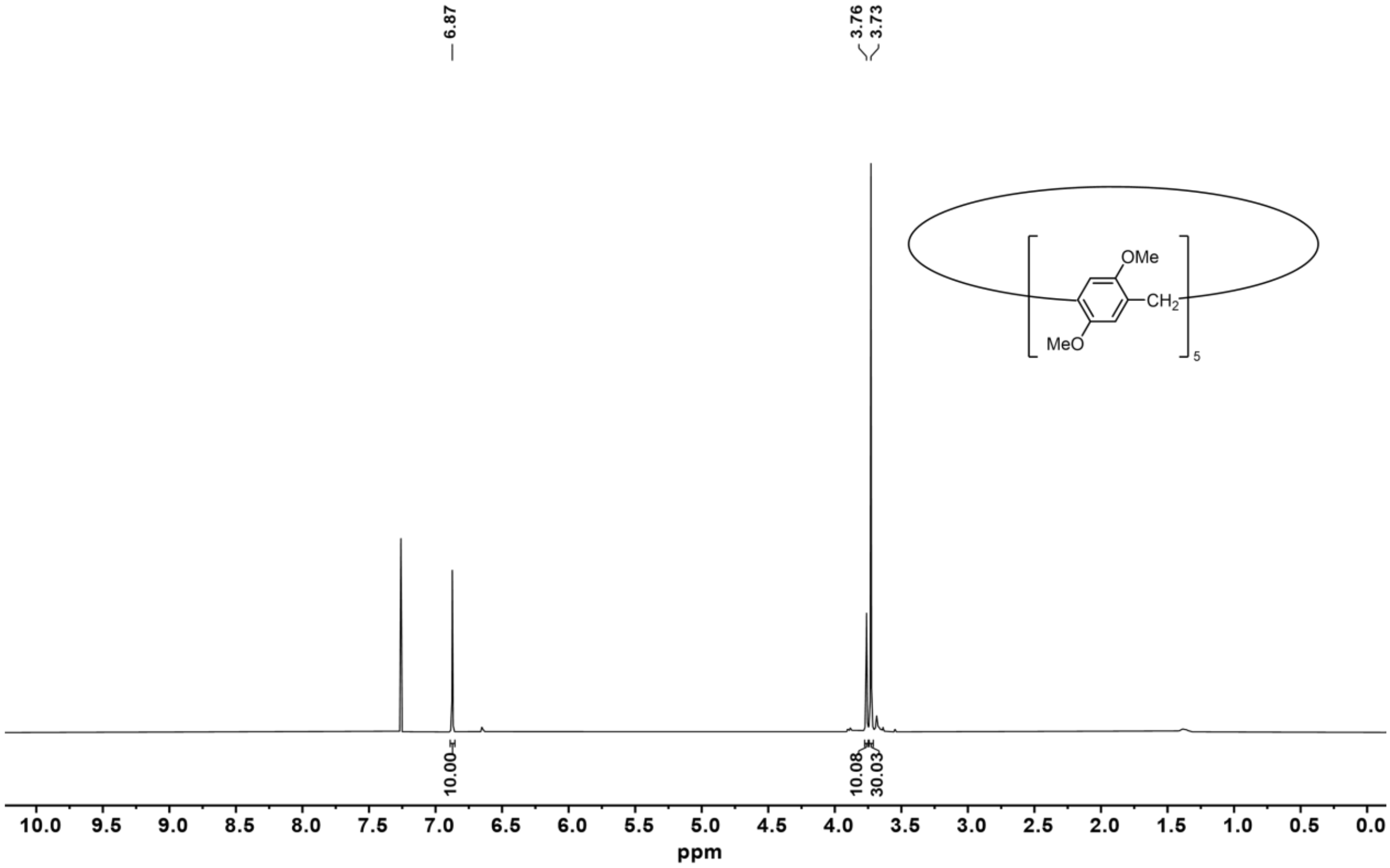

Figure S 2: ^{1}H NMR spectrum of **S1** ($CDCl_3$, 298 K, 400 MHz).

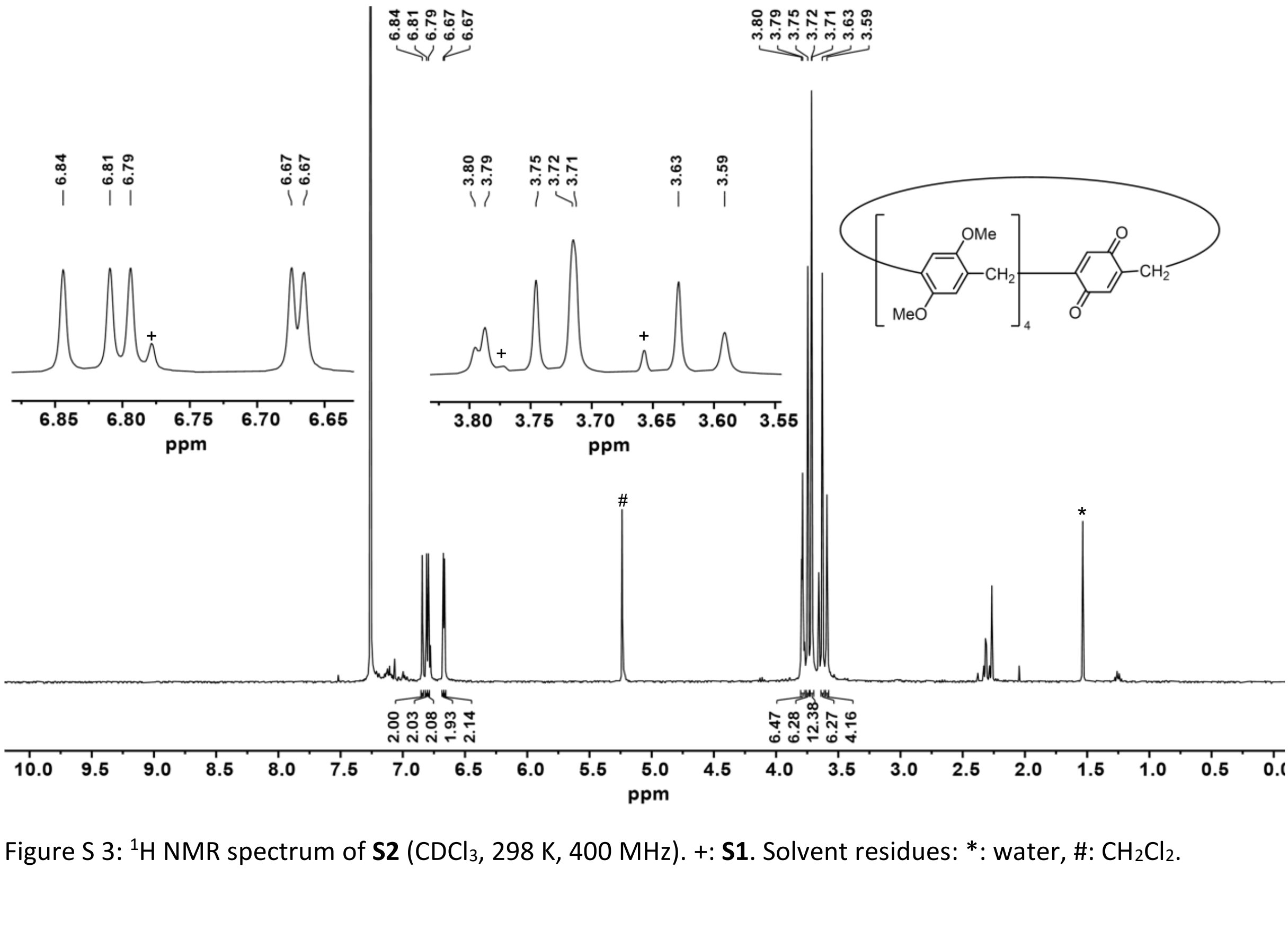

Figure S 3: ^{1}H NMR spectrum of **S2** ($CDCl_3$, 298 K, 400 MHz). +: **S1**. Solvent residues: *: water, #: CH_2Cl_2.

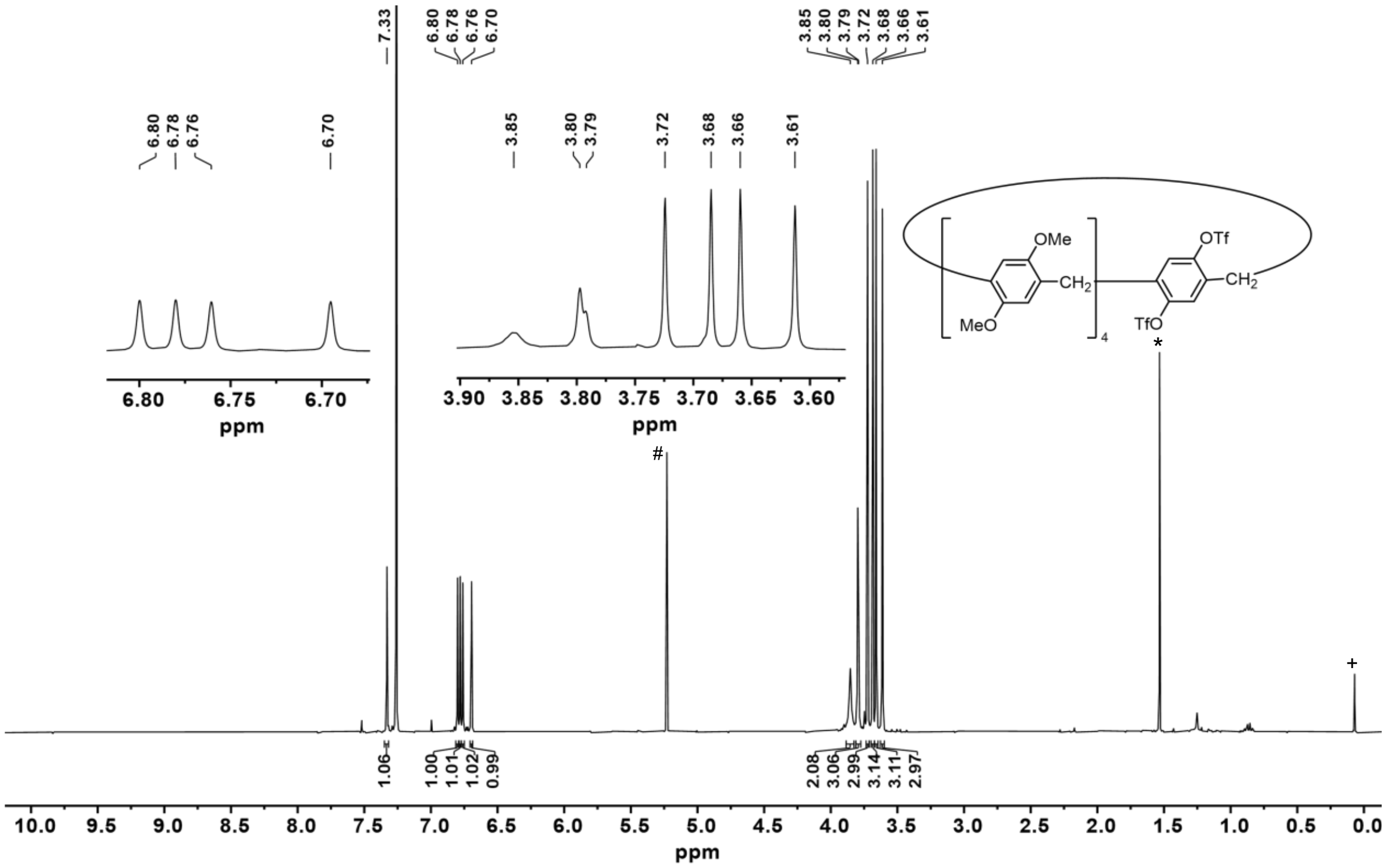

Figure S 4: ^{1}H NMR spectrum of **2** ($CDCl_3$, 298 K, 400 MHz). Solvent residues: *: water, #: CH_2Cl_2,+: H grease.

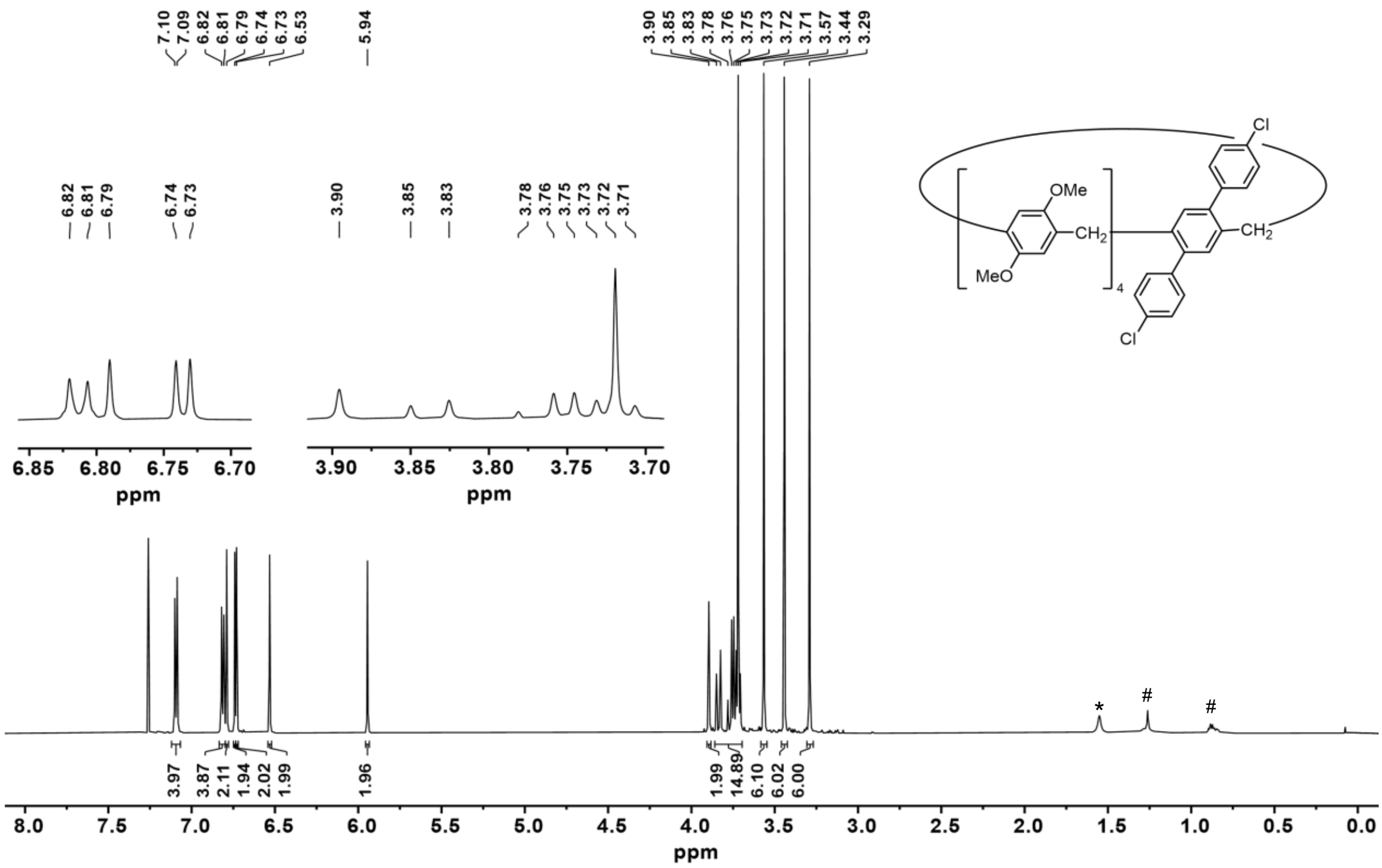

Figure S 5: ^{1}H NMR spectrum of **3** ($CDCl_3$, 298 K, 600 MHz). Solvent residues: *: water, #: H grease.

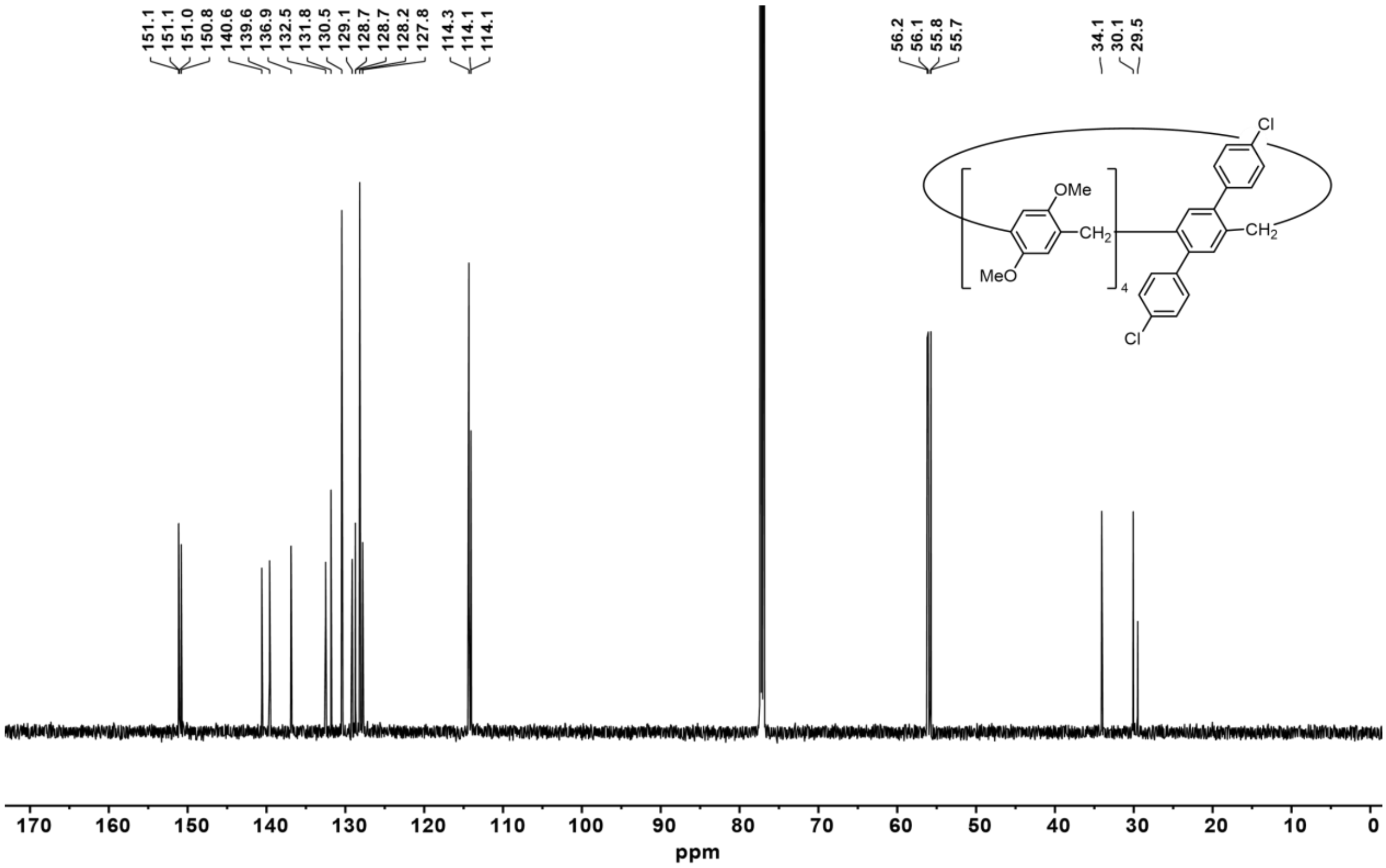

Figure S 6: ^{13}C NMR spectrum of **3** ($CDCl_3$, 298 K, 151 MHz).

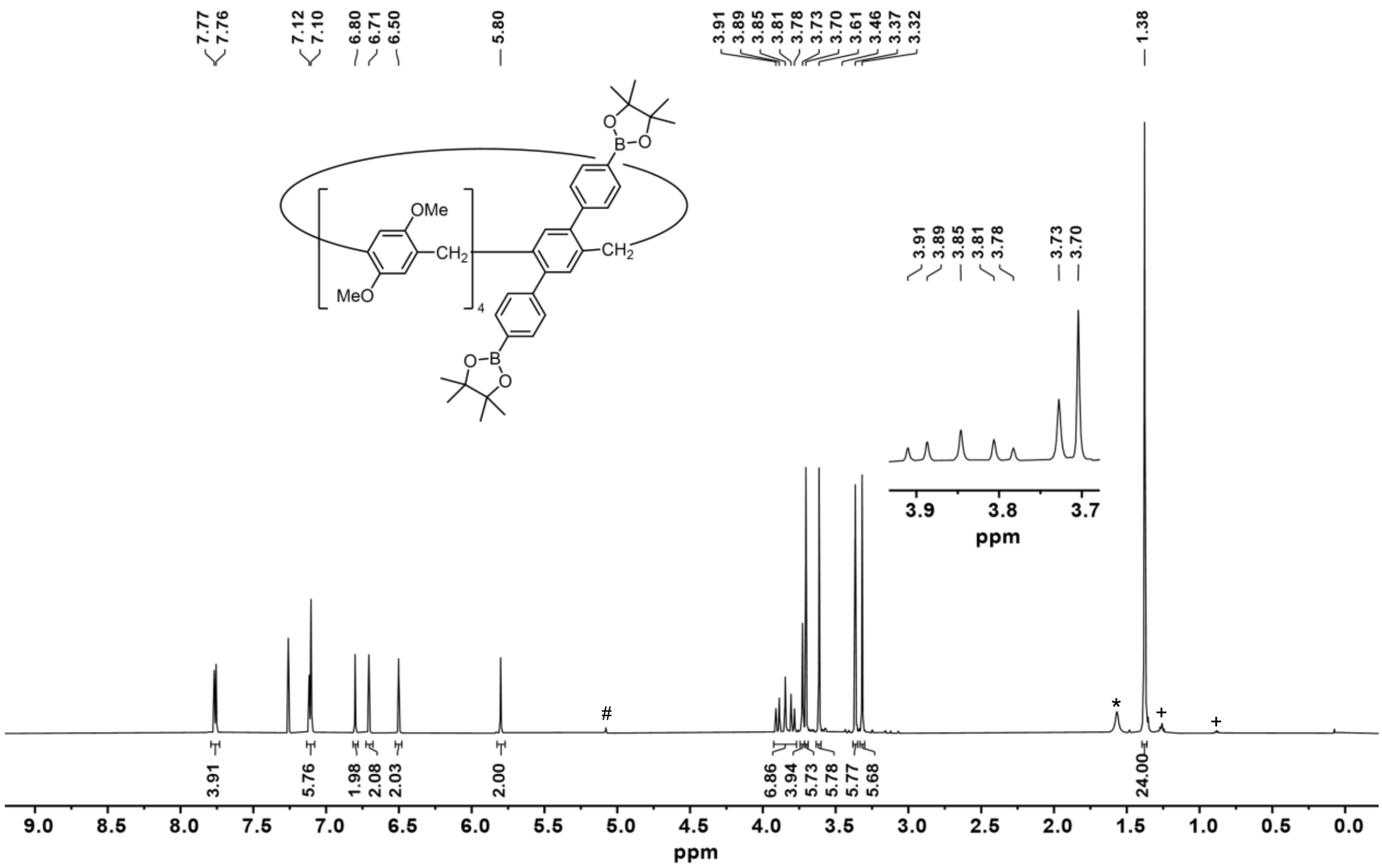

Figure S 7: ^{1}H NMR spectrum of **1** ($CDCl_3$, 298 K, 600 MHz). Solvent residues: *: water, #: CH_2Cl_2,+: H grease.

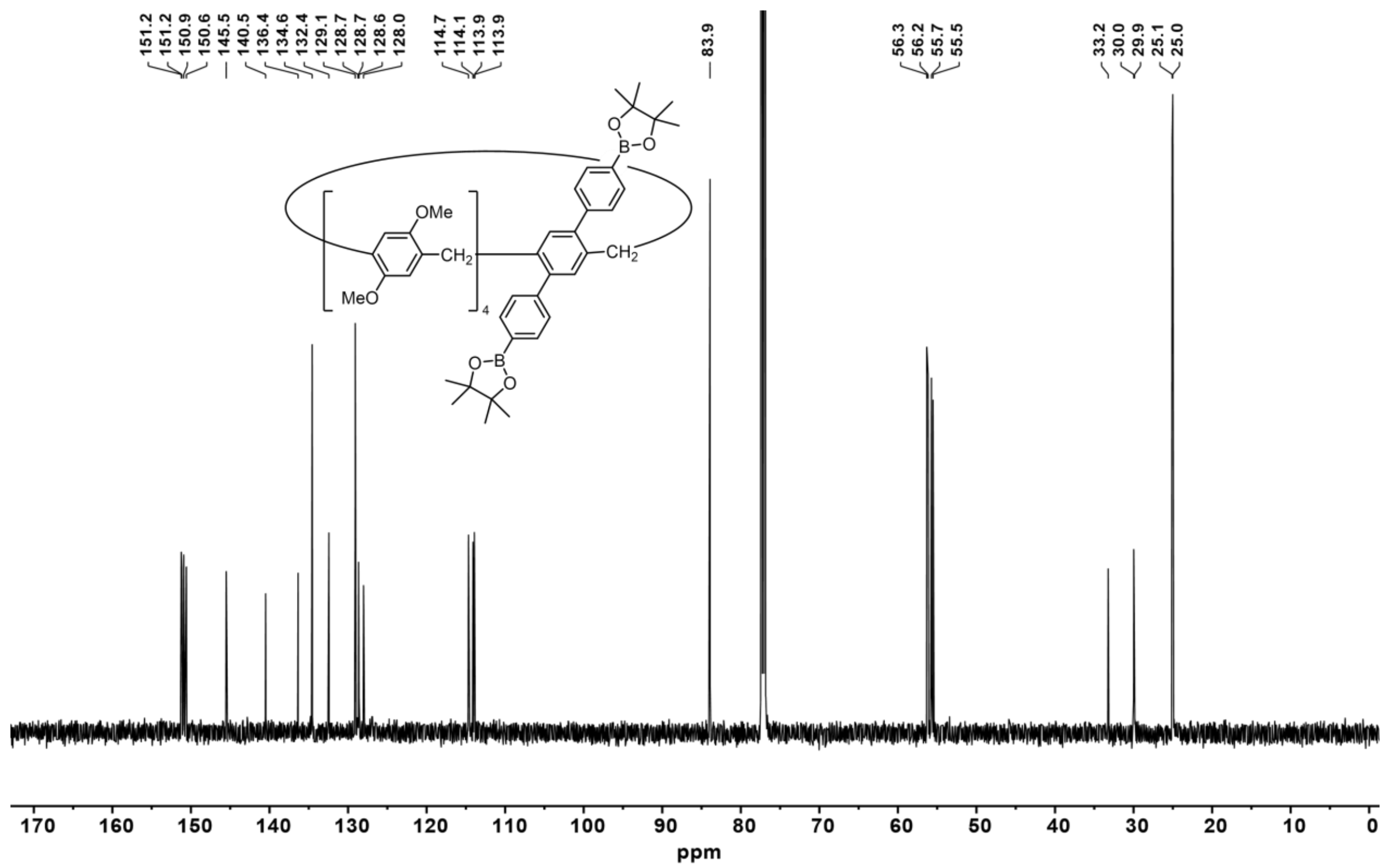

Figure S 8: ^{13}C NMR spectrum of **1** ($CDCl_3$, 298 K, 151 MHz).

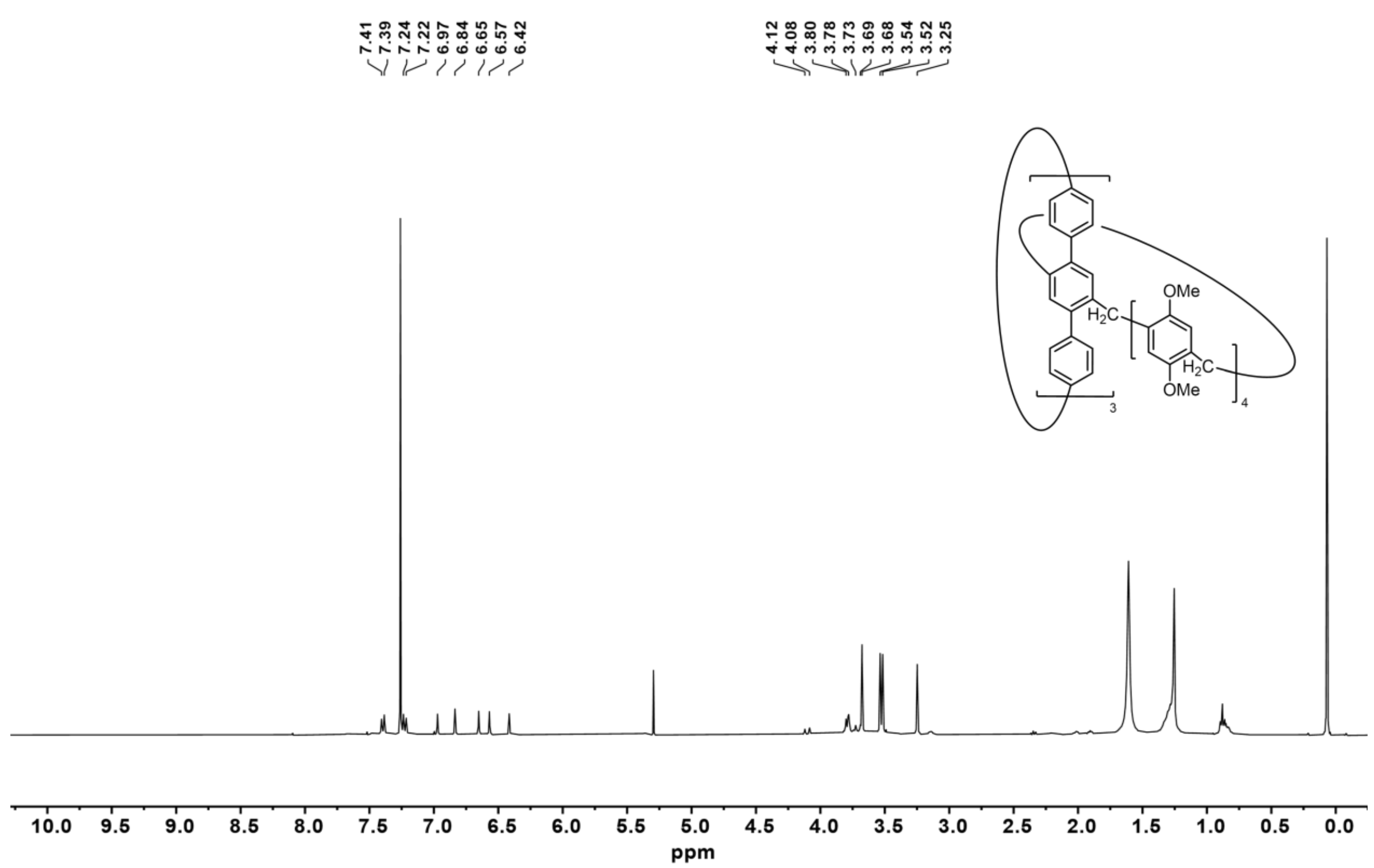

Figure S 9: ^{1}H NMR spectrum of **all-p*R*/all-p*S*-[9]CPP-PA₃** ($CDCl_3$, 298 K, 600 MHz).

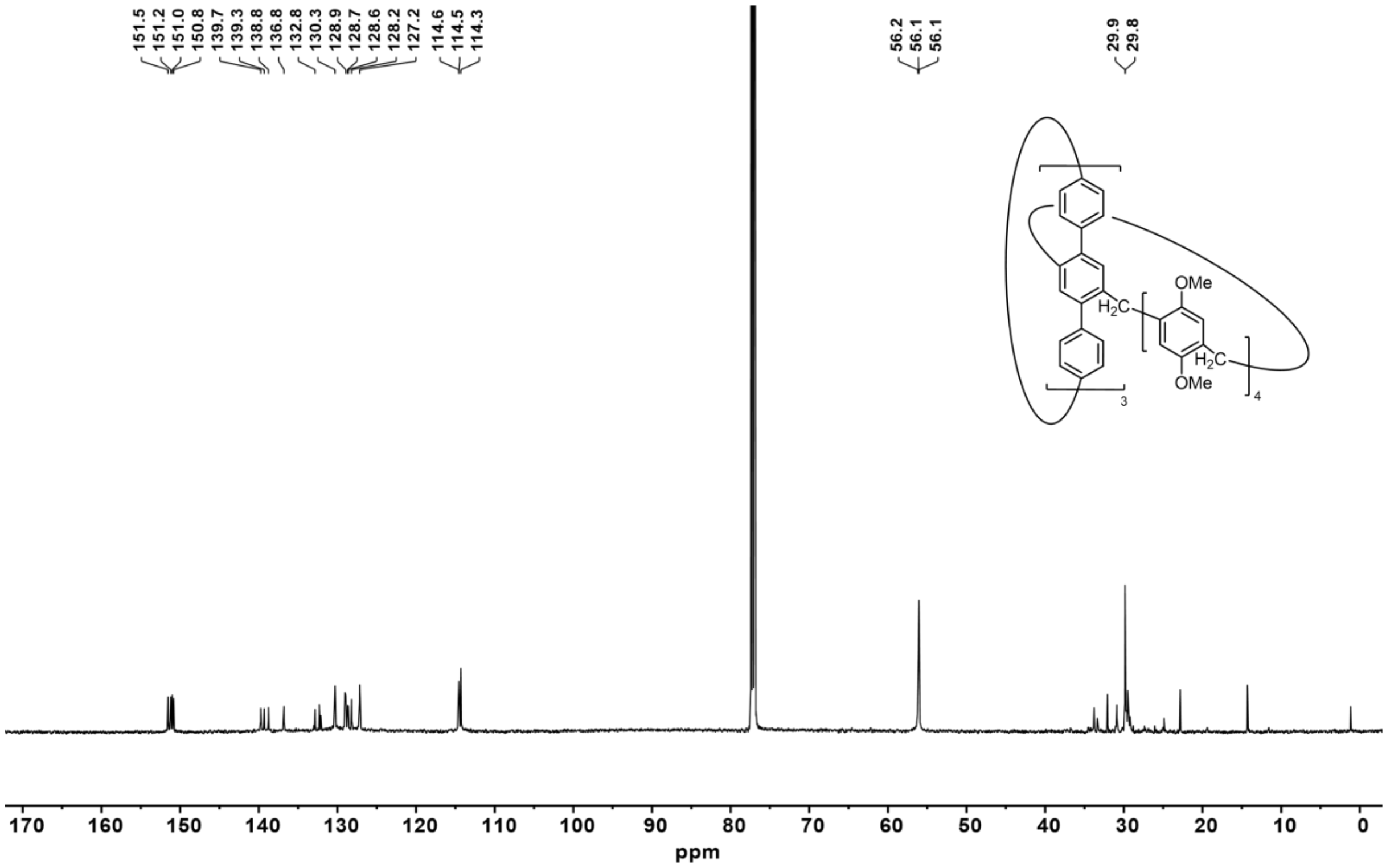

Figure S 10: ^{13}C NMR spectrum of **all-p*R*/all-p*S*-[9]CPP-PA$_3$** ($CDCl_3$, 298 K, 151 MHz).

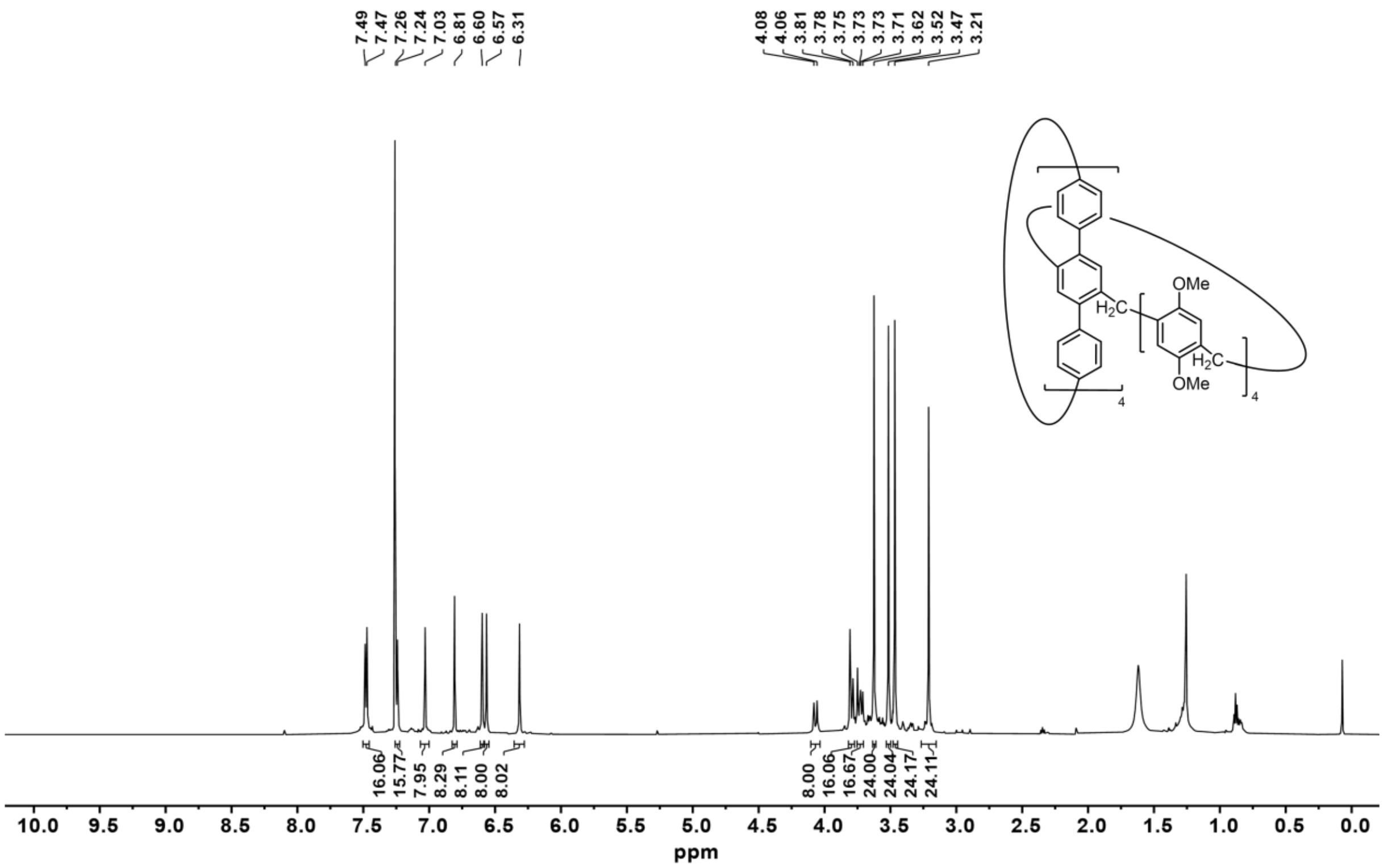

Figure S 11: ^{1}H NMR spectrum of **all-p*R*/all-p*S*-[12]CPP-PA$_4$** ($CDCl_3$, 298 K, 600 MHz).

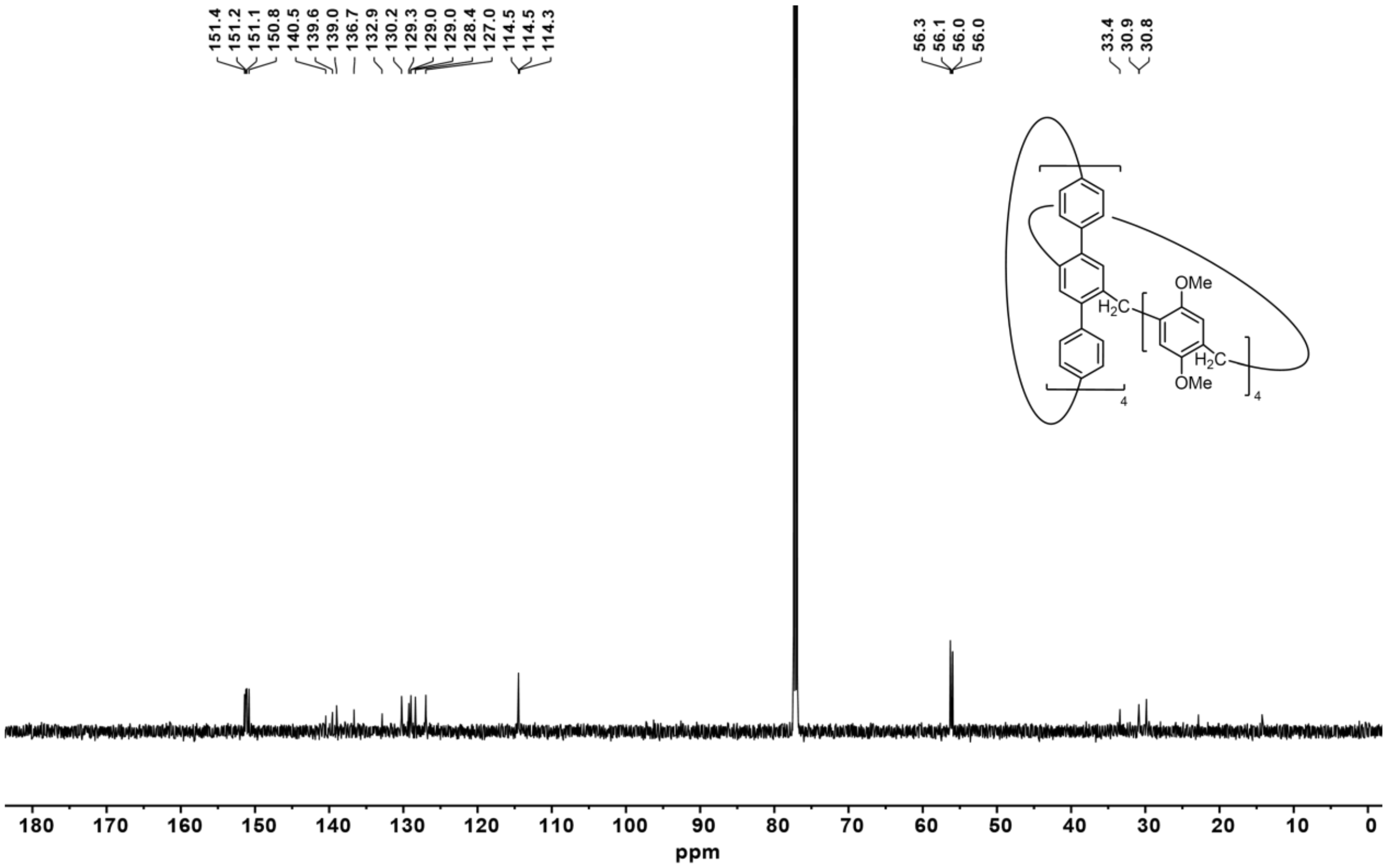

Figure S 12: ^{13}C NMR spectrum of **all-p*R*/all-p*S*-[12]CPP-PA$_4$** ($CDCl_3$, 298 K, 151 MHz).

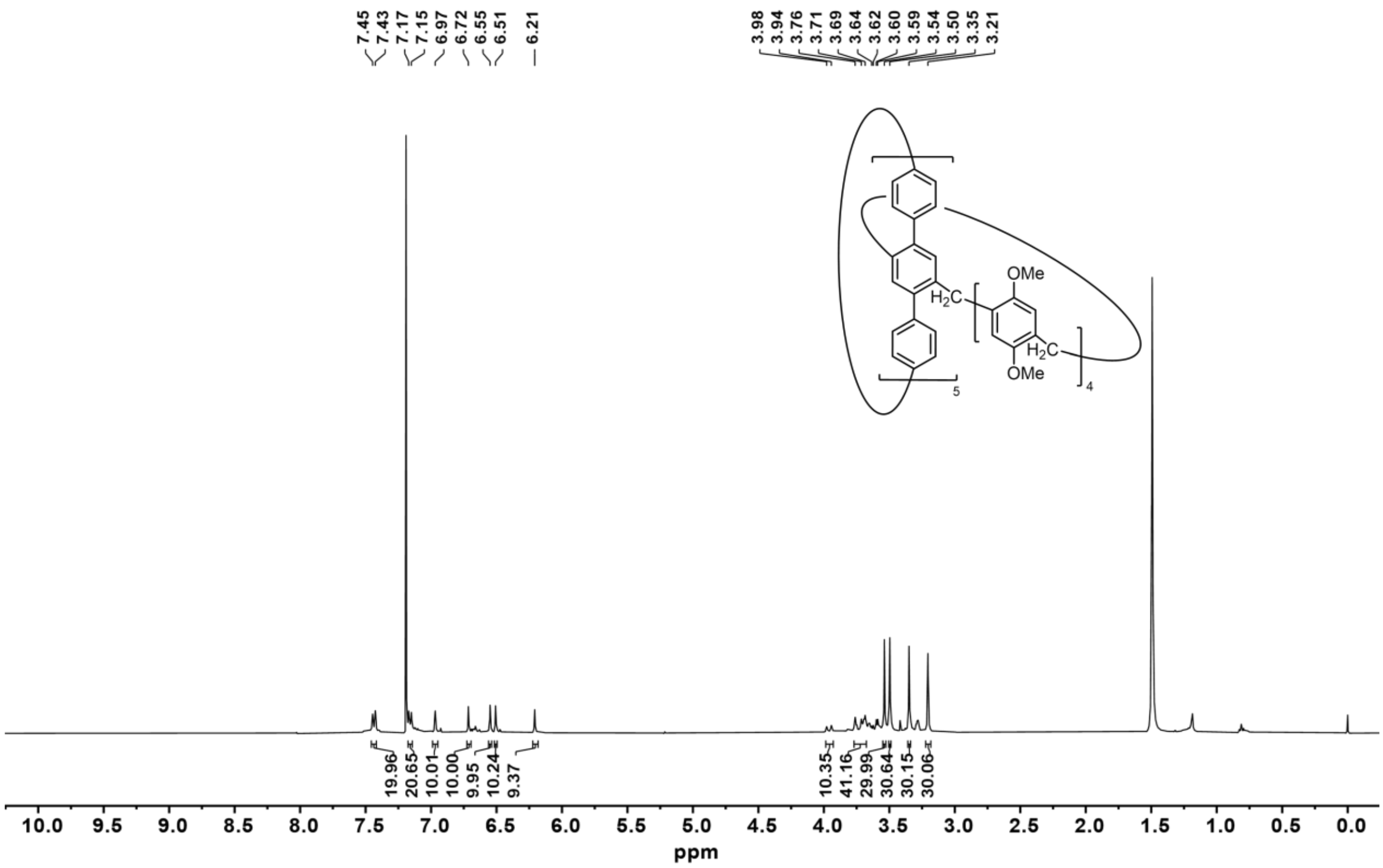

Figure S 13: ^{1}H NMR spectrum of **all-p*R*/all-p*S*-[15]CPP-PA$_5$** ($CDCl_3$, 298 K, 600 MHz).

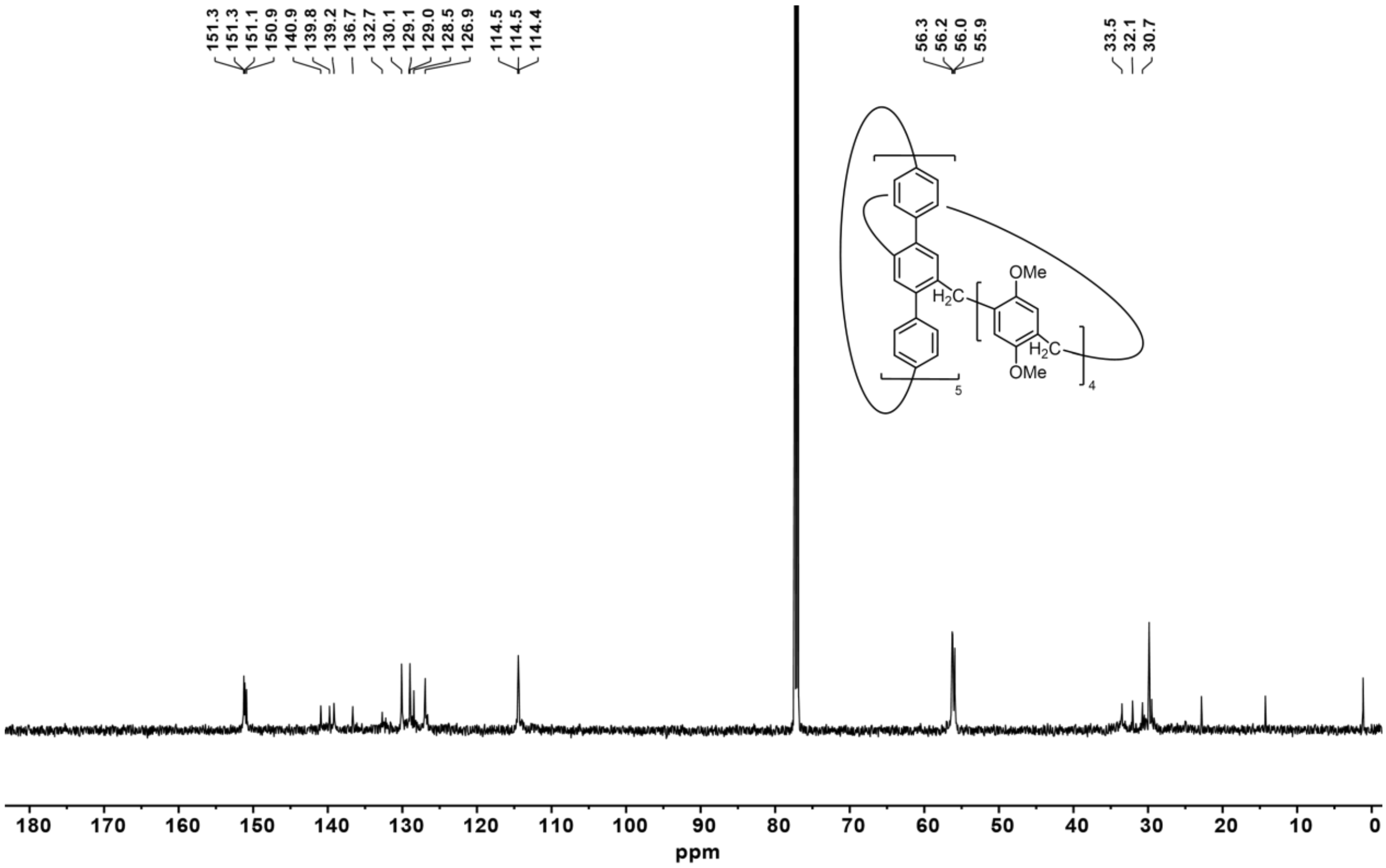

Figure S 14: ^{13}C NMR spectrum of **all-p*R*/all-p*S*-[15]CPP-PA$_5$** ($CDCl_3$, 298 K, 151 MHz).

4.2 2D NMR Spectra

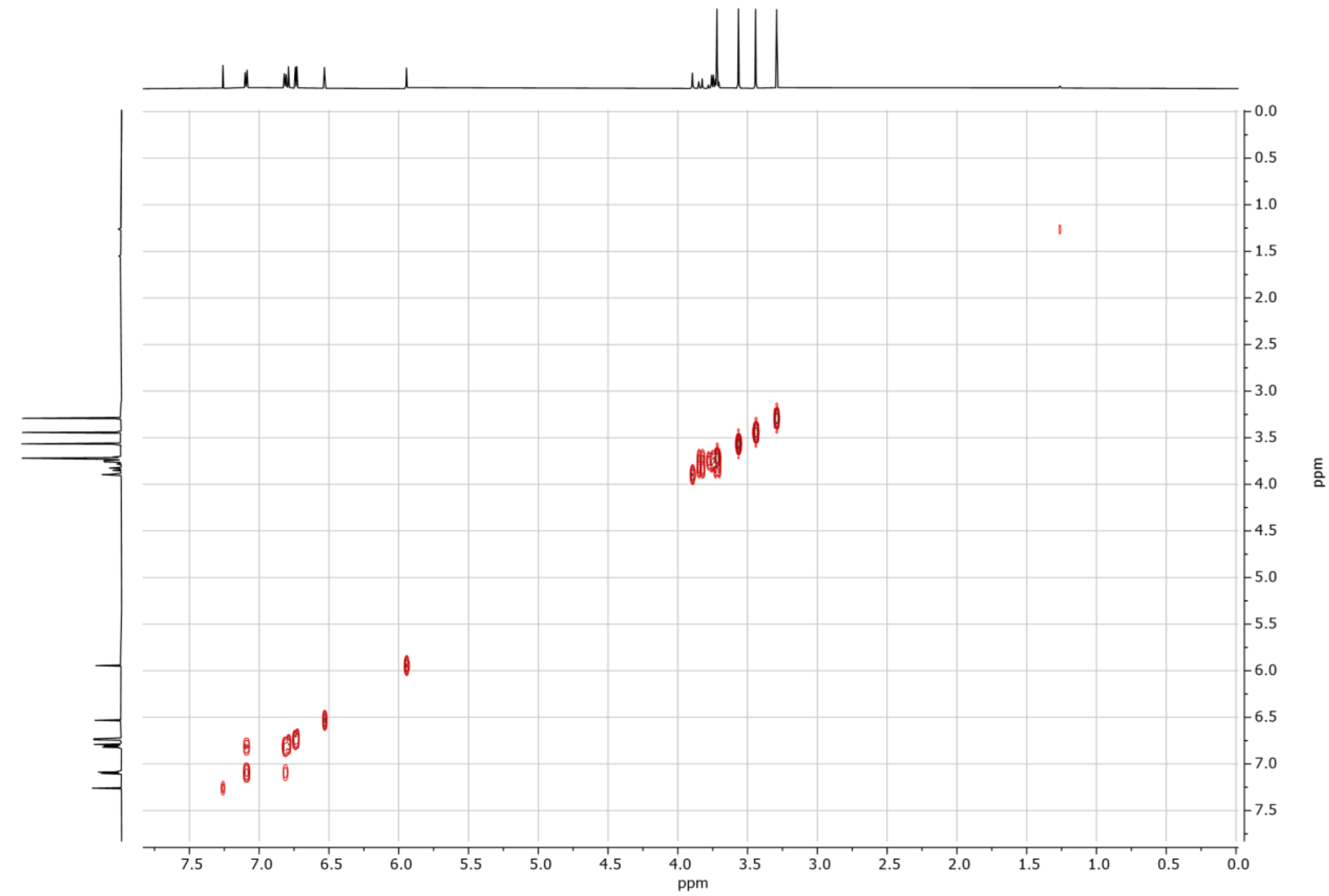

Figure S 15: ^{1}H,^{1}H COSY NMR spectrum of **3** ($CDCl_3$, 298 K, 600 MHz).

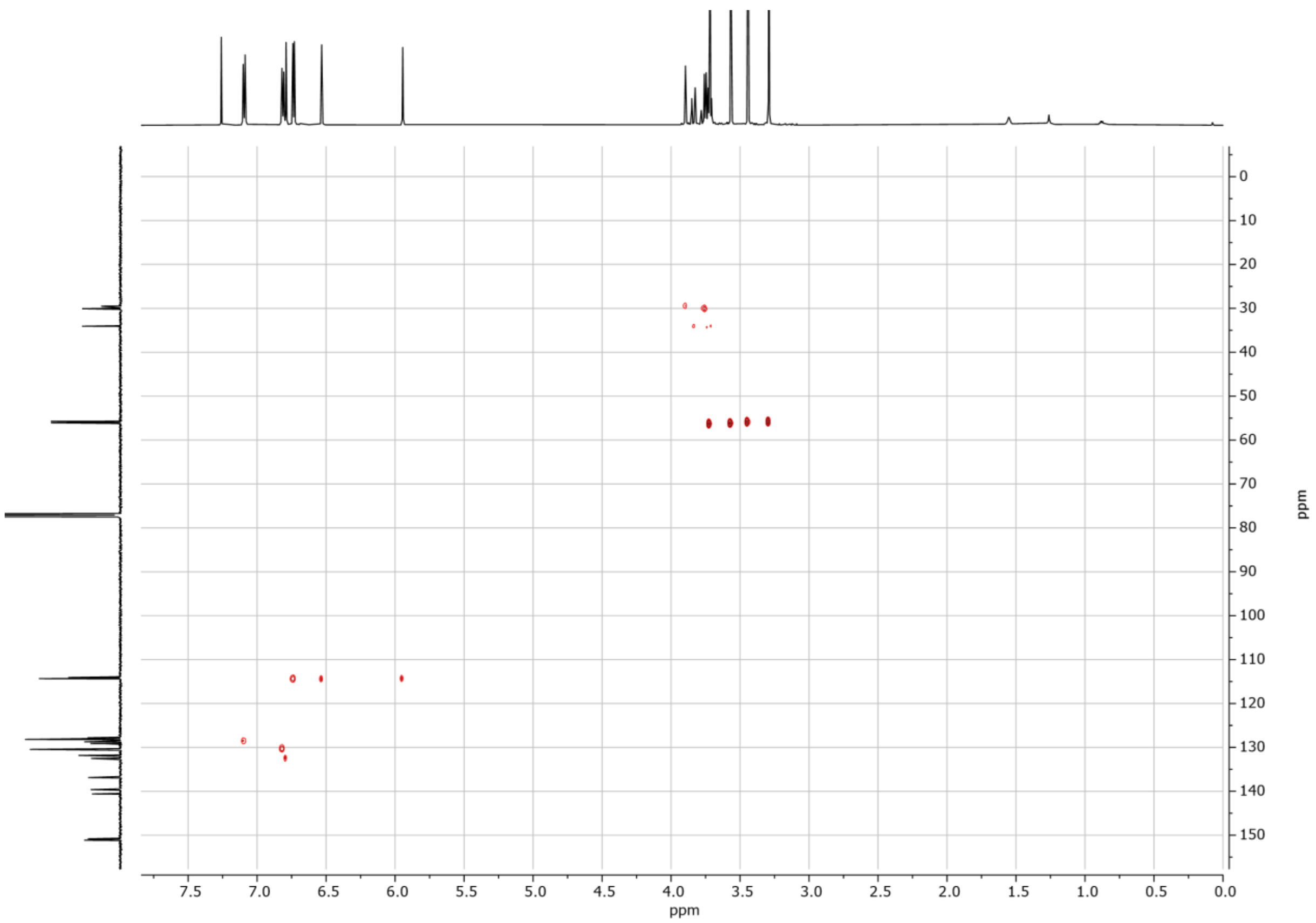

Figure S 16: $^{1}H,^{13}C$ HSQC NMR spectrum of **3** ($CDCl_3$, 298 K, 600 MHz, 151 MHz).

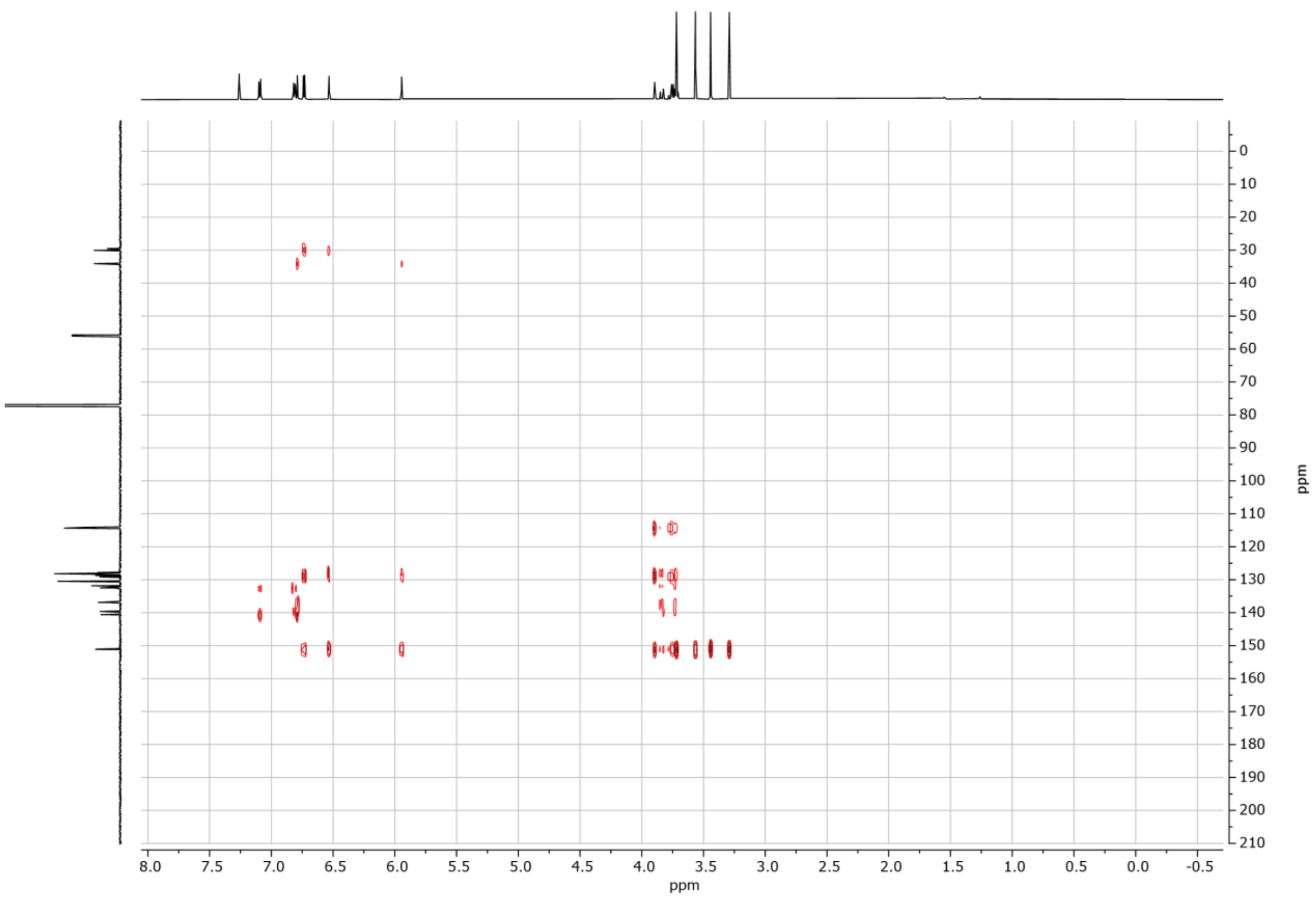

Figure S 17: $^{1}H,^{13}C$ HMBC NMR spectrum of **3** ($CDCl_3$, 298 K, 600 MHz, 151 MHz).

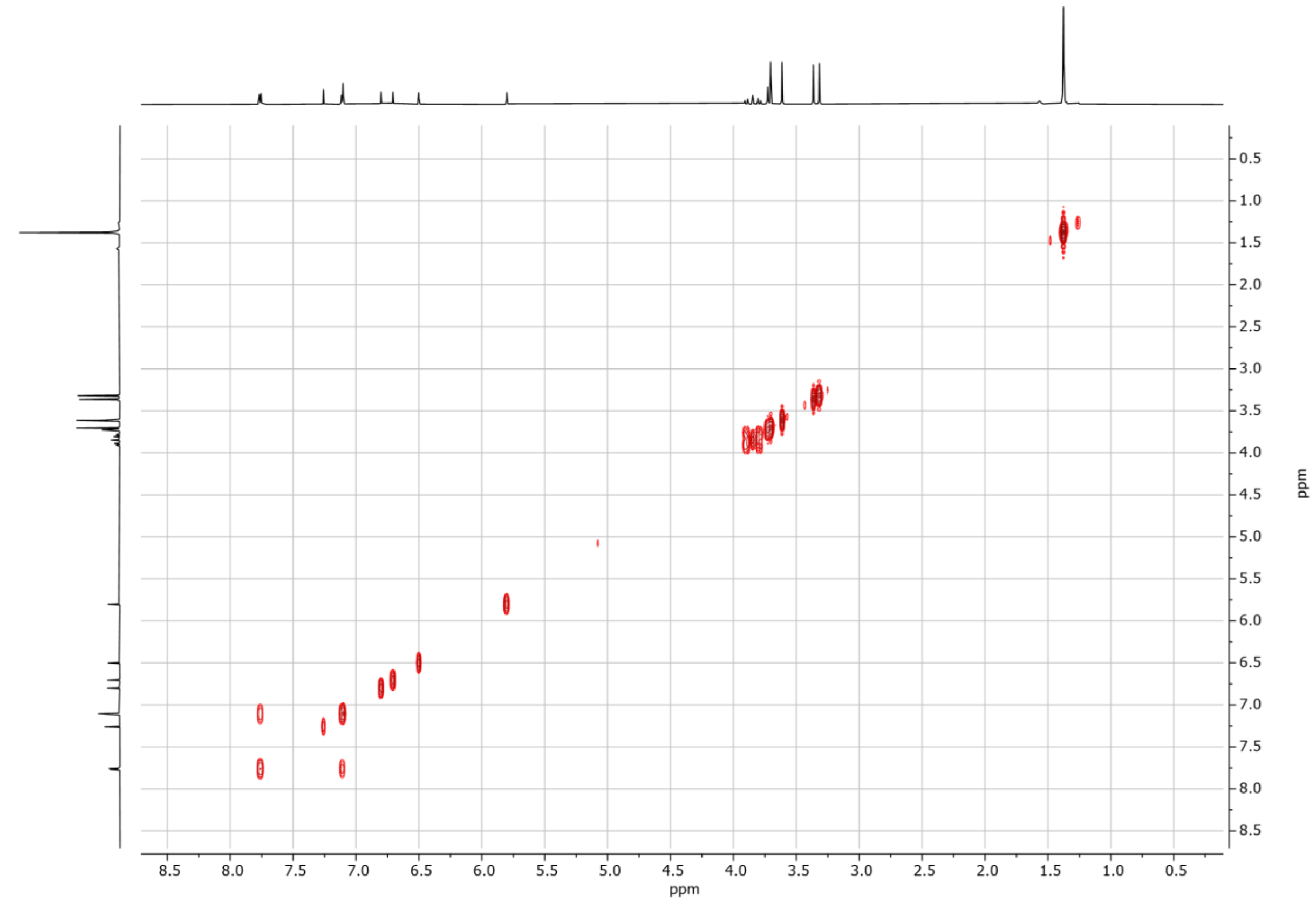

Figure S 18: $^{1}H,^{1}H$ COSY NMR spectrum of **1** ($CDCl_3$, 298 K, 600 MHz).

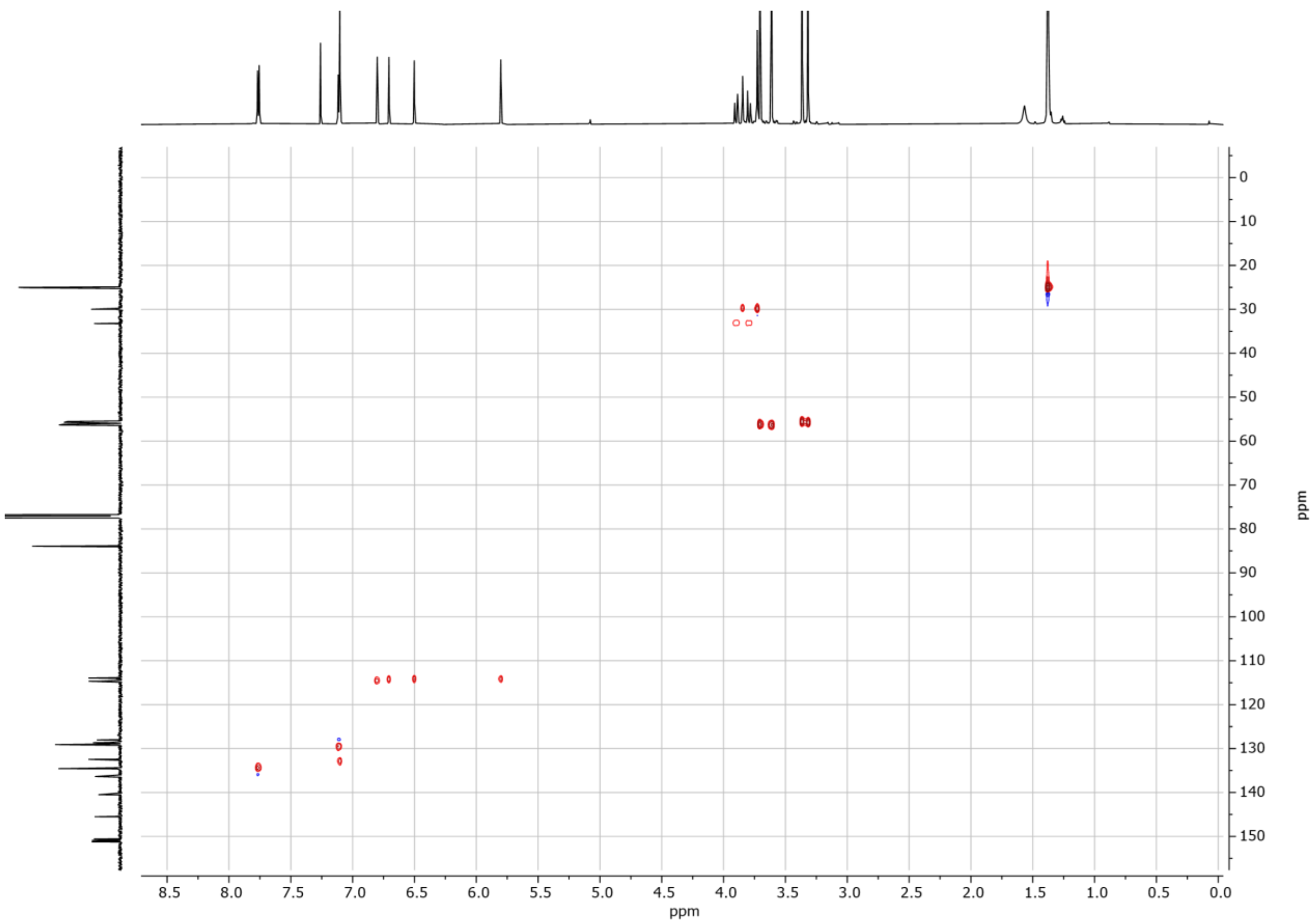

Figure S 19: $^{1}H,^{13}C$ HSQC NMR spectrum of **1** ($CDCl_3$, 298 K, 600 MHz, 151 MHz).

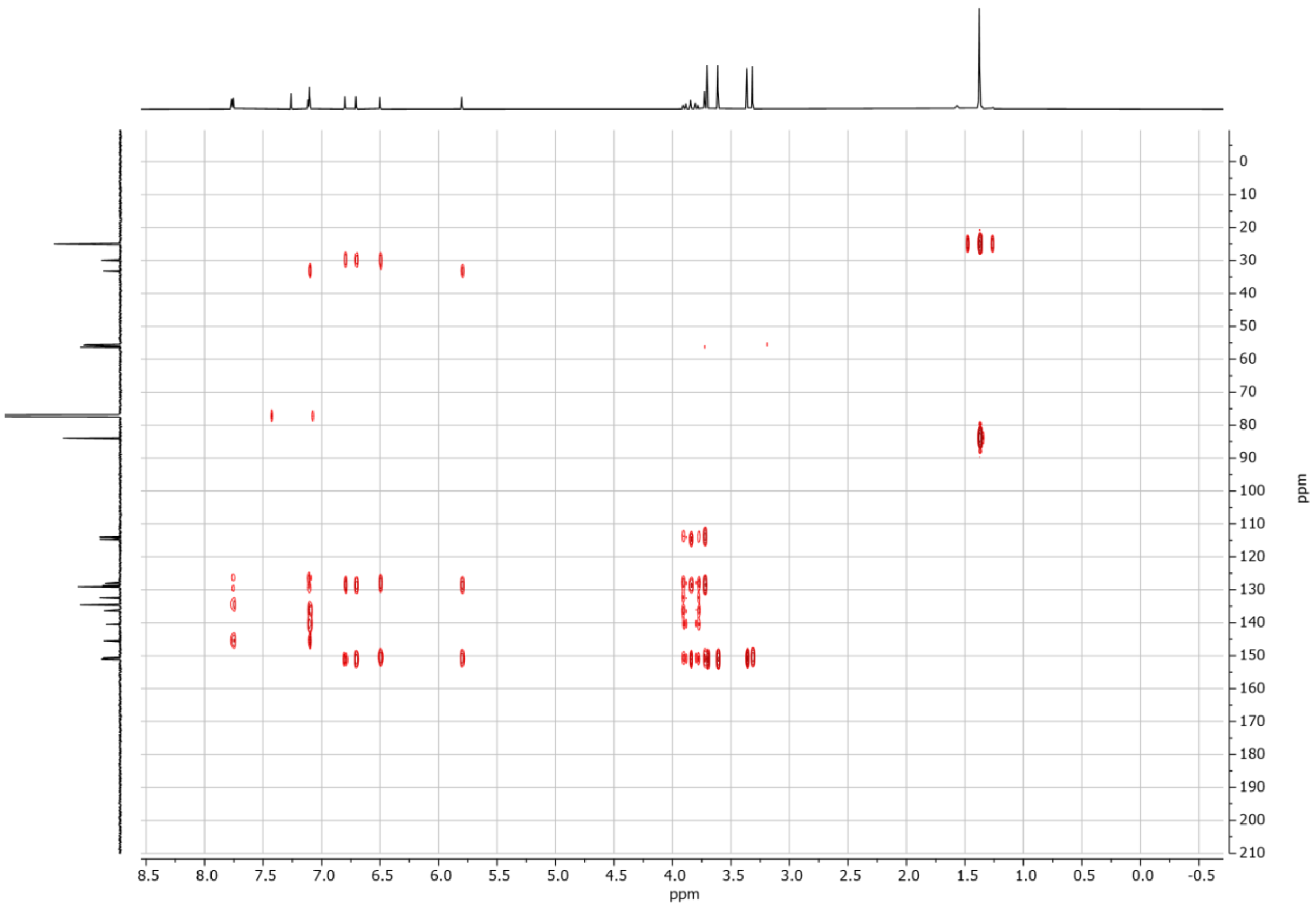

Figure S 20: ^{1}H,^{13}C HMBC NMR spectrum of **1** ($CDCl_3$, 298 K, 600 MHz, 151 MHz).

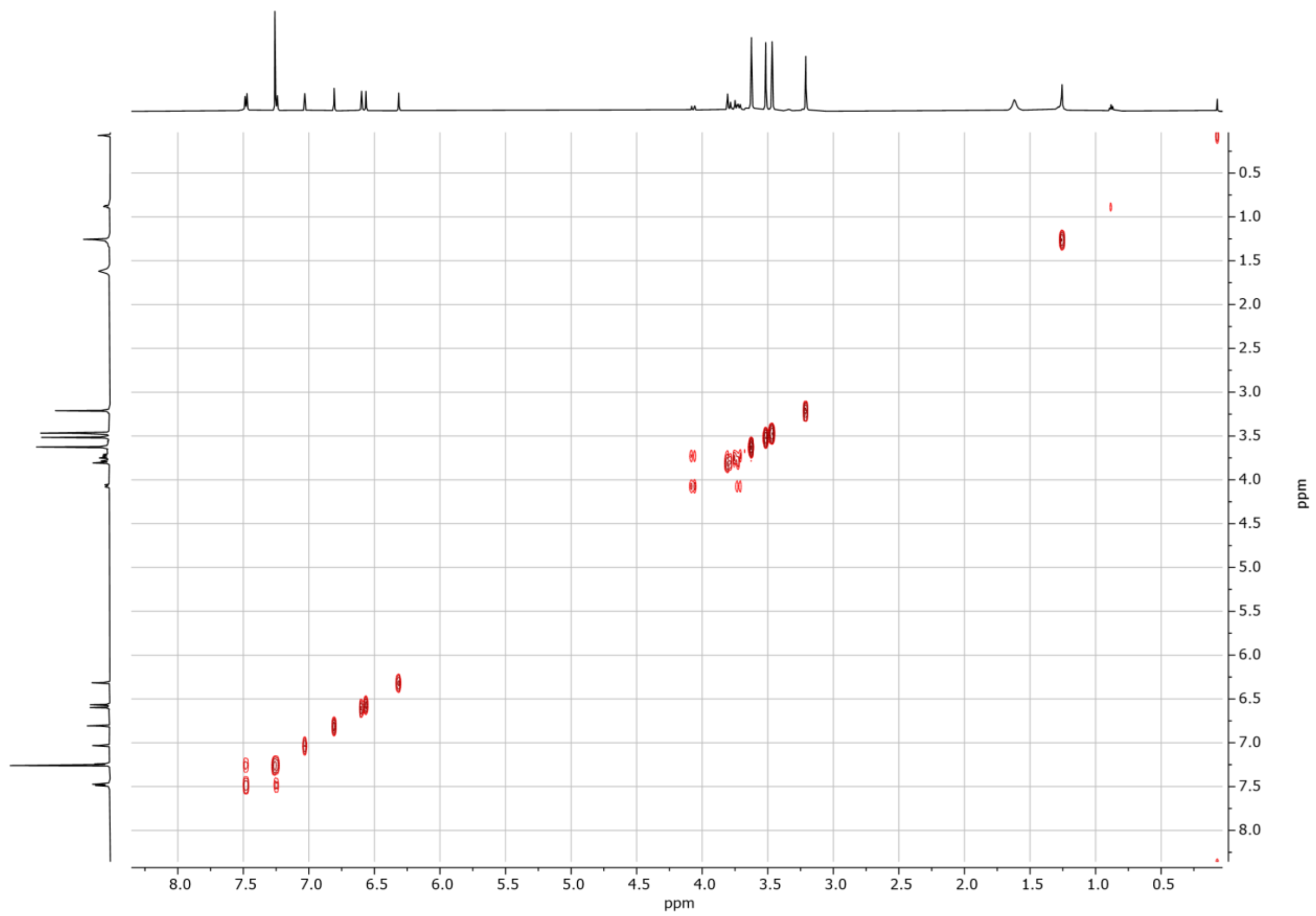

Figure S 21: ^{1}H,^{1}H COSY NMR spectrum of **all-p*R*/all-p*S*-[12]CPP-PA$_4$** ($CDCl_3$, 298 K, 600 MHz).

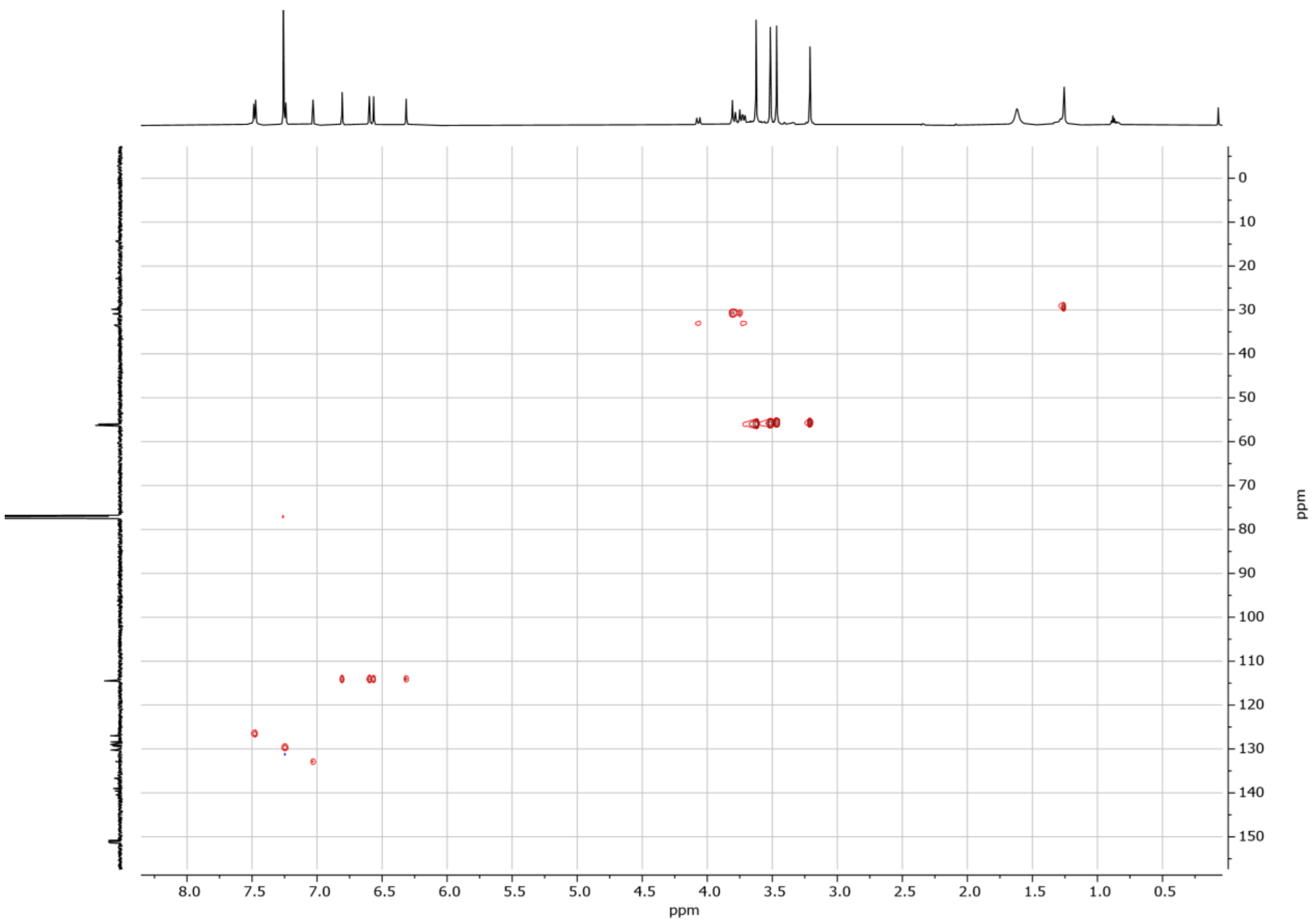

Figure S 22: $^{1}H,^{13}C$ HSQC NMR spectrum of **all-p*R*/all-p*S*-[12]CPP-PA$_4$** ($CDCl_3$, 298 K, 600 MHz, 151 MHz).

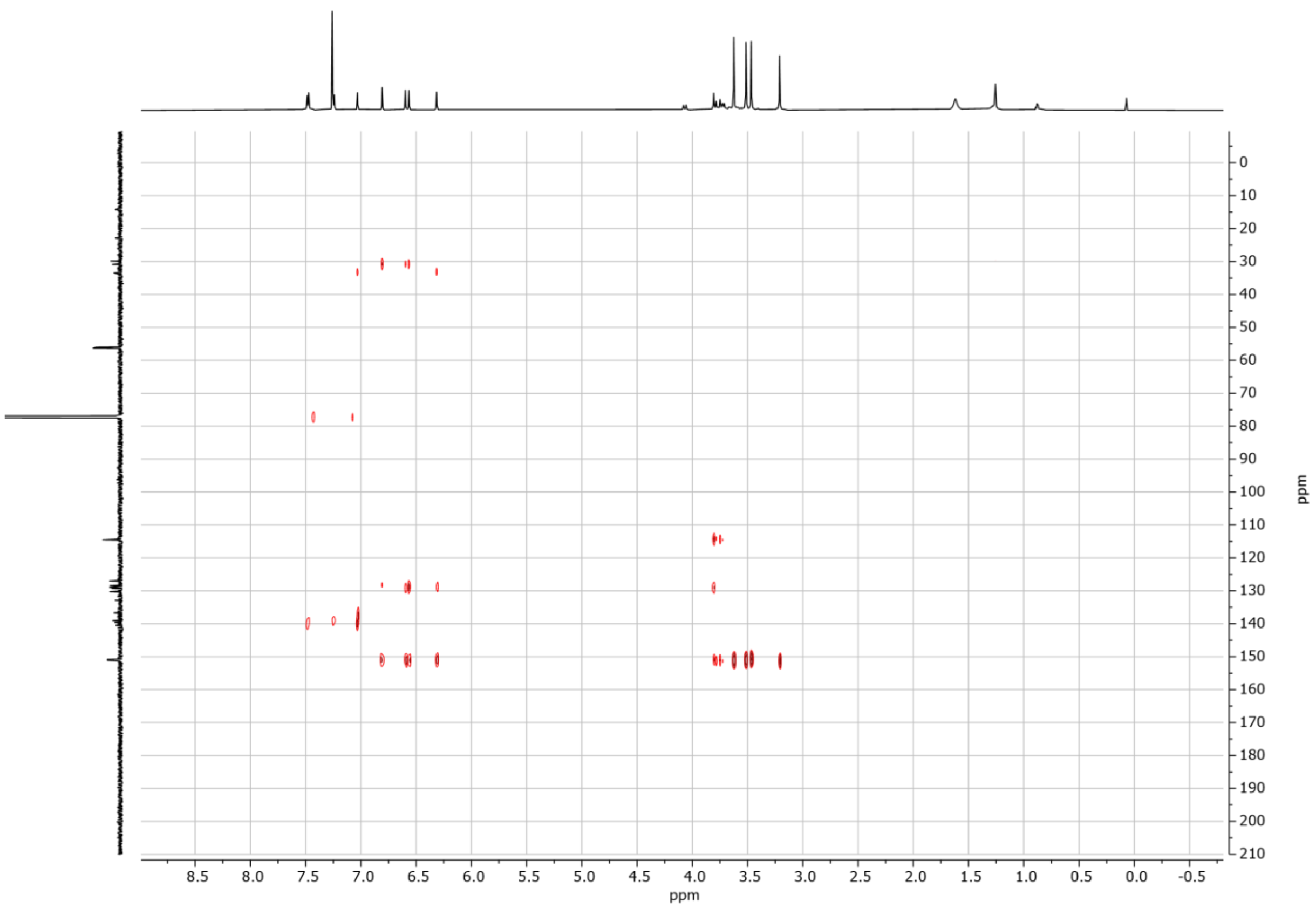

Figure S 23: $^{1}H,^{13}C$ HMBC NMR spectrum of **all-p*R*/all-p*S*-[12]CPP-PA$_4$** ($CDCl_3$, 298 K, 600 MHz, 151 MHz).

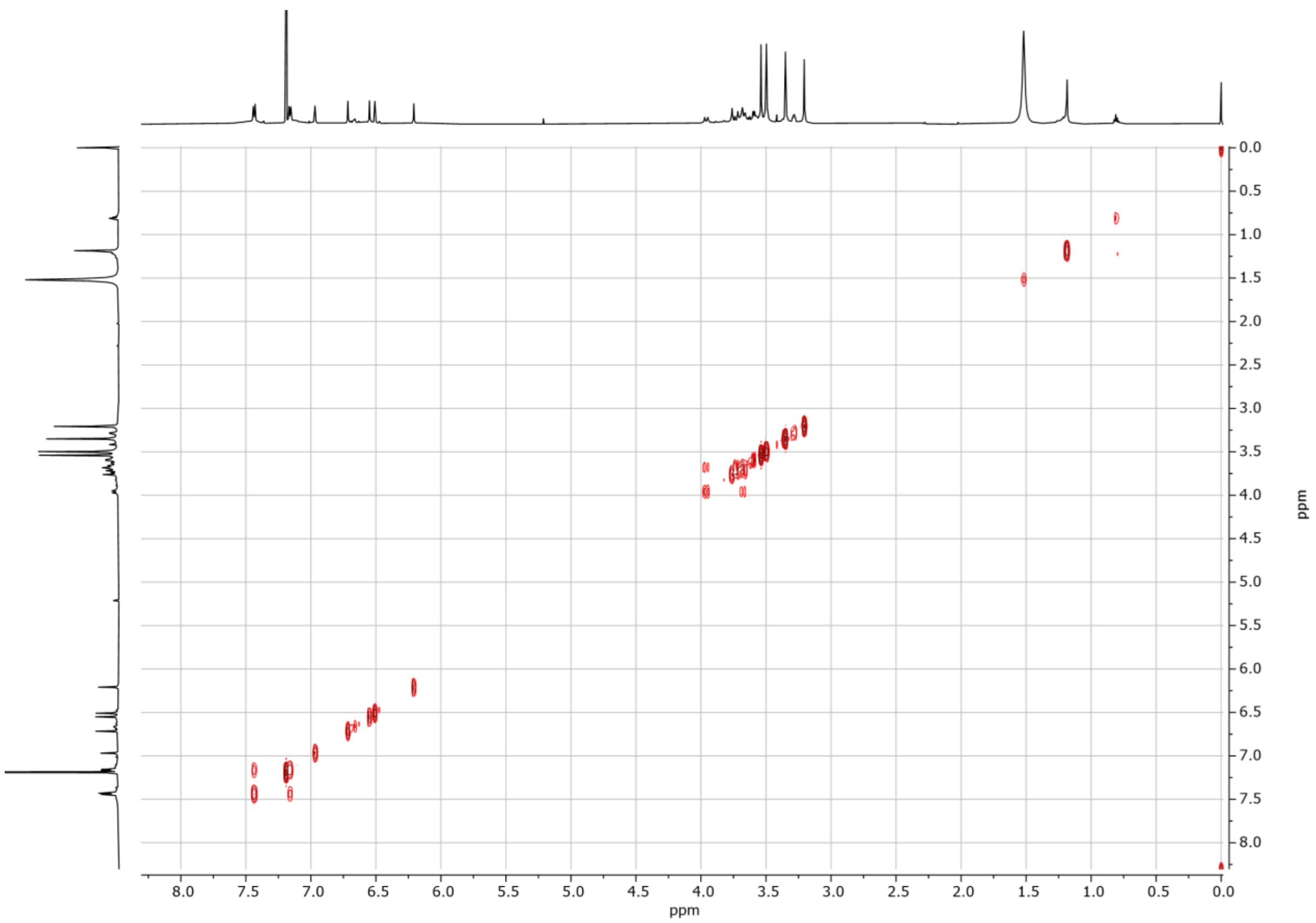

Figure S 24: $^{1}H,^{1}H$ COSY NMR spectrum of **all-p*R*/all-p*S*-[15]CPP-PA$_5$** ($CDCl_3$, 298 K, 600 MHz).

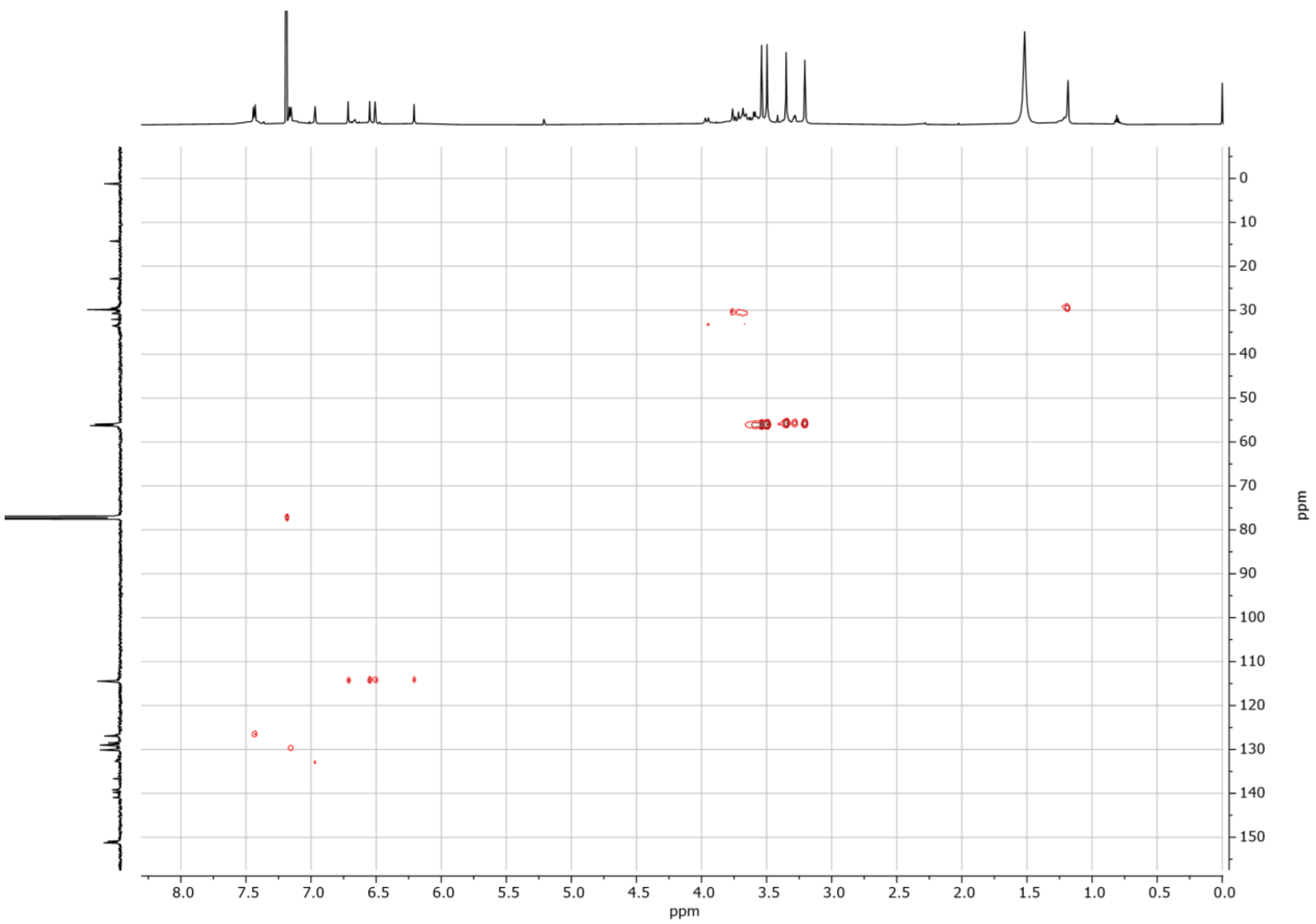

Figure S 25: $^{1}H,^{13}C$ HSQC NMR spectrum of **all-p*R*/all-p*S*-[15]CPP-PA$_5$** ($CDCl_3$, 298 K, 600 MHz, 151 MHz).

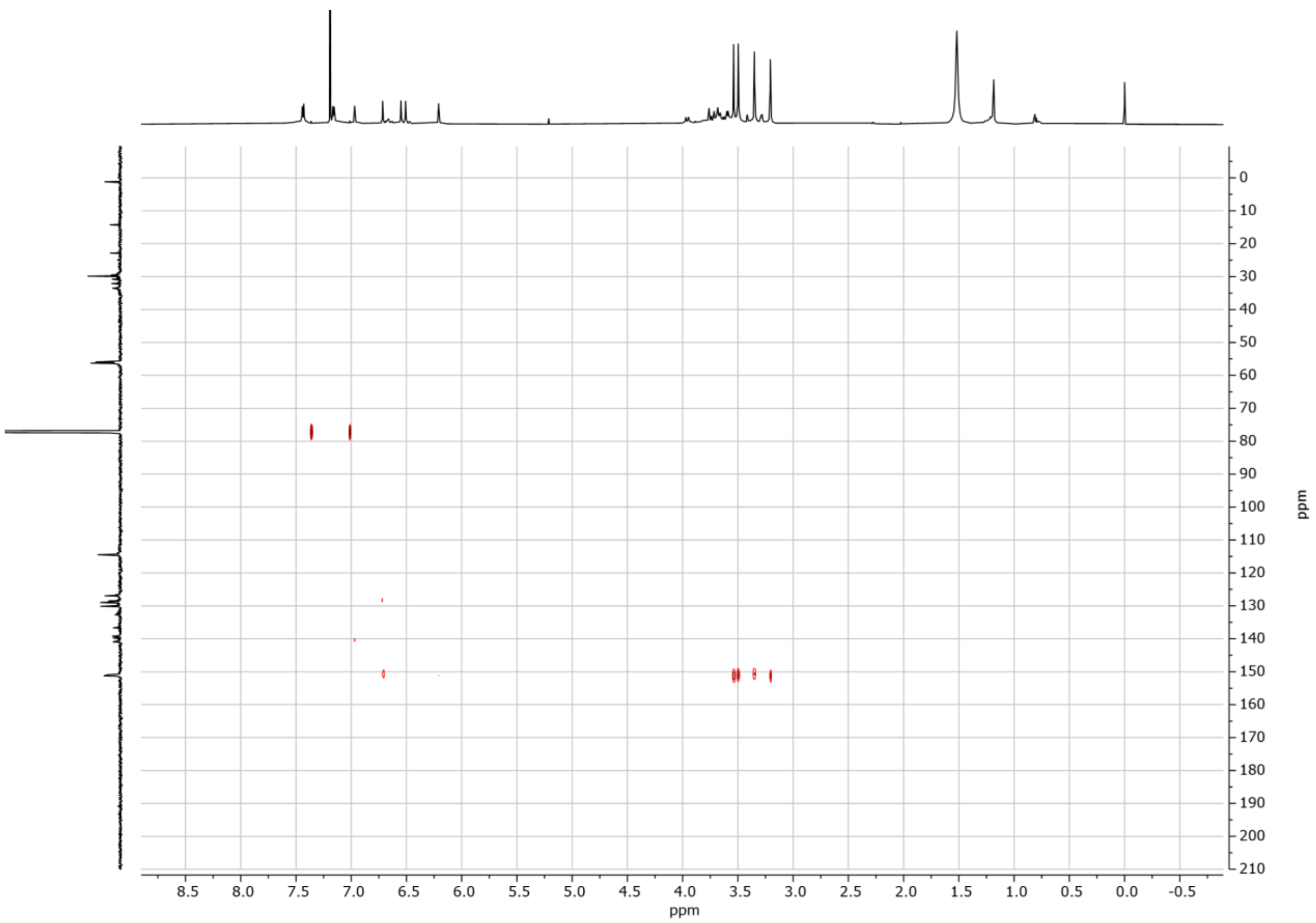

Figure S 26: $^{1}H,^{13}C$ HMBC NMR spectrum of **all-p*R*/all-p*S*-[15]CPP-PA$_5$** ($CDCl_3$, 298 K, 600 MHz, 151 MHz).

5. High-Resolution Mass Spectra

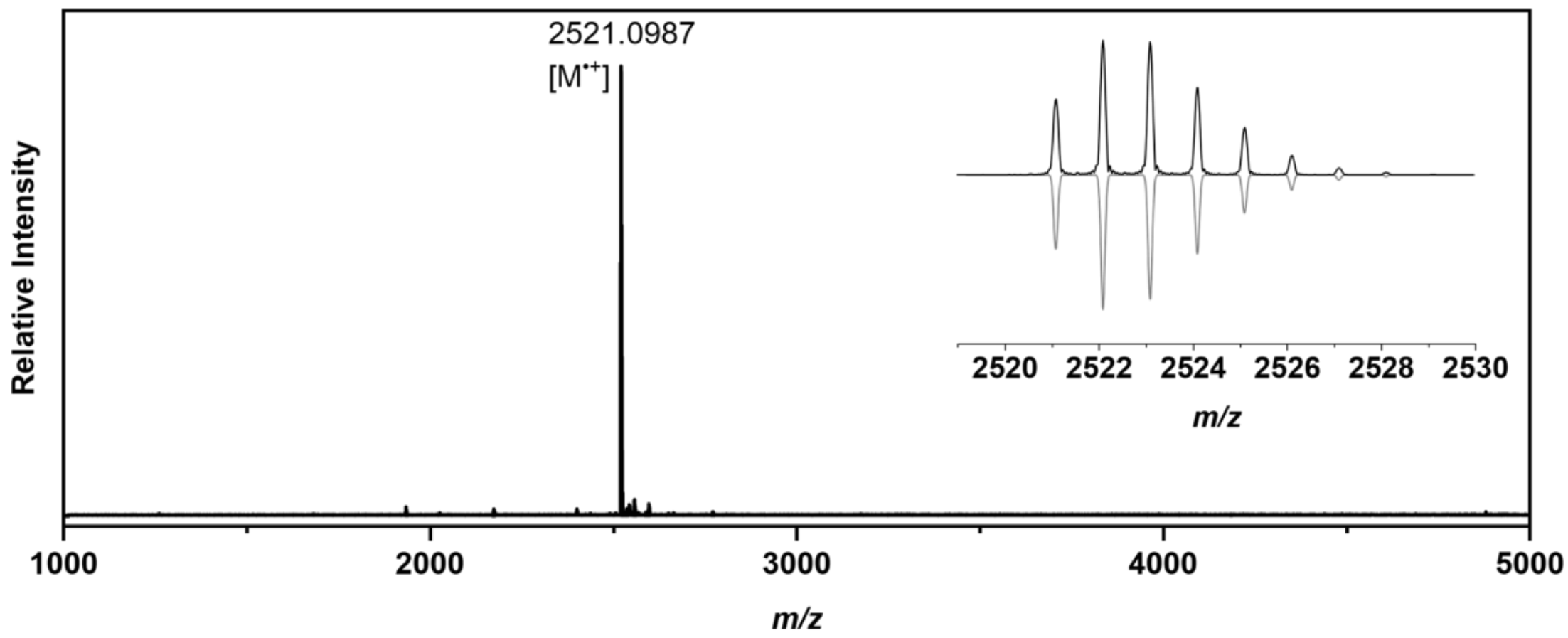

Figure S 27: HR-MALDI+ mass spectrum of **[9]CPP-PA$_3$**.

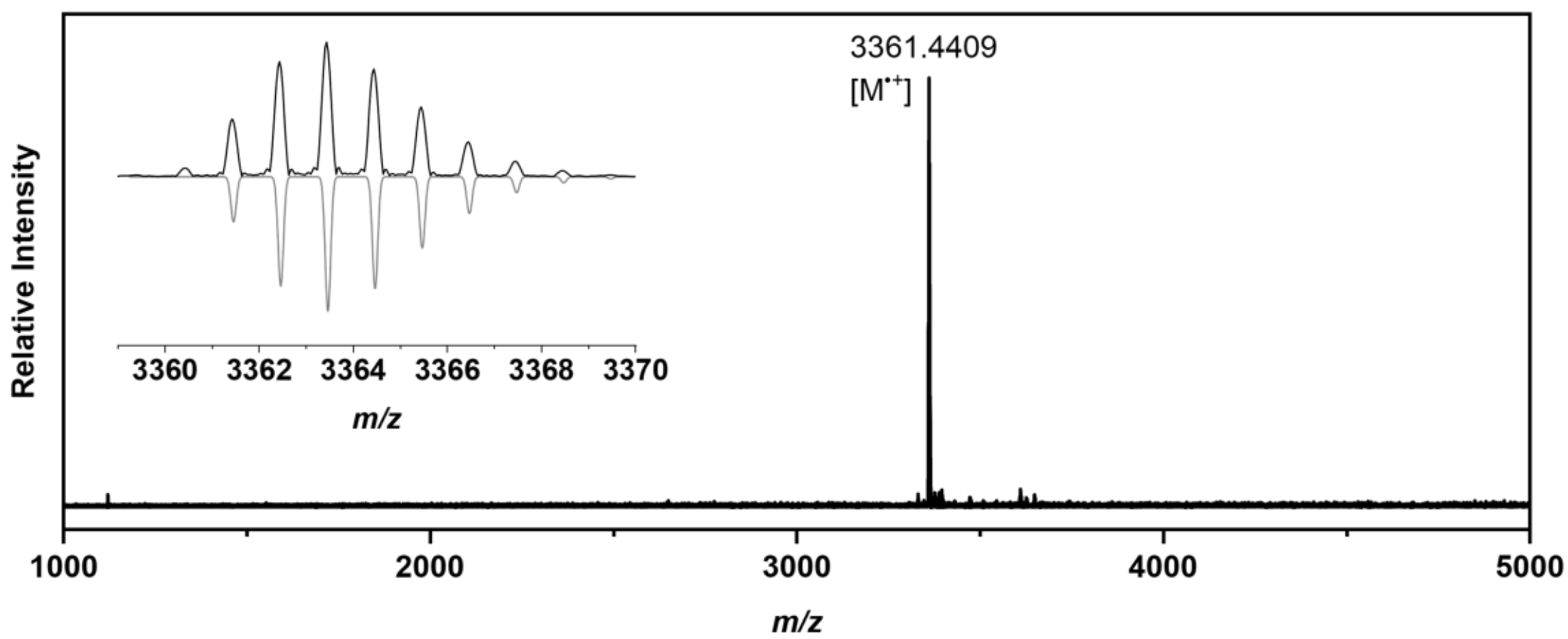

Figure S 28: HR-MALDI+ mass spectrum of **[12]CPP-PA$_4$**.

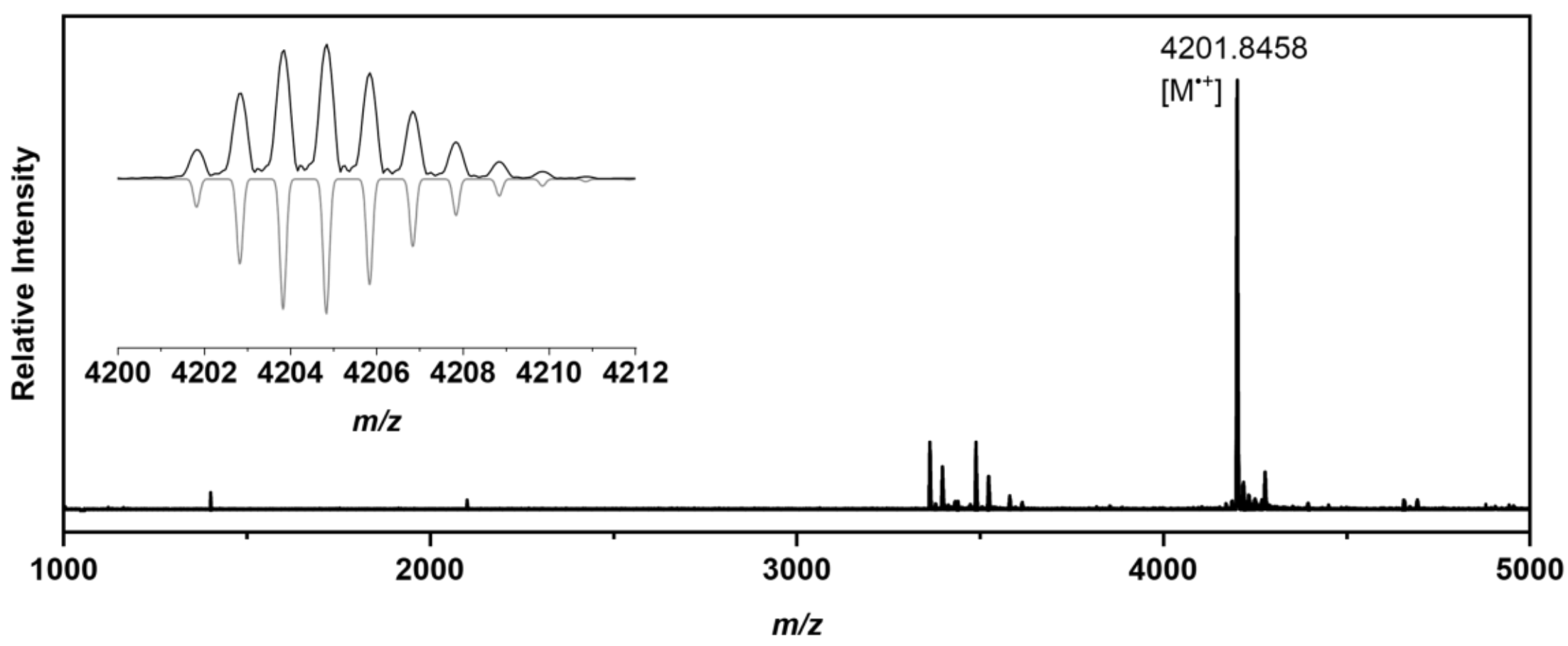

Figure S 29: HR-MALDI+ mass spectrum of **[15]CPP-PA$_5$**.

6. Single-crystal X-ray Diffraction of p*R*-3

A suitable colorless block-shaped crystal was obtained by solvent diffusion of MeOH into a $CHCl_3$ solution. X-ray diffraction data were collected on a Rigaku/Oxford SuperNova single crystal four-circle diffractometer equipped with a low temperature device, Atlas CCD detector and used Cu*K*α radiation (λ = 1.54184 Å) with a micro-focus sealed X-ray tube. Data were collected from a shock-cooled single crystal at 150(2) K.

All data were integrated using CrysAlisPro 43.112a, and a multi-scan absorption correction using SCALE3 ABSPACK was applied.[16] The structures were solved by dual methods using SHELXT 2018/2 and refined by full-matrix least-squares methods against F^2 by SHELXL-2019/3.[17,18] All nonhydrogen atoms were refined with anisotropic displacement parameters. All hydrogen atoms were refined isotropically on calculated positions using a riding model with their U_{iso} values constrained to 1.5 times the U_{eq} of their pivot atoms for terminal sp3 carbon atoms and 1.2 times for all other carbon atoms. Disordered moieties were refined using bond lengths restraints and displacement parameter restraints.

Crystallographic data for the structures reported in this paper have been deposited with the Cambridge Crystallographic Data Centre.[19] These data can be obtained free of charge from The Cambridge Crystallographic Data Centre via www.ccdc.cam.ac.uk/structures. The CIF files were generated using FinalCif (v156).[20] Mercury 2025.3.3 was used for structural representation.[21]

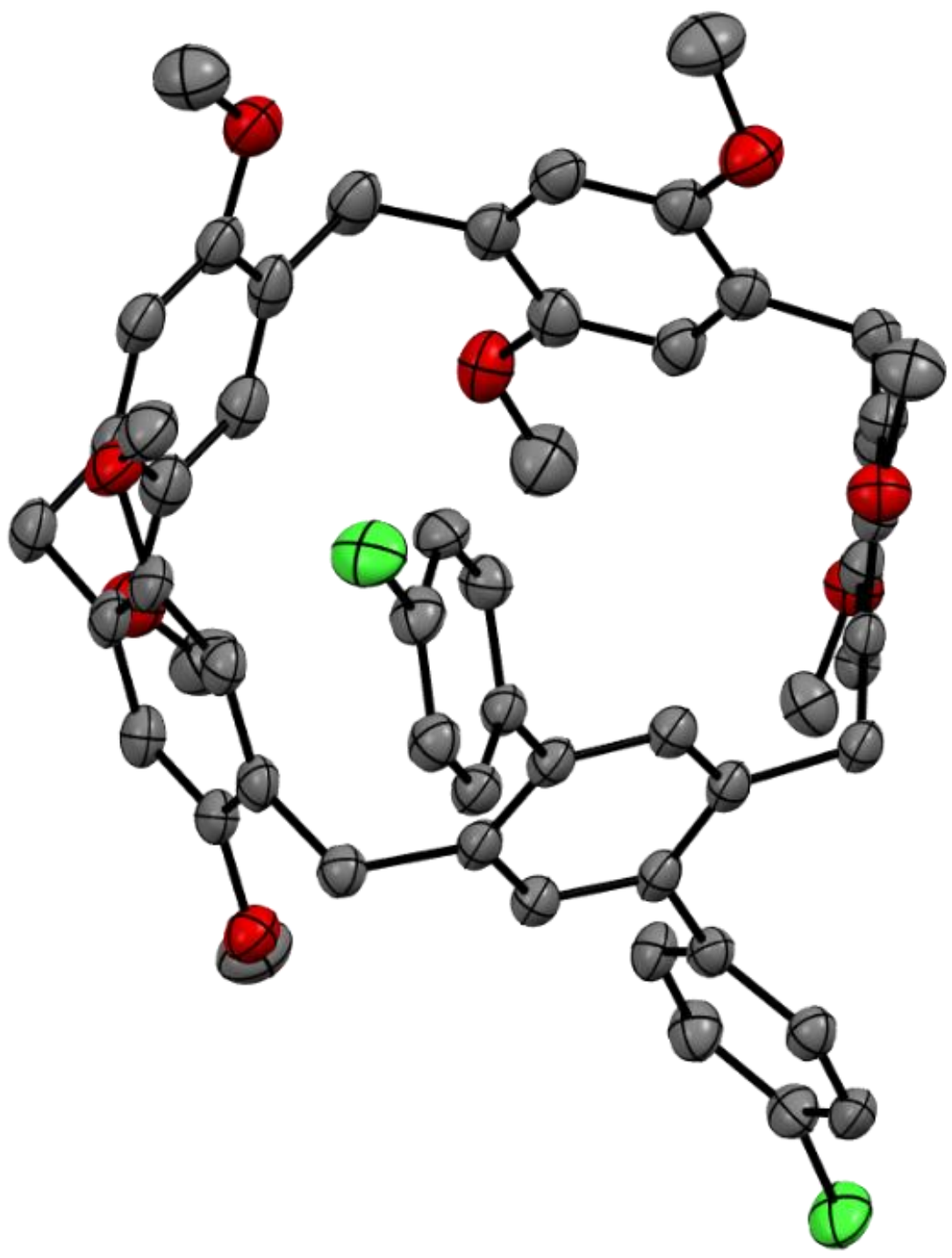

CCDC number	2546080
Empirical formula	$C_{61}H_{66}Cl_2O_8$
Formula weight	998.03
Temperature [K]	150.00(14)
Crystal system	orthorhombic
Space group (number)	$P2_12_12_1$ (19)
a [Å]	12.0933(2)
b [Å]	20.2972(5)
c [Å]	21.9221(7)
α [°]	90
β [°]	90
γ [°]	90
Volume [Å^3]	5381.0(2)
Z	4
ρ_{calc} [gcm^{-3}]	1.232
μ [mm^{-1}]	1.519
F(000)	2120
Crystal size [mm^3]	0.045×0.168×0.395
Crystal colour	clear colourless
Crystal shape	plate
Radiation	Cu K_α (λ=1.54184 Å)
2θ range [°]	8.07 to 133.20 (0.84 Å)
Index ranges	$-14 \le h \le 13$<br>$-24 \le k \le 24$<br>$-25 \le l \le 26$
Reflections collected	58490
Independent reflections	9510<br>R_{int} = 0.0889<br>R_{sigma} = 0.0521
Completeness to θ = 66.601°	99.9 %
Data / Restraints / Parameters	9510 / 248 / 703
Absorption correction T_{min}/T_{max} (method)	0.4115 / 1.0000 (multi-scan)
Goodness-of-fit on F^2	1.020
Final *R* indexes [$I \ge 2\sigma(I)$]	R_1 = 0.0434<br>wR_2 = 0.1029
Final *R* indexes [all data]	R_1 = 0.0567<br>wR_2 = 0.1129
Largest peak/hole [eÅ^{-3}]	0.24/−0.20
Flack X parameter	-0.013(12)

7. Chiral Stationary Phase Chromatograms

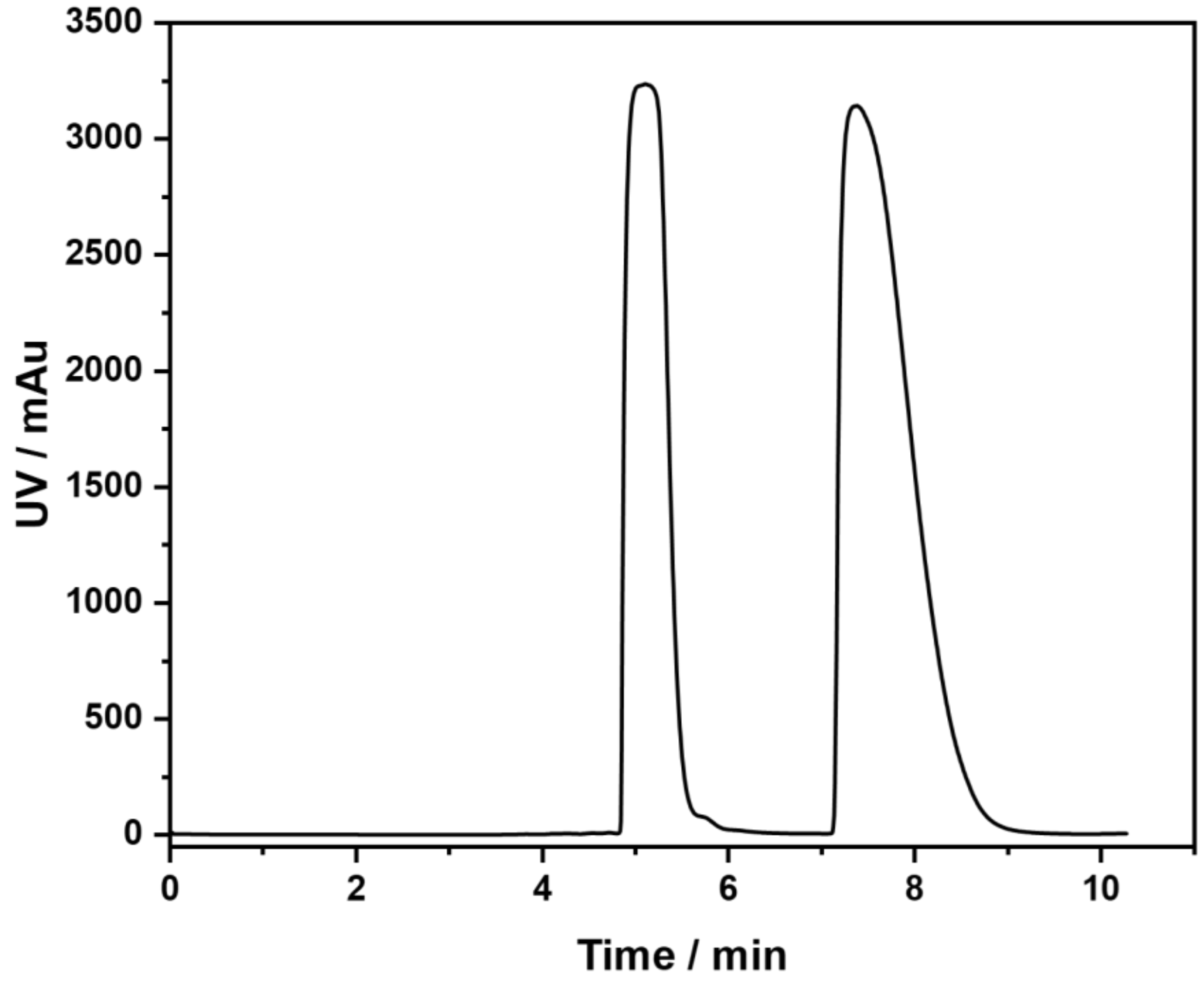

Figure S 30: HPLC chromatogram of racemic **3** (flow rate: 20 ml/min; injection volume 2 ml; solvent: CH_2Cl_2:heptane 45:55; CHIRALPAK® IK, 250 × 21 mm, 5 µm).

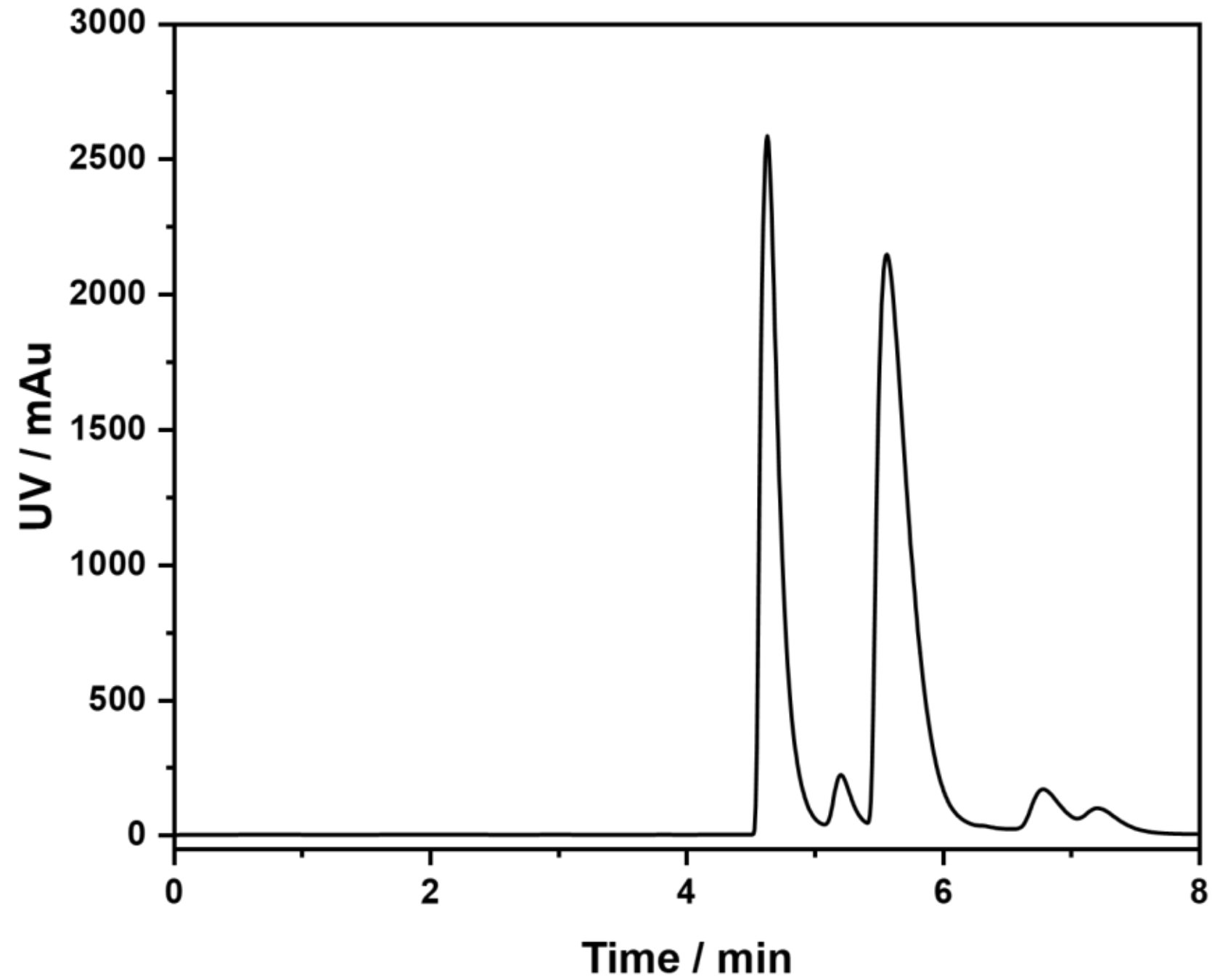

Figure S 31: HPLC chromatogram of racemic **1** (flow rate: 20 ml/min; injection volume 2 ml; solvent CH_2Cl_2:heptane 40:60; CHIRALPAK® IK, 250 × 21 mm, 5 µm).

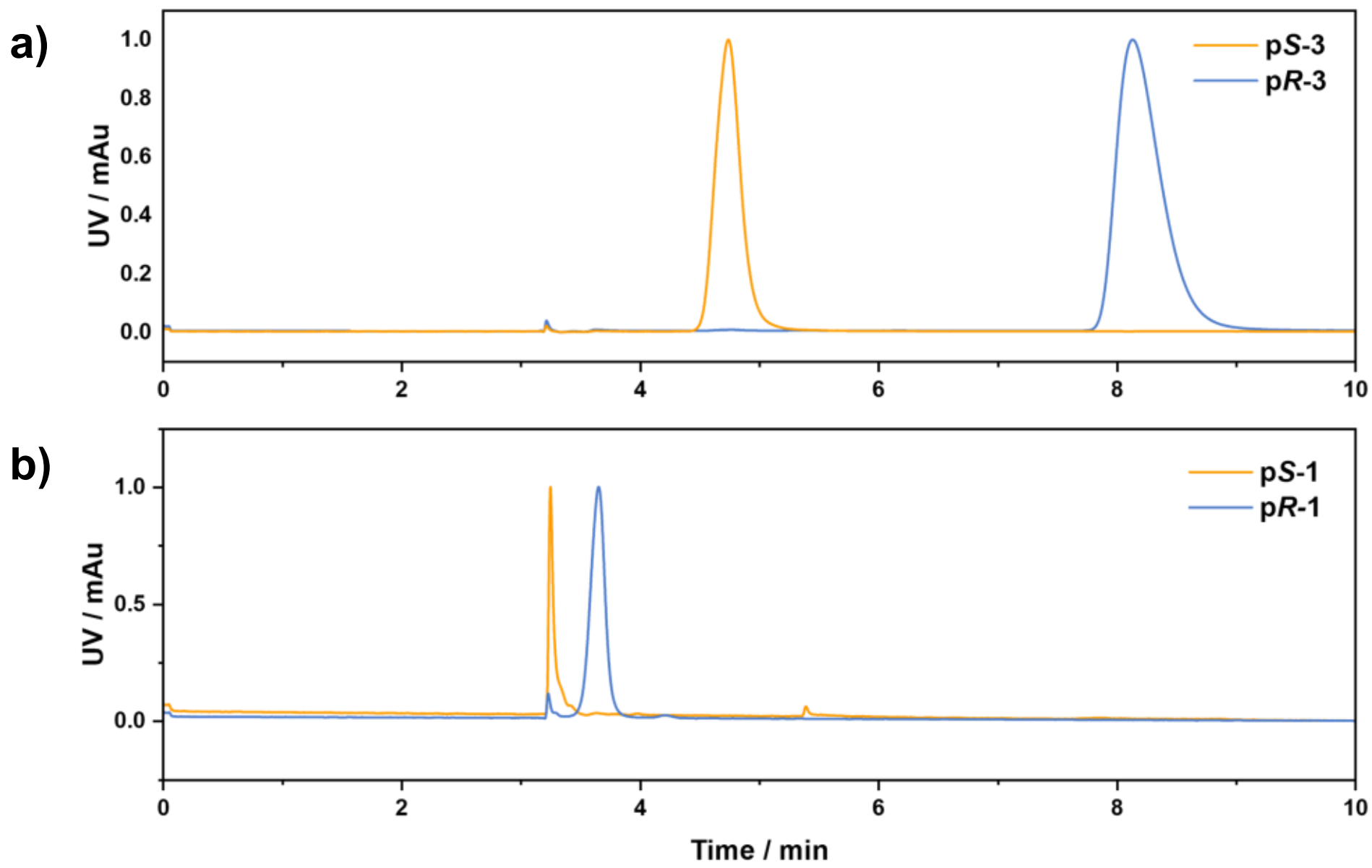

Figure S 32: Analytical HPLC chromatograms of **a) p*R*-3** and **p*S*-3**, and **b) p*R*-1** and **p*S*-1** after enantiomer separation (flow rate: 1 ml/min; solvent CH_2Cl_2:hexane 60:40; Chiralpak® IK, 250 × 4.6 mm, 5 µm).

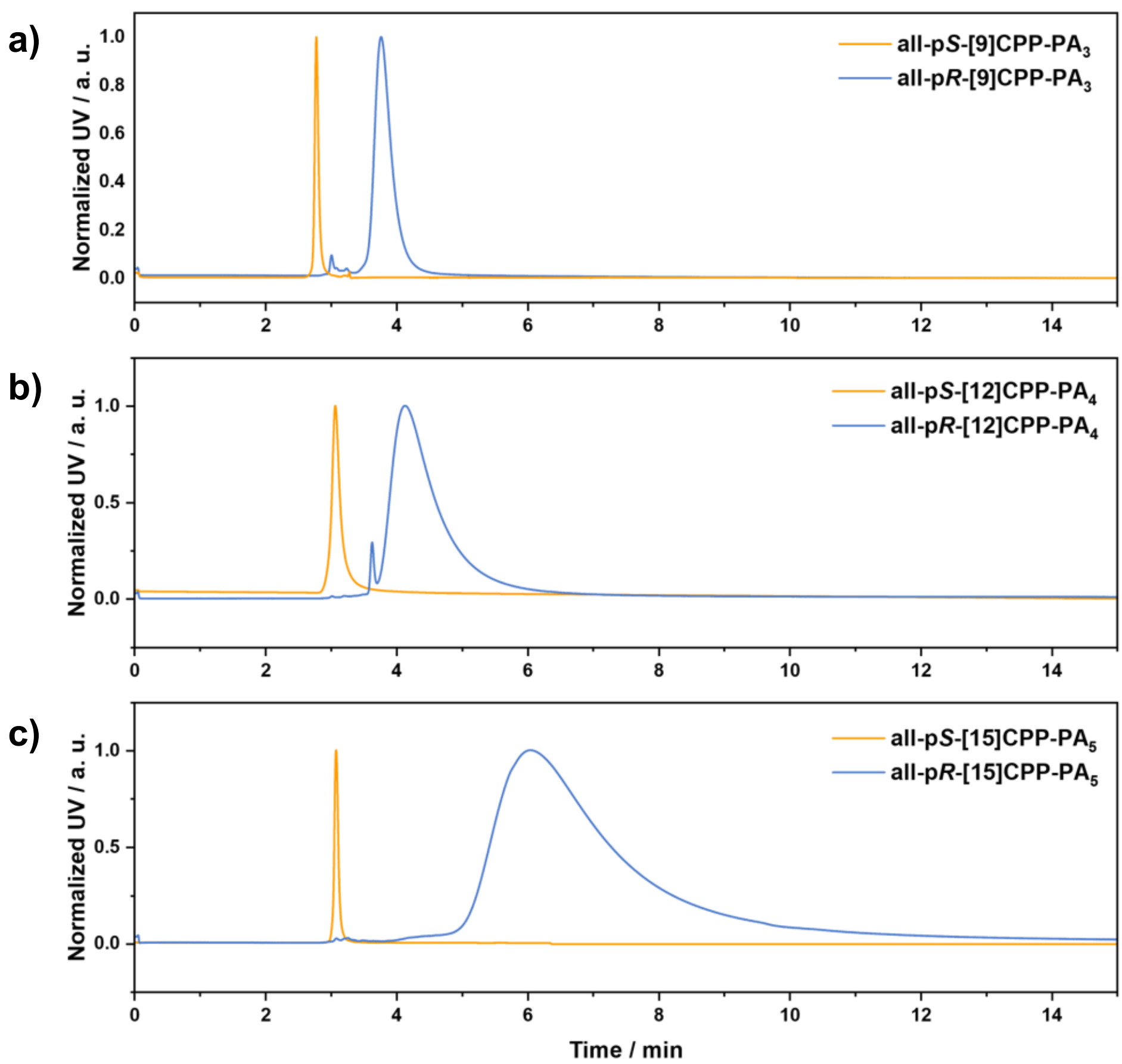

Figure S 33: Analytical HPLC chromatograms of **a) all-p*R*-** and **all-p*S*-[9]CPP-PA$_3$**, **b) all-p*R*-** and **all-p*S*-[12]CPP-PA$_4$**, and **c) all-p*R*-** and **all-p*S*-[15]CPP-PA$_5$** (flow rate: 1 ml/min; solvent CH_2Cl_2; CHIRALPAK® IK, 250 × 4.6 mm, 5 µm).

8. Analysis of the Statistical Isomer Mixture Obtained from the Cyclooligomerization of Racemic 1

8.1 [9]CPP-PA$_3$

Table S 1: List of all diastereomers of **[9]CPP-PA$_3$** with their statistical occurrence in the cyclooligomerization of racemic **1** (for chiral diastereomers the occurrence of the racemate is given) and their chirality.

Diastereomer	Statistical occurrence	Chirality
p*SSS*	1/4	Chiral
p*SSR*	3/4	Chiral

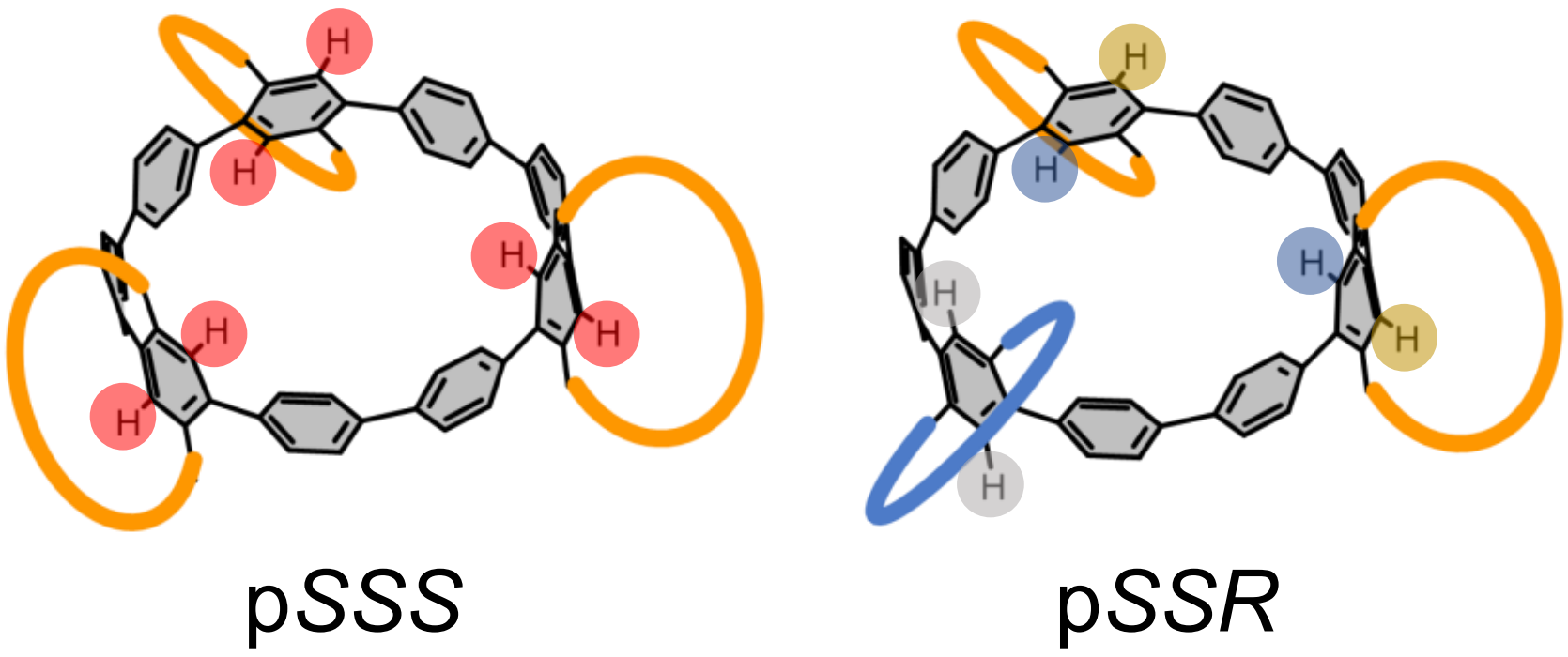

Figure S 34: Depiction of the two possible diastereomers of **[9]CPP-PA$_3$** (one enantiomer each) with schematic representation of the P[5]A rings as orange circles for p*S* configuration and blue circles for p*R* configuration. To demonstrate the chemical equivalency of the repeating units, the H atoms of the shared phenyl rings are depicted and color-coded according to their chemical equivalency.

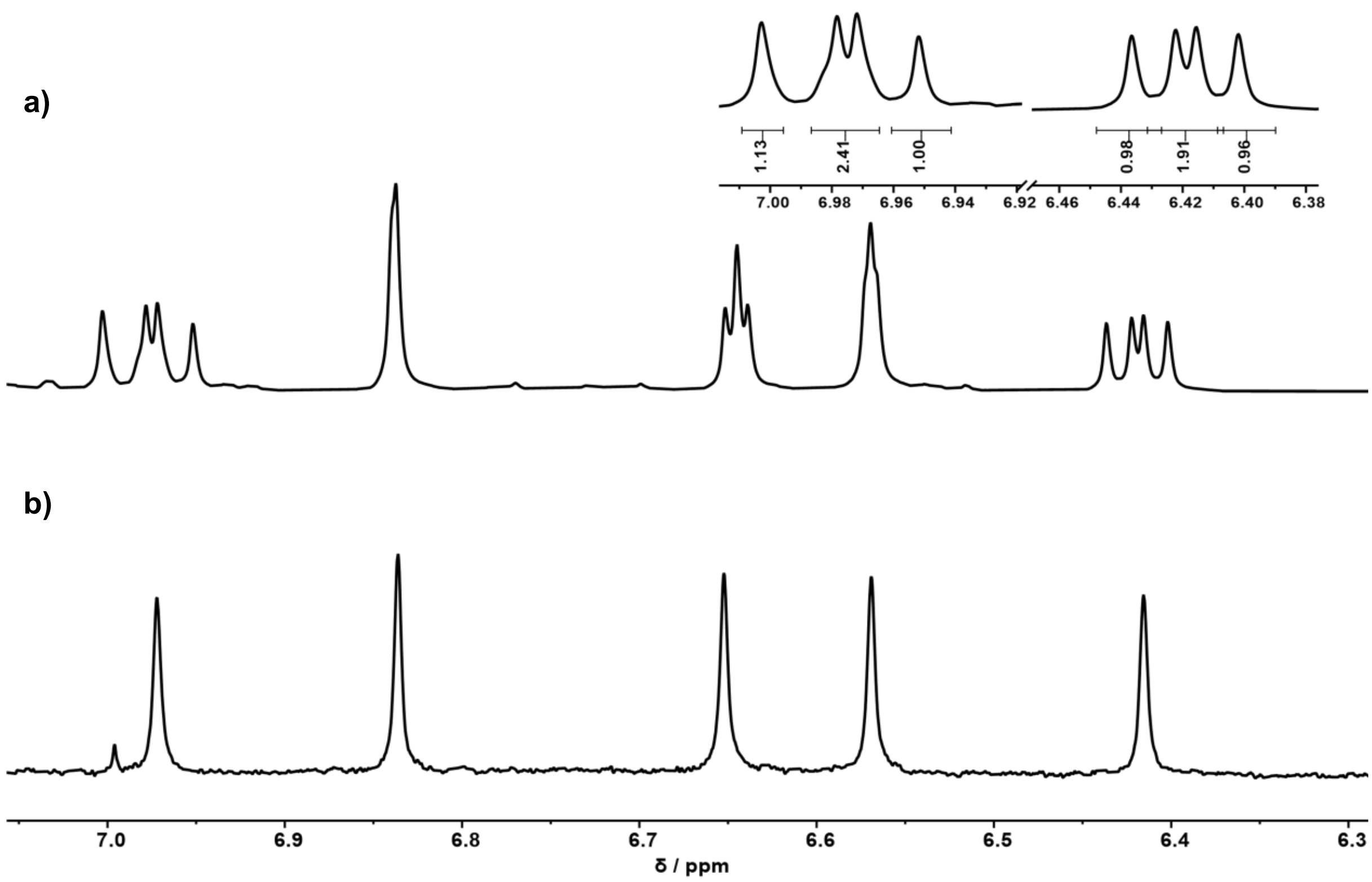

Figure S 35: Partial ^{1}H NMR spectrum ($CDCl_3$, 298 K, 600 MHz) of **a)** the stereoisomer mixture of **[9]CPP-PA$_3$** obtained from racemic **1**; and **b)** single diastereomer **all-p*R*/all-p*S*-[9]CPP-PA$_3$**. The integral ratio of 1:1:1:1 of the four singlets in the isomer mixture matching a single singlet in **all-p*R*/all-p*S*-[9]CPP-PA$_3$** confirms the statistical distribution in the stereoisomeric mixture in a).

In a statistical stereoisomer mixture of **[9]CPP-PA$_3$**, each set of protons shown in figure S34 and labelled with a different color would give a singlet in the ^{1}H NMR spectrum, resulting in four singlets. Multiplying the number of chemically equivalent protons with the statistical occurrence of the respective diastereomer reveals an expected integral ratio of 1:1:1:1. This ratio can be found in the measured ^{1}H NMR spectrum (figure S35) of the isomer mixture, confirming a statistical distribution.

8.2 [12]CPP-PA$_4$

Table S 2: List of all diastereomers of **[12]CPP-PA$_4$** with their statistical occurrence in the cyclooligomerization of racemic **1** (for chiral diastereomers the occurrence of the racemate is given) and their chirality.

Diastereomer	Statistical occurrence	Chirality
p*SSSS*	1/8	Chiral
p*SSSR*	1/2	Chiral
p*SSRR*	1/4	Achiral
p*SRSR*	1/8	Achiral

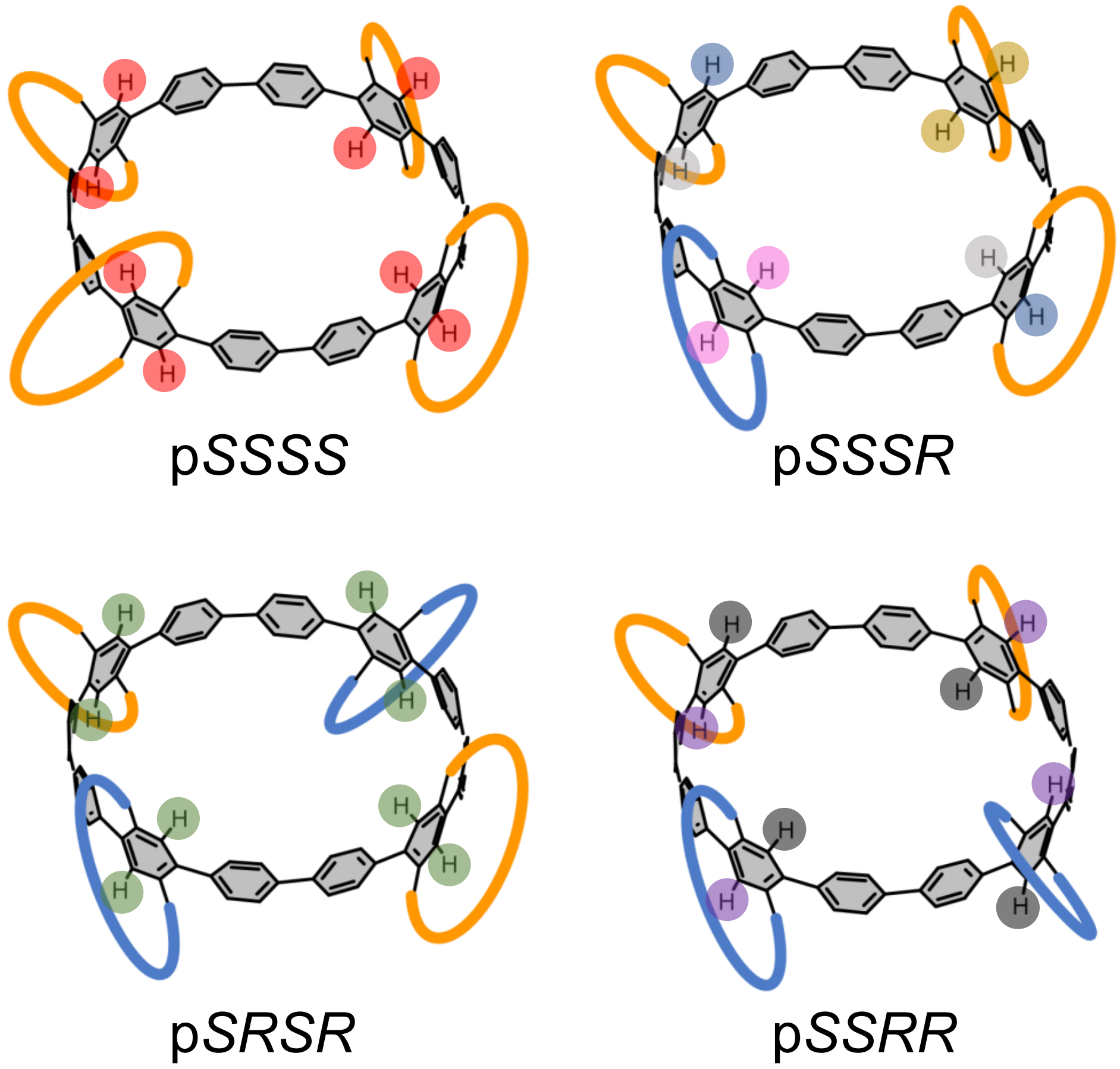

Figure S 36: Depiction of the four possible diastereomers of **[12]CPP-PA$_4$** (one enantiomer for each chiral diastereomer) with schematic representation of the P[5]A rings as orange circles for p*S* configuration and blue circles for p*R* configuration. To demonstrate the chemical equivalency of the repeating units, the H atoms of the shared phenyl rings are depicted and color-coded according to their chemical equivalency.

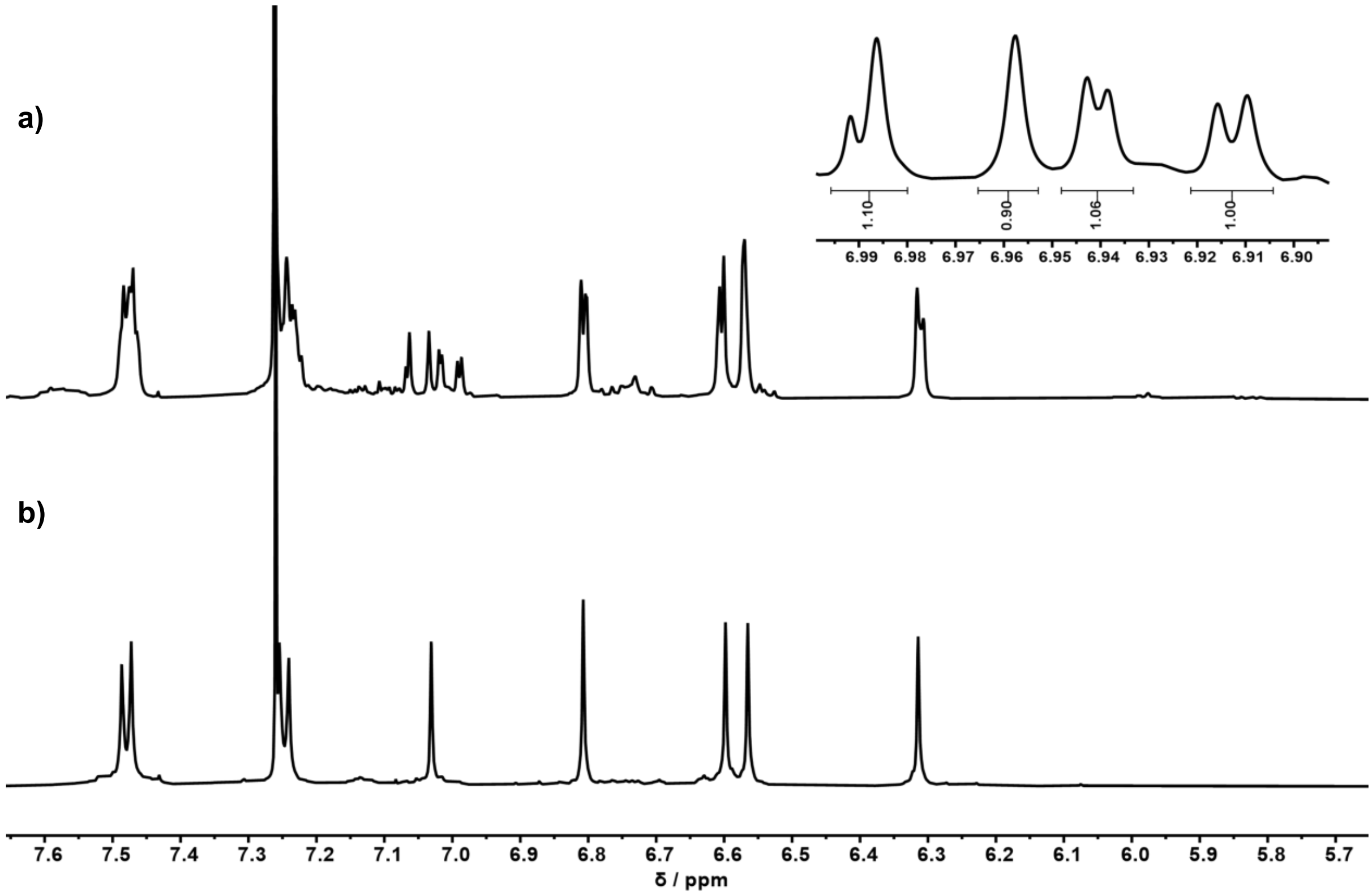

Figure S 37: Partial ^{1}H NMR spectrum ($CDCl_3$, 298 K, 600 MHz) of **a)** the stereoisomer mixture of **[12]CPP-PA$_4$** obtained from racemic **1**; and **b)** single diastereomer **all-p*R*/all-p*S*-[12]CPP-PA$_4$**. The integral ratio of 1:1:1:1:1:1:1:1 of the eight singlets in the stereoisomer mixture matching a single singlet in **all-p*R*/all-p*S*-[12]CPP-PA$_4$** confirms the statistical distribution of the stereoisomeric mixture.

In a statistical stereoisomer mixture of **[12]CPP-PA$_4$**, each set of protons shown in figure S36 would give a singlet in the ^{1}H NMR spectrum, resulting in eight singlets. Multiplying the number of chemically equivalent protons with the statistical occurrence of the respective diastereomer reveals an expected integral ratio of 1:1:1:1:1:1:1:1. This ratio can be found in the measured ^{1}H NMR spectrum (figure S27) of the isomer mixture, confirming a statistical distribution.

8.3 [15]CPP-PA$_5$

Table S 3: List of all diastereomers of **[15]CPP-PA$_5$** with their statistical occurrence in the cyclooligomerization of racemic **1** (for chiral diastereomers the occurrence of the racemate is given) and their chirality.

Diastereomer	Statistical occurrence	Chirality
p*SSSSS*	1/16	Chiral
p*SSSSR*	5/16	Chiral
p*SSSRR*	5/16	Chiral
p*SSRSR*	5/16	Chiral

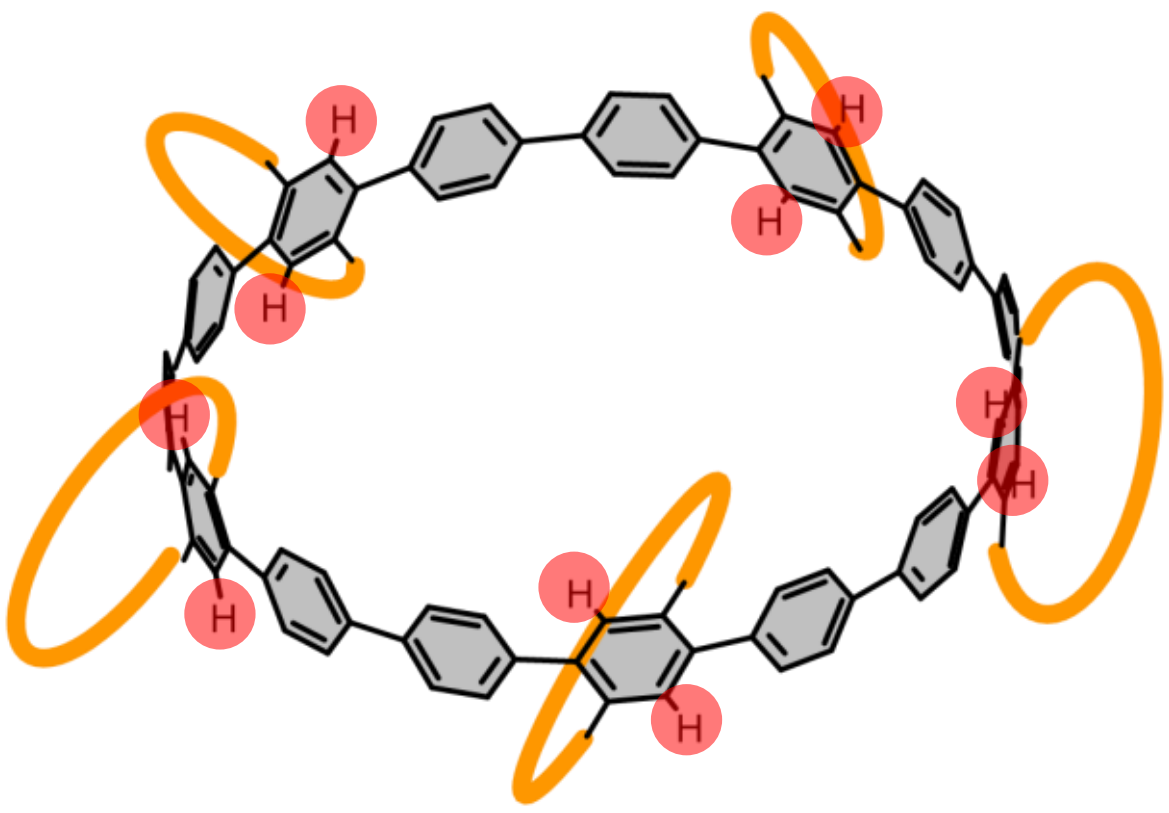

p*SSSSS*

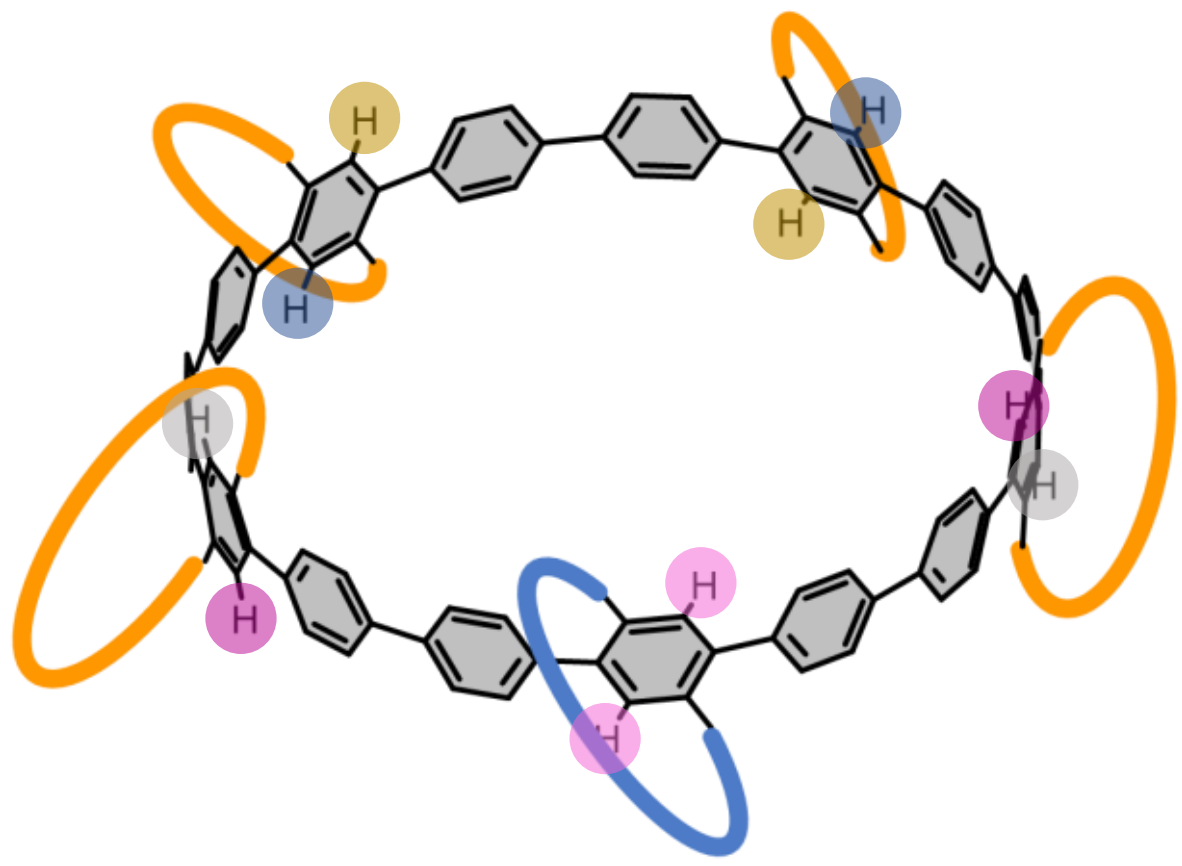

p*SSSSR*

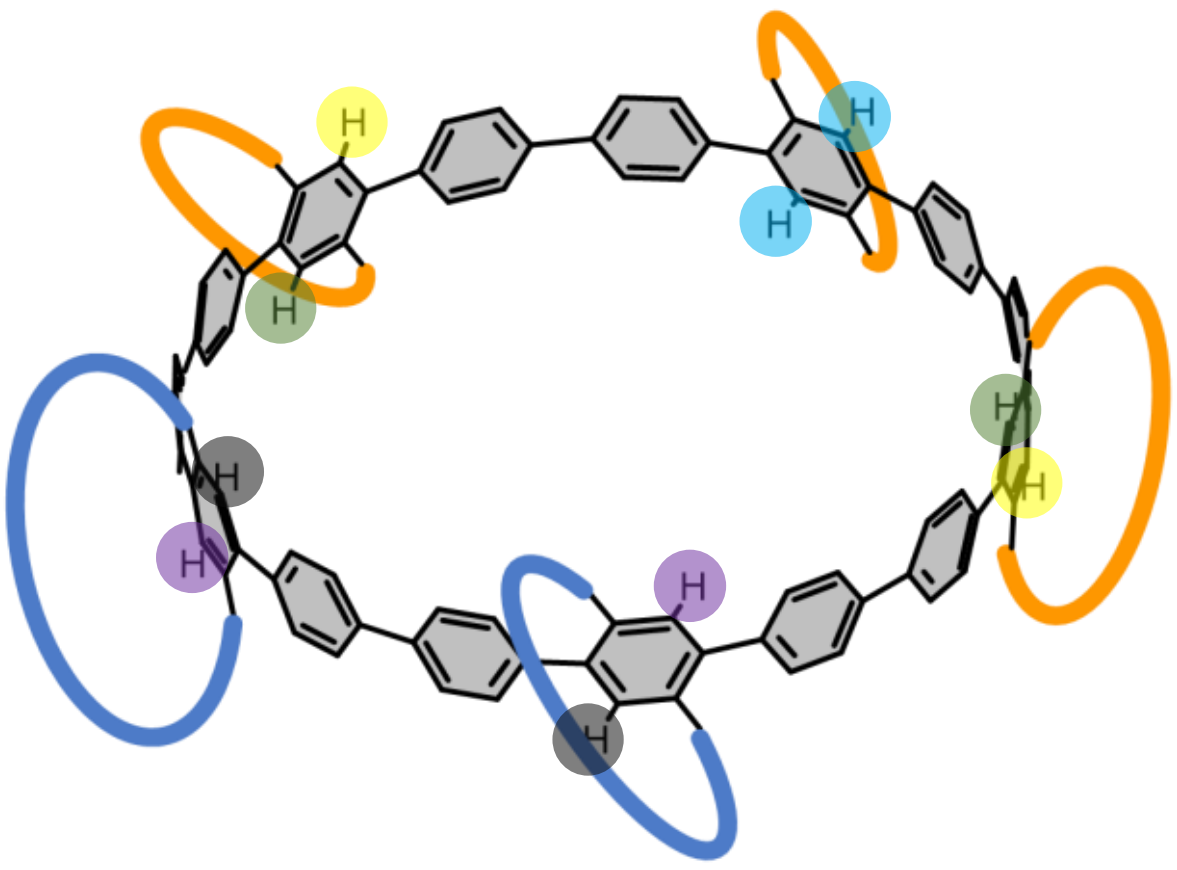

p*SSSRR*

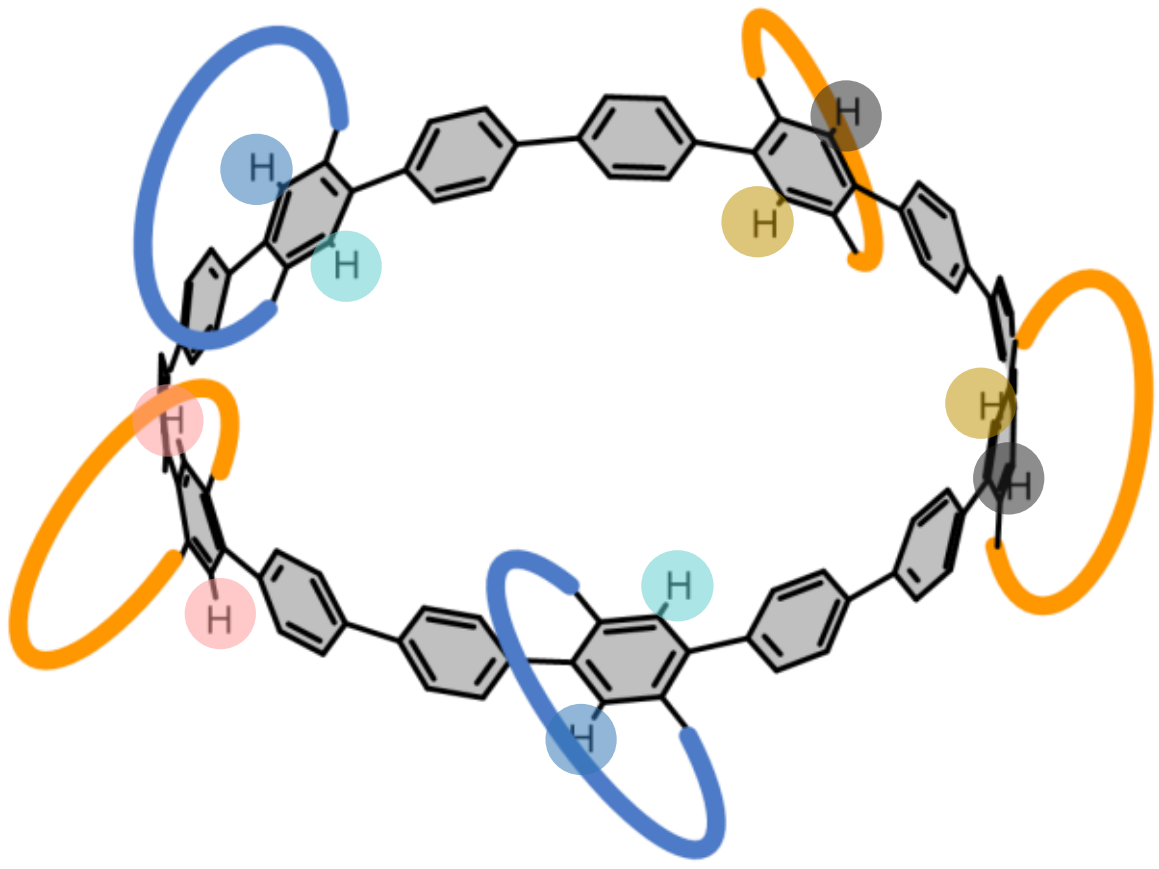

p*SSRSR*

Figure S 38: Depiction of the four possible diastereomers of **[15]CPP-PA$_5$** (one enantiomer each) with schematic representation of the P[5]A rings as orange circles for p*S* configuration and blue circles for p*R* configuration. To demonstrate the chemical equivalency of the repeating units, the H atoms of the shared phenyl rings are depicted and color-coded according to their chemical equivalency.

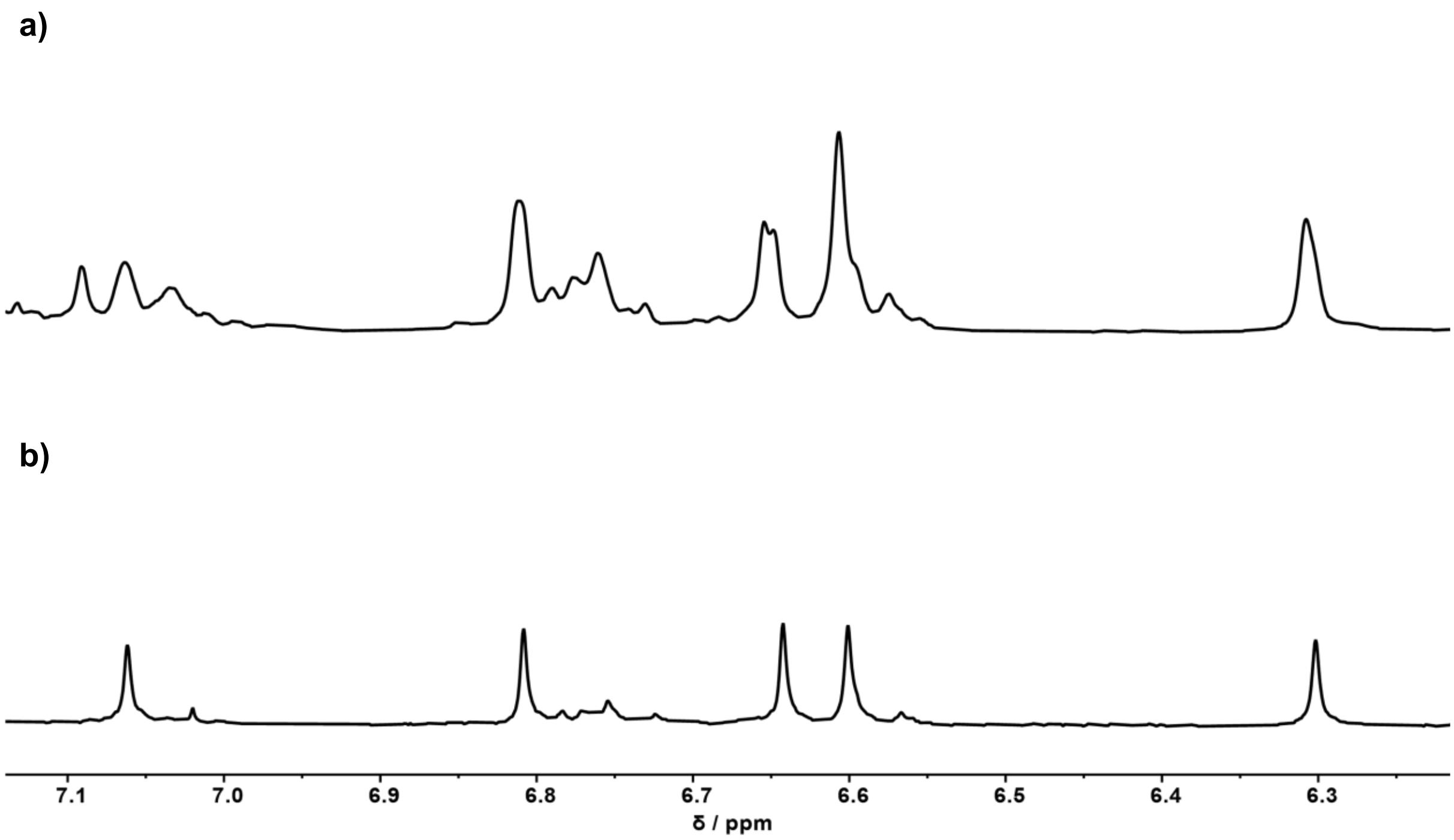

Figure S 39: Partial ^{1}H NMR spectrum ($CDCl_3$, 298 K, 600 MHz) of **a)** the stereoisomer mixture of **[15]CPP-PA$_5$** obtained from racemic **1**; and **b)** single diastereomer **all-p*R*/all-p*S*-[15]CPP-PA$_5$**. The large number of singlets in the stereoisomer mixture matching a single singlet in **all-p*R*/all-p*S*-[15]CPP-PA$_5$** prevents integration of the signals.

In a statistical stereoisomer mixture of **[15]CPP-PA$_5$**, each set of protons shown in figure S38 would give a singlet in the ^{1}H NMR spectrum, resulting in 16 singlets. Multiplying the number of chemically equivalent protons with the statistical occurrence of the respective diastereomer reveals an expected integral ratio of 1:1:1:1:1:1:1:1:1:1:1:1:1:1:1:1. This ratio cannot be confirmed in the measured ^{1}H NMR spectrum (figure S39) of the isomer mixture, since the 16 signals are much too close for individual integration. We assume statistical distribution as observed in the two smaller ring sizes.

9. Fluorescence Lifetime Measurements

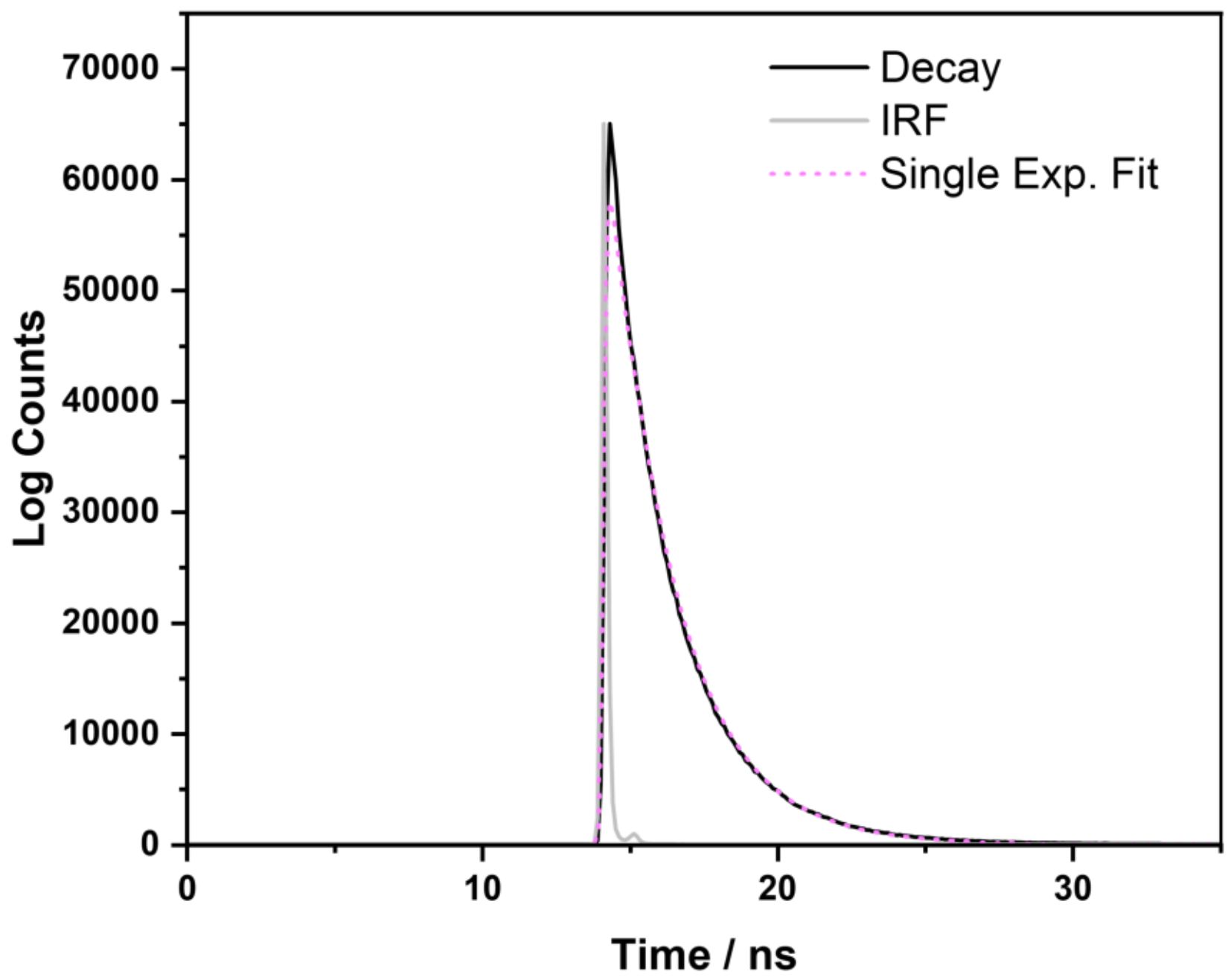

Figure S 40: Emission decay (TCSPC) of **all-p*R*/all-p*S*-[9]CPP-PA$_3$** in CH_2Cl_2 under ambient atmosphere.

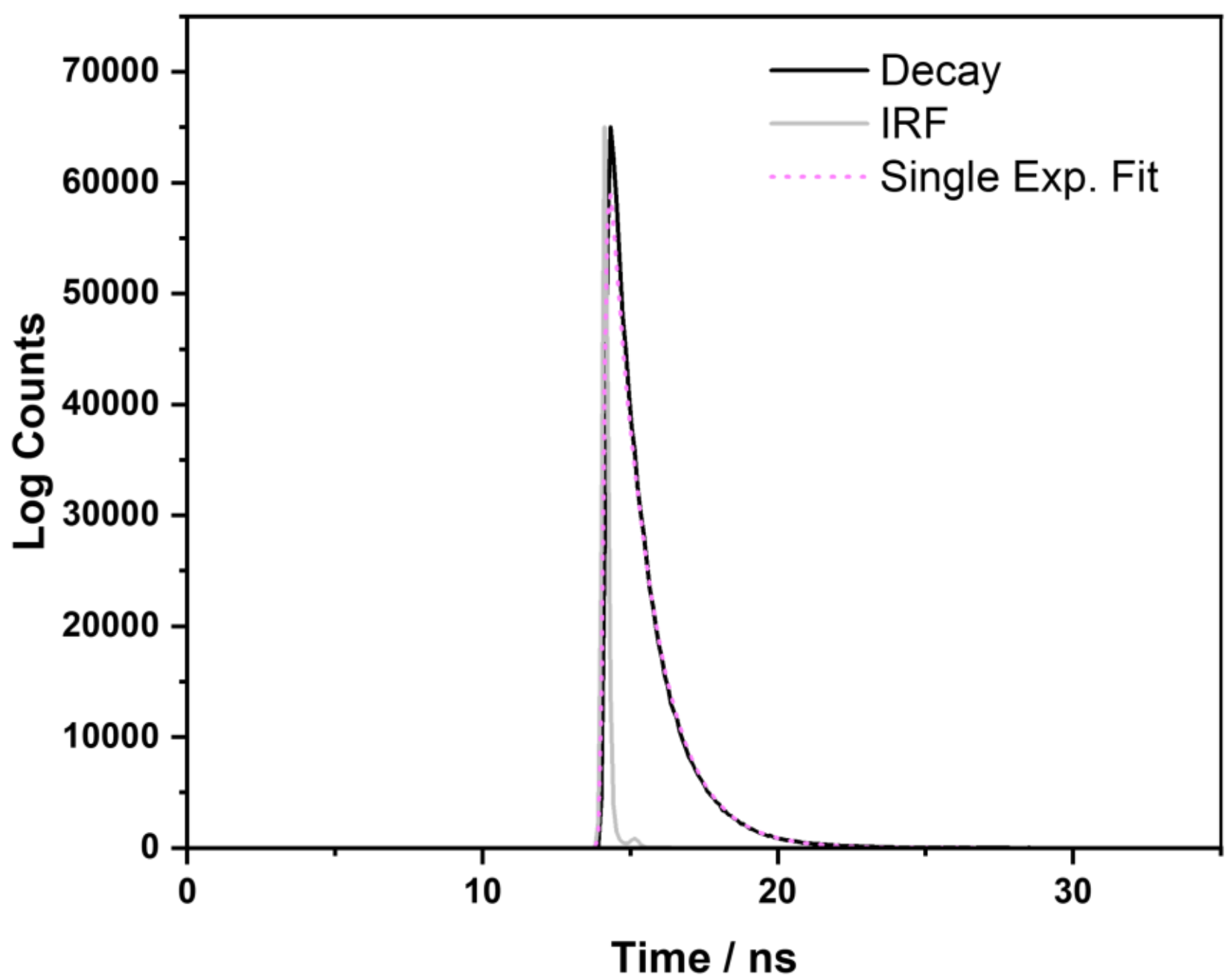

Figure S 41: Emission decay (TCSPC) of **all-p*R*/all-p*S*-[12]CPP-PA$_4$** in CH_2Cl_2 under ambient atmosphere.

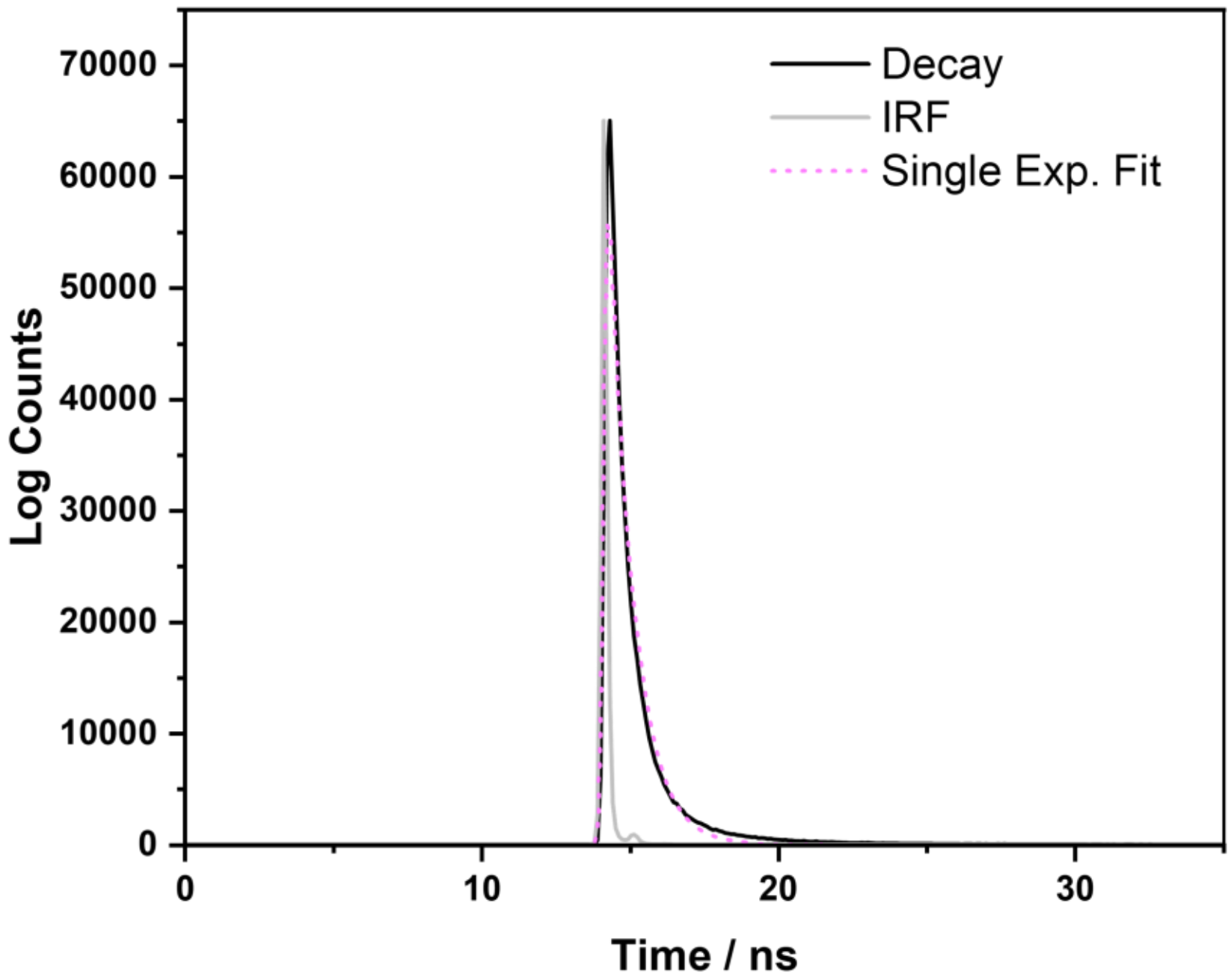

Figure S 42: Emission decay (TCSPC) of **all-p*R*/all-p*S*-[15]CPP-PA₅** in CH_2Cl_2 under ambient atmosphere.

10. Electronic Circular Dichroism Spectra

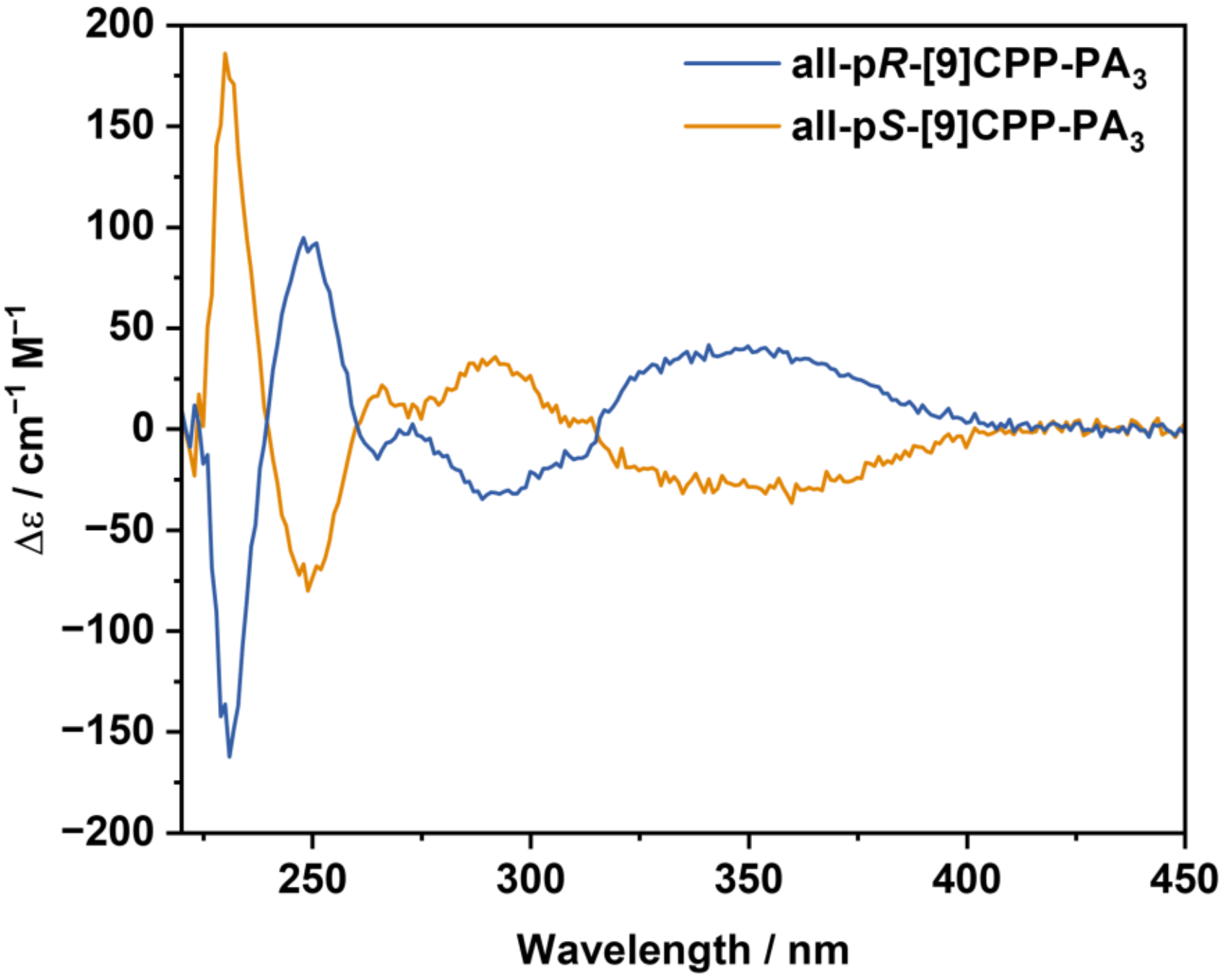

Figure S 43: ECD spectra of **all-p*R*-** and **all-p*S*-[9]CPP-PA₃** in CH_2Cl_2 (10^{-5} to 10^{-6} M).

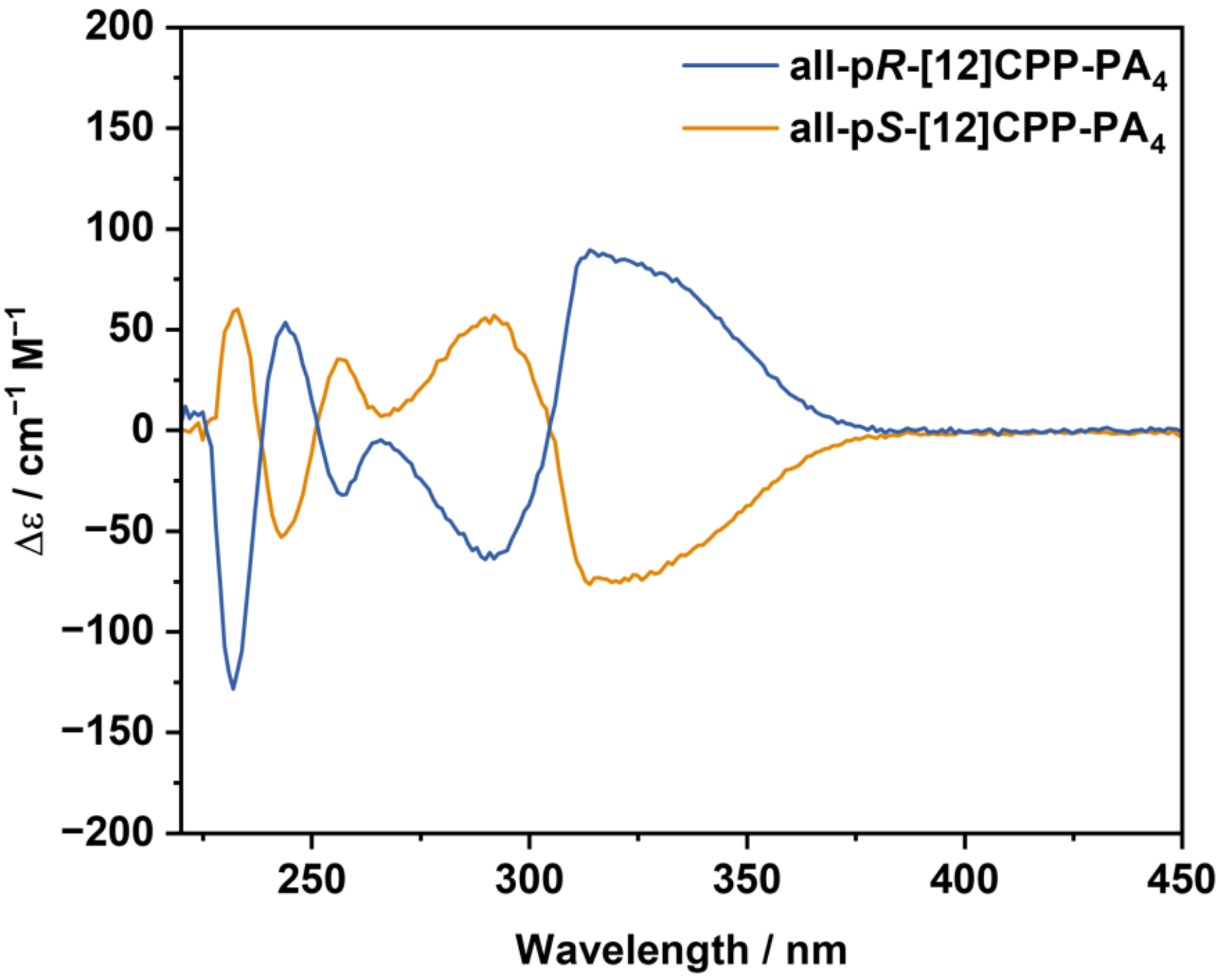

Figure S 44: ECD spectra of **all-p*R*-** and **all-p*S*-[12]CPP-PA₄** in CH_2Cl_2 (10^{-5} to 10^{-6} M).

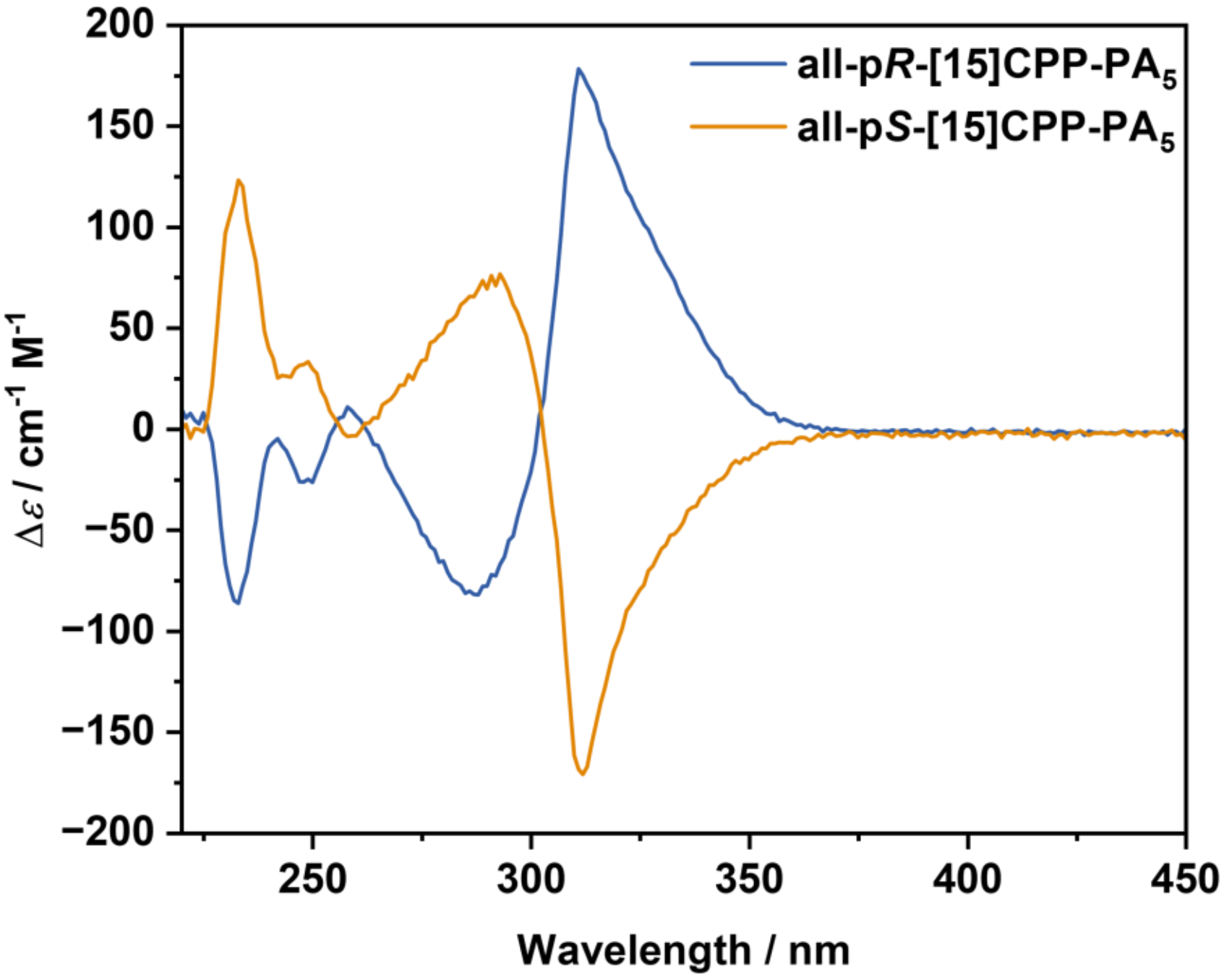

Figure S 45: ECD spectra of **all-p*R*-** and **all-p*S*-[15]CPP-PA₅** in CH_2Cl_2 (10^{-5} to 10^{-6} M).

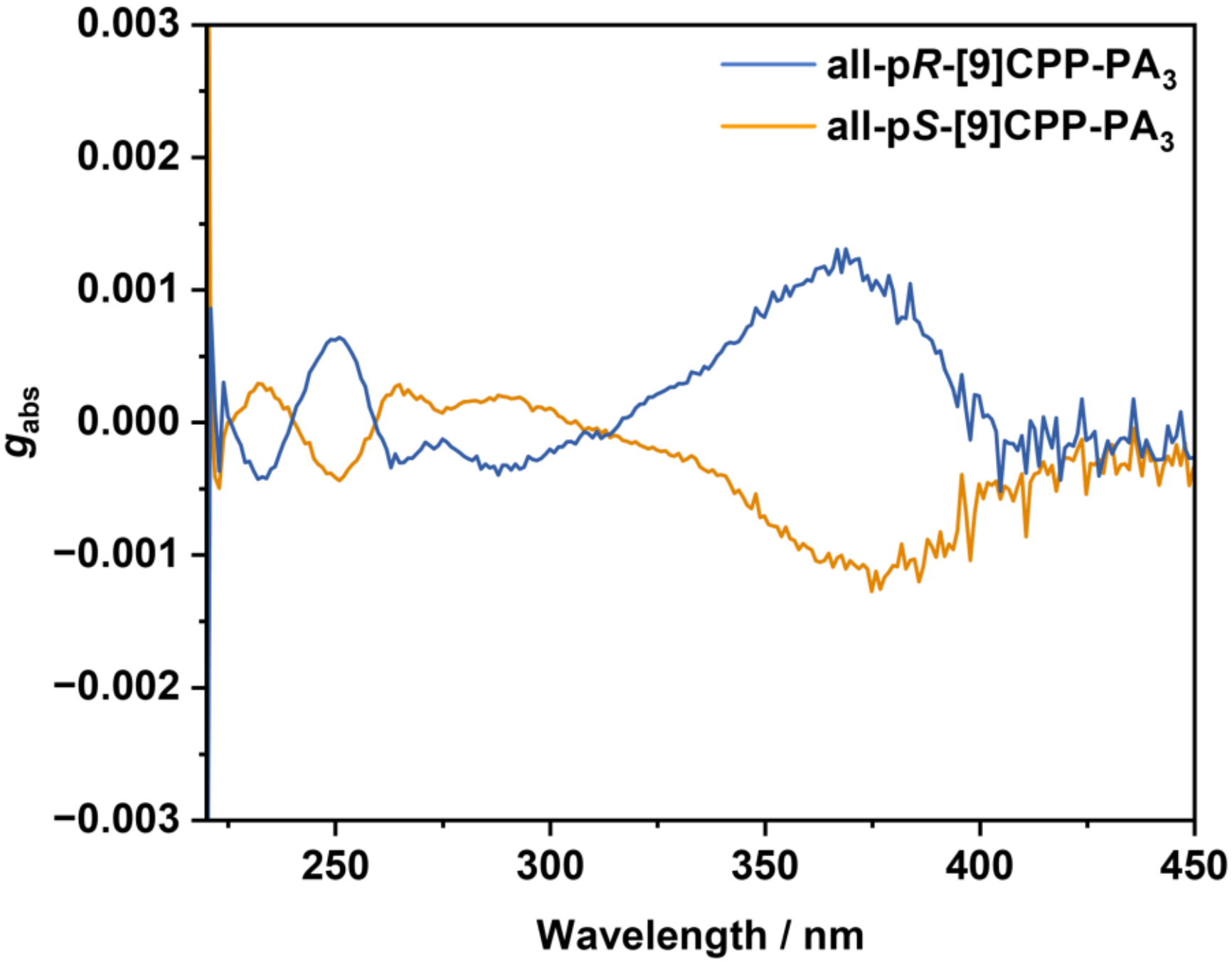

Figure S 46: Plot of g_{abs} of **all-p*R*-** and **all-p*S*-[9]CPP-PA₃** in CH_2Cl_2 (10^{-5} to 10^{-6} M).

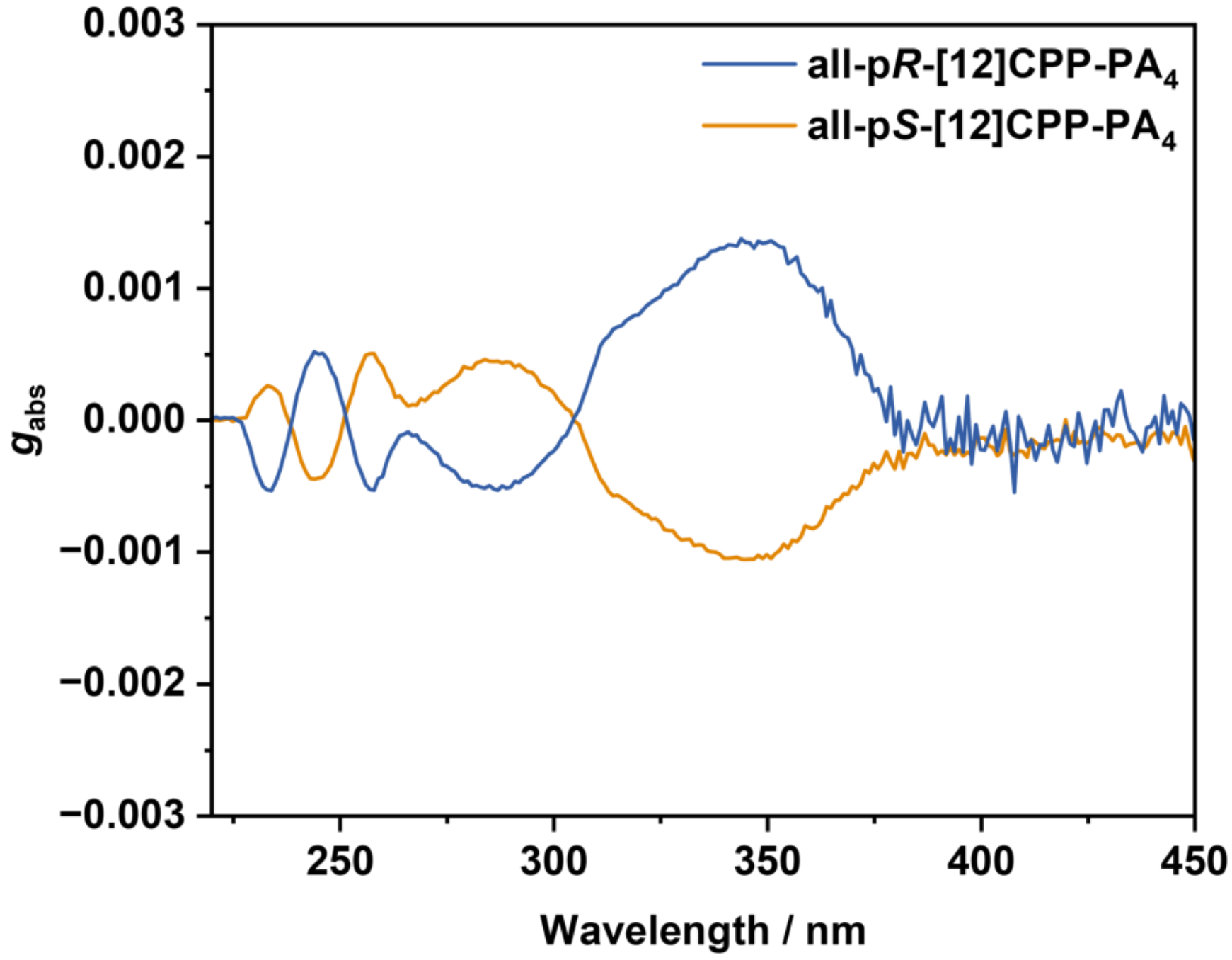

Figure S 47: Plot of g_{abs} of **all-p*R*-** and **all-p*S*-[12]CPP-PA$_4$** in CH_2Cl_2 (10^{-5} to 10^{-6} M).

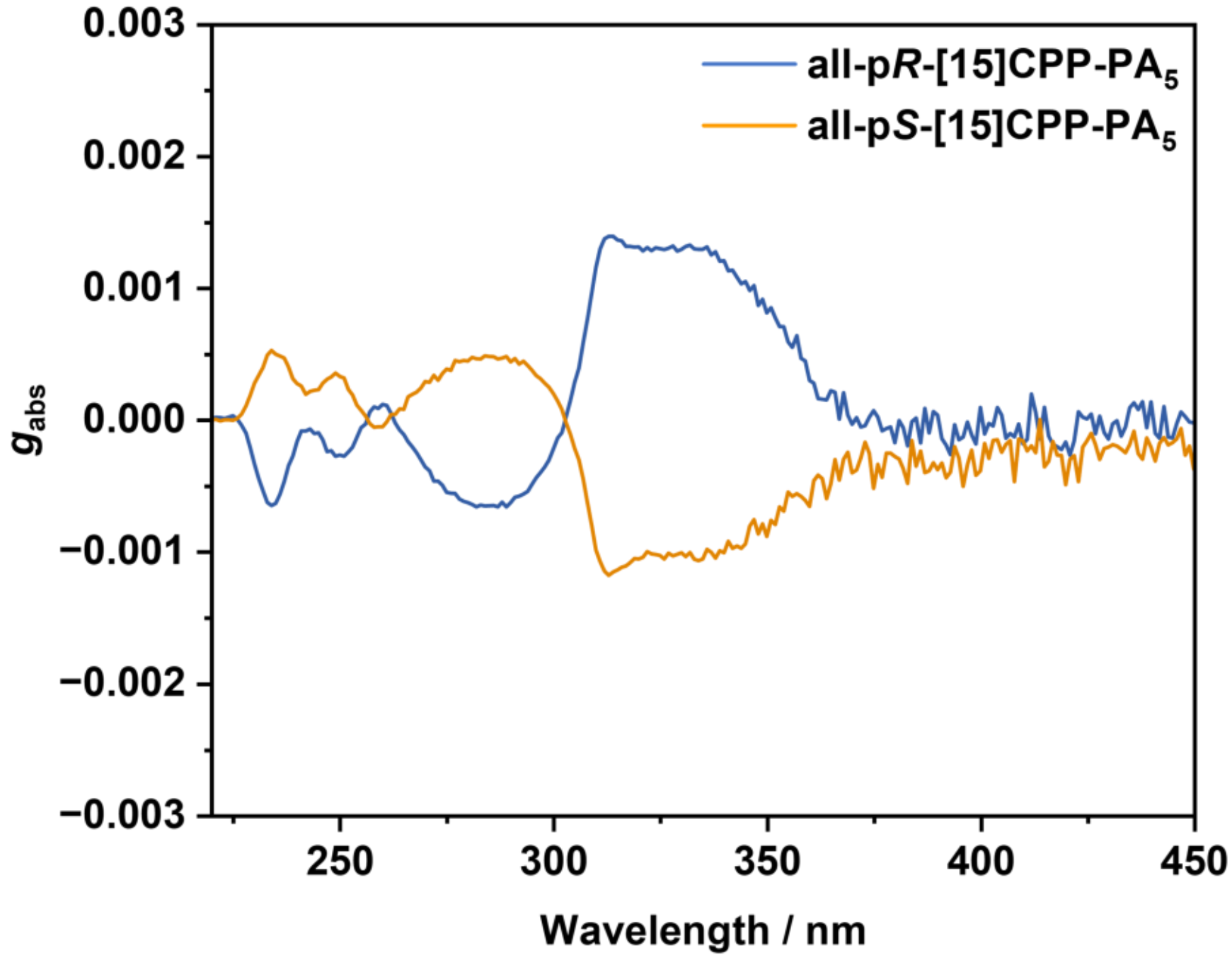

Figure S 48: Plot of g_{abs} of **all-p*R*-** and **all-p*S*-[15]CPP-PA$_5$** in CH_2Cl_2 (10^{-5} to 10^{-6} M).

11. Circularly Polarized Luminescence Spectra

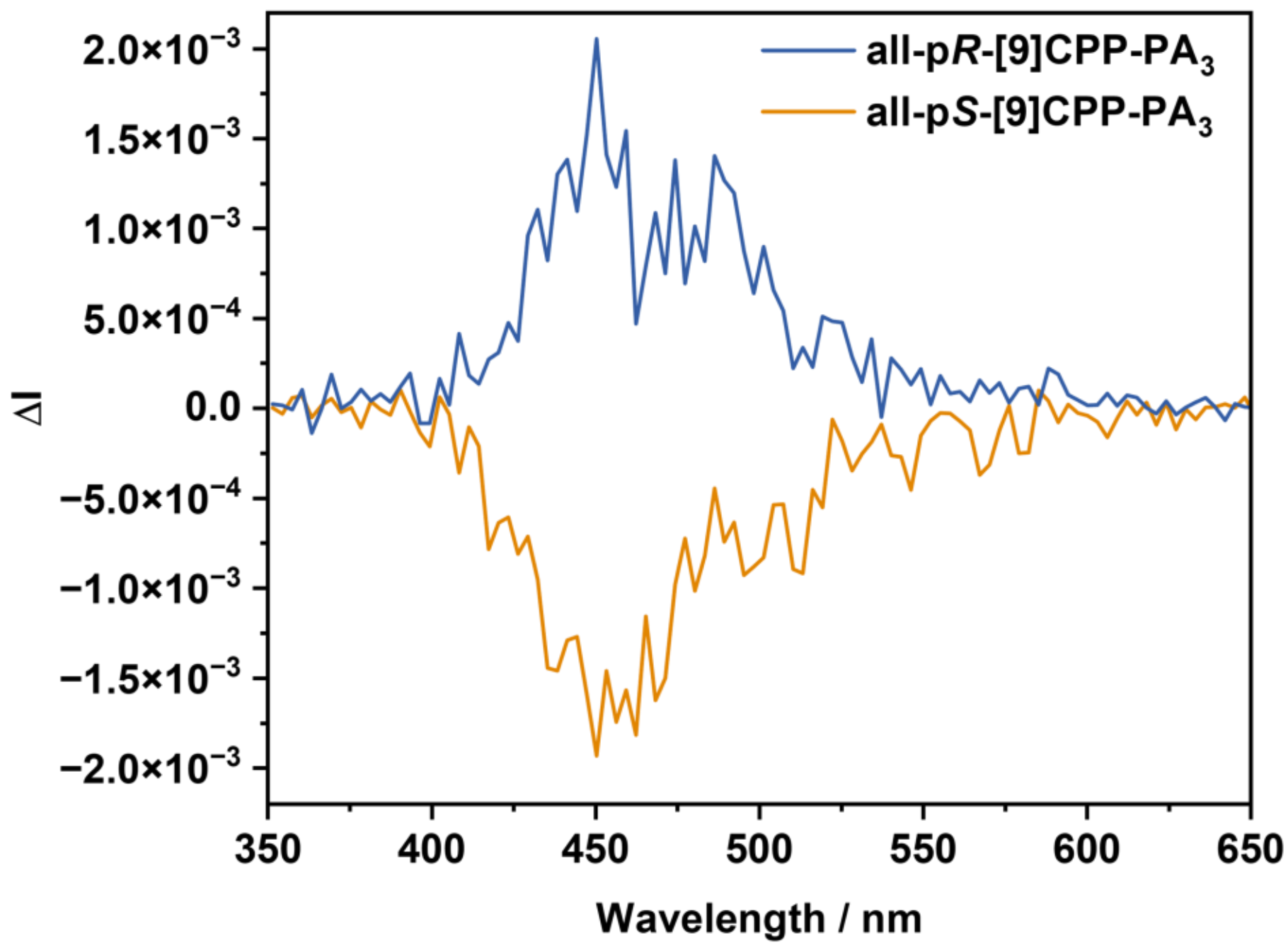

Figure S 49: CPL spectra of **all-p*R*-** and **all-p*S*-[9]CPP-PA₃** in CH_2Cl_2 (PEM-based setup, 10^{-5} to 10^{-6} M, λ_{exc} = 343 nm).

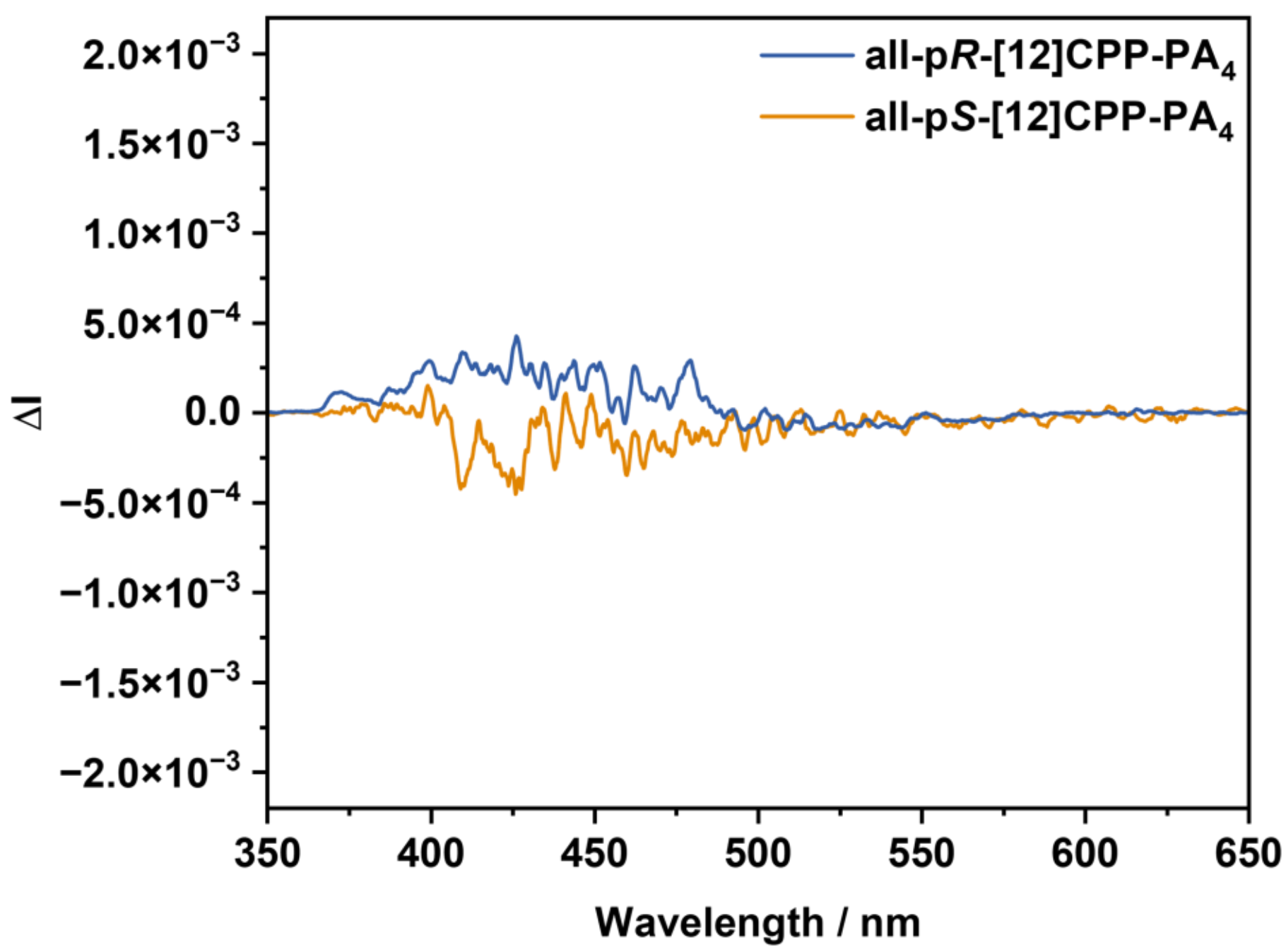

Figure S 50: CPL spectra of **all-p*R*-** and **all-p*S*-[12]CPP-PA₄** in CH_2Cl_2 (QWP-based setup, 10^{-5} to 10^{-6} M, λ_{exc} = 335 nm).

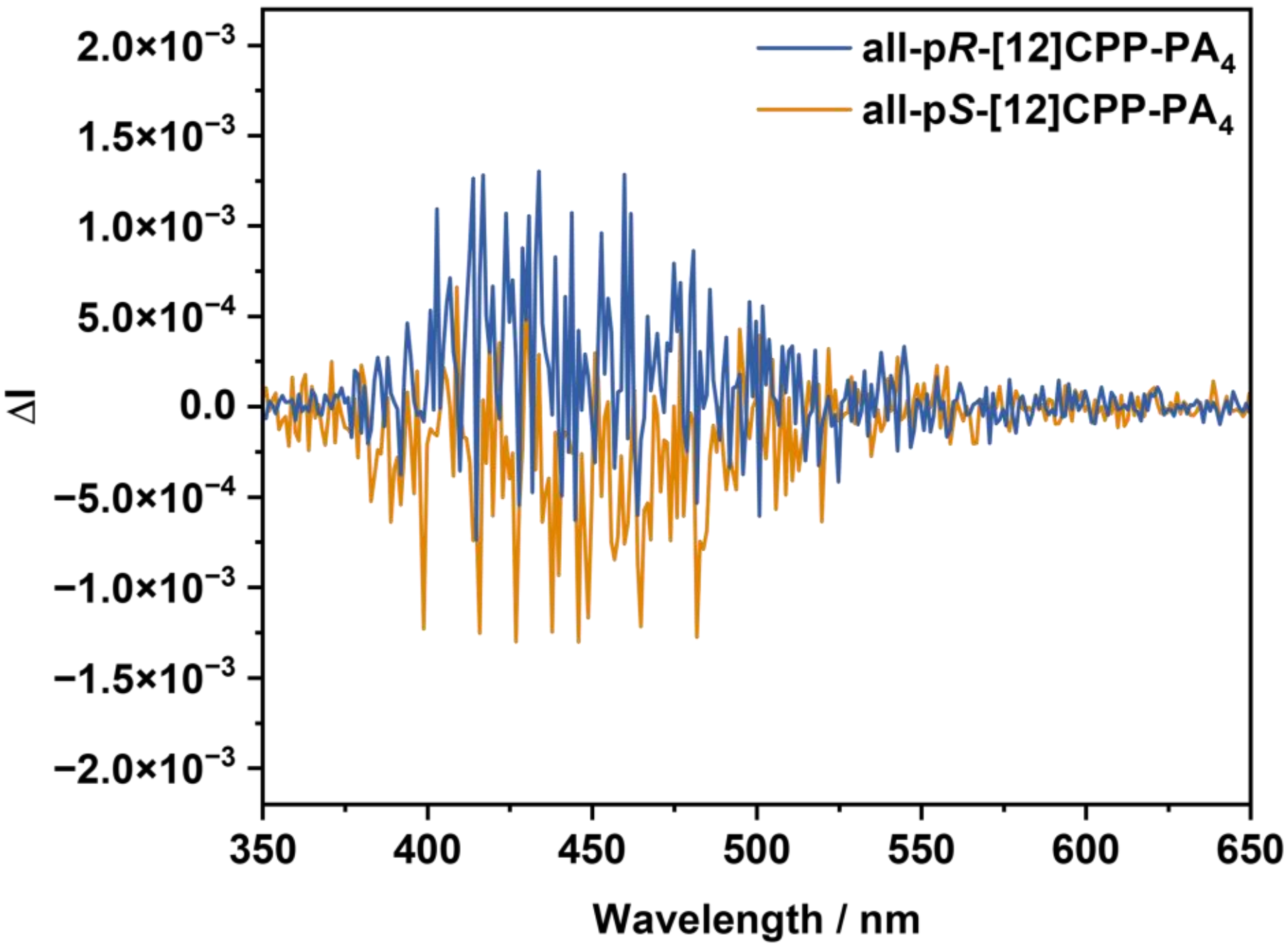

Figure S 51: CPL spectra of **all-p*R*-** and **all-p*S*-[12]CPP-PA$_4$** in CH_2Cl_2 (PEM-based setup, 10^{-5} to 10^{-6} M, λ_{exc} = 343 nm).

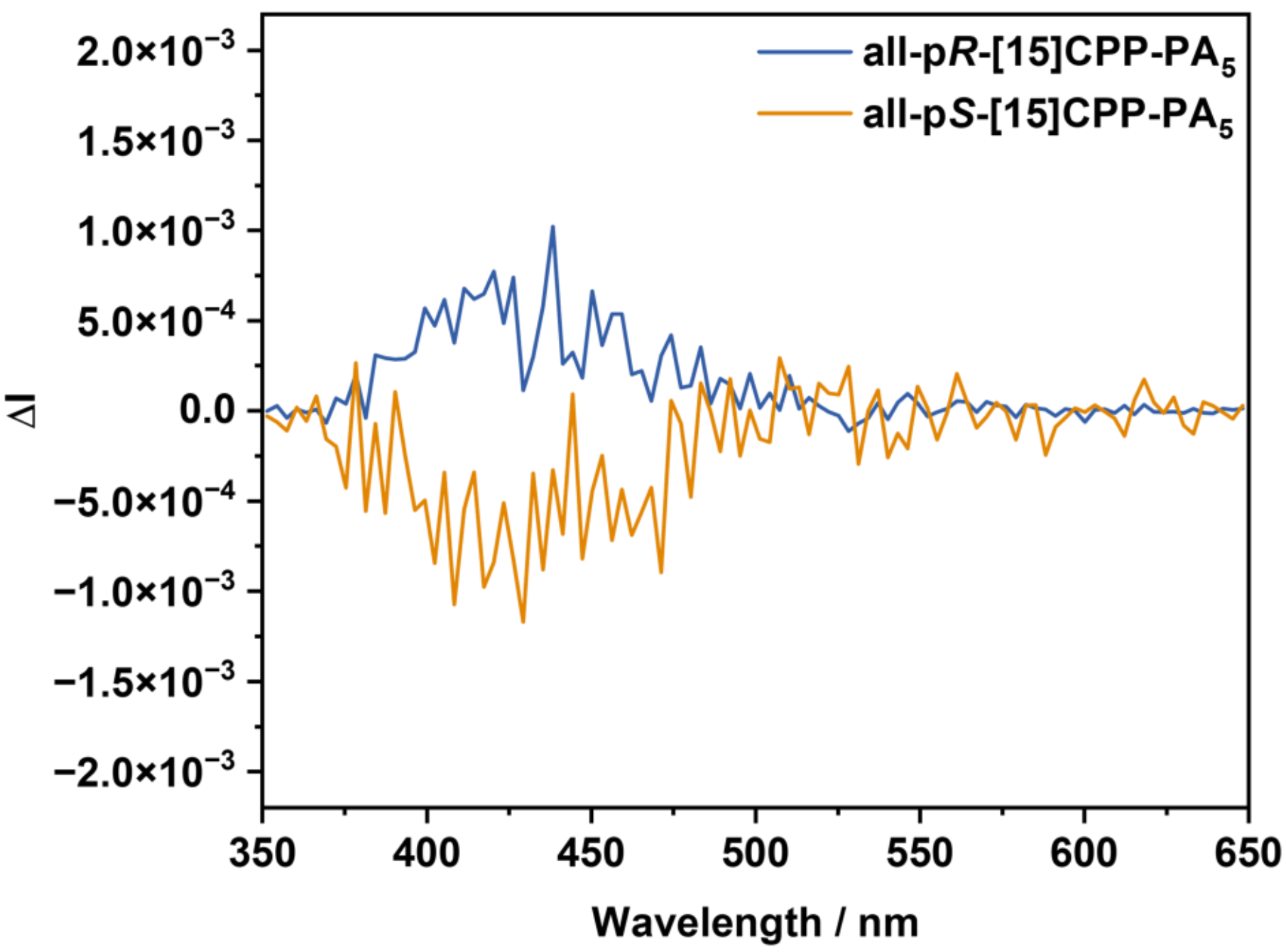

Figure S 52: CPL spectra of **all-p*R*-** and **all-p*S*-[15]CPP-PA$_5$** in CH_2Cl_2 (PEM-based setup, 10^{-5} to 10^{-6} M, λ_{exc} = 343 nm).

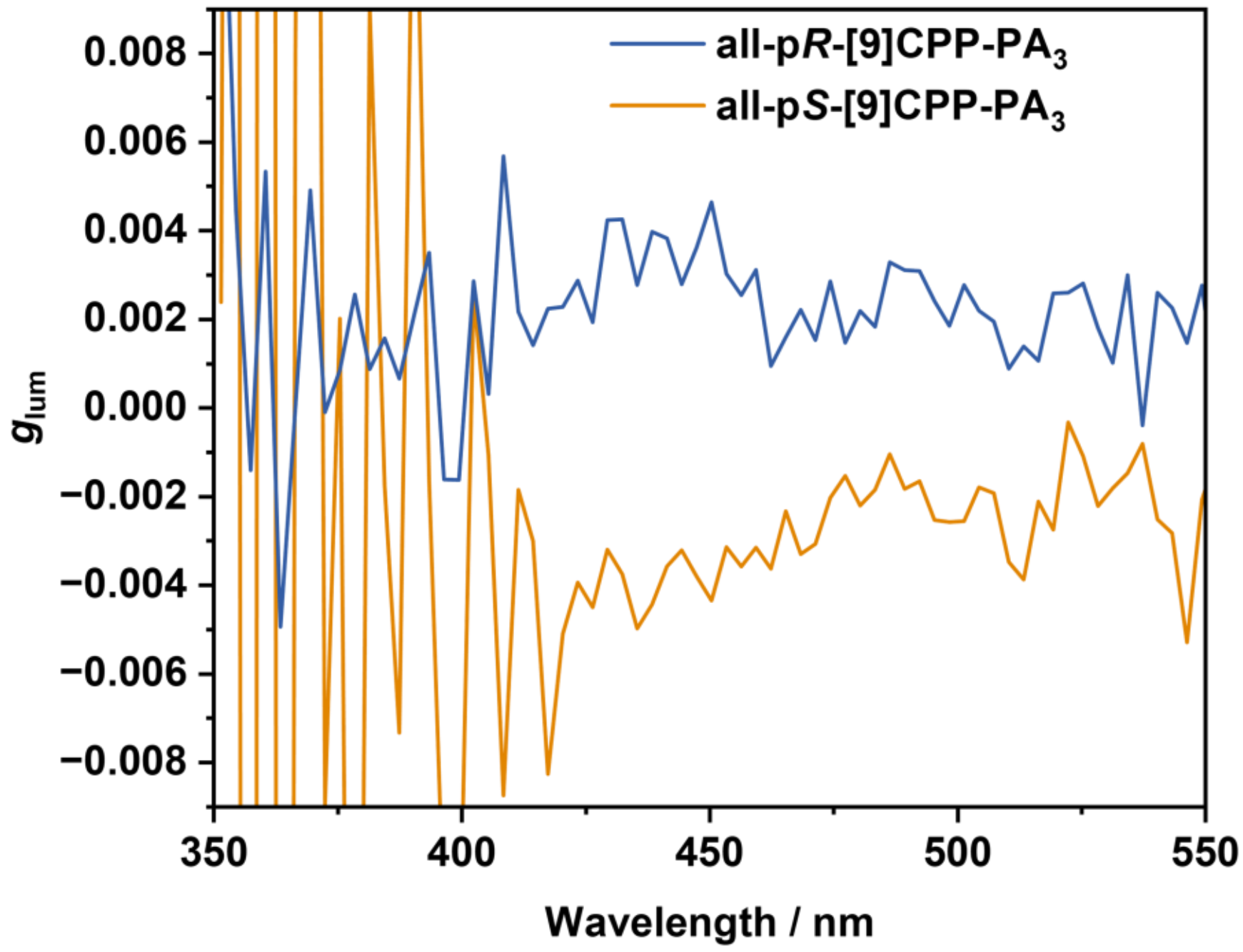

Figure S 53: Plot of g_{lum} of **all-p*R*-** and **all-p*S*-[9]CPP-PA₃** in CH_2Cl_2 (PEM-based setup, 10^{-5} to 10^{-6} M, λ_{exc} = 343 nm).

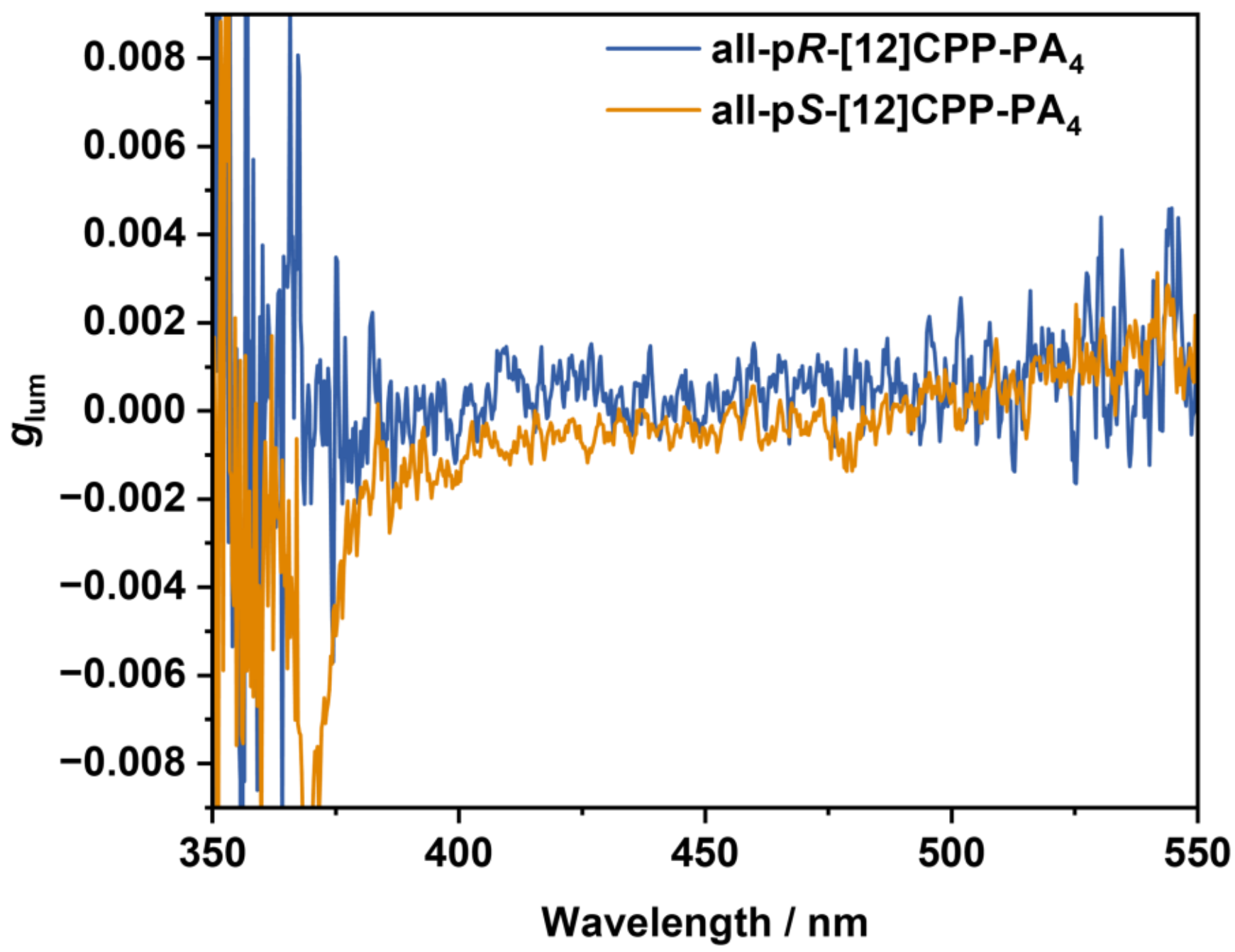

Figure S 54: Plot of g_{lum} of **all-p*R*-** and **all-p*S*-[12]CPP-PA$_4$** in CH_2Cl_2 (QWP-based setup, 10^{-5} to 10^{-6} M, λ_{exc} = 335 nm).

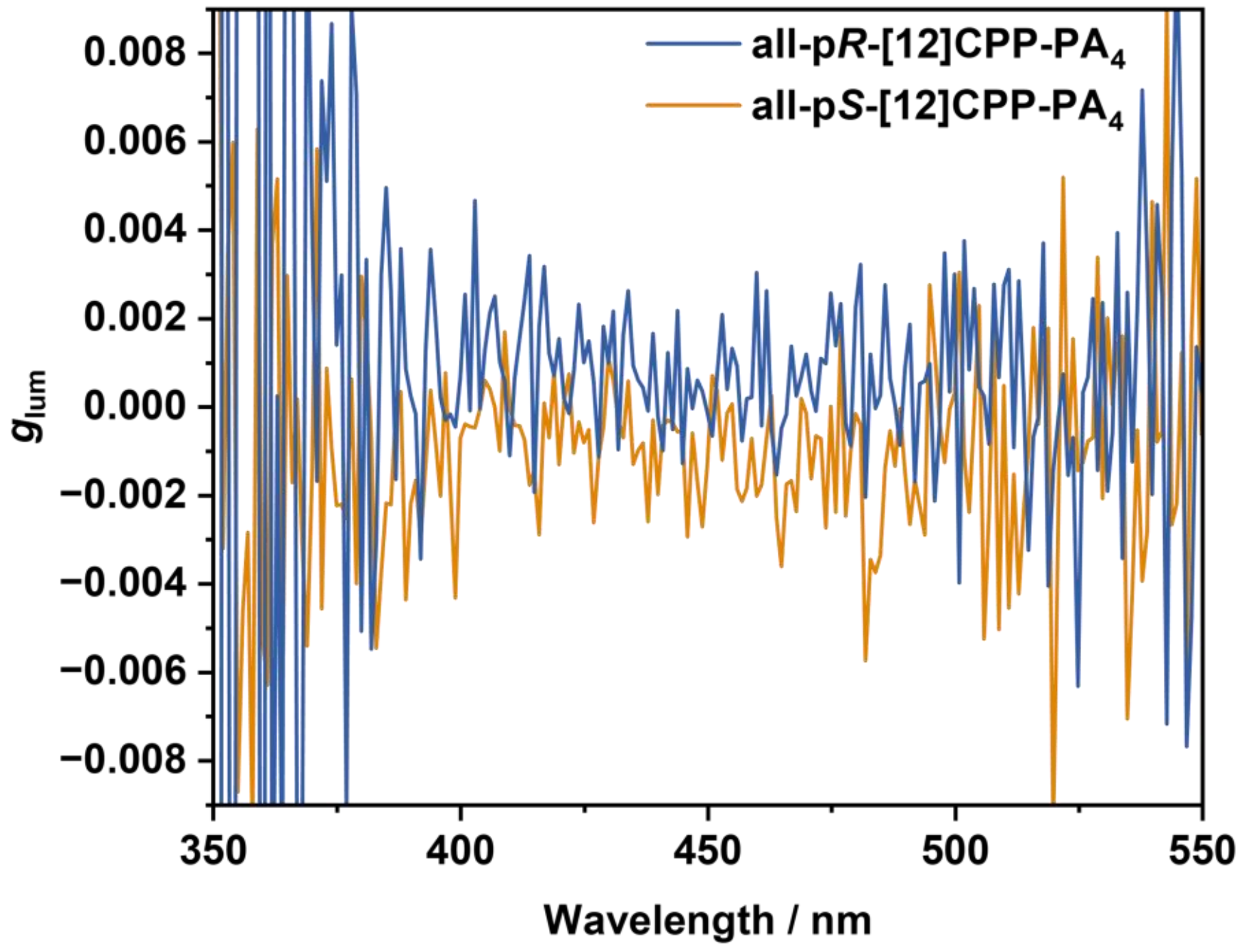

Figure S 55: Plot of g_{lum} of **all-p*R*-** and **all-p*S*-[12]CPP-PA$_4$** in CH_2Cl_2 (PEM-based setup, 10^{-5} to 10^{-6} M, λ_{exc} = 343 nm).

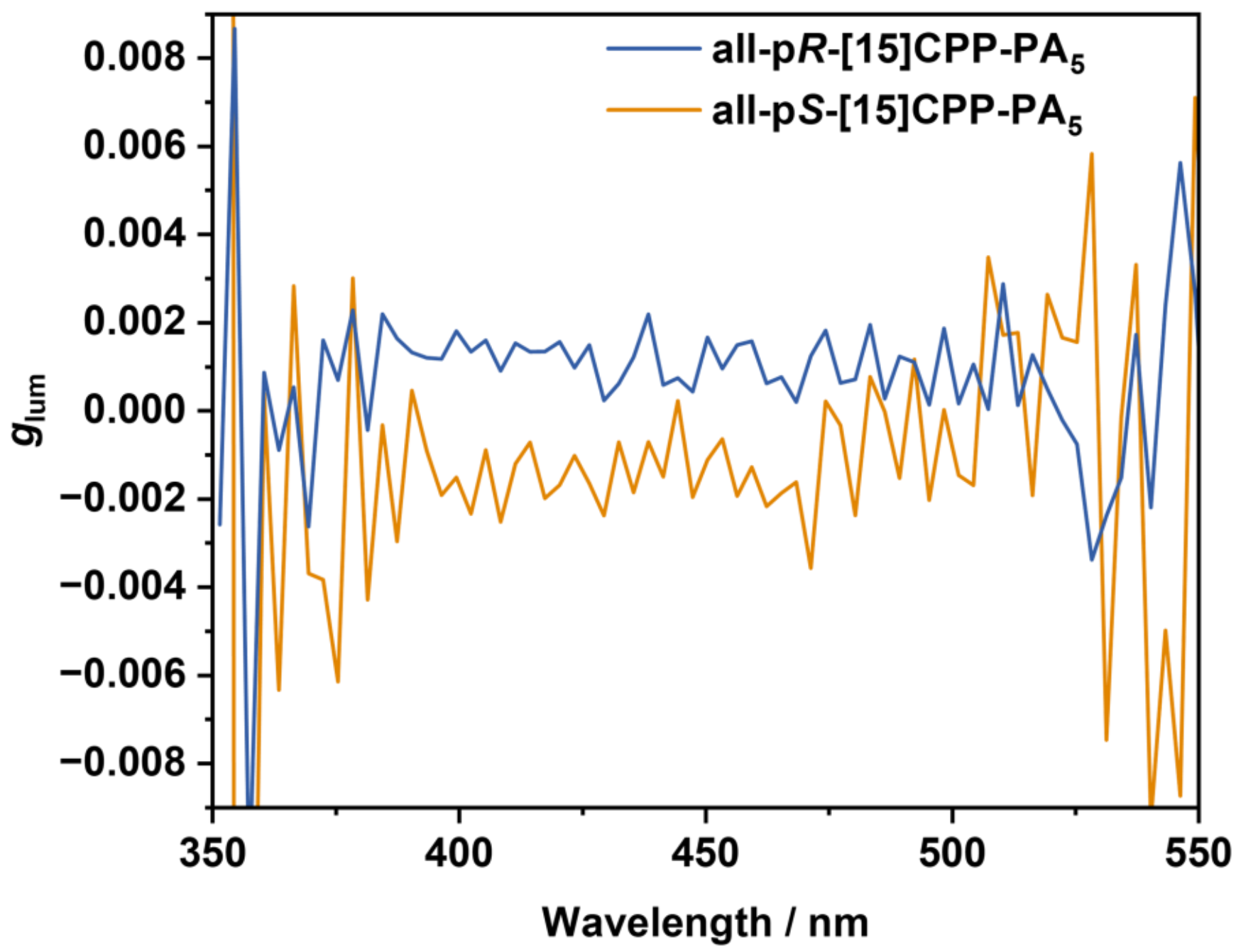

Figure S 56: Plot of g_{lum} of **all-p*R*-** and **all-p*S*-[15]CPP-PA$_5$** in CH_2Cl_2 (PEM-based setup, 10^{-5} to 10^{-6} M, λ_{exc} = 343 nm).

12. Computational Results

12.1 Optimized Geometries

XYZ coordinates of the optimized geometries can be found in Section 12.5.

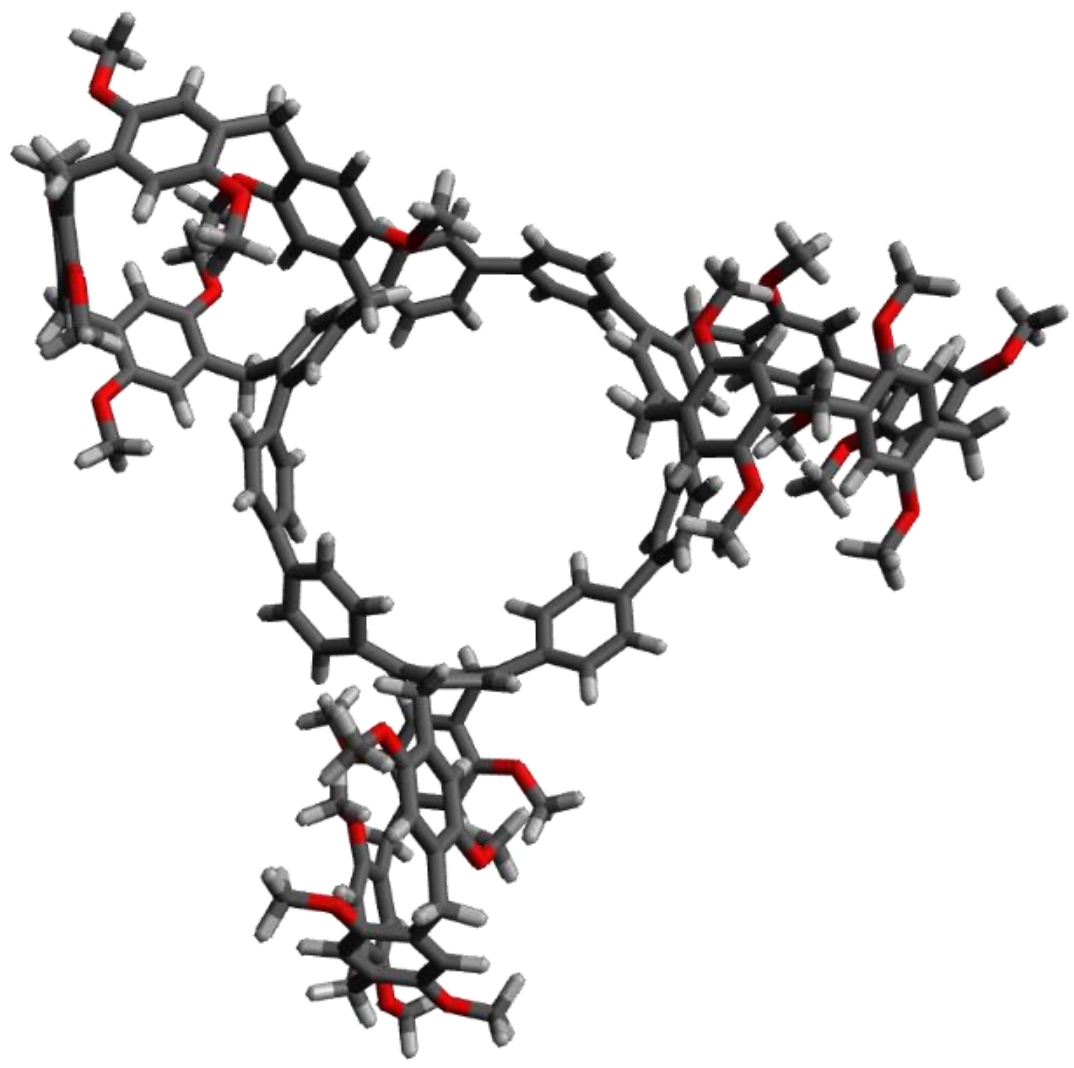

Figure S 57: Optimized geometry (GFN2-xTB) of **all-p*R*-[9]CPP-PA$_3$**.

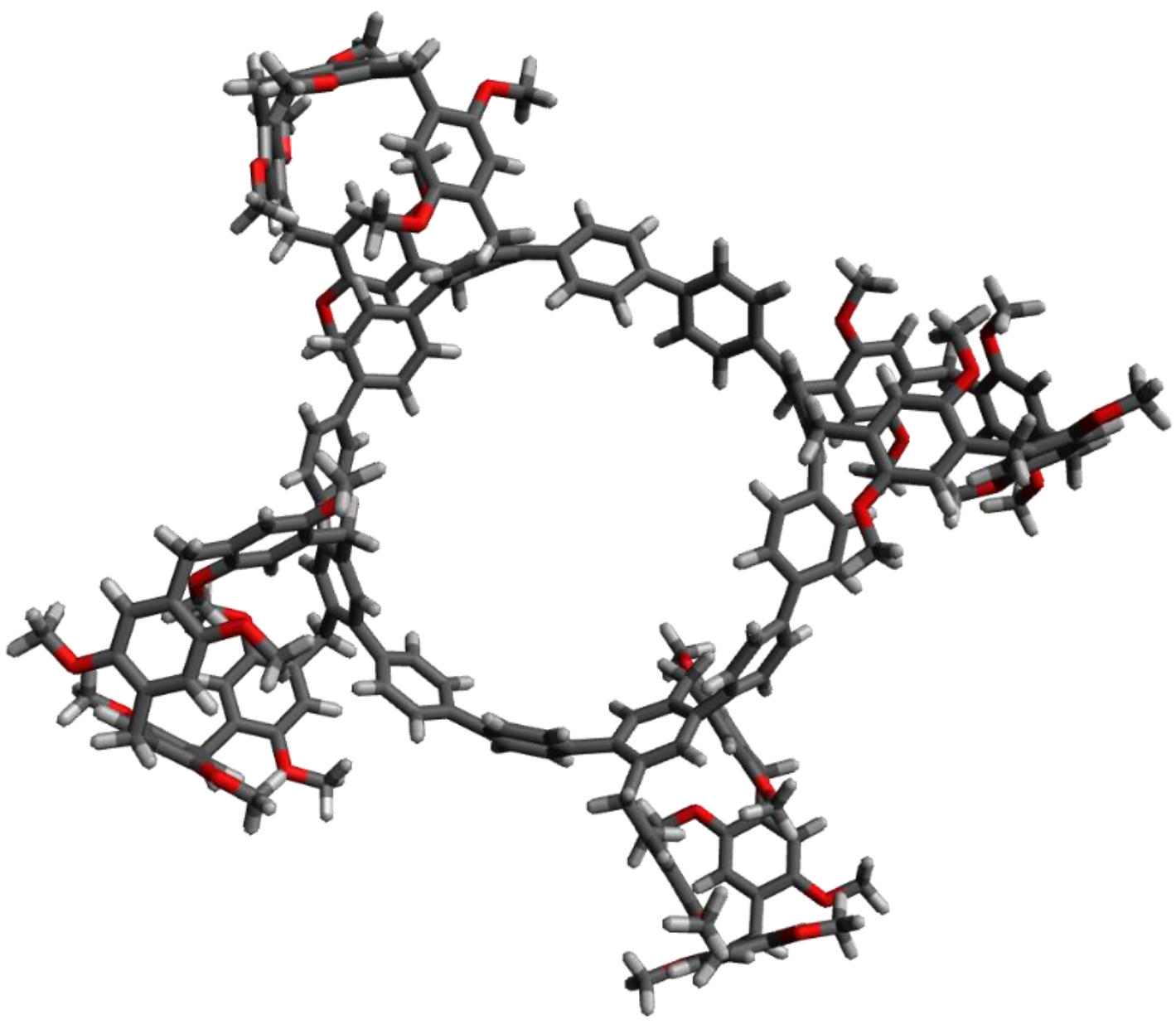

Figure S 58: Optimized geometry (GFN2-xTB) of **all-p*R*-[12]CPP-PA$_4$**.

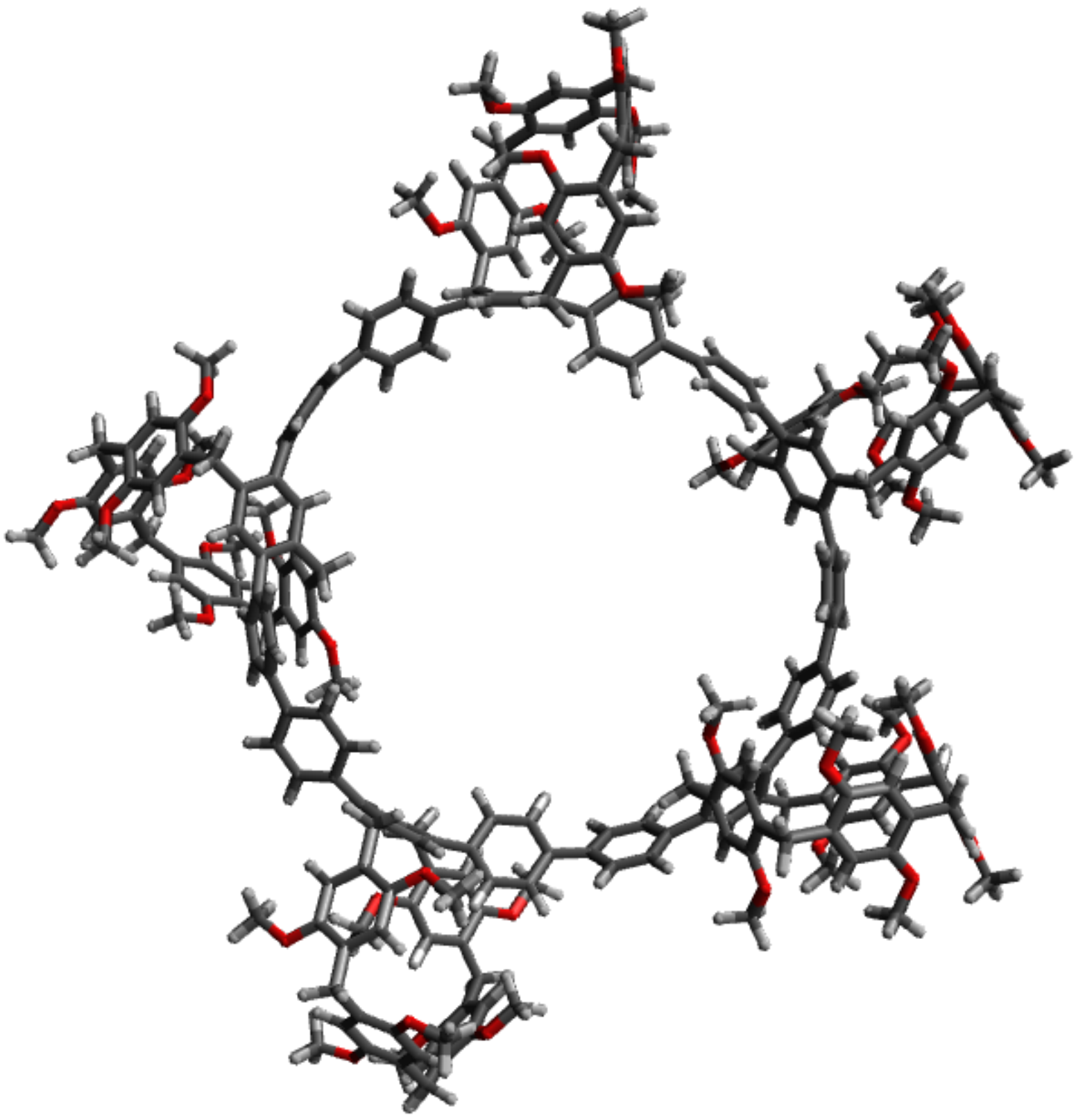

Figure S 59: Optimized geometry (GFN2-xTB) of **all-p*R*-[15]CPP-PA₅**.

12.2 sTD-DFT Results

Table S 4: Vertical excitations below 5 eV calculated for **all-p*R*-[9]CPP-PA$_3$** at the sTDDFT/ωB97X/def2-SV(P) level of theory. E_{exc} = excitation energy. λ = corresponding wavelength. f = oscillator strength. R = rotatory strength.

**Exc. #**	E_{exc} **/ eV**	λ **/ nm**	f	R
1	3.55	349	0.04	279
2	3.99	311	1.55	483
3	4.13	300	1.39	-605
4	4.58	271	0.04	49.1
5	4.71	263	0.03	-285
6	4.76	261	0.01	16.9
7	4.76	260	0.04	-251
8	4.78	260	0.04	-37.4
9	4.79	259	0.09	786
10	4.83	257	0.18	168
11	4.86	255	0.01	5.37
12	4.87	254	0.09	-87.4
13	4.90	253	0.07	22.8
14	4.91	253	0.24	-375
15	4.94	251	0.15	-236
16	4.95	251	0.15	-183
17	4.95	250	0.02	7.9
18	4.95	250	0.07	126
19	4.96	250	0.11	40.5
20	4.97	250	0.01	-5.31
21	4.99	248	0.00	-5.82
22	5.00	248	0.03	0.0282

Table S 5: Vertical excitations below 5 eV calculated for **all-p*R*-[12]CPP-PA$_4$** at the sTDDFT/ωB97X/def2-SV(P) level of theory. E_{exc} = excitation energy. λ = corresponding wavelength. f = oscillator strength. R = rotatory strength.

**Exc. #**	**E_{exc} / eV**	**λ / nm**	***f***	***R***
1	3.89	319	0.04	553
2	4.13	300	1.55	-541
3	4.30	288	1.39	603
4	4.61	269	0.04	-221
5	4.63	268	0.03	-205
6	4.78	260	0.01	50.2
7	4.78	259	0.04	-364
8	4.80	259	0.04	317
9	4.81	258	0.09	31.9
10	4.89	254	0.18	384
11	4.91	253	0.01	295
12	4.93	252	0.09	249
13	4.93	251	0.07	-236
14	4.95	251	0.24	45.4
15	4.97	250	0.15	286
16	4.98	249	0.15	334
17	4.98	249	0.02	-659
18	4.99	249	0.07	238
19	4.99	248	0.11	-776
20	5.00	248	0.01	-0.703

Table S 6: Vertical excitations below 5 eV calculated for **all-p*R*-[15]CPP-PA$_5$** at the sTDDFT/ωB97X/def2-SV(P) level of theory. E_{exc} = excitation energy. λ = corresponding wavelength. f = oscillator strength. R = rotatory strength.

**Exc. #**	$\boldsymbol{E_{exc}}$ **/ eV**	$\boldsymbol{\lambda}$ **/ nm**	$\boldsymbol{f}$	$\boldsymbol{R}$
1	4.08	304	0.09	609
2	4.25	292	2.43	169
3	4.28	289	2.48	32.6
4	4.52	274	0.04	-237
5	4.63	268	0.09	-281
6	4.78	259	0.01	-196
7	4.80	259	0.04	-594
8	4.80	258	0.05	212
9	4.81	258	0.03	-5.57
10	4.81	258	0.05	453
11	4.89	253	0.10	479
12	4.91	252	0.11	-218
13	4.93	252	0.07	73.1
14	4.94	251	0.35	665
15	4.95	251	0.14	-298
16	4.96	250	0.31	698
17	4.96	250	0.16	-452
18	4.98	249	0.03	191
19	4.98	249	0.27	25.3
20	5.00	248	0.14	-141
21	5.00	248	0.12	-449

12.3 Calculated Absorption Spectra

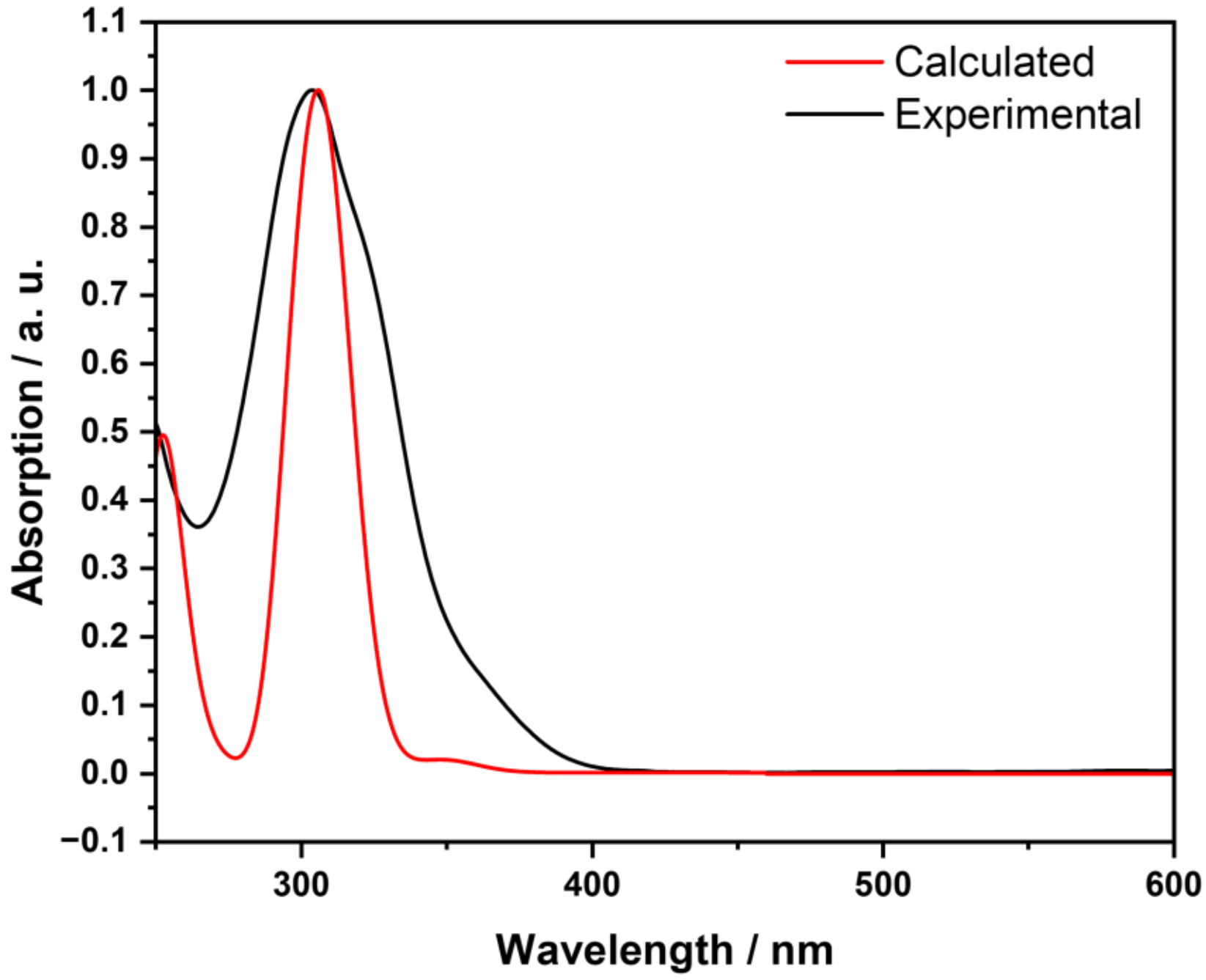

Figure S 60: Calculated (sTDDFT/ωB97X/def2-SV(P) level of theory, σ = 0.16) compared to experimental absorption spectrum (normalized) of **all-p*R*-[9]CPP-PA$_3$**.

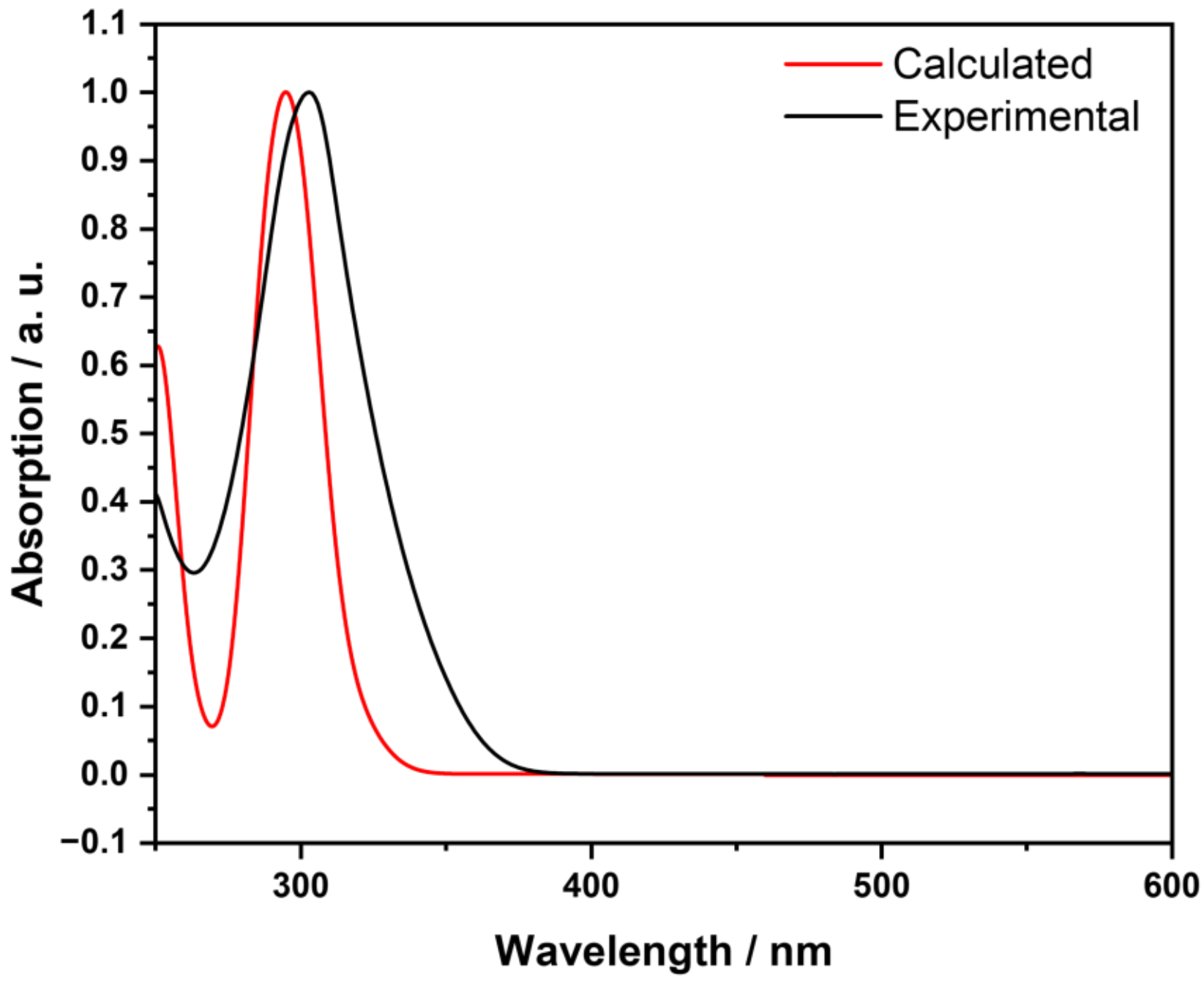

Figure S 61: Calculated (sTDDFT/ωB97X/def2-SV(P) level of theory σ = 0.16) compared to experimental absorption spectrum (normalized) of **all-p*R*-[12]CPP-PA$_4$**.

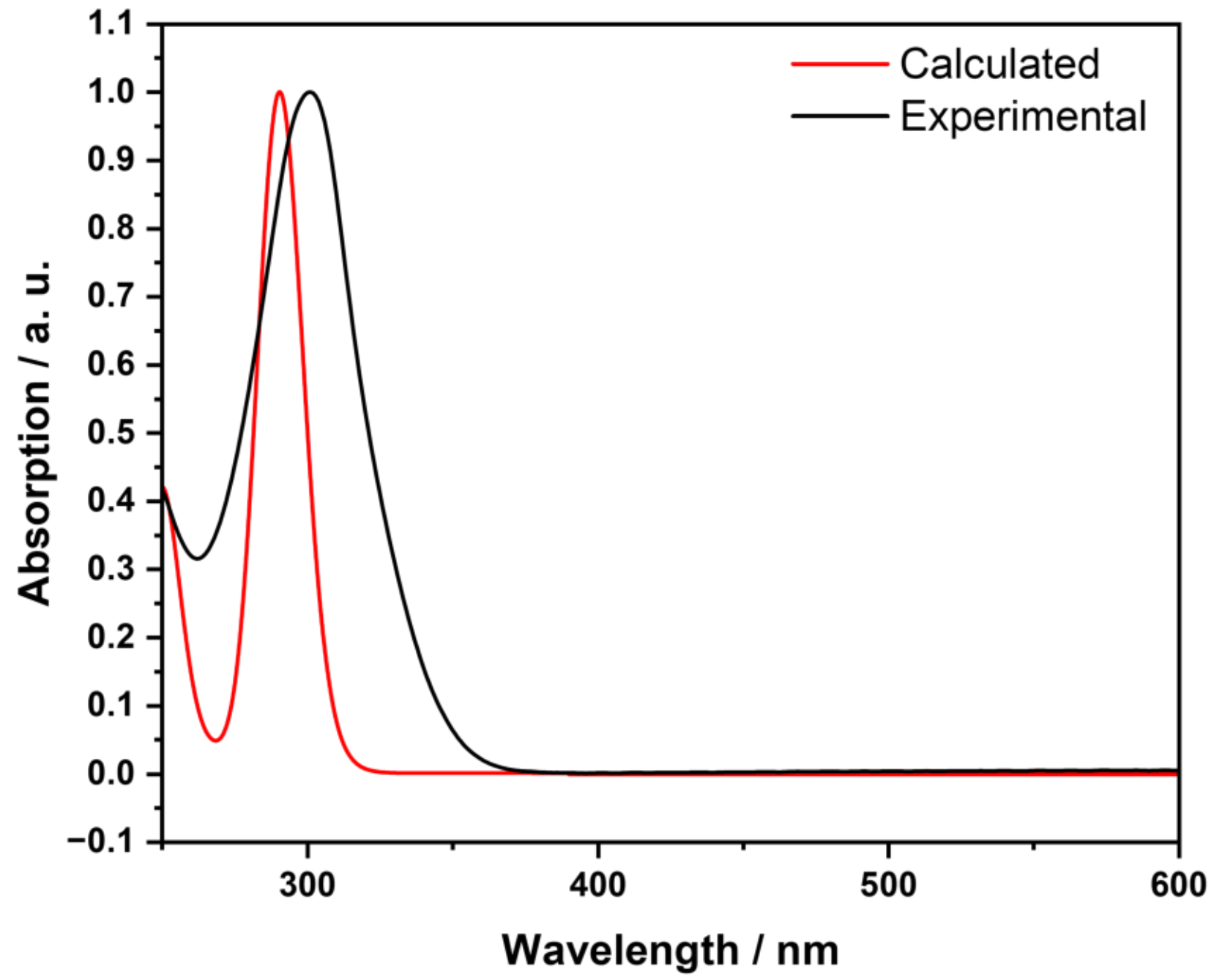

Figure S 62: Calculated (sTDDFT/ωB97X/def2-SV(P) level of theory σ = 0.16) compared to experimental absorption spectrum (normalized) of **all-p*R*-[15]CPP-PA$_5$**.

12.4 Calculated ECD Spectra

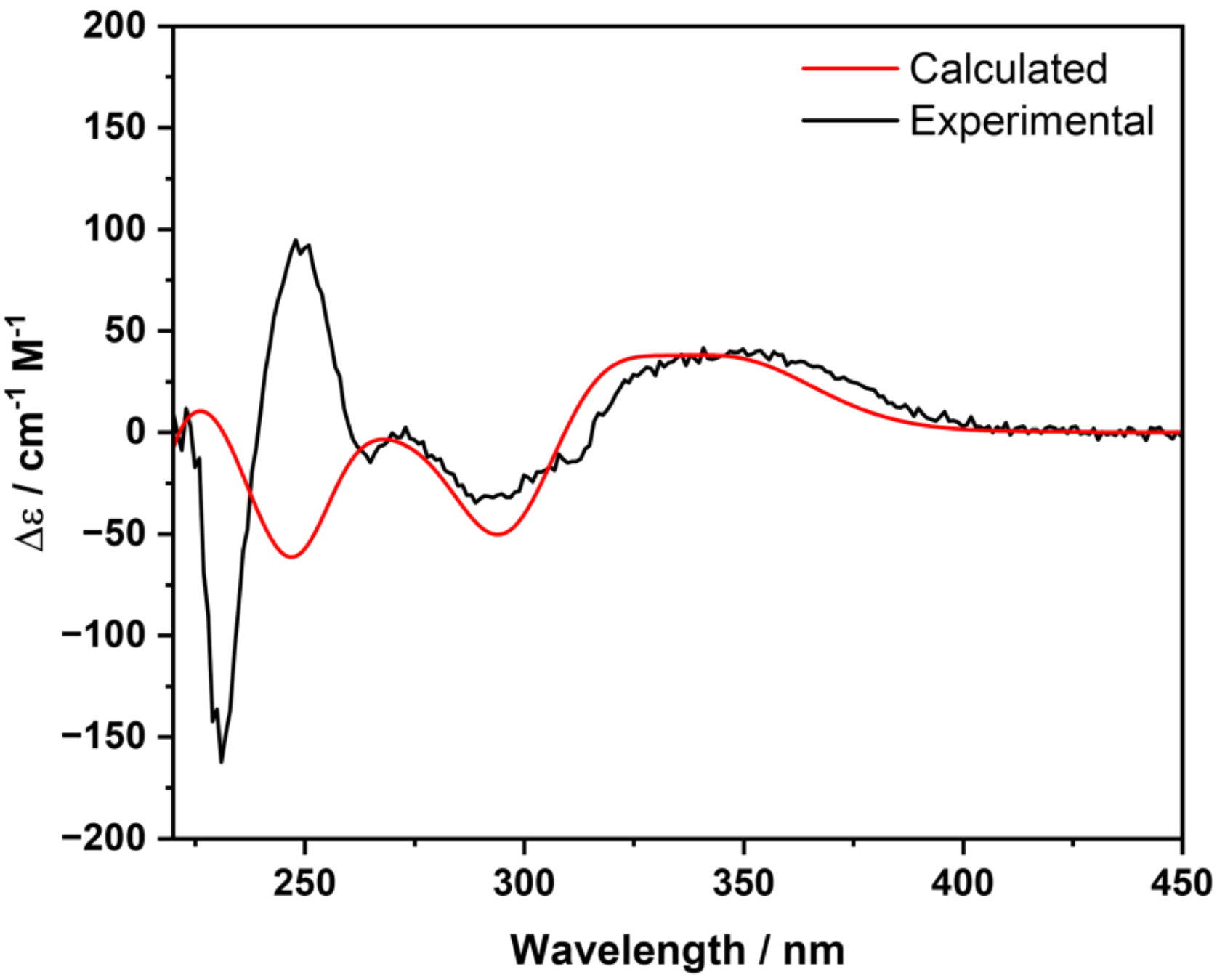

Figure S 63: Calculated (sTDDFT/ωB97X/def2-SV(P) level of theory, σ = 0.25, intensity adjusted to fit experimental spectrum) compared to experimental ECD spectrum of **all-p*R*-[9]CPP-PA$_3$**.

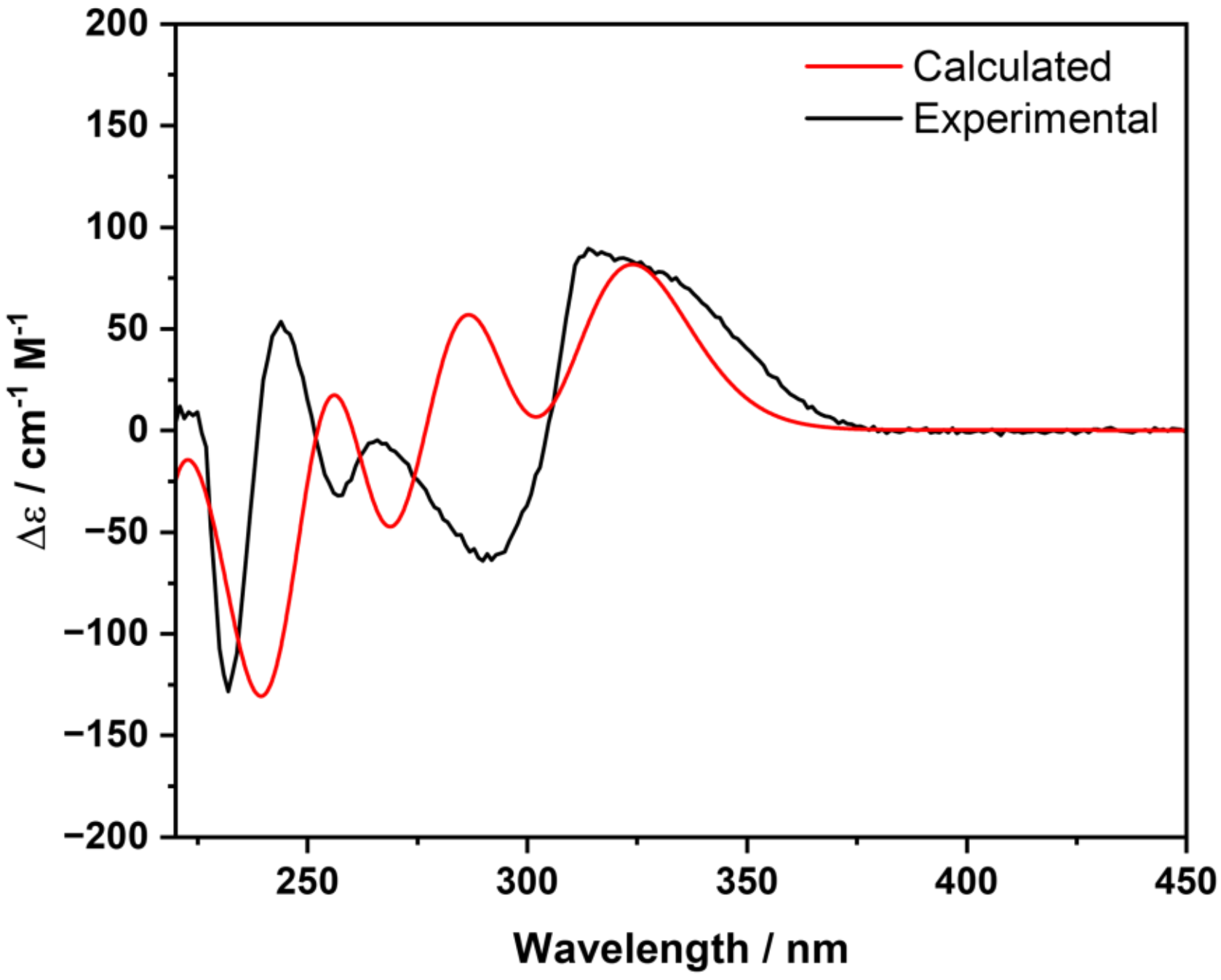

Figure S 64: Calculated (sTDDFT/ωB97X/def2-SV(P) level of theory, σ = 0.25, intensity adjusted to fit experimental spectrum) compared to experimental ECD spectrum of **all-p*R*-[12]CPP-PA$_4$**.

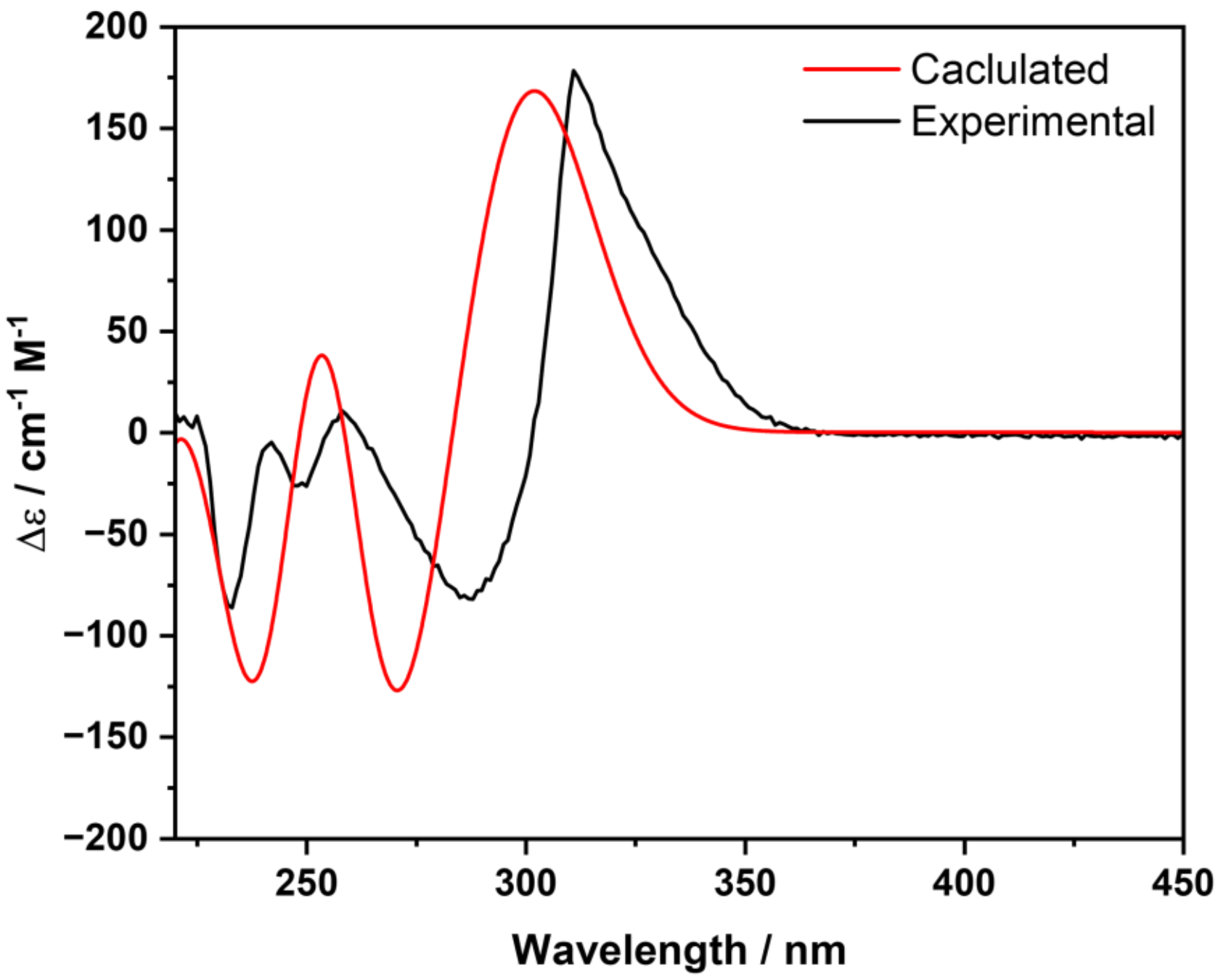

Figure S 65: Calculated (sTDDFT/ωB97X/def2-SV(P) level of theory, σ = 0.25, intensity adjusted to fit experimental spectrum) compared to experimental ECD spectrum of **all-p*R*-[15]CPP-PA$_5$**

12.5 XYZ Coordinates of Optimized Geometries

Table S 7: XYZ coordinates of the optimized geometry (GFN2-xTB) of **all-p*R*-[9]CPP-PA₃**.

C	-2.83087941463615	-7.39021356618902	4.79684325373752
C	-4.11931971150646	-7.93042869084526	4.73423602738278
C	-4.74912038574237	-8.31514536666337	5.91072929293940
C	-4.14108372935812	-8.16172631435852	7.14667860700647
C	-2.86542686349983	-7.59308840621032	7.21121628034145
C	-2.22721034752222	-7.22679845870886	6.03362854921117
C	-4.85748295586941	-8.61306255563479	8.39385756748126
C	-5.88346300064461	-7.59998476956305	8.83569254635943
C	-7.24721485939958	-7.80191323756045	8.60743689133724
C	-8.15700719086751	-6.86106508011739	9.07127887344219
C	-7.74777642734018	-5.71041917962823	9.72559147928249
C	-6.38024685082458	-5.48781887203271	9.91610925988972
C	-5.47242774463047	-6.44434823016704	9.48147802019670
C	-8.77635330109309	-4.69532043067081	10.16010345909441
C	-9.08562027512382	-3.80902135044106	8.98040577927725
C	-10.10886329158994	-4.12995985006612	8.08459798009716
C	-10.26187909092984	-3.37216584239570	6.93108957912167
C	-9.42158413049335	-2.30985847301814	6.64401272365192
C	-8.44539426238404	-1.94385694915713	7.57673855762783
C	-8.28772476329924	-2.70504574863325	8.72558550809508
C	-9.48749679145709	-1.60840115750120	5.31345222959188
C	-8.13915191711709	-1.63487545811439	4.63676115560504
C	-7.38953532637462	-2.81498422924657	4.58338579431225
C	-6.11104060155946	-2.78552324821666	4.04290986836666
C	-5.57063866127973	-1.62767908673629	3.50988375468315
C	-6.33848846597085	-0.45881062026210	3.51838612531424
C	-7.60345935438270	-0.47852726501429	4.09012745500502
O	-7.97616633258539	-3.95686944157351	5.07015062482898
O	-5.75316928966142	0.64845153252899	2.95453150068978
C	-7.18883890000202	-5.11125772370746	5.22192528447887
C	-6.48594313666448	1.84959001314153	2.91880040850916
O	-10.89628930109525	-5.21287005146865	8.40276761245832
O	-7.68685843499627	-0.84155570767945	7.27103911262921
C	-6.57496357705998	-0.54866687097813	8.08048605624854
C	-11.94963485892665	-5.55527025516931	7.53455293898523
O	-7.60378756276398	-8.94592064997249	7.93197955067198
C	-8.97298287576521	-9.20253307213957	7.72815375043100
O	-6.02210329870564	-4.32032129212885	10.54731748098501
C	-4.65475433447720	-4.07432292647887	10.77327285008931
O	-2.31274388385490	-7.44499358991482	8.46087258958965
C	-0.98821404437234	-6.97763198708518	8.56021135834523
O	-4.67178364861550	-8.06527974185984	3.48507678596797
C	-5.88827359901690	-8.76305703198098	3.36248614267343
C	-5.41090084161442	-2.74041519853034	0.39603765421930
C	-4.27710311841371	-3.51003501225255	0.64727903234680
C	-3.37876795360624	-3.70087254986347	-0.40458258520128
C	-3.49585103434046	-2.98380119892765	-1.57357147795103
C	-4.52717838107994	-2.05584961988425	-1.75201443424910

C	-5.53633246277381	-2.03315805060093	-0.78698818501070
C	-4.33777459612843	-1.00089361330744	-2.75586864040833
C	-3.51442566947016	-1.18302201437820	-3.87005994916297
C	-2.90537896246940	-0.10984004674275	-4.48705045253130
C	-3.11453979911963	1.19460973351278	-4.03463829801926
C	-4.13816849579725	1.38328146160743	-3.10111902999716
C	-4.72765684108990	0.31023701541395	-2.46656107976719
C	-2.08586519308496	2.22415438900938	-4.25079607562194
C	-1.32097393007483	2.41036812871831	-5.40994395065954
C	-0.09121899954511	3.03448419658844	-5.28147023286773
C	0.39364693910313	3.54407203904199	-4.07419861996718
C	-0.49393832888857	3.59725995457015	-2.98607454098842
C	-1.68217758619884	2.89737930146827	-3.09880779059754
C	1.84589459501908	3.65831156162227	-3.86484339340737
C	2.61524885278603	2.57076537840981	-4.29591892823132
C	3.83917240089600	2.29952939631114	-3.72995611543201
C	4.33563927334800	3.09223118292326	-2.68975158724083
C	3.69951462206197	4.31162167574929	-2.46237720432205
C	2.48105574625939	4.59804590412527	-3.05391769753694
C	5.26190467803660	2.51169390069102	-1.70846643646165
C	5.02480359208810	1.17362757755428	-1.38067048109860
C	5.62097807230405	0.59126526810398	-0.29012177009223
C	6.49494368213223	1.31054548254440	0.52941425925227
C	6.79753463583791	2.61862131795078	0.15964261197595
C	6.19044817673289	3.20945486820396	-0.93849575563020
C	6.80754830816035	0.67704074272525	1.81886728866191
C	6.58278748767389	1.37715017978832	3.00440281287540
C	6.33235162441061	0.73915077942532	4.21136815714819
C	6.19894496105270	-0.65864096591967	4.20244803081284
C	6.68141590624638	-1.34302936051414	3.08574441163387
C	7.03178789289237	-0.70494717886576	1.91116653812781
C	5.29386960831173	-1.40409704104533	5.09010638651912
C	5.43326085090824	-2.76853820156997	5.35403932613022
C	4.34210970194076	-3.54121412384173	5.70641609736181
C	3.07942141571409	-2.96921781147134	5.84498806547949
C	2.98410422586535	-1.57846953350257	5.78042985240962
C	4.06283730758653	-0.81447828063545	5.39442970006174
C	1.81856956352370	-3.71886197150337	5.73297826344344
C	1.68140070697034	-4.55972359597468	4.62775597979936
C	0.43385272557802	-4.92198154162599	4.16692671695810
C	-0.72451416758621	-4.48438818988032	4.81170642001813
C	-0.57558666628688	-3.79783952476438	6.01751831218771
C	0.67400671016240	-3.41123020949204	6.46573762900463
C	-1.98512799826413	-4.47565225827933	4.04981722259771
C	-2.46029420136760	-5.52488643479124	3.25113312740985
C	-3.31550891661547	-5.21624339847366	2.20421693005069
C	-3.78765477358942	-3.92548273393320	1.97470510302185
C	-3.52578669821915	-2.94224593993736	2.94143477619653
C	-2.60477795256282	-3.23308661623337	3.93227402787722
C	-0.34803172098745	5.78569462262143	-1.74486885176117
C	-1.43502178881093	6.39209954112035	-2.38695815207264
C	-1.46847660057712	7.77609015240259	-2.49972870626614
C	-0.47218324511318	8.58049767855536	-1.97296851353463

C	0.57234379835209	7.98365839600714	-1.26272286434211
C	0.61105331613784	6.59887197308617	-1.15552501924653
C	-0.49782017431055	10.06234228179870	-2.24724531026294
C	-0.01931006707181	10.27489575908530	-3.66232108874170
C	-0.91350989429467	10.51647051444234	-4.70924518206232
C	-0.42073795055780	10.64839799799311	-6.00114172283423
C	0.93040142519887	10.52248300420182	-6.28392466169169
C	1.82170643542526	10.26302697152945	-5.23809097342676
C	1.33213877769658	10.15387711853578	-3.94377675474201
C	1.40593778966576	10.63306425142072	-7.70964476707072
C	1.11250069556241	9.36454500613307	-8.47347892543826
C	0.25596583477849	9.35651444990623	-9.57685293188315
C	0.03452255667587	8.16152302392813	-10.25409164375225
C	0.63270770983372	6.97649292891006	-9.85835215918841
C	1.47590394694013	6.98271108266884	-8.73962509851158
C	1.70291917183390	8.17616391512257	-8.06999858930825
C	0.38276400516002	5.69154894667988	-10.60281013741970
C	-0.23026466308045	4.65429131762265	-9.69678076461898
C	0.34575891872946	3.38955290109451	-9.55269421122715
C	-0.19248605040240	2.50777907281393	-8.62358143897472
C	-1.29654693269035	2.84042200626051	-7.85343561346810
C	-1.91067945937015	4.08144660006701	-8.04719993169118
C	-1.36271453876588	4.96978804269388	-8.96227122550693
O	1.42728239450056	3.10495567867124	-10.34727086791781
O	-3.03079866191831	4.33691645945520	-7.29660331640992
C	2.10930148836958	1.89120522255135	-10.14497952190107
C	-3.74595584463831	5.51958557724380	-7.55715940063014
O	-0.32044721265135	10.55589701431290	-9.92633954783392
O	2.03794918794021	5.78383549954757	-8.39084978176056
C	2.81535142913388	5.72282092248718	-7.22088280148427
C	-1.12730982235532	10.60631549065959	-11.07851767292114
O	-2.24548992604438	10.60746798687047	-4.38076951865116
C	-3.16785854227303	10.91508447426182	-5.39870605599522
O	3.14906554406935	10.12815177776145	-5.56973811262255
C	4.07295542159486	9.86789368368185	-4.54008804440217
O	1.51852135376333	8.82493127866300	-0.72434152731589
C	2.54707772640219	8.26929039352903	0.05883858827252
O	-2.41957833590988	5.57155909484380	-2.86174460241808
C	-3.45981174078355	6.13187974168614	-3.62318752836256
C	8.83951797075051	-2.23078063657627	1.23892935179512
C	10.07358744877505	-1.57484233780306	1.23709979517413
C	11.18686844946891	-2.22321884842840	1.75643514824015
C	11.10437502952603	-3.50428761621223	2.27767220461412
C	9.88190921059942	-4.18174562295250	2.22970269945205
C	8.76881578717303	-3.53081728828466	1.71575119929368
C	12.31697217409109	-4.14724201440298	2.89701129371186
C	12.92311095457149	-3.31891605385157	4.00135704934458
C	12.12335834713171	-2.75795937949406	5.00387500228208
C	12.72654774261353	-2.04051661293490	6.02674241104576
C	14.09983491224877	-1.86600682299847	6.09664857805939
C	14.90100707915585	-2.42662945538392	5.09858047973886
C	14.29633989114851	-3.13755839430223	4.06670583321954
C	14.70119429349232	-1.07623647870114	7.23838054944184

C	13.93277595123373	0.20081401363234	7.43334254055474
C	14.19252870679822	1.30251083431245	6.61257390030456
C	13.37044703083079	2.41846082281684	6.69425373458626
C	12.28513360964616	2.45813484877390	7.55340375687760
C	12.06510899020941	1.38516882004751	8.42273900866771
C	12.89613070334683	0.27459385962224	8.35219731863937
C	11.29614635541447	3.59247855331911	7.49641686334205
C	9.98406770556752	3.08773147177047	6.94719763550438
C	9.92609784350486	2.49605089066494	5.68125465366643
C	8.72147458829132	1.98370840792394	5.21627178443392
C	7.55965362534713	2.06863027302334	5.96271757448902
C	7.61306752920580	2.67519048150762	7.22329408715017
C	8.82152469426098	3.16732974314014	7.69760107394625
O	11.08771376292171	2.47432961509221	4.95237382674123
O	6.42694696053962	2.73240396152519	7.91459772535774
C	11.10668623950874	1.77376503769862	3.73441543218638
C	6.43182087087269	3.28587379121767	9.20901076680201
O	15.25610553324686	1.18691356159205	5.75413291758668
O	11.00947274918779	1.51067990579278	9.29207753425421
C	10.70175985883462	0.42985226901897	10.13749619400668
C	15.61805303923954	2.30254030400821	4.97802251991486
O	10.77106704676897	-2.96552923714171	4.91419810331482
C	9.92798586059480	-2.34794876784335	5.85365516514544
O	16.25568009629333	-2.24483696738249	5.21572954750050
C	17.09301586518248	-2.73428582230594	4.19602733794588
O	9.86608995793128	-5.46903953877895	2.70517379244223
C	8.63468428225552	-6.14645036086222	2.77248053654943
O	10.09704405237390	-0.30594539598730	0.70864970225339
C	11.34492747474503	0.23839861376576	0.34895471630763
C	-2.10729096348247	-6.96208013450618	3.54332313109368
C	7.59836540540971	-1.51883990009125	0.76948506650857
C	-0.20568371076234	4.28135595145173	-1.67307989642479
C	6.22647786735637	1.53833070882819	5.47831025693497
C	-1.77460999025457	1.91720600718037	-6.76761904059253
C	-4.17738848948568	-1.59235837927819	2.92502272123207
H	6.52661961461128	2.45689591149203	2.96993930576034
H	5.55921358912731	2.39609643463642	5.34038343428118
H	5.81179594074651	0.93081477613818	6.28477227088102
H	6.69626406597514	-2.42209539512503	3.10667233641074
H	6.86682769126828	-2.25756109992355	0.43166514664716
H	7.85109229208550	-0.86018594140391	-0.06141341712753
H	-5.73454293399442	-8.75193191690433	5.89398508238151
H	-1.23437873637724	-6.80658860668923	6.05842994742895
H	-4.12381687564994	-8.75134758232412	9.18909913590248
H	-5.36146379671859	-9.55988203699247	8.19805017466383
H	-9.21628073932977	-6.99770255422517	8.92912517439855
H	-4.41316636827175	-6.31591506735732	9.63551998360671
H	-8.37562510825554	-4.09938678033838	10.97974643648624
H	-9.68394561853002	-5.20464365112684	10.48407781034968
H	-11.01484716582145	-3.63013429129719	6.20318999517145
H	-7.51418465939908	-2.47437306052781	9.43999802256306
H	-9.79246615395291	-0.56842034046904	5.45232167002522
H	-10.22800389053064	-2.10659992815831	4.68238371617113

H	-5.51380211028022	-3.68236791416211	4.03052505163450
H	-8.19385180428708	0.42298351028934	4.13794629199761
H	-7.84042039354049	-5.84699063652652	5.69219171802688
H	-6.32728091481100	-4.93758773979501	5.87569183550756
H	-6.83782907007837	-5.49420354831682	4.25735322065006
H	-5.83982397548998	2.57050795724330	2.41897001181179
H	-6.71919897213328	2.21010612521286	3.92616363468456
H	-7.41395218090552	1.73805969996746	2.34823977435258
H	-6.07671918110165	0.29192200688079	7.59830771728935
H	-5.88352494501234	-1.39602413144749	8.13432583378576
H	-6.87592156900280	-0.25929263490475	9.09333082041952
H	-12.67224199252011	-4.73890437928640	7.43491018982982
H	-12.43958329460589	-6.41408934326907	7.99313445248120
H	-11.57926219042528	-5.83627207790062	6.54273845027303
H	-9.44433333001019	-8.41966093398209	7.12461242271644
H	-9.50980840197409	-9.30182612318613	8.67694308689892
H	-9.01575464533722	-10.14875309257833	7.18930002280467
H	-4.09445876268110	-4.02402787097484	9.83351553980111
H	-4.20920264937383	-4.83625906580219	11.42090450929768
H	-4.60984119666948	-3.10667891875150	11.27268819953225
H	-0.88633321302534	-5.95948819631375	8.17098141254255
H	-0.28815840221463	-7.63667628470742	8.03681376854245
H	-0.75720192058928	-6.98092295197736	9.62512859964824
H	-6.09895425163297	-8.79657200374690	2.29390170796140
H	-6.70637060031281	-8.24773257824066	3.87565163496598
H	-5.80427952630738	-9.78411118486622	3.74893882686807
H	-6.17937641025548	-2.65521508300267	1.15103906565891
H	-2.52239876724092	-4.34385644972031	-0.25594280032707
H	-2.71830686824845	-3.07332447179847	-2.31701484362227
H	-6.40749633350248	-1.41023874311518	-0.93291240712604
H	-3.28918989509907	-2.18200435244291	-4.21535871946181
H	-2.17885798384196	-0.29889410665475	-5.26171989432631
H	-4.41677973928612	2.38673610698485	-2.81171101734807
H	-5.42788306112157	0.49605339489267	-1.66506683614060
H	0.54289161492730	3.10761460108672	-6.15406376907451
H	-2.29933638906045	2.81158145186792	-2.21686609440828
H	2.19541987634102	1.87534329508058	-5.00847981961820
H	4.38567882721376	1.41973798775476	-4.03890727516064
H	4.11464369933462	4.99886024157649	-1.73820662090712
H	1.98650309837509	5.53276097901626	-2.84114148409027
H	4.29773608801671	0.60752118307847	-1.94372744864870
H	5.34395757005875	-0.41519150215464	-0.01671817632217
H	7.49715751734660	3.19298035470990	0.75130406395411
H	6.42785163524071	4.23513067915914	-1.18587517431895
H	6.39368298647918	-3.24612920028009	5.21784912443018
H	4.46034254558583	-4.60944090831475	5.82716346371183
H	2.02223730871628	-1.10656189858499	5.92282357498606
H	3.91727480592890	0.24061756184745	5.22030522235762
H	2.55998000642537	-4.83828970457835	4.06315599910739
H	0.35562586155255	-5.46264520843901	3.23490783250061
H	-1.45647383181368	-3.51025193206107	6.57568431646642
H	0.76065241041630	-2.81726116777037	7.36536456340881
H	-3.60828579644237	-6.00241597256365	1.52200496836079

H	-2.29700319068560	-2.43769406712150	4.59691898850854
H	-2.27653308257687	8.26565013487538	-3.01834503438867
H	1.41265472622212	6.12021491679493	-0.61376645810430
H	0.16181216318979	10.57511147822748	-1.54753644057971
H	-1.51201584822887	10.44715612488076	-2.14457364156879
H	-1.08418923875079	10.84521864178267	-6.82851744343840
H	1.99408353767527	9.96204910266506	-3.11488440420855
H	2.48264571682810	10.81091164376490	-7.71013048077158
H	0.90643790074602	11.46635452460949	-8.20359120665336
H	-0.61595327411410	8.13657893330875	-11.11499413750736
H	2.35855889128933	8.21327817310340	-7.21572687280600
H	1.32924001225572	5.29892250374228	-10.97738833004016
H	-0.27577929742830	5.88094499065754	-11.45422496409994
H	0.25608265002546	1.53789488019556	-8.46976908450304
H	-1.79023306522756	5.95047939083428	-9.10165451257449
H	1.47290415009307	1.02800071093530	-10.36848281224196
H	2.49181295569353	1.80948912560126	-9.12221195259872
H	2.94729347500307	1.90732555236559	-10.84112444289389
H	-4.62558686636802	5.47978098172176	-6.91588845148023
H	-4.06373418164376	5.57362119492843	-8.60399957135644
H	-3.15736995987206	6.41025243607577	-7.31154459007972
H	3.11402592870625	4.68005162935687	-7.11888038137620
H	2.23839411255004	6.01945538118326	-6.33841364090396
H	3.71225178865554	6.34684059541309	-7.29705677955645
H	-2.01954944568563	9.97950747478555	-10.97518765868417
H	-0.57166930710858	10.30712617733558	-11.97305952165323
H	-1.43065587447154	11.64829466393323	-11.17570048357682
H	-4.13956867169484	10.96198265142655	-4.90783802152215
H	-3.18943803480087	10.13866997181393	-6.17100707204572
H	-2.94914027399911	11.88267296847478	-5.86145741775150
H	5.04375175577933	9.79885105219113	-5.03012923857492
H	3.85430995142308	8.92130925218173	-4.03415127716145
H	4.09553782327470	10.67831255941659	-3.80449099177102
H	3.19234692301650	7.61034980032845	-0.53186911039236
H	2.14928471389918	7.71704659426624	0.91623172766245
H	3.13185517853626	9.11634630171401	0.41635079933991
H	-4.04477521360271	5.28516617351534	-3.98002845777382
H	-3.07322718332519	6.68994292666864	-4.48102037705002
H	-4.09582734822300	6.78781270661090	-3.01803243016212
H	12.14438636624116	-1.72624213601408	1.79224031841122
H	7.81562040687915	-4.03503968761997	1.67216424680207
H	13.07203568535882	-4.32808310470212	2.12723934347428
H	12.01394151176312	-5.11303661658665	3.30739648499292
H	12.12399453979979	-1.59386046241950	6.80040812254985
H	14.89935486889907	-3.57805663313005	3.28757714184959
H	15.74048438313130	-0.85683891300499	6.99825087442169
H	14.66848949494105	-1.67850321220193	8.15032920977047
H	13.53104903057095	3.26496346118111	6.04645631692963
H	12.73506689522080	-0.57117222697222	9.00357430720672
H	11.68652281616717	4.37959883486395	6.84802746880430
H	11.13433530036510	3.99867195309183	8.49622151556226
H	8.67785799086347	1.50530545639533	4.25201354722430
H	8.88221397720851	3.61992972901798	8.67429324541169

H	12.13345831147565	1.84624871440037	3.37739473165844
H	10.43342089811283	2.22009556214642	2.99469234564661
H	10.84741200823733	0.71789578293496	3.86706944286408
H	5.40713158035513	3.19672638525599	9.56829273995067
H	6.71906774429853	4.34244642151244	9.19433494265349
H	7.10040433314228	2.73577223142372	9.87906530496321
H	9.83245208093858	0.74641168306687	10.71291659779658
H	11.52484704984352	0.20449027192870	10.82410055829957
H	10.44523825901176	-0.46874322901267	9.56596711253099
H	16.50303099373118	1.99522972768301	4.42160049887444
H	15.86562610714883	3.16586304209028	5.60490751607512
H	14.82761454882224	2.58126676655956	4.27351761105747
H	8.91393884796552	-2.57468269205915	5.52874563627320
H	10.05764234187505	-1.26043727343613	5.87312899481990
H	10.08361555703063	-2.74920669981058	6.86146098458422
H	17.04129251448543	-3.82575916554264	4.12044250783522
H	18.10253566475421	-2.44259944253681	4.48358939942673
H	16.85052950103985	-2.28804849522000	3.22597861329616
H	8.85689279821936	-7.11254208011433	3.22461257062453
H	8.20541323880560	-6.30595539427525	1.77739204267869
H	7.91440065845263	-5.61176812086506	3.40037592114870
H	11.11855003676141	1.16977553296146	-0.16908018928047
H	11.89050229531891	-0.43076878877653	-0.32464716664299
H	11.96467462823276	0.45515626758746	1.22404148742904
H	-2.42443589800577	-7.59228089746350	2.71202198435722
H	-1.03608653017127	-7.08467830675660	3.69919506351562
H	0.80310956121210	4.03627700801182	-1.33158661967598
H	-0.91415369294247	3.90399283511097	-0.92933501227857
H	-1.37279998545636	0.91715497250401	-6.95409465030726
H	-2.86466326579018	1.85951339141958	-6.77673679063807
H	-3.58213607467418	-0.88254517265368	3.50698043842867
H	-4.23871684190156	-1.19572974524453	1.90801532331994

Table S 8: XYZ coordinates of the optimized geometry (GFN2-xTB) of **all-p*R*-[12]CPP-PA$_4$**.

C	-4.28370635331849	-6.49293867938662	7.74285326853204
C	-5.44870553357616	-7.16065398898869	7.35698750579262
C	-6.06709752490578	-8.01303841100478	8.26122929829779
C	-5.58084102247958	-8.19657550176398	9.54596194167237
C	-4.43390567969210	-7.50256241340305	9.94297811527278
C	-3.79846811058775	-6.67232359462039	9.02893340745127
C	-6.30828879601780	-9.12999489447049	10.48341125830904
C	-7.64948604674420	-8.55516524001413	10.86197266365259
C	-8.80249293304711	-8.87190965380266	10.13747483211208
C	-10.00583446238093	-8.26648387059733	10.47498938056461
C	-10.09983585536135	-7.36638771053732	11.52294549914030
C	-8.95674103101722	-7.08780106630077	12.28081218726318
C	-7.74916969575267	-7.67687652895664	11.92972859437168
C	-11.40303204644676	-6.66913232046242	11.82006562888617
C	-11.29207169583845	-5.21224830644934	11.44918799888061
C	-11.16827191874090	-4.83307169385849	10.10731253448228

C	-10.96688538497775	-3.49525514254456	9.79742615103850
C	-10.89273395646573	-2.52184805270961	10.78305125851649
C	-11.06039785389529	-2.89535639057062	12.11994983758362
C	-11.25766123519969	-4.23582112813473	12.43017176938860
C	-10.54904604415209	-1.10856834668855	10.40985929161877
C	-9.06946143556333	-0.93112697521061	10.16011081871715
C	-8.61423571730228	0.20312809668940	9.47976243903067
C	-7.25529364625560	0.35236021863640	9.23410867044759
C	-6.33741157039368	-0.60352580566694	9.63821891177706
C	-6.79095375517185	-1.72631728145605	10.33737149100169
C	-8.14717263453211	-1.87088942903660	10.58963939039201
O	-9.57624695020721	1.09911716176286	9.08391413816299
O	-5.83598828269024	-2.63111915283658	10.72957650571390
C	-9.18119996966106	2.21320307842994	8.31920508901125
C	-6.25956646755366	-3.84411789027991	11.29755792814372
O	-11.25974241915126	-5.83587409746635	9.17450773556376
O	-10.99803743241155	-1.89012042566842	13.05301658819447
C	-11.15541350379137	-2.22418318133175	14.41103820344124
C	-11.06259823685787	-5.51208485283693	7.82023390396697
O	-8.66417353443190	-9.78050429501022	9.11357594823319
C	-9.82105632285986	-10.21886116158149	8.44176927384522
O	-9.11591114968158	-6.24042646453672	13.34916043554303
C	-8.05185100827275	-6.11107524274253	14.26216492834846
O	-3.99824794531658	-7.69854554358221	11.23256024122585
C	-2.78717411905926	-7.09940963717020	11.62860820948485
O	-5.90642172758041	-6.93400265743214	6.08094867407458
C	-7.08607712230288	-7.58336330584233	5.67058272335631
C	-7.63275361359643	-1.81885423211271	6.58721394380271
C	-6.31586503896507	-1.45084023633713	6.83713008997898
C	-5.82551654038169	-0.30212442267001	6.21510392869153
C	-6.60312267064047	0.41838087892046	5.34070639296826
C	-7.91114189043123	0.02467217971834	5.04604716783392
C	-8.42064404585411	-1.08854889817185	5.71134033620146
C	-8.56706132513239	0.71994922929777	3.92950622123801
C	-9.25807931166416	0.02363240196991	2.93920489765361
C	-9.50108770926622	0.59599868514106	1.70266475592209
C	-9.03868754214443	1.87910472866041	1.41024552596955
C	-8.53752395603913	2.63992665629402	2.47006985448748
C	-8.31551249891667	2.07624447107949	3.70621187988746
C	-8.83217989541736	2.41787336982426	0.05533187071242
C	-8.16360811846940	1.66447260238348	-0.92238016110738
C	-7.61880117306471	2.33413281725324	-2.00567833032828
C	-7.73448710738336	3.70860725858359	-2.18358821015632
C	-8.56533250938351	4.42319779424826	-1.31410659653957
C	-9.07753269428325	3.76672293036885	-0.20717038155379
C	-6.79955080062824	4.30342768533847	-3.15609273638553
C	-6.67254240027732	3.82291246679060	-4.45757596577100
C	-5.56527322106378	4.13625944077503	-5.22425285031790
C	-4.54547165271419	4.93740539800363	-4.71237049243328
C	-4.73303543575962	5.51126104745401	-3.45427989068997
C	-5.83589203553808	5.19630947812353	-2.68873074766945
C	-3.21384633221314	5.02367374290986	-5.32847506068388
C	-2.62165938020433	3.85929914676013	-5.81830734692040

C	-1.27046416206382	3.81363549849622	-6.09205194331576
C	-0.45983112546172	4.93097748805955	-5.88849210918222
C	-1.07592621696519	6.12724517157211	-5.52617890600155
C	-2.42907675076500	6.17373748179325	-5.25258953173968
C	0.99734655326865	4.74134180547458	-5.81780413773148
C	1.75545657276143	4.03677228020528	-6.75871619080346
C	3.00022605822823	3.56146903725085	-6.38056057092826
C	3.55983520256506	3.82363144003791	-5.12795682127737
C	2.90362879757796	4.74831120677621	-4.29814678886487
C	1.62661578422558	5.15241999188789	-4.64827561065891
C	4.63649811587684	2.96161556005068	-4.60558349886803
C	4.43933347249617	1.58336864791812	-4.73877985326157
C	5.15310082801542	0.68236266025450	-3.98093346536795
C	6.08856231995123	1.12124621886566	-3.04100624923132
C	6.42007004199517	2.47454542068596	-3.05030368852609
C	5.71520778634270	3.37899878593384	-3.82555427362138
C	6.51651295069459	0.23564535907088	-1.94924926757775
C	5.54547131981786	-0.60993831334794	-1.40731517874253
C	5.77918788365189	-1.30568957020797	-0.24535194317239
C	6.99642840484688	-1.19333492340528	0.42786199007643
C	7.98736289040171	-0.40094713766203	-0.13810868393390
C	7.75274093554026	0.30016128030541	-1.31094899076095
C	7.01964436314947	-1.76057428988709	1.78916887590490
C	7.12948402349194	-0.87999422864248	2.86055108723732
C	6.66793650005356	-1.20091411824552	4.12871861961683
C	6.06700380369183	-2.44844354719180	4.32156958269257
C	6.17791026023879	-3.40272367613525	3.30922109241909
C	6.64886524121575	-3.08705433748666	2.04731775470732
C	5.07061272087405	-2.70053009544154	5.37687098156914
C	4.94933529609885	-3.88858104374375	6.08949620267206
C	3.79697500917957	-4.17550507373072	6.80710612678868
C	2.73130982310849	-3.28558155107454	6.81140297769769
C	2.89046442214427	-2.05463035781913	6.17502548868926
C	4.03880381939797	-1.76429197257761	5.47605462644498
C	1.35725117693758	-3.59064189068864	7.26578437127894
C	0.40909830132234	-3.68357230260017	6.24624893494919
C	-0.93338166705705	-3.74546950947427	6.53443749873355
C	-1.38368404122598	-3.70473115494869	7.85679759883255
C	-0.43532845003503	-3.69357322576349	8.87368794878264
C	0.92147070684540	-3.64577286558092	8.58228426963569
C	-2.82557770262904	-3.44753993397397	8.02257021284648
C	-3.79162089468425	-4.14576828152992	7.28868233574170
C	-5.00616888741602	-3.52798283277598	7.03652142683746
C	-5.30055822358206	-2.26311137412416	7.53114398510197
C	-4.45024617194429	-1.70766840360952	8.49708509301534
C	-3.21624179193828	-2.29588130283300	8.70516591036605
C	4.31665862245388	6.57461953135927	-3.39573792474037
C	4.38614753519419	7.64213646449535	-2.49506564160525
C	5.13577038947728	8.76375413980024	-2.82692160447416
C	5.84280904142915	8.85374060038556	-4.01704175712599
C	5.78426273676711	7.77956754226548	-4.91063090279169
C	5.00799293003466	6.67282974684105	-4.59262658834427
C	6.68511350144448	10.07627905819975	-4.29235676611024

C	6.62134175563979	10.54268885851772	-5.72029958062335
C	5.40493834574982	10.95730961820668	-6.27235887455699
C	5.35957593983833	11.33225769713101	-7.60824515979100
C	6.49356517017419	11.31957829704963	-8.40646621120318
C	7.71575431709487	10.94466912119955	-7.84251362189841
C	7.76014393847997	10.56694263824424	-6.50725012324292
C	6.37967821594461	11.63174074138929	-9.87834161983205
C	5.90109618307673	10.38861922956438	-10.58644434640756
C	4.60133983584925	10.28533311040035	-11.09023778898801
C	4.18491362062157	9.07833635038198	-11.63809388654731
C	5.01357875170560	7.96530323375621	-11.67394885465586
C	6.32473129799665	8.08173679003722	-11.20174879424370
C	6.74777412712763	9.29485383654595	-10.67595812340667
C	4.47938428907533	6.64263842485441	-12.14043581795502
C	3.68307431210817	5.92076531321575	-11.07624554876875
C	3.35460066230734	4.57348225760895	-11.26392477832964
C	2.59842643495919	3.91461633962737	-10.30231998397088
C	2.13686030409855	4.55977791537184	-9.16383849524497
C	2.46424010879407	5.90742710574226	-8.97927232350125
C	3.23923834609000	6.55776346564461	-9.93067682298602
O	3.81227341173543	3.99583140177400	-12.42178246625263
O	1.97721359182650	6.52132231450775	-7.85475980740423
C	3.53747568010121	2.63405439650863	-12.65081466427335
C	2.31274303271101	7.86329215624029	-7.61129952262834
O	3.80386398153599	11.40156831319961	-10.99179643695875
O	7.11610584828727	6.96306096206032	-11.28459851989452
C	8.48160349102189	7.08602752204542	-10.96516926389558
C	2.51140623064745	11.35564487778154	-11.54735664135472
O	4.32368887149484	10.96019695752388	-5.42724035569541
C	3.10574539105461	11.48274879504713	-5.89961529364911
O	8.81499161659630	10.96613576116075	-8.67083049291895
C	10.07487985297680	10.65667968481836	-8.12482010050124
O	6.51970360961841	7.87673557724450	-6.06496331263101
C	6.48613555528537	6.81389017537573	-6.98437772825787
O	3.68912053099785	7.49930345291519	-1.31913493062987
C	3.70316381903452	8.56095496063185	-0.39478600759861
C	8.20502548291076	-4.40081083831218	0.65374775529807
C	8.71321921504475	-4.16561970833976	-0.62602045916741
C	10.06181930704455	-4.40344616022879	-0.87013605690114
C	10.91211110931632	-4.86492859188862	0.12151061100142
C	10.40067028866138	-5.09520146156703	1.40445060587575
C	9.05547382987861	-4.86100669107053	1.64828332209130
C	12.37180086226973	-5.09224335385788	-0.14875610138368
C	13.24328523875587	-4.05397756227805	0.51533293968508
C	14.52774505967291	-4.38812874732775	0.95366740196825
C	15.30579520936840	-3.42155373458830	1.58129754361344
C	14.84413075157329	-2.12930653778720	1.77260911548767
C	13.56677459999953	-1.79088619405178	1.31158581299530
C	12.78770566763045	-2.75712435028510	0.69161452862517
C	15.66224482161708	-1.10119936587574	2.50838867077071
C	14.89109477301420	-0.60181468458041	3.70464869261698
C	14.64637397747520	0.75968201816612	3.89739882705254
C	13.82175573205752	1.15540118435712	4.94323709406823

C	13.24806074228590	0.23771556734273	5.80764873201673
C	13.53943110452427	-1.12062955262922	5.64698720639155
C	14.35336334233989	-1.51752040704497	4.59579477254550
C	12.29133972508122	0.69508016606892	6.87934488777776
C	10.86741404398994	0.47093989129213	6.43477766618140
C	10.23421216702408	1.37946138168166	5.58035650496407
C	8.91893219661218	1.14778588044442	5.19646640006172
C	8.21884752852439	0.03389196879200	5.63198254693434
C	8.85960135287809	-0.88528247747377	6.46887573825026
C	10.17031670682011	-0.64794930819457	6.86136359705334
O	10.96460251972334	2.47705747279499	5.19582193236794
O	8.11522805124670	-1.96513575507224	6.86839581975532
C	10.35986055452119	3.42437117698538	4.34969406725442
C	8.69725830436460	-2.89107761130056	7.75321751112836
O	15.24546084347987	1.62852208538786	3.02009370105312
O	12.98649054263200	-1.98648035883692	6.56018822121091
C	13.27858616632656	-3.35772718765854	6.43980370361353
C	14.90247815359830	2.99146153963000	3.09039658357396
O	14.93453934070212	-5.68032689131035	0.72833678130775
C	16.19307655130435	-6.08457979693960	1.21259933341098
O	13.17093712390255	-0.49385230567861	1.50935030151934
C	11.83162490865169	-0.15429421483990	1.25944296335439
O	11.29027199232259	-5.55165949020943	2.34209827483266
C	10.81153099839888	-5.83070133270720	3.63548226792972
O	7.82579691429541	-3.71894405421452	-1.57348183536130
C	8.31598097351321	-3.36474362987319	-2.84325115125140
C	-9.89905857922465	5.91282423737070	-2.74545669432818
C	-11.26584883016709	5.69080617086281	-2.54627178136661
C	-12.12568432458795	5.74537604838288	-3.63496153571213
C	-11.66229202954978	5.99726204672481	-4.91673444850016
C	-10.29314711832990	6.19398829521702	-5.11920095819870
C	-9.43530878460421	6.16007609329982	-4.02735742253519
C	-12.63624158683118	6.04661850826254	-6.06629707527676
C	-12.97184438008784	4.65630847187145	-6.54453307467874
C	-14.16487238849603	4.02922297395977	-6.17438725309150
C	-14.44083307859419	2.75948052212582	-6.66359660528726
C	-13.55204215178048	2.08508858817738	-7.48552318792327
C	-12.34121893749076	2.69693915953608	-7.82401911009567
C	-12.07808140156451	3.97833721954827	-7.35848628422283
C	-13.88376679134222	0.68924180513754	-7.95234919191395
C	-13.54598123143309	-0.27881853229241	-6.84708956642229
C	-14.50748640707430	-0.67636518293626	-5.91459190855184
C	-14.12570387200143	-1.47841923808131	-4.84704619197533
C	-12.81288291732118	-1.88425193411323	-4.67646859428717
C	-11.86095316928436	-1.52761694925934	-5.63755779964701
C	-12.24216447457404	-0.72657892328469	-6.70432307245608
C	-12.39081600483943	-2.64050070753999	-3.44468303296083
C	-11.28594801747922	-1.91295898406327	-2.71979024896714
C	-11.38642413643067	-0.54275506346668	-2.45831806909969
C	-10.32229305177329	0.11658910951994	-1.85781113791516
C	-9.16616756947349	-0.54920984405069	-1.48638175189005
C	-9.08124981050405	-1.92712986575895	-1.71114798603646
C	-10.13794899412225	-2.58430594057640	-2.32721640208262

O	-12.55671783331294	0.08065696097405	-2.81398713794757
O	-7.91678229302185	-2.53224801280275	-1.30555988992674
C	-12.60983607803382	1.48499327483669	-2.78183307928670
C	-7.76481102789174	-3.91388054186335	-1.52878985183789
O	-15.79078951979494	-0.22029758302059	-6.11304587808127
O	-10.58788750591812	-2.00322606223947	-5.44555893172742
C	-9.56811507025000	-1.54949799237464	-6.30096640806243
C	-16.79391713415514	-0.62306404293047	-5.21164880628251
O	-15.00537319722446	4.73078827903907	-5.34177042795841
C	-16.25153119682966	4.16242197897136	-5.01572220509885
O	-11.48211824580287	1.97858500598220	-8.62171815618705
C	-10.27648735438706	2.58542845416109	-9.02160762233622
O	-9.88198589529495	6.42607010981475	-6.41011143480004
C	-8.52443225438190	6.71983285548238	-6.64146436945363
O	-11.66782911747676	5.45059686902078	-1.25675028230211
C	-13.04683290600191	5.34896516538955	-0.99270138886521
C	-3.56201701943585	-5.55472599749013	6.80548878461698
C	6.75390655374628	-4.14549170707895	0.97974562728748
C	3.50711688432510	5.34094444944466	-3.05849125542203
C	-8.93624462545895	5.85967278891980	-1.58577044534270
C	-7.99381232039792	0.17526788806860	-0.86428748610480
C	6.78023901182017	-0.18505088729495	5.24006521184295
C	1.26639758705594	3.83010611945500	-8.16848116222720
C	-4.87757077927374	-0.49491874167173	9.28859362933753
H	-6.94963287095622	-8.56501699797040	7.98573580985782
H	-2.90262616669908	-6.13677076797200	9.30132756879819
H	-5.70251863790106	-9.27935210554878	11.37650107783725
H	-6.46204665191001	-10.08885194219633	9.98508459887199
H	-10.90127781851339	-8.47716895802299	9.91335026888782
H	-6.84892916090045	-7.45688957476775	12.48133419143994
H	-11.62942974915499	-6.75340513789309	12.88424125777027
H	-12.20221333409530	-7.13565566753895	11.24172295167748
H	-10.83717569698622	-3.17846266628777	8.77429035025055
H	-11.36035627811734	-4.55108614666414	13.45561668293402
H	-11.08453925503618	-0.80945428790647	9.50545797618666
H	-10.85301370506773	-0.43263141848803	11.21224232549837
H	-6.88973985858428	1.21344918502122	8.69692153990361
H	-8.51213368619200	-2.73691142973068	11.11705411339902
H	-8.51369850068180	2.87265031385372	8.88380455002056
H	-10.10121378253477	2.74804503149691	8.08585335608016
H	-8.69348558671602	1.90712968422216	7.38846742490497
H	-5.35138785956654	-4.41948037094501	11.47121667046412
H	-6.91268149629711	-4.40675400956144	10.62121214242705
H	-6.77808169777384	-3.68237855035020	12.24886218668822
H	-12.13912378791057	-2.66360736394803	14.60684620812192
H	-11.06608275596583	-1.28381004098245	14.95398194135955
H	-10.37419700846196	-2.91293043010260	14.74868003078590
H	-10.06184620697492	-5.10222913240547	7.64641156536629
H	-11.81477909384009	-4.80315912642087	7.45834965006408
H	-11.16652969113228	-6.44998652003101	7.27535133765010
H	-10.30710431257163	-9.40153033600530	7.89891290302581
H	-10.53823218177524	-10.67492469437680	9.13139822813481
H	-9.47802689104400	-10.96927997115810	7.72985323676913

H	-8.43186453201525	-5.48532366274084	15.06936494856395
H	-7.18157082211928	-5.62615112991223	13.80898780369719
H	-7.75326336433214	-7.08317176277432	14.66809269498671
H	-2.84508670461697	-6.00638249303643	11.59556139440055
H	-1.95016534598353	-7.43667084781922	11.00873474424582
H	-2.62605233489583	-7.42010730334871	12.65744555052904
H	-7.27067593650210	-7.23771546899846	4.65377436581133
H	-7.93522254502579	-7.31189605332963	6.30676797827664
H	-6.96685709563599	-8.67166412094737	5.66779965554518
H	-8.04145788601259	-2.69132105172712	7.07822712473526
H	-4.79688941118199	-0.01206514326225	6.37600549278674
H	-6.17157991156201	1.26394039403670	4.82508449573216
H	-9.44082051679630	-1.39781490636755	5.52982981444399
H	-9.53544861003234	-1.00767958242372	3.10610702318343
H	-10.01833531601794	0.02364915405777	0.94659065000876
H	-8.24218540339016	3.66432720420220	2.29308928842324
H	-7.88805608432794	2.68053452591727	4.49383816693504
H	-7.02226528482138	1.77329239320515	-2.71256499983430
H	-9.67944232505063	4.32805190793212	0.49348870924772
H	-7.43504250212458	3.16619804271668	-4.85464081561166
H	-5.47189253093152	3.72579359147566	-6.22040164290106
H	-3.96262897277379	6.14477899295076	-3.03880223110119
H	-5.91139699511225	5.58102923185938	-1.68134229199713
H	-3.21201564145017	2.95828686921836	-5.90578794312600
H	-0.81876808039825	2.87774841756800	-6.38923132605594
H	-0.47839873379476	7.02134722279073	-5.42169057589566
H	-2.87872473793840	7.10685570339500	-4.94213323697364
H	3.55893394393980	2.95467225102527	-7.08155535504132
H	1.07441500375079	5.77160372446930	-3.95461216758339
H	3.65591078996208	1.22400958559708	-5.39089738289917
H	4.94839561846439	-0.37429039138709	-4.07971559594666
H	7.18797583188079	2.84053336503802	-2.38320781115738
H	5.99237235704386	4.42183895357297	-3.79090262071313
H	4.57029873318800	-0.66697009022600	-1.86835491771402
H	4.98564844813206	-1.89686186476306	0.18976533190945
H	8.94475942073240	-0.31315944115159	0.35677319483130
H	8.53677854547726	0.91998827956126	-1.72439871244398
H	5.74902133209071	-4.61516045915085	6.05268007701853
H	3.70270105928541	-5.12522004020460	7.31597079500830
H	2.07604534340642	-1.34338217765886	6.19440367214221
H	4.11051245840778	-0.83416792773813	4.93131169942838
H	0.73972602666037	-3.66526222912558	5.21688612223341
H	-1.65469489455457	-3.75484461331156	5.72993324895704
H	-0.75787291334479	-3.68938728131766	9.90608679023133
H	1.64189206183625	-3.59726206649691	9.38735754997344
H	-5.70962024405850	-4.00960274582245	6.37149655019188
H	-2.50324896838082	-1.80948774372370	9.35735686764330
H	5.18682757189548	9.60056011161092	-2.14805541574879
H	4.92617788024043	5.85955703435476	-5.29478125200019
H	6.34442421878269	10.88246952179278	-3.63751897530295
H	7.72526403545235	9.84823490301713	-4.04071716158929
H	4.43031971127400	11.63317912031648	-8.06592018050780
H	8.68984222964903	10.25331724784149	-6.05946100175922

H	5.66679172517302	12.43935066521446	-10.04015336865842
H	7.35737944105418	11.92522360461960	-10.26160140477641
H	3.18382611360599	8.96773248246997	-12.02605639070786
H	7.74835330788430	9.41385953422759	-10.29488664000491
H	5.30464221078751	5.99796797982372	-12.44609589656282
H	3.83110372921967	6.78343566467919	-13.01120069554958
H	2.34068455945889	2.87499508388563	-10.43471911310772
H	3.50742457205983	7.59114513307070	-9.78791981248216
H	2.46076677503965	2.44610285387617	-12.71857081940615
H	3.97199705015587	1.99860182065384	-11.87217161905089
H	4.00359432261057	2.40006041296110	-13.60719346734980
H	1.87818834014977	8.09997086474984	-6.64088238233372
H	1.89030493086314	8.52774343139481	-8.37398450148160
H	3.39562039628013	8.01890467210294	-7.55891909964679
H	8.91743848730249	6.10807309519551	-11.16654430643584
H	8.63209537581645	7.33612164072946	-9.90987291339370
H	8.97286899077786	7.83987049245840	-11.58884792254618
H	1.88214005837726	10.61100345280093	-11.04776593467610
H	2.54129396159485	11.14512945651767	-12.62115218932399
H	2.08958684216262	12.34736570983252	-11.38692049065902
H	2.41643335750474	11.42085062884028	-5.05824065943039
H	2.70829396580133	10.89741742300050	-6.73454643982437
H	3.21145730551856	12.52872458835156	-6.20724899429832
H	10.78338817256309	10.76739425778707	-8.94539412273676
H	10.11167058883607	9.62748557287614	-7.75181897953115
H	10.34503878704396	11.34627771225948	-7.31869203294679
H	7.09291117751252	7.14240249571814	-7.82664845007419
H	5.47344103873845	6.60354911831850	-7.34247110334999
H	6.91384328054498	5.89786239515876	-6.56157438769549
H	4.71539087595887	8.76334682509444	-0.02962986918331
H	3.08104765041364	8.23091839023944	0.43660073723045
H	3.27958792501809	9.47494829508760	-0.82381234317360
H	10.47673226581351	-4.23104192378845	-1.85134090401704
H	8.63555554375765	-5.02487763678468	2.62824100520923
H	12.55826872351014	-5.08078018413709	-1.22645386489342
H	12.66787637595320	-6.07093209954395	0.23359064993604
H	16.29350456359936	-3.66539899486988	1.94161419281099
H	11.79522479823764	-2.51885182642542	0.34315899701690
H	15.86761514169812	-0.25263157468888	1.85451026824696
H	16.61324587612103	-1.53735885536108	2.82317296506183
H	13.58790377354894	2.19637877640003	5.09540233547615
H	14.56320922558508	-2.56195668453705	4.42539899748968
H	12.47583579439424	0.12959220459859	7.79378343341498
H	12.44377850043177	1.75812603334202	7.07054699507890
H	8.40616364453339	1.84947296618033	4.55691375933831
H	10.68289533346824	-1.33531816906448	7.51379622088289
H	11.11169209559351	4.19628977314866	4.18709020345191
H	9.47750465310071	3.87743160667662	4.81406013926383
H	10.07895511584219	2.98467346537307	3.38654447576344
H	7.92532317939336	-3.63521678405608	7.94549422897367
H	8.98621518373596	-2.41928378556377	8.69832814478228
H	9.57094222094110	-3.37842249233983	7.30796076778342
H	12.92575229217647	-3.76198883640198	5.48541115244660

H	12.74865964663150	-3.84478421624209	7.25819986390538
H	14.35179111611292	-3.55087104650733	6.54129262246120
H	15.22842118343485	3.44166825657622	4.03427579808452
H	13.82500935573031	3.13989983685413	2.96794552374441
H	15.42997402802389	3.46853486774558	2.26475934572064
H	17.00593605999156	-5.52142244171502	0.74225379334019
H	16.28364130720020	-7.13669101744467	0.94455168670654
H	16.25853536084064	-5.98196640076643	2.30078958976268
H	11.72653196509528	0.87821877526490	1.59208927854007
H	11.14308621534921	-0.78848849971580	1.82963413505409
H	11.59088353672329	-0.21685773673054	0.19190875832762
H	11.67247729213448	-6.19874545303535	4.19259132581928
H	10.03606909749502	-6.60386642321619	3.61976066829450
H	10.41820045600874	-4.93137902262899	4.12226033290487
H	7.45747933425316	-2.97388078242928	-3.38802123929756
H	8.71977086573060	-4.23187375991290	-3.37685420160874
H	9.08226159556412	-2.58590598802548	-2.77613041282905
H	-13.18592932048972	5.59407291523606	-3.51225388293080
H	-8.37852364833484	6.32997698132750	-4.15853036245449
H	-13.55340183062334	6.53993587073024	-5.74366466511928
H	-12.18813946485344	6.61151430382503	-6.88456661865852
H	-15.36020881579993	2.25612990677826	-6.41328887212313
H	-11.16407037945812	4.48735565465745	-7.61827327042463
H	-14.94689181578543	0.62070198044776	-8.18352686937785
H	-13.29886977874935	0.45766209855941	-8.84225883202868
H	-14.84464726738598	-1.76950657616453	-4.09762408937021
H	-11.52414010337334	-0.40895650583466	-7.44312296192687
H	-12.03432553692122	-3.63474200454312	-3.72425541613128
H	-13.25254027966708	-2.75324493013352	-2.78177394219133
H	-10.38272084316209	1.17726367614610	-1.67700557659409
H	-10.07468349947466	-3.64076864931642	-2.53545975516146
H	-13.57220844003137	1.74927877613611	-3.21934369409260
H	-11.81371958275745	1.93928214061168	-3.38167060579863
H	-12.55471195182894	1.86868910632557	-1.75689411819202
H	-8.52568956310850	-4.49365228863468	-0.99605119786957
H	-6.78031798325507	-4.16565957524339	-1.13597475998284
H	-7.80255616299710	-4.15616198111809	-2.59595386549302
H	-8.64761174740324	-1.98649584296101	-5.91493259781809
H	-9.48518271881449	-0.45781262524236	-6.28300659883867
H	-9.72480720633162	-1.88577414932010	-7.33170920109015
H	-17.71364837702509	-0.16382057147466	-5.57363357273402
H	-16.58825030843009	-0.27017300403180	-4.19536577247413
H	-16.91392134115268	-1.71116958512560	-5.20167474020159
H	-16.13533284490430	3.22201856825470	-4.46683243102741
H	-16.86028178997682	3.98818670259715	-5.90857811914280
H	-16.74788166357553	4.89324817991004	-4.37769766614381
H	-9.76957596213269	1.84741633477479	-9.64295452012581
H	-9.64449113591135	2.83154408443215	-8.16175567847040
H	-10.45717744825408	3.49078439198906	-9.60992052459846
H	-7.87465060031076	5.88588013225506	-6.35716134783070
H	-8.21079662461604	7.62188988786776	-6.10635401383175
H	-8.44216851344102	6.89193849987496	-7.71434028990452
H	-13.50008155485325	4.50568055800997	-1.52306023852859

H	-13.57446855924469	6.27069912896218	-1.25941579375470
H	-13.12684178864505	5.18416900163231	0.08143766366180
H	-3.95541363494332	-5.68426384829078	5.79732560268587
H	-2.49752910612913	-5.79350866449649	6.81095511160182
H	4.14552319532629	4.61712397870487	-2.54429114829170
H	2.71960861811413	5.63434004074305	-2.36232924752525
H	-9.41537687052450	6.28366297993540	-0.70231783268608
H	-8.05222396593897	6.44790475559496	-1.83195323542569
H	-7.09377231222626	-0.11656075533829	-1.41281562121171
H	-7.86183294165027	-0.17119203837385	0.16411561943216
H	1.25476841920816	2.76616685948443	-8.41881769364964
H	0.24752268960140	4.21632990711579	-8.25386890902952
H	-4.70068272911169	0.42607617712903	8.72852517259876
H	-4.28207327851333	-0.45308460712832	10.20452539318874
H	5.78398141284291	-4.39418265773396	3.49031190185738
H	6.29335719245237	-5.06995623859666	1.33741457807473
H	6.24903995511040	-3.83551756509205	0.06669667489682
H	7.49361016238182	0.12070229370728	2.67202180777903
H	6.34797633683148	0.76496631838676	4.91581031081645
H	6.23885012404797	-0.54431120806862	6.11564850882747

Table S 9: XYZ coordinates of the optimized geometry (GFN2-xTB) of **all-p*R*-[15]CPP-PA$_5$**.

C	-3.75828360377590	0.65307626408458	-3.83699639612614
C	-5.10871951675810	0.69183620816775	-4.19476585419944
C	-6.02118467250715	-0.06223746981081	-3.46997375206138
C	-5.63318631113720	-0.83400190708459	-2.38649646154285
C	-4.28703112462229	-0.84907947831541	-2.00907177134621
C	-3.37050916929018	-0.11231846280285	-2.74803909959200
C	-6.67293293865424	-1.62318785273892	-1.63020044353188
C	-7.47278620163889	-0.70907279256256	-0.73703628139883
C	-8.67048998470731	-0.13697707317284	-1.17492629695585
C	-9.35730250473924	0.72844404661507	-0.33326578569278
C	-8.88661948715819	1.04203157096898	0.93087410188390
C	-7.69649028956334	0.45560582295968	1.37611153575908
C	-7.00765354878680	-0.40604777192845	0.53294244729823
C	-9.62876330099502	2.02447144845546	1.80136758362264
C	-8.87316881932820	3.32724137506536	1.86026371109117
C	-8.82276979519036	4.16637536557307	0.74105617045854
C	-8.05050942496723	5.31849303081808	0.79283595565395
C	-7.33999626149263	5.66876512763296	1.93143477201171
C	-7.42232477455501	4.85045295171298	3.06189698043547
C	-8.18627403742138	3.69081473099989	3.00596641293259
C	-6.45421939470624	6.88249131032108	1.91151865186675
C	-5.16322955424247	6.62813685100756	1.17090697356475
C	-4.49496295188305	7.68401509299243	0.54335889739867
C	-3.31381742905574	7.43080237161202	-0.14232590569159
C	-2.78448150929388	6.15347475708148	-0.22879675916379
C	-3.44192866090164	5.10193374204658	0.41793999883073
C	-4.61836685614122	5.35606594235785	1.10740794177765
O	-5.07811023325976	8.92305946558535	0.64694002245703

O	-2.86046415613914	3.86291740648065	0.31970556128197
C	-4.49128355268969	10.00001307392167	-0.04365282873567
C	-3.56707946025625	2.75043762337342	0.80675073347985
O	-9.56846984036196	3.78401500474406	-0.34615648966206
O	-6.71486758063088	5.25316328691915	4.16853491759202
C	-6.78623529168765	4.46631297919542	5.33309887620149
C	-9.54449672759677	4.59493126969408	-1.49510461881166
O	-9.09927858758722	-0.48169943653954	-2.43566064785830
C	-10.33748519057379	0.01560805470582	-2.88408462848159
O	-7.29321914304481	0.76849365564545	2.65039878957678
C	-6.16067715890676	0.11491539747268	3.17163592192170
O	-3.95492931621983	-1.61714207754533	-0.91788453719941
C	-2.59264508643513	-1.78419470606735	-0.60451371459013
O	-5.44599194057334	1.48041360876927	-5.26882095089930
C	-6.79665094966980	1.53328278613868	-5.66127684407217
C	-3.26673445468639	7.09169037254508	-3.49701328302016
C	-3.84984177535200	5.95678493644188	-2.93544576846408
C	-5.22118186720166	5.96373178392336	-2.70839095840514
C	-5.99068514617347	7.05938900502702	-3.06036547502409
C	-5.41480039982983	8.17480081045562	-3.66409896050733
C	-4.03195871326855	8.17867132478968	-3.85374153652097
C	-6.18729093094562	9.28612313477655	-4.23771347433170
C	-7.35621245640952	9.05107552408536	-4.95846445250373
C	-7.91485710800724	10.03824247685944	-5.75021548093495
C	-7.32191231801832	11.29384839997534	-5.85122190679572
C	-6.24027568473152	11.56979225749985	-5.01357399936177
C	-5.68279414056623	10.58708751865083	-4.22416651092738
C	-7.64193011028368	12.30994195583790	-6.87465842376815
C	-7.49514703345280	12.03244468092088	-8.23792563215247
C	-7.43742298779805	13.09808847800129	-9.12348227247793
C	-7.54828334599554	14.42035412836975	-8.71571047688790
C	-7.87439523862804	14.68463683424875	-7.38124268815326
C	-7.88767969993081	13.62756417213160	-6.48700194196774
C	-7.12984757449792	15.44460587025333	-9.69048224136623
C	-7.65492756573533	15.51503386201484	-10.97744215453906
C	-7.02925898113370	16.26681474204937	-11.95381824573895
C	-5.85529976353136	16.96489841383558	-11.67836382596943
C	-5.38755544907936	16.97078688399368	-10.36456161556156
C	-6.01125117540178	16.21978295580433	-9.39076198193960
C	-4.99681722840734	17.51400089465762	-12.73813893256799
C	-4.74711083463716	16.73431113085379	-13.86724215625501
C	-3.73270603331219	17.05952472737080	-14.74319857053280
C	-2.93630452487851	18.18526041484429	-14.53194593188624
C	-3.25710471995573	19.02088940873901	-13.46418856660197
C	-4.26822893518125	18.69181066012323	-12.58210447599472
C	-1.67439953944865	18.31537450454147	-15.27694730725343
C	-1.54270514926044	18.10097608454314	-16.65292026624831
C	-0.27868685761332	17.84191424743891	-17.16030402095155
C	0.86883951020423	17.85955673046957	-16.37032468511525
C	0.75449812170062	18.29304864716100	-15.04131078073583
C	-0.51508129284788	18.48170838765116	-14.52291848531312
C	2.10745072607260	17.22881331014089	-16.86465982614328
C	1.99183071209631	15.90934468002376	-17.30649103295109

C	3.10899045629607	15.13636675812438	-17.53323763794380
C	4.38892674177499	15.65597873522267	-17.33794407124637
C	4.50487720071730	17.00815364452569	-17.02688332084202
C	3.38251547653639	17.78370734408688	-16.79007963053095
C	5.54600781168439	14.74817362598611	-17.32013668002172
C	5.40500246499161	13.54025949428510	-16.63530570710937
C	6.46424932087589	12.67164029432345	-16.51015707349941
C	7.70506262295612	12.96991091053891	-17.07292250641602
C	7.84995062204792	14.16984517908674	-17.75516409386851
C	6.78486168294230	15.04837313706043	-17.87733606545516
C	8.79439617023948	12.01217481150319	-16.79836333821664
C	9.85565940655500	12.38835955538499	-15.98531398382885
C	10.70119044555990	11.45763970949237	-15.39530510614210
C	10.45362781308636	10.09992210620229	-15.61379303254071
C	9.50553253955483	9.74141100441123	-16.57314978918454
C	8.67925487617982	10.66930603881775	-17.17924336489881
C	10.95631303726274	9.02386685677680	-14.74376057355132
C	11.38883789792421	7.79149596009314	-15.22694740940858
C	11.54346643786202	6.70942079776067	-14.37936234077244
C	11.27146974221530	6.82485395283129	-13.01793475116175
C	10.93691460670473	8.08546347715898	-12.52150243162520
C	10.77719404183740	9.16127149907086	-13.36688323720114
C	2.55025790475623	1.14846583216923	-4.79244495815041
C	1.55049755831339	1.67427311393353	-5.61388179420313
C	0.35874829085959	2.11667397187881	-5.08751778350072
C	0.11388201965743	2.05426237100097	-3.71486702765657
C	1.07636635238198	1.46273043874700	-2.90268194749546
C	2.27367726688493	1.01294816755767	-3.43353015455238
C	-1.05947410545595	2.79354956442560	-3.21668699260560
C	-2.32896255691621	2.65370788106865	-3.78387632156991
C	-3.26383469529644	3.65525041966070	-3.57130161752457
C	-2.98293655596767	4.77188797705561	-2.79431041718730
C	-1.79188680589022	4.80110292433891	-2.05960872096570
C	-0.84589423527749	3.82028936212719	-2.29829930921347
C	2.38440574134481	20.01008200091061	-14.28325458521768
C	3.12975717068748	20.60885014583084	-13.26203361684786
C	3.52877334706858	21.93145062966960	-13.39962363901535
C	3.22356584446213	22.68707744191260	-14.52524024752071
C	2.49095544896481	22.08198865727255	-15.55016368769782
C	2.07849512825046	20.76290999236707	-15.40258207926277
C	3.72379210070356	24.11252251284870	-14.59695538372572
C	2.84927874489986	25.02509018696701	-15.40875045387207
C	1.51154643186409	25.22057944291235	-15.04752664066197
C	0.69124634475493	25.97715683574570	-15.87166968720124
C	1.17143198489835	26.56527830536409	-17.03295057886032
C	2.52385277992946	26.42759089917633	-17.35418569199885
C	3.34205103390296	25.65960617687154	-16.53558800408080
C	0.21576284723882	27.25651965570279	-17.97275803420963
C	-0.49880970690922	26.20619413003583	-18.78890399495853
C	-1.88020687262112	26.01725968997093	-18.69885108180781
C	-2.47054284626858	24.98883392003288	-19.42533149006359
C	-1.72336969404149	24.12273575256632	-20.20987608888407
C	-0.34310477076162	24.32684709183786	-20.31524375288353

C	0.24134584407824	25.37266605215574	-19.61395397010109
C	-2.37377575680150	22.96167947381085	-20.90861250026655
C	-2.41273664502986	21.71746186243458	-20.04886310681831
C	-2.18134885230462	20.45753459838019	-20.61005976720777
C	-2.26831883988166	19.32711170530578	-19.80378088585782
C	-2.59694612964170	19.41109383148404	-18.46038186346014
C	-2.82805359116431	20.67198057998539	-17.90076025264846
C	-2.72594383308086	21.80040954006204	-18.70166037727055
O	-1.89777659029363	20.42304285253525	-21.95194571273979
O	-3.16802123510699	20.70077358729519	-16.57281410499274
C	-1.60121543670169	19.18316179091368	-22.54825623089447
C	-3.51392183880438	21.93614575586691	-15.99854391552966
O	-2.57401123390218	26.87493134890887	-17.87752322018694
O	0.34857435307125	23.45870622746059	-21.11969345535432
C	1.72519369054262	23.67898720933242	-21.31702884180538
C	-3.97375101343450	26.74858843661690	-17.79877129085209
O	1.10455885399024	24.61421430029338	-13.88591518406558
C	-0.24624767228021	24.72118902619507	-13.51085239248348
O	2.95676956292047	27.05685742088244	-18.49964418985779
C	4.32329992769895	26.98974757764661	-18.82930576024035
O	2.21131905560861	22.81226335464163	-16.67867762372711
C	1.18263500200825	22.36029514181893	-17.52591713353727
O	3.41104855624297	19.82122350472702	-12.17264061533287
C	4.11896847328381	20.38833648648309	-11.09576606573428
C	8.13604823006121	10.80780722961538	-19.58094992621131
C	7.27146166383260	11.57700921606499	-20.36322951956720
C	7.72735431890486	12.08063936790212	-21.57723096577069
C	9.01051940882492	11.83065738263400	-22.03592461186046
C	9.87362230489902	11.05634659570506	-21.25112194455292
C	9.42304154763237	10.56146372887319	-20.03628811012097
C	9.48872814880006	12.37906427849910	-23.35013919072002
C	10.55690253865164	13.43286641737163	-23.18848008140659
C	11.54753054291155	13.58351555573744	-24.16326485423813
C	12.53897465319606	14.54208281694914	-23.98537284455674
C	12.55935294281523	15.36570659870984	-22.87183493128022
C	11.54763506738074	15.23560096228482	-21.91376541714271
C	10.56538433370150	14.27039199494089	-22.08438410682533
C	13.67634611462185	16.34794516514107	-22.63477196282709
C	14.37689850961946	15.99477495532728	-21.34592092675480
C	14.49135475126151	16.91313948477912	-20.29957014076661
C	15.01997487845061	16.48964032232118	-19.08621122650369
C	15.45447397865316	15.18863525514557	-18.89294376348887
C	15.39466123905246	14.28721769005143	-19.96041869855623
C	14.85047121023377	14.70417877512243	-21.16656972240366
C	15.95328384933617	14.74584665125413	-17.54091449088878
C	14.86010622136619	14.03497964709607	-16.78214935309508
C	13.91396938359214	14.75378053257310	-16.04438214823245
C	12.94048233451217	14.06320568381263	-15.33194621104499
C	12.87076731163333	12.67896985617198	-15.34673177144554
C	13.79516319298302	11.96282033240788	-16.11447013619565
C	14.77933737952095	12.65206243884924	-16.81045836017458
O	14.03573570609806	16.12181037238321	-16.05273133252308
O	13.67399201307251	10.59717098664628	-16.09842168106458

C	13.10902267343617	16.88506687079563	-15.31909234762896
C	14.60785494341478	9.83392239726019	-16.82290224243778
O	14.06430508978778	18.19329084083564	-20.54610581903859
O	15.88744915074064	13.02349300498063	-19.73576685888932
C	15.89074501231500	12.10950318991555	-20.80585364199775
C	14.06050912128780	19.11355042904223	-19.48166590054014
O	11.46840561960433	12.74776222412519	-25.25046703765788
C	12.46635335991111	12.83285094030824	-26.23964439801781
O	11.60544320340647	16.10001112195965	-20.85209522420146
C	10.75347182174494	15.87835548328637	-19.75912818045424
O	11.12648932567913	10.83273421801281	-21.76079590300915
C	12.01969797103130	10.04550467076147	-21.01149329920405
O	6.00425324464500	11.77543509057523	-19.87436619402436
C	5.11851848217979	12.58603992334405	-20.60667752145836
C	-9.06114583250515	16.77834051359921	-7.96770570852110
C	-10.27198950850642	16.20466678589803	-8.36943900014465
C	-11.00646768817672	16.80385585175335	-9.38166945721555
C	-10.57276442363860	17.96192849591123	-10.00664317282706
C	-9.37606911119495	18.55100795107747	-9.58764433007504
C	-8.64220042562820	17.95247495077144	-8.57105948494861
C	-11.38558344869731	18.58800904797756	-11.11421388038596
C	-12.10248178145802	17.55825399843820	-11.94288206451074
C	-13.46008302317363	17.28820893426908	-11.74649221414565
C	-14.06785932385278	16.28391900086499	-12.49042622252967
C	-13.36457742465070	15.54734676608000	-13.42818632563422
C	-12.01417611017478	15.84091053175998	-13.65047823359426
C	-11.40313370827790	16.83348294358692	-12.89656708353638
C	-14.03000539198434	14.41738202621515	-14.17160276130521
C	-13.54890403451273	13.09458083112494	-13.62994247497172
C	-13.98814993988301	12.63661054452316	-12.38289556981760
C	-13.49754051828959	11.43523217171926	-11.88980227120392
C	-12.58167139603395	10.67599336565435	-12.60048111821547
C	-12.14792451992136	11.12910196238822	-13.84937022908227
C	-12.64317584231135	12.32850566618128	-14.34526180533754
C	-12.04381096419186	9.39763828116081	-12.00818070592249
C	-10.82853171903754	9.69736985511049	-11.16795876228750
C	-10.94990593935091	9.99224625483398	-9.80664644399329
C	-9.80953112967409	10.29690031121208	-9.07716437970907
C	-8.54868353744824	10.30566493130126	-9.65451342408593
C	-8.42949944287045	10.01702758278577	-11.01746312055932
C	-9.57178774743373	9.71916779569452	-11.75010450879009
O	-12.21555856720015	9.95142465470463	-9.27056238710943
O	-7.16373255915011	10.04808765784454	-11.55212708360018
C	-12.35360077590633	10.03918198149337	-7.87249312841200
C	-7.00931950367245	9.77412621644679	-12.92431092066408
O	-14.90718354068713	13.41710111349110	-11.72594623137481
O	-11.24872820238269	10.33265065048577	-14.51841166318540
C	-10.82459105389319	10.72970920649848	-15.80042137741445
C	-15.40378380740361	12.96477698971704	-10.48885483571438
O	-14.11036171863225	18.04603923327507	-10.80204335927324
C	-15.48474838713164	17.82552237931826	-10.59484912643294
O	-11.38589867745713	15.11794505089635	-14.63366358481908
C	-10.02685363674141	15.37197158579591	-14.89062495495016

O	-8.99770423005245	19.70158173859935	-10.23571598390425
C	-7.74724634014597	20.26681133401029	-9.92204022525460
O	-10.65246584617173	15.06157811958737	-7.71293407915684
C	-11.80248396689507	14.38629105915919	-8.15511530537255
C	-2.72205624455227	1.44457719659309	-4.59537544365215
C	7.70305706693629	10.24966997102142	-18.24638892929453
C	1.93708225685135	18.57051185095338	-14.15909158892636
C	-8.22086080226395	16.08336001742702	-6.93012497100087
C	-7.34071567208877	10.64948471515026	-8.81603150249341
C	11.84139496377151	11.93917980817122	-14.53070428149547
C	-2.71653408462615	18.17919411720472	-17.59855717445551
C	-1.54014933775438	5.87350643483485	-1.02787364841873
H	-7.06593330399810	-0.07130520537504	-3.73245935444479
H	-2.32296949632261	-0.11741090953445	-2.48938260542093
H	-6.17528611523190	-2.38278666905454	-1.02774036985239
H	-7.34710815295788	-2.10465012113679	-2.34001768400715
H	-10.27755372011342	1.18948298899974	-0.65257651726005
H	-6.08219561253266	-0.86285684217095	0.84498166692237
H	-9.72443891462307	1.61730805437688	2.80914630315066
H	-10.62188055489894	2.19750999272372	1.38381375758300
H	-7.96867011744404	5.96968040004411	-0.06325543146331
H	-8.24518023728653	3.03093303722586	3.85589523227681
H	-6.96509304713644	7.71874591709839	1.42994771001174
H	-6.22155765449471	7.17747034227825	2.93743183622520
H	-2.79182602157069	8.23152459758775	-0.64236745434397
H	-5.14464281127779	4.55580101755422	1.60167871134305
H	-4.43432941142701	9.80100929145348	-1.11791235503001
H	-3.49160847832014	10.22806338615289	0.34105452824149
H	-5.14706733337721	10.85198646518328	0.13252254555267
H	-2.96188090910224	1.88422698021294	0.54226514995896
H	-4.55234080792993	2.65540611288049	0.33665990553447
H	-3.68623780698938	2.79332860061337	1.89517554552206
H	-6.39176175536407	3.45930354055895	5.16272937082016
H	-7.81166378540027	4.39735118536229	5.71074754703420
H	-6.16486324075498	4.97711819771051	6.06799381718861
H	-8.54412817309716	4.63348596798377	-1.93834638299634
H	-9.88930553579015	5.61196423612749	-1.28103476298845
H	-10.23142369739561	4.12743596118486	-2.20019918692148
H	-10.33013891157018	1.10806603127699	-2.95596863600843
H	-11.15849918588846	-0.30103829469453	-2.23309799006840
H	-10.48089452499488	-0.41004579254597	-3.87711569275467
H	-6.05819860925877	0.47523719410522	4.19489384743098
H	-5.25508975075638	0.36202265984435	2.60827047278709
H	-6.29503725858484	-0.97172846983947	3.18459428464479
H	-2.12535322879306	-0.83933145172913	-0.30826262709814
H	-2.03708709283738	-2.21603962465212	-1.44316684365667
H	-2.56953429455340	-2.47546483238030	0.23754599794502
H	-6.82804889126504	2.22034762457983	-6.50651489147461
H	-7.43355231294710	1.91740360301082	-4.85755009680140
H	-7.16267883338216	0.55128132009373	-5.97787209835714
H	-2.20542953142402	7.09452513722757	-3.70177504617500
H	-5.68488778480975	5.09841726804607	-2.25465060883627
H	-7.05616959452851	7.04832081139986	-2.87661983949390

H	-3.56295292660162	9.02336826383969	-4.33810391558949
H	-7.79586027096879	8.06361532193934	-4.95388344310576
H	-8.80559907790879	9.81204202709555	-6.31713215927388
H	-5.79724070301117	12.55560109972637	-5.03509273723081
H	-4.82239579095801	10.82377600878777	-3.61454370553108
H	-7.23509069281222	12.89091577136286	-10.16552965658597
H	-8.07892833488745	13.82815012914060	-5.44093698684344
H	-8.54754295750698	14.95167934211192	-11.21422756179564
H	-7.43203829337098	16.29189413462456	-12.95689513920578
H	-4.48238540832243	17.51150893516723	-10.12517076309816
H	-5.58757490119801	16.17724431728103	-8.39744055159468
H	-5.30520405268556	15.82054073102132	-14.01477456197002
H	-3.50392189671749	16.39008660489946	-15.56003271088687
H	-2.68149646483262	19.92192323792231	-13.30573257313867
H	-4.47592576978237	19.34251564671203	-11.74375301462024
H	-0.17702521650533	17.60090702148741	-18.21000898762220
H	-0.61118905464168	18.71092751991482	-13.47065603495713
H	1.00808008686131	15.47278773991522	-17.40859378432122
H	2.99237528979210	14.10551462989533	-17.83558870305643
H	5.48650767742126	17.44490384827602	-16.90593140488084
H	3.50445317994555	18.82302564479026	-16.52192171060922
H	4.46119488989169	13.30249545990704	-16.16560391081088
H	6.34623793405821	11.75771755044861	-15.94428473267783
H	8.80513107141452	14.41630590184623	-18.19593564718966
H	6.91551312108243	15.97500475725007	-18.41984445213196
H	11.57472625118672	7.67196214924744	-16.28526433541174
H	11.85209679361248	5.75473385630640	-14.78280048433384
H	10.72415740957989	8.20241121072422	-11.46847043882783
H	10.43313169826300	10.10418758084361	-12.96692863259806
H	1.73972921444808	1.80165038502469	-6.66953828004533
H	-0.37338368436717	2.57915290933183	-5.73397551430338
H	0.89482751579193	1.37450125789764	-1.84013148103885
H	3.01124311434358	0.56921926675024	-2.77919120382859
H	-4.21558509292540	3.59547337538356	-4.08021991404821
H	0.11472685734509	3.88210887686286	-1.80454469731279
H	4.09320731362980	22.40925147265717	-12.61385148818282
H	1.50031658549336	20.30031482231529	-16.18547678926169
H	3.79355200278420	24.49719779924920	-13.57557919305066
H	4.73089976020342	24.10867863226213	-15.02542033335557
H	-0.35420331624766	26.10978106464554	-15.64027823193422
H	4.37989844331877	25.50914094337929	-16.78855903959824
H	-0.51676772571338	27.83806534456263	-17.41423123705345
H	0.77571182522243	27.92002895293775	-18.63312199607298
H	-3.53892468355070	24.83942782950414	-19.38750675840355
H	1.30048321656421	25.55741656122174	-19.68108292709712
H	-1.82333751027455	22.72963136245669	-21.81943796137496
H	-3.40041895935118	23.22450333298350	-21.18191750529084
H	-2.09922833356305	18.34769957261767	-20.22408837903807
H	-2.88832829026049	22.77959856428311	-18.28307453946187
H	-2.45565631966892	18.49938718106583	-22.50320708660209
H	-0.72852221947782	18.71106883485420	-22.08518499673642
H	-1.37753234585320	19.40786449046749	-23.59053030218914
H	-3.79688895225164	21.71301714837399	-14.97090496805048

H	-4.36439914898902	22.39490815035190	-16.51441272180403
H	-2.66922004089983	22.63372511069276	-16.00083358479577
H	2.04723451228203	22.90053476379057	-22.00780999620000
H	2.28802716540001	23.58822954178462	-20.38225846634370
H	1.91495140699645	24.66108120114752	-21.76266215258011
H	-4.26980397462401	25.77657750513618	-17.38946300994521
H	-4.44746590520679	26.89424233191326	-18.77489280934637
H	-4.29939564780740	27.53667551705792	-17.12063909103145
H	-0.33919799141073	24.14975252482164	-12.58788246869096
H	-0.90985831419745	24.29245045220687	-14.26893898100482
H	-0.53302995905176	25.76175640152398	-13.32417665289502
H	4.43330307764211	27.58338195831082	-19.73648352559268
H	4.64020171569171	25.96073320478889	-19.03066801080908
H	4.94972815068291	27.41681994846656	-18.03958092812414
H	0.93357013595124	23.20504084393274	-18.16701837109465
H	0.29040260203340	22.07017535855136	-16.96294505348018
H	1.50255834114177	21.51508564402678	-18.14579858718428
H	5.12183770251052	20.70830235896813	-11.39684226625146
H	4.20301010750244	19.59550367432090	-10.35322345855523
H	3.57980723917717	21.23642520066819	-10.66115829577437
H	7.07840267395889	12.68182991741627	-22.19518133260284
H	10.07076712304628	9.97138734019010	-19.40731624810941
H	8.64686246910598	12.80486050258463	-23.90373344157317
H	9.90887011979024	11.56945125339917	-23.95060041270237
H	13.32470846652913	14.65772055331006	-24.71621140021093
H	9.78826434519930	14.14092982112601	-21.34741499238484
H	13.27353081016947	17.35711659237189	-22.54313795684668
H	14.37887976478068	16.32365731372137	-23.47096221815567
H	15.08702331532140	17.16525350213112	-18.24915627916326
H	14.75429323975972	14.01834700079396	-21.99372327136251
H	16.79640048600647	14.06653002109339	-17.67454208728997
H	16.27411077511863	15.61811128283530	-16.97017784291199
H	12.22248373598152	14.59999270669412	-14.73114106410462
H	15.51409792850748	12.12345971860633	-17.39469200208072
H	13.39481816867492	17.92488831061926	-15.47656499632712
H	13.15591611693267	16.65936707314781	-14.24843574376158
H	12.08586667294951	16.73296478562856	-15.67949492331514
H	14.32654436576335	8.79468890927961	-16.65807731296625
H	15.62809780604760	9.99291290259182	-16.45801839646719
H	14.56667193920018	10.05777345756985	-17.89412362219433
H	14.87590036209571	11.89971509296457	-21.15889405376906
H	16.33358395160922	11.19535299585065	-20.41059557491227
H	16.49833779812482	12.47191060625733	-21.64179871291963
H	13.44229746927694	18.76287266934589	-18.64910697704163
H	13.63293577207467	20.02938534944232	-19.88892459677726
H	15.07471666671020	19.31773417261186	-19.12165424167047
H	12.48089328316956	13.82000467562659	-26.71339792348600
H	12.20340390432387	12.08170852419785	-26.98365905318488
H	13.45793149638403	12.60720738688411	-25.83354688195385
H	11.04172793334650	16.61387048430273	-19.00838335209375
H	10.88878536021125	14.87136300238681	-19.34796568811888
H	9.70188040254549	16.03006524807426	-20.02746703493532
H	12.92364246342673	9.97456946915854	-21.61556676054608

H	11.62412028221244	9.03881411817712	-20.83964028808088
H	12.25729388458423	10.51228036161846	-20.04904151210809
H	4.20001516124021	12.61370834012676	-20.02254013798698
H	4.90531805142320	12.16154609923980	-21.59353480955146
H	5.50265668811473	13.60485569982675	-20.71997322833970
H	-11.93280245583823	16.36769896768631	-9.71779301234916
H	-7.71073135195391	18.38719553586500	-8.24418712893691
H	-12.11939343830385	19.26668213551255	-10.67268049726978
H	-10.71392048825033	19.17689153784693	-11.74129391610409
H	-15.10721156761118	16.04178433291306	-12.34092391837038
H	-10.35747489101564	17.06385604767023	-13.02708342584294
H	-15.11129380341999	14.48910666699437	-14.04481309398164
H	-13.78272427391780	14.48457336723763	-15.23214595400828
H	-13.81068456818234	11.06178047455147	-10.92873753059029
H	-12.32028530975149	12.70174627365870	-15.30325099566621
H	-11.77136441655422	8.71618206089546	-12.81448774071256
H	-12.80785489886207	8.93753133608324	-11.38083972485153
H	-9.88435192736037	10.55606572074374	-8.03362216459265
H	-9.51066860650902	9.49718666964958	-12.80286838419383
H	-13.41632775226604	9.90317180809786	-7.67327464754450
H	-12.03865036189814	11.01671202954881	-7.49327163246401
H	-11.78690224861552	9.25249673822078	-7.36384499526025
H	-7.56297665554412	10.48880960755984	-13.54195488406305
H	-7.32863367054991	8.75595050346771	-13.16923713335254
H	-5.94274045475500	9.87507489043656	-13.12296127942788
H	-10.14319864883918	9.94895280640672	-16.13821756362747
H	-10.29301015327660	11.68626632310183	-15.76910946683425
H	-11.66469188411872	10.80233887955062	-16.49859956122652
H	-16.12199025221102	13.71838737615874	-10.16698292894672
H	-14.60831509741318	12.87921167672610	-9.74127201544035
H	-15.91470243761501	12.00142394311678	-10.58854708216789
H	-15.68014127465491	16.80773694159102	-10.24181426114302
H	-16.06481598743870	18.01353797265853	-11.50424044121591
H	-15.78080338651367	18.53709872157309	-9.82467983092953
H	-9.76119450801785	14.73292424611621	-15.73254820326933
H	-9.40295684282773	15.11201484109292	-14.02942596899113
H	-9.85376900184996	16.41719234104216	-15.16738871905115
H	-6.93419339758009	19.55287535618588	-10.08577810544294
H	-7.71646231811356	20.62718700650073	-8.88830246707300
H	-7.63105042801055	21.11170448855410	-10.60009883806504
H	-12.70030985703080	15.00674111265213	-8.06004589836351
H	-11.89624408487888	13.52000940500834	-7.50139844081651
H	-11.69976291888879	14.05022517335924	-9.19338794831290
H	-3.14546913855504	1.75531703311583	-5.55154704387777
H	-1.85147336268949	0.81306359017689	-4.77789919802977
H	2.77635943810338	17.91494758219819	-14.40841616476798
H	1.68008517839021	18.38502397919924	-13.11383519958771
H	-8.77882259846657	16.01627715157271	-5.99235337792123
H	-7.31476242444617	16.66461555810394	-6.74248893480797
H	-6.45680375869467	10.61979052601975	-9.45490819997202
H	-7.21400908426493	9.90846862786009	-8.02562924055058
H	-2.75003358909406	17.29137672518672	-18.23412959216580
H	-3.64282123537938	18.24954788088492	-17.02791723825617

H	-1.19709586670262	6.79539400135742	-1.50356164697657
H	-0.74833772503306	5.52725075172355	-0.35780326482999
H	9.36243451270080	8.69097008267198	-16.78953292118797
H	7.67025435297745	9.15817668704899	-18.30291376144136
H	6.69992158953325	10.61403990381074	-18.03144204304184
H	9.98378001849387	13.43669867839060	-15.75244632797400
H	11.45717179653008	12.60160052795531	-13.75074134445527
H	12.33020025846977	11.08326113489190	-14.06484957286506
H	2.88606776466624	4.55857196611276	-4.20256463049602
H	6.01155939271764	5.11430075206796	-2.48262395387153
H	8.58897831560691	6.71203210277925	-1.62084873257335
O	4.77999680881067	4.58588849135748	-4.84015425644154
H	7.84888767210081	5.20420396987409	-1.05820205848783
C	3.87205448727247	4.74375995998693	-3.77739828558517
C	6.64932850222946	5.10011327302244	-3.35286330232099
C	8.54132263705705	5.63208278742256	-1.78817036495829
H	4.05385138933043	4.02253661867399	-2.97459635746021
H	9.89870608622207	1.45613298477417	-3.34135014534895
C	6.11489963284190	4.80809963332682	-4.60284848076320
H	10.77846133352699	6.88916358585912	-1.01136535921417
C	7.99847139463986	5.36509674507286	-3.17197077999842
H	3.90744701752937	5.75924715613201	-3.36841300377957
C	9.91320020911202	5.04528096141407	-1.58607086536789
C	10.97619376449308	5.84616039558855	-1.20299928054533
C	9.24646415170643	1.56975806672357	-2.46832062010925
H	9.66878111437323	1.01196438047147	-1.62524520916672
C	6.97379241348620	4.71818898350040	-5.70251236930755
O	13.35699849149274	6.09984105635935	-0.72927198531186
H	8.25841967667369	1.17315951765440	-2.70026596593296
C	10.14364981714918	3.68669660285819	-1.82785823857783
C	12.27017163099035	5.34881510979415	-1.10587854609192
C	8.83288569940383	5.38428565619102	-4.29471544235650
O	9.04591951571465	2.92367784738610	-2.14647611121873
H	14.17470228190303	7.87058322450758	-0.30146985063283
H	6.33556875694321	5.20544927578907	-7.67672368924439
C	13.18291193229578	7.48235508197947	-0.53166570663781
H	12.80110390503112	7.97274940103696	-1.43299016983483
C	6.46273953131802	4.30696996567994	-7.06414626583021
C	8.31276566159316	5.02753110007454	-5.53050099527078
H	5.48410286300248	3.84298129617291	-6.94086566620191
C	11.43801487745471	3.19338966170583	-1.74822943267733
O	10.13397569423512	5.76838743632030	-4.10455615556808
C	12.50925645599408	4.00721711196599	-1.41262661226787
H	5.27033606669514	0.56053864395684	-8.45376903804778
H	11.64657350490141	2.16001381022619	-1.97618943965303
H	12.51180887412082	7.68853199987543	0.30908770318166
H	8.38198817221553	4.94961741423756	-8.81737908346732
H	8.95721882551105	4.97992852999903	-6.39311614266726
C	13.91176662464305	3.44651519598415	-1.38877843245112
C	5.19923086545022	0.79909833693605	-7.40137366836835
C	7.41652835490051	3.37107885764939	-7.75904268672539
H	14.05493550949544	2.86416932779252	-0.47428413833617
C	11.06242702417716	5.56766937159514	-5.13942189304310

H	14.60879742282640	4.28506548378813	-1.37709898881935
C	8.36569851145125	3.88428986426084	-8.62514707487843
H	11.19546401463523	4.92242569447210	-8.81468090939038
H	10.87473177488194	6.23782028024103	-5.98655826129359
H	12.03071183282765	5.79853178002500	-4.69821255844111
H	12.08233552129887	6.55490308524709	-10.42307976424447
C	6.27149248127218	1.42126225187599	-6.76763078768413
H	11.06383722737353	4.52843704082778	-5.48704584441454
C	7.36781278383869	1.98349657393420	-7.58318905081057
C	10.96237213893008	4.82505550149687	-9.86671208836158
H	14.90830079926418	6.22102802938135	-4.84259688313956
O	14.38890652392411	4.53896699432899	-3.89556510394009
C	14.18094008978782	2.58265944523671	-2.58953918185195
H	10.24615840610094	-0.94435184869390	-5.15350148293339
C	11.45606233235594	5.74461394553156	-10.77022280544639
C	14.86304377948641	5.15607665693411	-5.06896570384601
C	9.27824331841455	3.07987929356086	-9.30395427547536
H	11.26422261694336	-2.14985833073215	-4.32612662596593
H	14.08278386008329	0.77033711280660	-1.50057797075447
C	14.38168748098451	3.16742446497385	-3.84485668117953
C	11.08452704483238	-1.63969360662824	-5.27173981850833
C	6.19850904052855	1.63619209845972	-5.39550549777872
C	5.02035097319586	1.40402557593675	-4.71339246926044
C	10.11710858872874	3.80038422095086	-10.28353965972299
C	14.21934582960164	1.20140111135983	-2.48072139603805
H	14.18772170884995	4.99656548251737	-5.91636910351986
C	8.27646857692823	1.17958632644354	-8.25664904950597
H	10.83659513834182	-2.38068332397263	-6.03936908276238
C	11.10821264347519	5.67312889771657	-12.11885135284686
C	9.24999451378046	1.70171347374744	-9.10157389602773
H	15.86470595746273	4.79869335691771	-5.33027947477862
H	10.35883435174733	-0.54566735474068	-7.42468496867764
O	12.28368879647715	-0.96793743464791	-5.56967305289384
H	7.04890976706163	2.05534852374853	-4.87746841063152
C	14.56828141680167	2.34915007073343	-4.95031607539407
H	4.96100962631310	1.64885080158184	-3.66290719229970
C	14.43871174463647	0.38125086226917	-3.58277811329722
C	9.89171391559383	3.64269178532981	-11.64780824601205
C	11.31694221650639	-0.09640587892783	-7.62846765530718
H	8.20865328044619	0.10798439286521	-8.12467358890176
C	10.38183541618065	4.56436954109304	-12.55194412429054
C	12.36750803533093	-0.25835733245096	-6.73702517284856
H	14.69548118673039	2.77717695590849	-5.93219778939182
C	14.58975757738856	0.96700981184918	-4.84276045456663
O	14.53670167338309	-0.98391002687968	-3.51464180779595
C	10.27625278908719	0.79401693055821	-9.72947239140666
C	11.45529433528569	0.63675660397842	-8.79710595977557
H	9.25852534124915	2.83727128645240	-11.99355810987257
H	10.63105031809283	1.21310504060192	-10.66899415687245
C	13.60932824361298	0.31648085799986	-7.03189971431352
C	14.31688287144452	-1.61569399679385	-2.27733176212351
C	14.76551428127307	0.12763102019224	-6.08475751124884
H	13.31597871822449	-1.40295499891119	-1.88731375158544

H	9.83275155674527	-0.18698789989028	-9.91676583332086
H	15.06776572641904	-1.32362691152930	-1.53500132145102
C	12.68792538407473	1.22947195999748	-9.08398219707054
C	13.74597077396374	1.05359009701136	-8.19739617295468
H	14.81722714502502	-0.91926541254831	-5.78647916761063
H	14.40608370029021	-2.68335613343099	-2.47563761066801
H	15.69676213145406	0.40403567379584	-6.58467180270764
O	12.77349690264791	1.93757768308494	-10.25665516904737
H	14.71157505945897	1.48378623338055	-8.41521403540307
H	14.24303736783223	3.32832240934876	-9.79382357944294
C	13.97566222695610	2.59848299421257	-10.56498091885341
H	13.78579580763307	3.12256249234740	-11.50109960948980
H	14.80146731436258	1.89269844290848	-10.70480176107018
C	4.01940465955920	0.57774823316704	-6.72123059252294
C	3.88094321565863	0.96695780673441	-5.38780093952332
H	3.17907743983504	0.14029496654673	-7.24162485849982
H	10.12554160754447	4.47241579055557	-13.59792119339223

13. References

1 G. R. Fulmer, A. J. M. Miller, N. H. Sherden, H. E. Gottlieb, A. Nudelman, B. M. Stoltz, J. E. Bercaw and K. I. Goldberg, *Organometallics*, 2010, **29**, 2176–2179.

2 W. R. Kitzmann, J. Freudenthal, A.-P. M. Reponen, Z. A. VanOrman and S. Feldmann, *Advanced Materials*, 2023, **35**, 2302279.

3 A.-P. M. Reponen, M. Mattes, Z. A. VanOrman, L. Estaque, G. Pieters and S. Feldmann, *Nature*, 2025, **643**, 675–682.

4 C. Bannwarth, E. Caldeweyher, S. Ehlert, A. Hansen, P. Pracht, J. Seibert, S. Spicher and S. Grimme, *WIREs Computational Molecular Science*, 2021, **11**, e1493.

5 C. Bannwarth, S. Ehlert and S. Grimme, *J. Chem. Theory Comput.*, 2019, **15**, 1652–1671.

6 S. Ehlert, M. Stahn, S. Spicher and S. Grimme, *J. Chem. Theory Comput.*, 2021, **17**, 4250–4261.

7 F. Neese, *WIREs Computational Molecular Science*, 2022, **12**, e1606.

8 C. Bannwarth and S. Grimme, *Computational and Theoretical Chemistry*, 2014, **1040–1041**, 45–53.

9 J.-D. Chai and M. Head-Gordon, *J. Chem. Phys.*, DOI:10.1063/1.2834918.

10 F. Weigend and R. Ahlrichs, *Phys. Chem. Chem. Phys.*, 2005, **7**, 3297–3305.

11 T. Bruhn, A. Schaumlöffel, Y. Hemberger and G. Bringmann, *Chirality*, 2013, **25**, 243–249.

12 T. Boinski and A. Szumna, *Tetrahedron*, 2012, **68**, 9419–9422.

13 C. Xie, W. Hu, W. Hu, Y. A. Liu, J. Huo, J. Li, B. Jiang and K. Wen, *Chinese Journal of Chemistry*, 2015, **33**, 379–383.

14 G. Wang, H. Qiang, Y.-Z. Guo, J. Yang, K. Wen and W.-B. Hu, *Organic & Biomolecular Chemistry*, 2019, **17**, 4600–4604.

15 N. L. Strutt, D. Fairen-Jimenez, J. Iehl, M. B. Lalonde, R. Q. Snurr, O. K. Farha, J. T. Hupp and J. F. Stoddart, *J. Am. Chem. Soc.*, 2012, **134**, 17436–17439.

16 Crysalispro, 1.171.44.85, 2024, Rigaku OD.

17 G. M. Sheldrick, *Acta Cryst A*, 2015, **71**, 3–8.

18 G. M. Sheldrick, *Acta Cryst C*, 2015, **71**, 3–8.

19 C. R. Groom, I. J. Bruno, M. P. Lightfoot and S. C. Ward, *Acta Cryst B*, 2016, **72**, 171–179.

20 D. Kratzert, FinalCif, V156 https://dkratzert.de/finalcif.html.

21 C. F. Macrae, I. Sovago, S. J. Cottrell, P. T. A. Galek, P. McCabe, E. Pidcock, M. Platings, G. P. Shields, J. S. Stevens, M. Towler and P. A. Wood, *J Appl Cryst*, 2020, **53**, 226–235.
